# Supplementary material for: Calcium signaling from damaged lysosomes induces cytoprotective stress granules
Source: EMBO J. 2024 Nov 12;43(24):6410–43. doi: 10.1038/s44318-024-00292-1 (PMC11649789; doi:10.1038/s44318-024-00292-1)
Supplement: Supplementary file 1 — Appendix [file 44318_2024_292_MOESM1_ESM.pdf]

Appendix for

Calcium signaling from damaged lysosomes induces cytoprotective stress granules

Jacob Duran[1,2†], Jay E. Salinas[1,2†], Rui ping Wheaton[1,2†], Suttinee Poolsup[1,2], Lee Allers[2,3], Monica Rosas-Lemus[2,3], Li Chen[2], Qiuying Cheng[1], Jing Pu[3], Michelle Salemi[5], Brett Phinney[5], Pavel Ivanov[6], Alf Håkon Lystad[7], Kiran Bhaskar[3,4], Jaya Rajaiya[3], Douglas J. Perkins[1], Jingyue Jia[1,2,8*]

[1]Center for Global Health, Department of Internal Medicine, University of New Mexico Health Sciences Center, Albuquerque, NM 87106, USA
[2]Autophagy, Inflammation and Metabolism Center of Biochemical Research Excellence, Albuquerque, NM 87106, USA
[3]Department of Molecular Genetics and Microbiology, University of New Mexico Health Sciences Center, Albuquerque, NM 87106, USA
[4]Department of Neurology, University of New Mexico Health Sciences Center, Albuquerque, NM 87106, USA
[5]Proteomics Core Facility, University of California Davis Genome Center, University of California, Davis, CA 95616, USA
[6]Department of Medicine, Brigham and Women's Hospital and Harvard Medical School; HMS Initiative for RNA Medicine, Boston, MA 02115, USA
[7]Centre for Cancer Cell Reprogramming, University of Oslo; Department of Molecular Cell Biology, Institute for Cancer Research, Oslo University Hospital, Oslo, Norway
[8]Lead Contact
[†]These authors contributed equally to this work

*Correspondence:
Jingyue Jia, Ph.D.
Center for Global Health, Department of Internal Medicine
University of New Mexico Health Sciences Center
700 Camino de Salud, NE
Albuquerque, NM 87106, USA
JJia@salud.unm.edu

Table of Contents

Appendix Figure S1: Stress granules are important for cell survival in response to lysosomal damage in disease states ……………………………………………………………………2-3

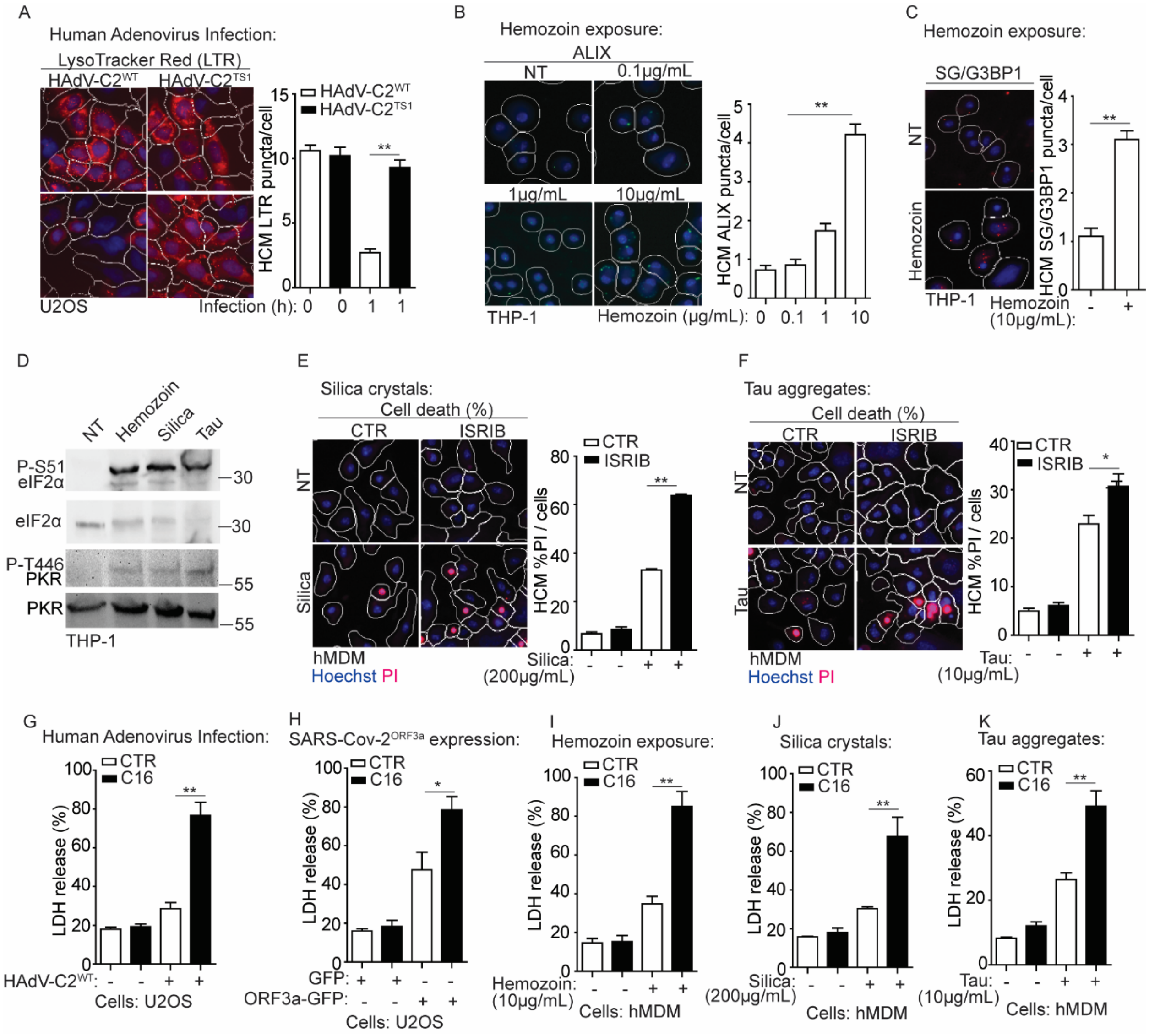

Appendix Figure S1. Stress granules are important for cell survival in response to lysosomal damage in disease states. (A) Quantification by HCM of status of acidified organelles assessed by LysoTracker Red (LTR) in U2OS cells infected with wildtype human adenovirus C2 (HAdV-C2^{WT}) or C2 TS1 mutant (HAdV-C2^{TS1}) at MOI=10 for 1h. White masks, algorithm-defined cell boundaries; red masks, computer-identified LTR puncta. (B) Quantification by HCM of ALIX puncta in THP-1 cells treated with hemozoin for 4h at the indicated dose. White masks, algorithm-defined cell boundaries; green masks, computer-identified ALIX puncta. (C) Quantification by HCM of G3BP1 puncta in THP-1 cells treated with 10 µg/ml hemozoin for 4h. White masks, algorithm-defined cell boundaries; red masks, computer-identified G3BP1 puncta. (D) Immunoblot analysis of phosphorylation of eIF2α (S51) and PKR (T446) in THP-1 cells treated with 10 µg/ml hemozoin, 200 µg/mL silica or 10 µg/mL tau oligomer for 4h. (E) Quantification by HCM of cell death by a propidium iodide (PI) uptake assay in human peripheral blood monocyte-derived macrophages (hMDM) during silica treatment. Cells were treated with 200 µg/mL silica for 4h in the presence or absence of 100 nM ISRIB, and then stained with propidium iodide PI (dead cells) and Hoechst-33342 (total cells). White masks, algorithm-defined cell boundaries; red

masks, computer-identified PI+ nuclei. (F) Quantification by HCM of cell death by a propidium iodide (PI) uptake assay in human peripheral blood monocyte-derived macrophages (hMDM) during the treatment of tau oligomer. Cells were treated with 10 µg/mL tau oligomer for 4h in the presence or absence of 100 nM ISRIB, and then stained with propidium iodide PI (dead cells) and Hoechst-33342 (total cells). (G) Cell death analysis of supernatants of U2OS cells by a LDH release assay during human adenovirus C2 infection. Cells were infected with wildtype human adenovirus C2 (HAdV-C2^{WT}) at MOI=10 for 1h in the presence or absence of 210 nM imidazolo-oxindole C16. (H) Cell death analysis of supernatants of U2OS cells by a LDH release assay during SARS-Cov-2^{ORF3a} expression. Cells were transfected with the GFP-SARS-Cov-2^{ORF3a} construct overnight in the presence or absence of 210 nM imidazolo-oxindole C16. (I) Cell death analysis of supernatants of human peripheral blood monocyte-derived macrophages (hMDM) by a LDH release assay during hemozoin exposure. Cells were treated with 10 µg/ml hemozoin for 4h in the presence or absence of 210 nM imidazolo-oxindole C16. (J) Cell death analysis of supernatants of human peripheral blood monocyte-derived macrophages (hMDM) by a LDH release assay during silica treatment. Cells were treated with 200 µg/mL silica for 4h in the presence or absence of 210 nM imidazolo-oxindole C16. (K) Cell death analysis of supernatants of human peripheral blood monocyte-derived macrophages (hMDM) by a LDH release assay during the treatment of tau oligomer. Cells were treated with 10 µg/mL tau oligomer for 4h in the presence or absence of 210 nM imidazolo-oxindole C16. White masks, algorithm-defined cell boundaries; red masks, computer-identified PI+ nuclei. CTR, control; NT, untreated cells. Data, means ± SEM (n = 3); HCM: n ≥ 3 (each experiment: 500 valid primary objects/cells per well, ≥5 wells/sample). *$p < 0.05$, **$p < 0.01$, ANOVA. See also Figure 7.
