# Supplementary figures and images for: Calcium signaling from damaged lysosomes induces cytoprotective stress granules (part 1 of 3)
Source: EMBO J. 2024 Nov 12;43(24):6410–43. doi: 10.1038/s44318-024-00292-1 (PMC11649789; doi:10.1038/s44318-024-00292-1)

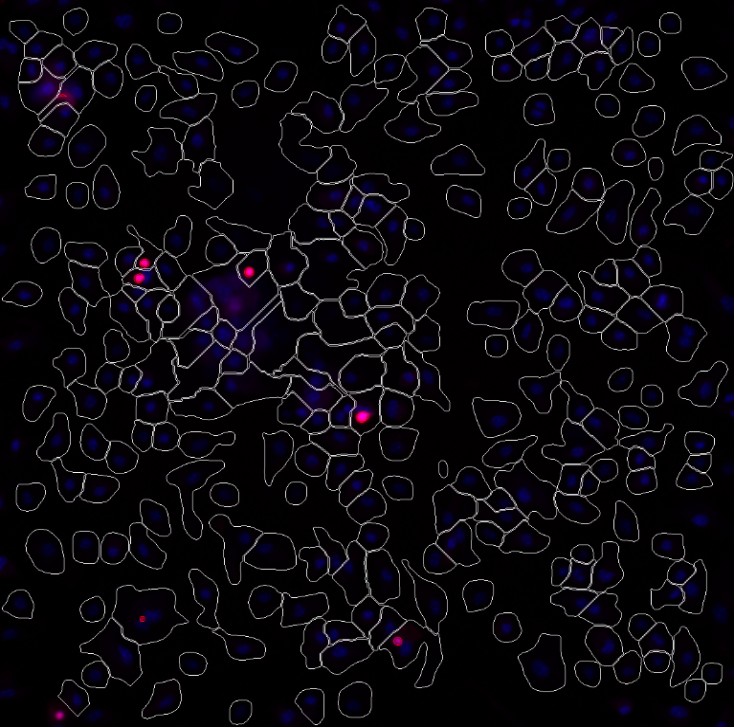

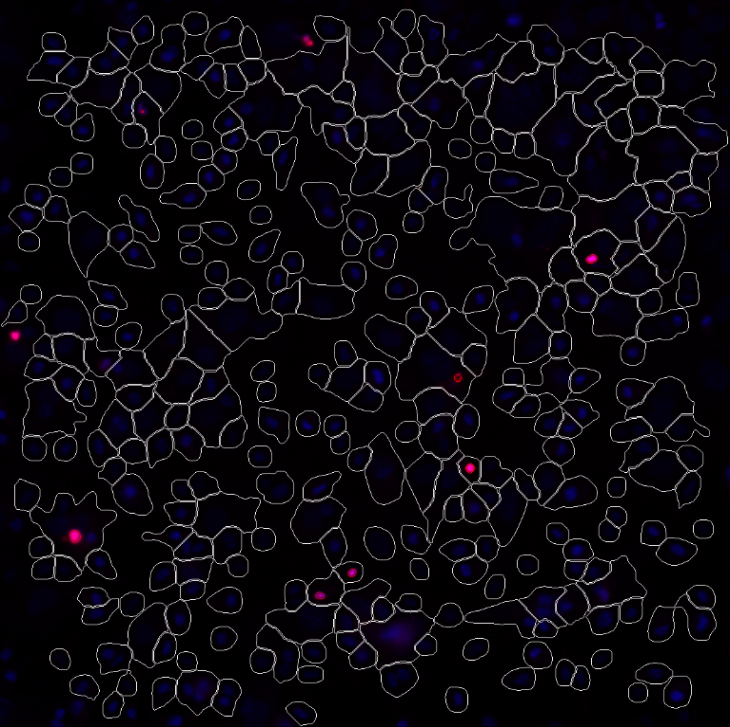

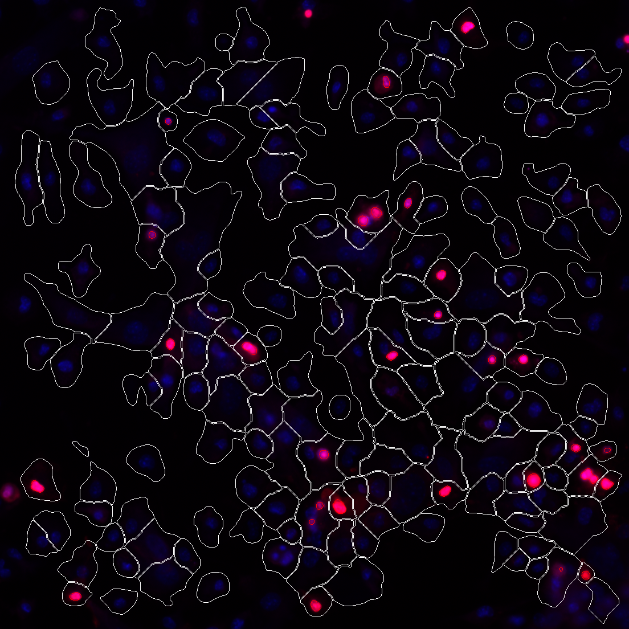

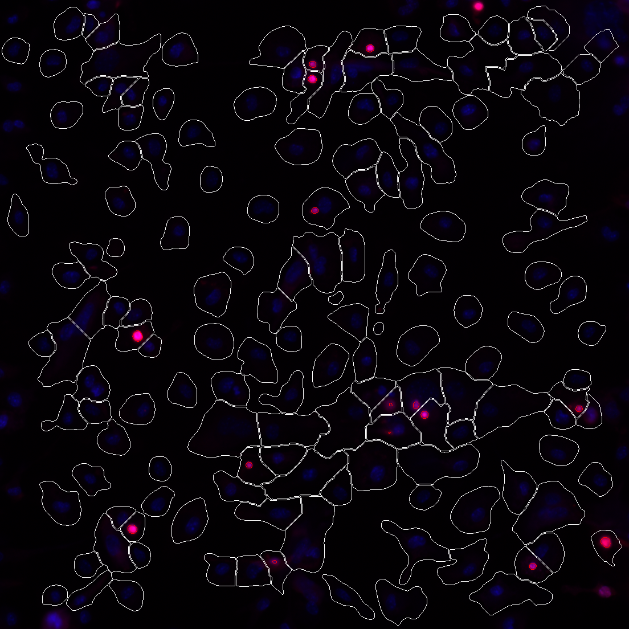


ΔΔG3BP1/2 -NT

ΔΔG3BP1/2 -LLOMe

WT-LLOME

WT-NT

Supplement: Supplementary file 4 — Source data Fig. 1 [file 44318_2024_292_MOESM4_ESM.zip › Figure 1/1A/README.docx]

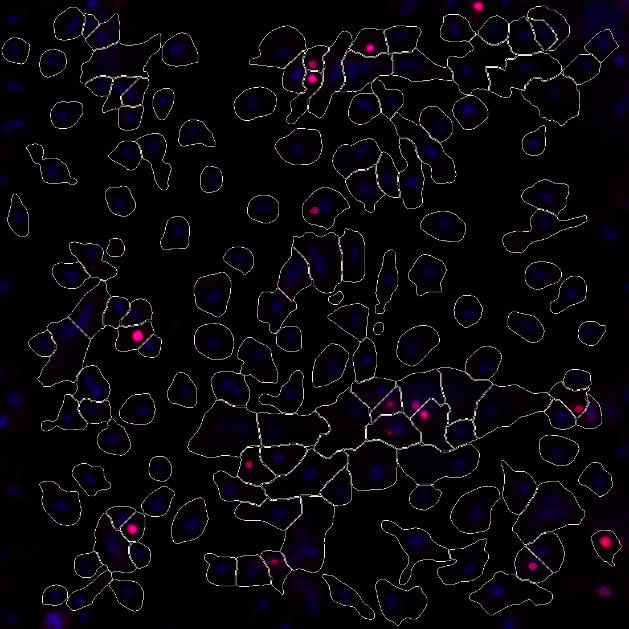

Supplement: Supplementary file 4 — Source data Fig. 1 [file 44318_2024_292_MOESM4_ESM.zip › Figure 1/1A/WT-LLOMe.tif]

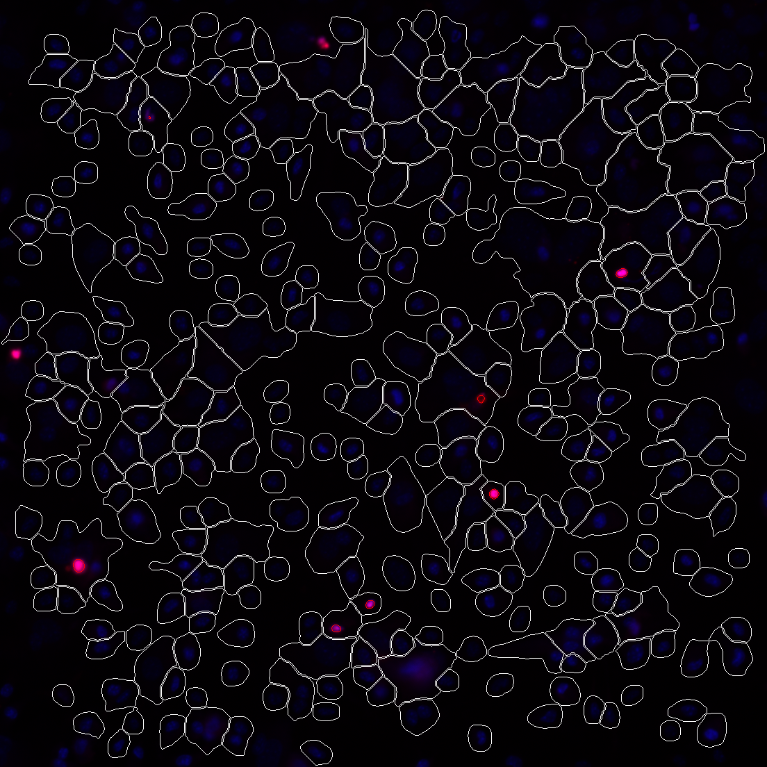

Supplement: Supplementary file 4 — Source data Fig. 1 [file 44318_2024_292_MOESM4_ESM.zip › Figure 1/1A/WT-NT.tif]

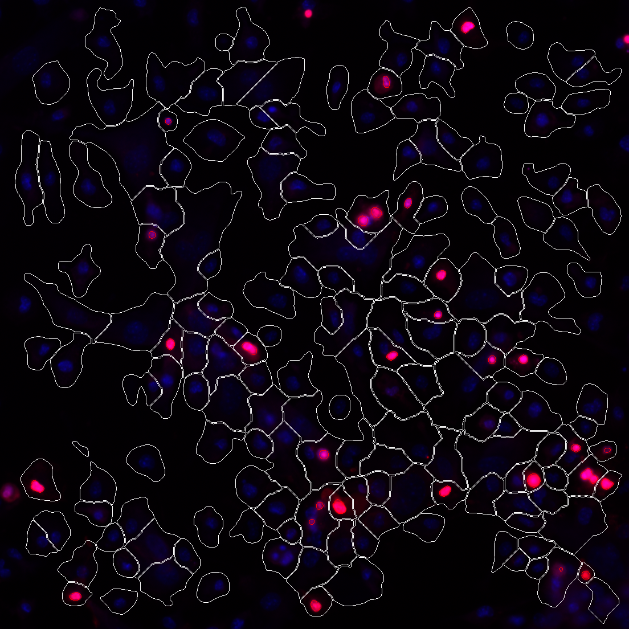

Supplement: Supplementary file 4 — Source data Fig. 1 [file 44318_2024_292_MOESM4_ESM.zip › Figure 1/1A/ΔΔG3BP-LLOMe.tif]

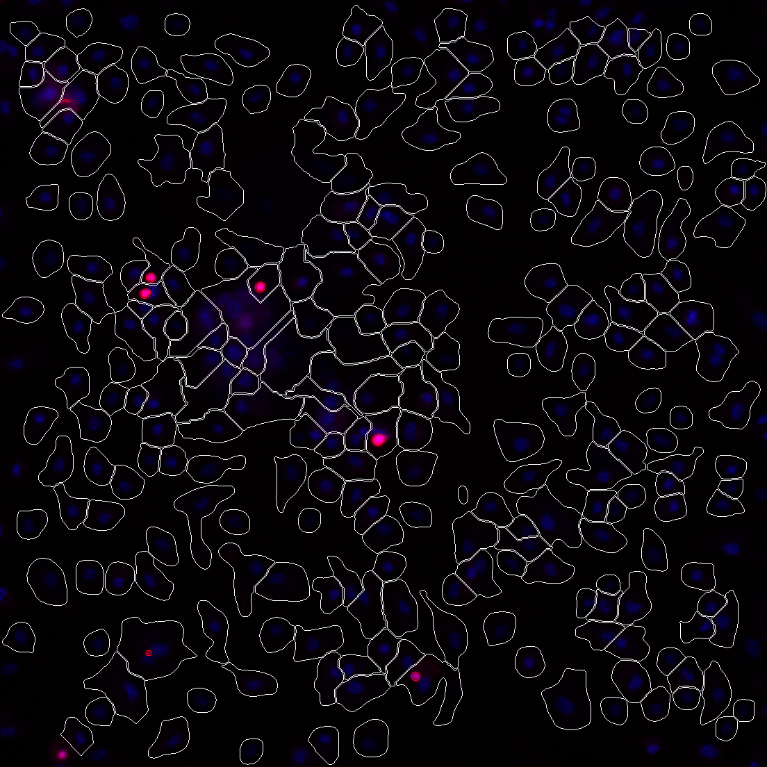

Supplement: Supplementary file 4 — Source data Fig. 1 [file 44318_2024_292_MOESM4_ESM.zip › Figure 1/1A/ΔΔG3BP-NT.tif]

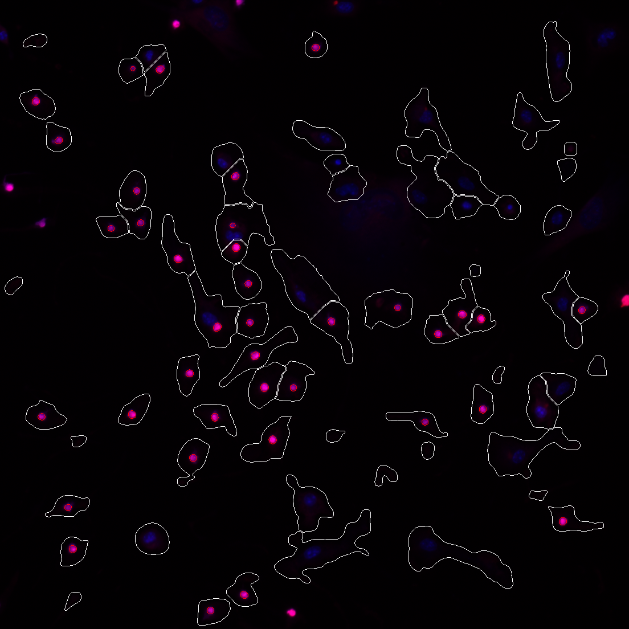

Supplement: Supplementary file 4 — Source data Fig. 1 [file 44318_2024_292_MOESM4_ESM.zip › Figure 1/1C/CHX-LLOMe.tif]

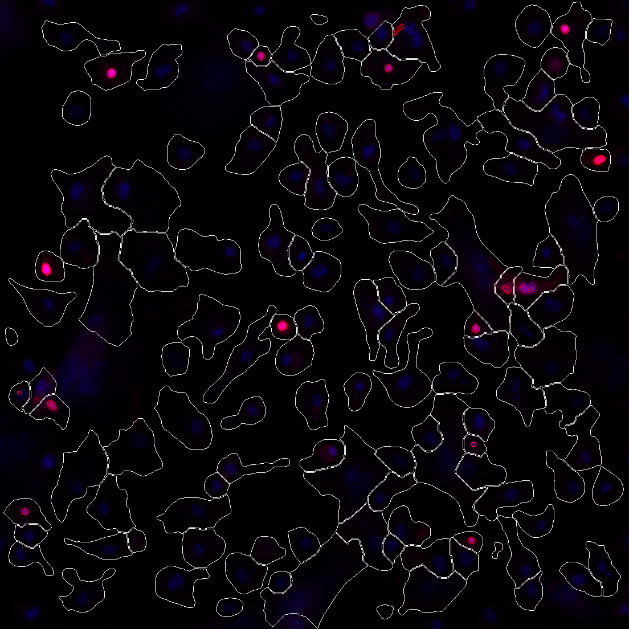

Supplement: Supplementary file 4 — Source data Fig. 1 [file 44318_2024_292_MOESM4_ESM.zip › Figure 1/1C/CHX-NT.tif]

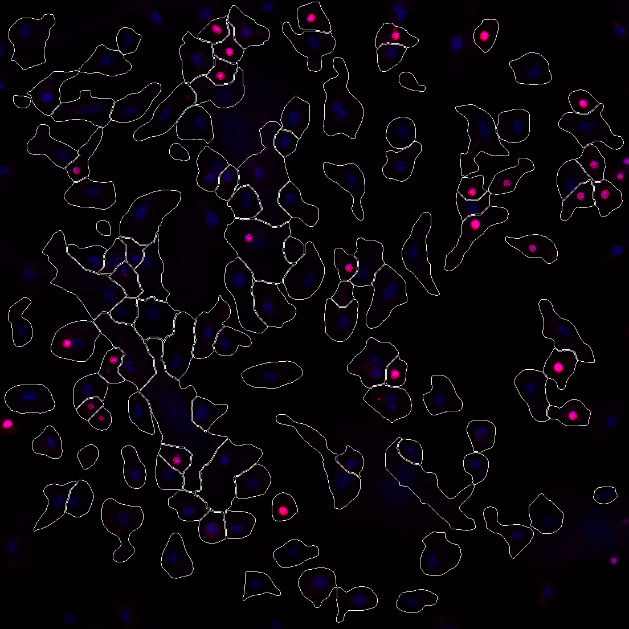

Supplement: Supplementary file 4 — Source data Fig. 1 [file 44318_2024_292_MOESM4_ESM.zip › Figure 1/1C/CTR-LLOMe.tif]

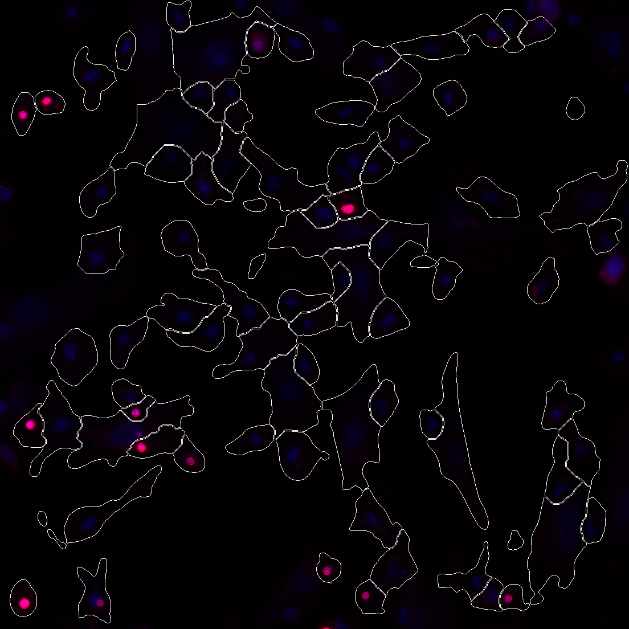

Supplement: Supplementary file 4 — Source data Fig. 1 [file 44318_2024_292_MOESM4_ESM.zip › Figure 1/1C/CTR-NT.tif]

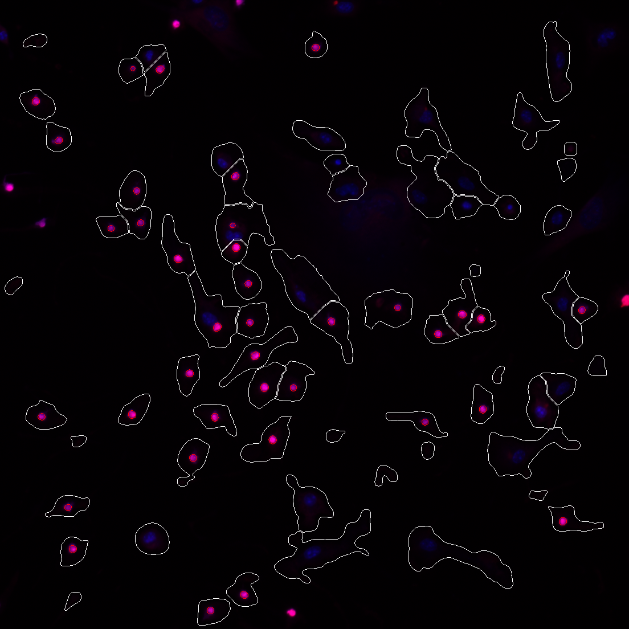

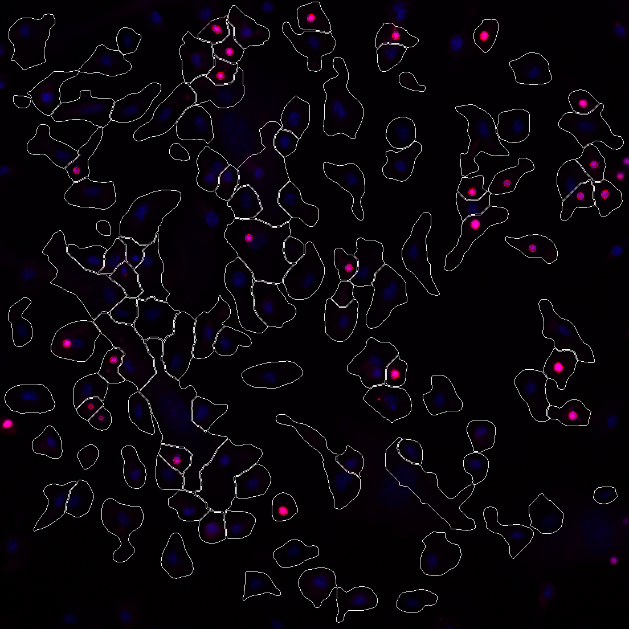

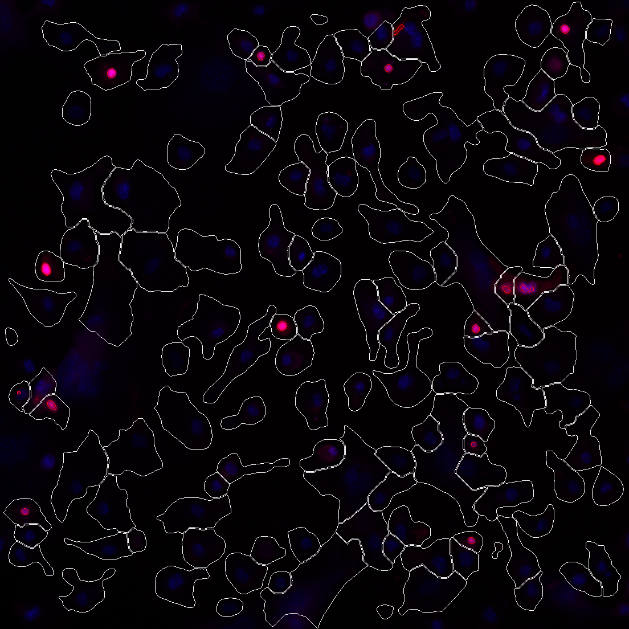

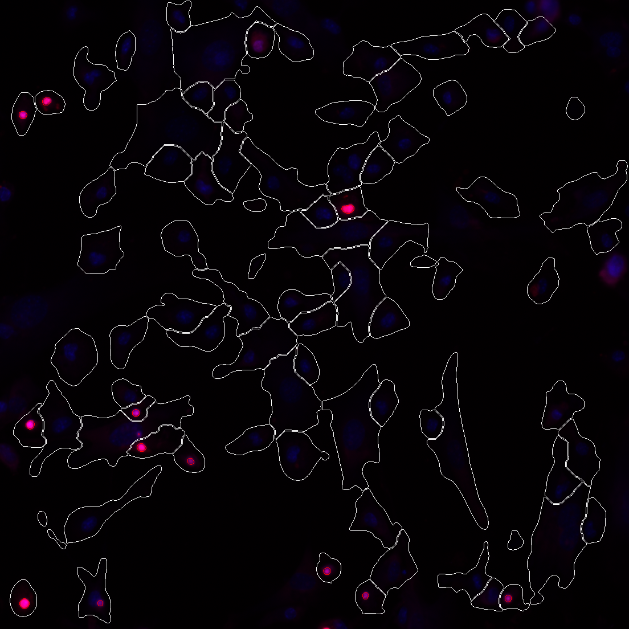


CHX -LLOMe

CHX -NT

CTR-LLOME

CTR-NT

Supplement: Supplementary file 4 — Source data Fig. 1 [file 44318_2024_292_MOESM4_ESM.zip › Figure 1/1C/README.docx]

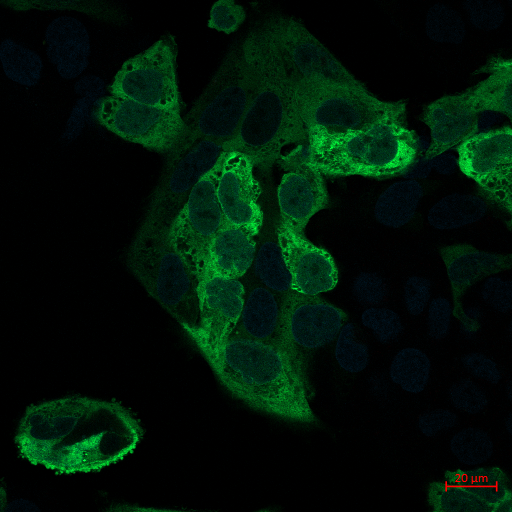

Supplement: Supplementary file 4 — Source data Fig. 1 [file 44318_2024_292_MOESM4_ESM.zip › Figure 1/1D/CHX-LLOMe.tif]

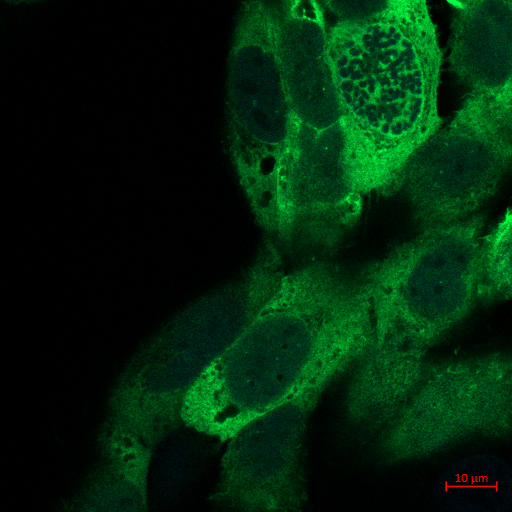

Supplement: Supplementary file 4 — Source data Fig. 1 [file 44318_2024_292_MOESM4_ESM.zip › Figure 1/1D/CHX-NT.tif]

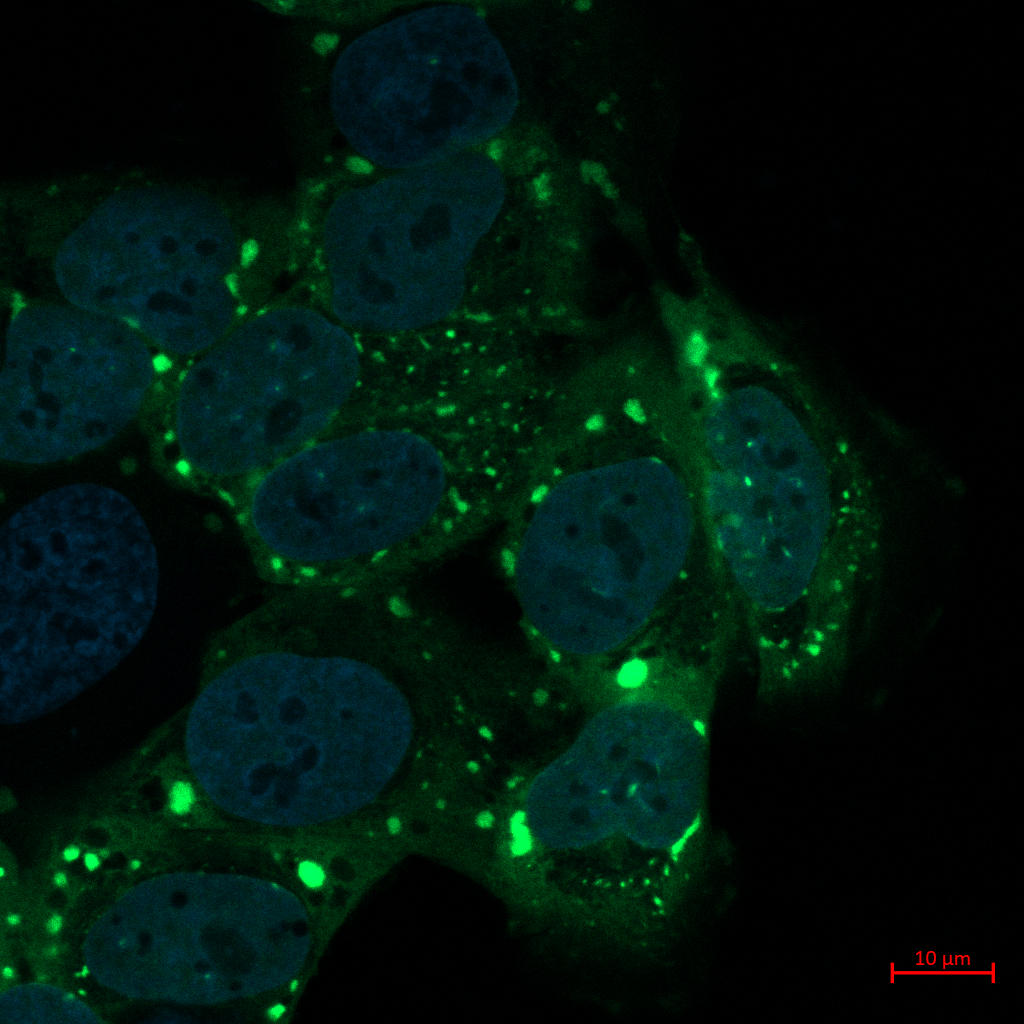

Supplement: Supplementary file 4 — Source data Fig. 1 [file 44318_2024_292_MOESM4_ESM.zip › Figure 1/1D/CTR-LLOMe.tif]

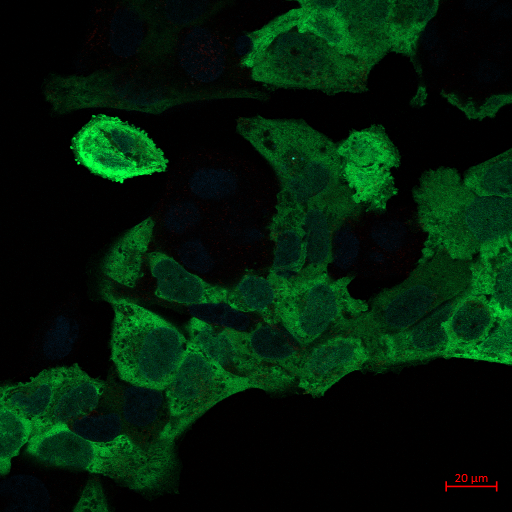

Supplement: Supplementary file 4 — Source data Fig. 1 [file 44318_2024_292_MOESM4_ESM.zip › Figure 1/1D/CTR-NT.tif]

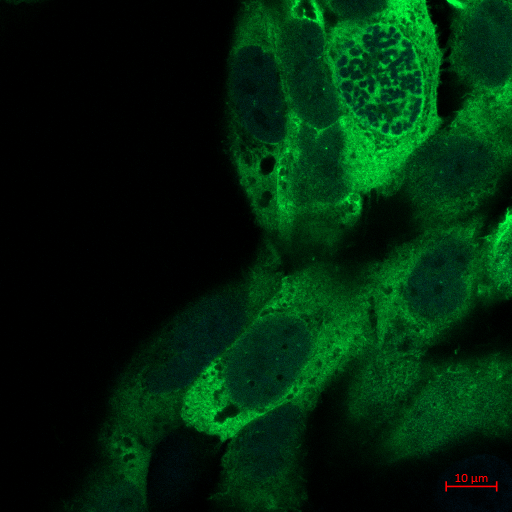

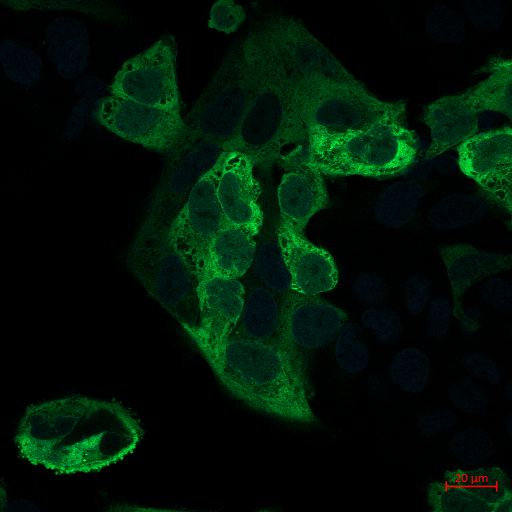

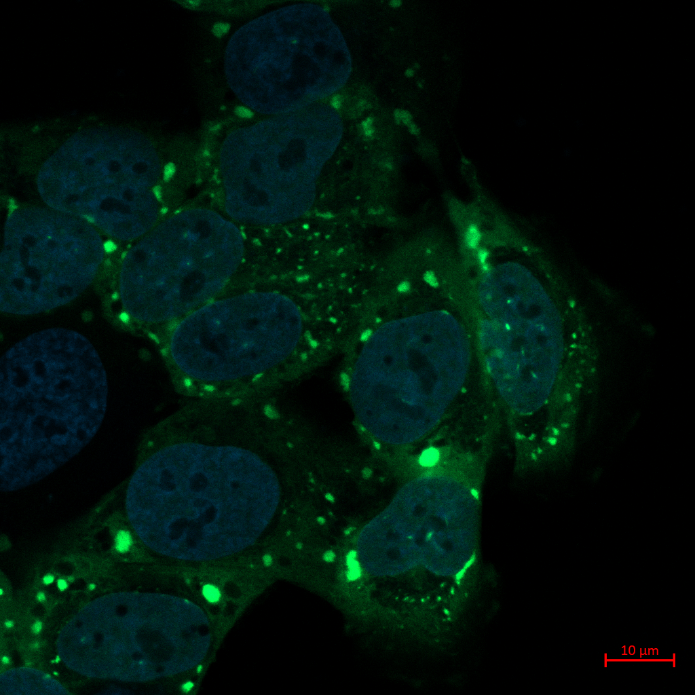

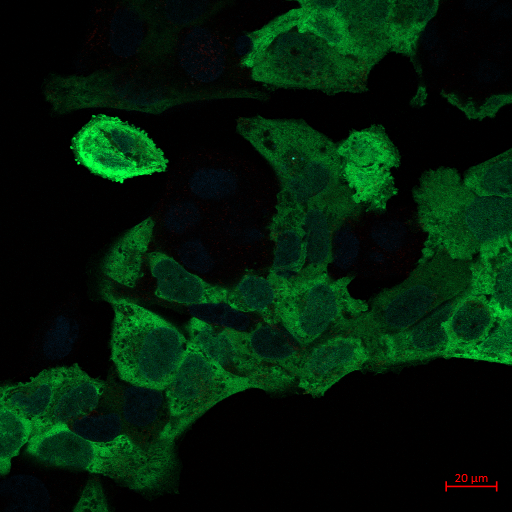


CHX -NT

CHX -LLOMe

CTR-LLOME

CTR-NT

Supplement: Supplementary file 4 — Source data Fig. 1 [file 44318_2024_292_MOESM4_ESM.zip › Figure 1/1D/README.docx]

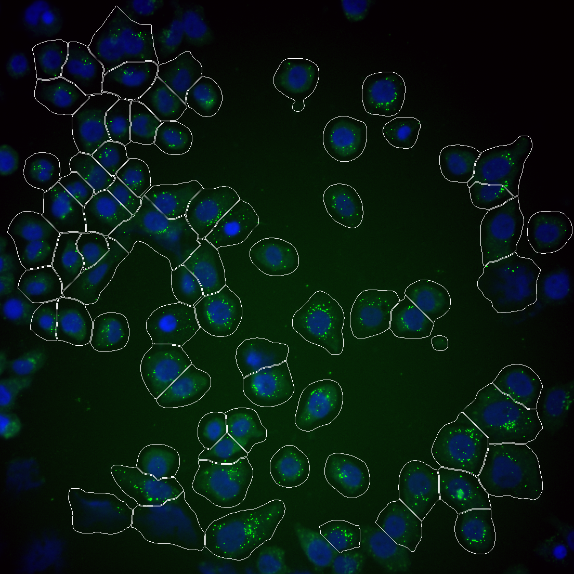

Supplement: Supplementary file 4 — Source data Fig. 1 [file 44318_2024_292_MOESM4_ESM.zip › Figure 1/1F/G3BPDKD-LLOMe-eIF4G.tif]

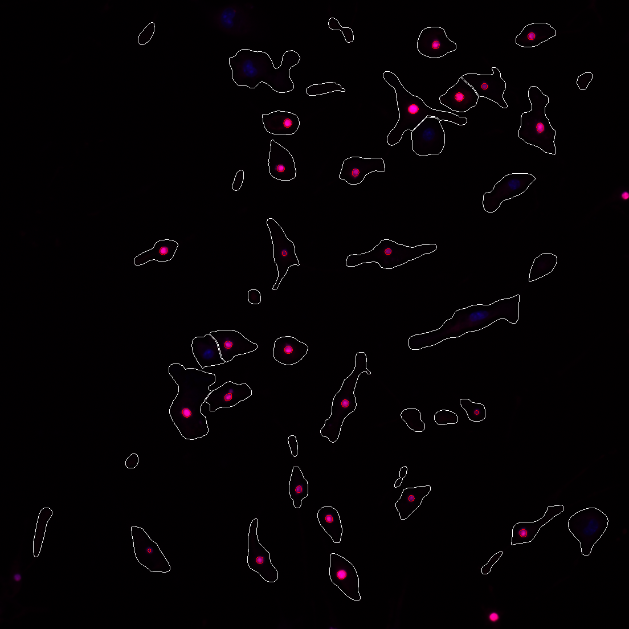

Supplement: Supplementary file 4 — Source data Fig. 1 [file 44318_2024_292_MOESM4_ESM.zip › Figure 1/1F/G3BPDKD-LLOMe-PI.tif]

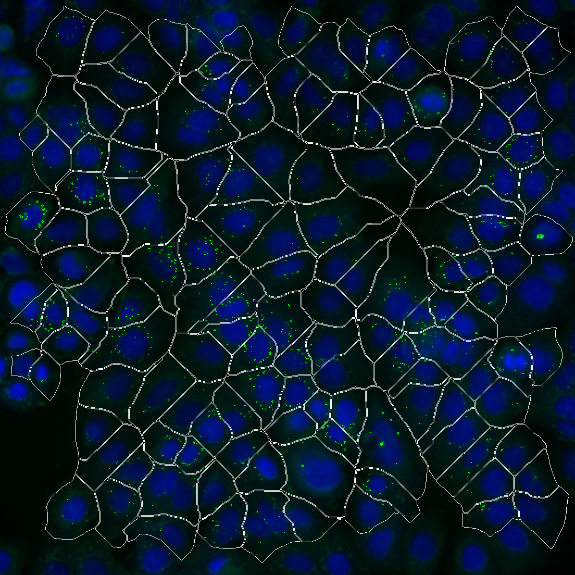

Supplement: Supplementary file 4 — Source data Fig. 1 [file 44318_2024_292_MOESM4_ESM.zip › Figure 1/1F/G3BPDKD-NT-eIF4G.tif]

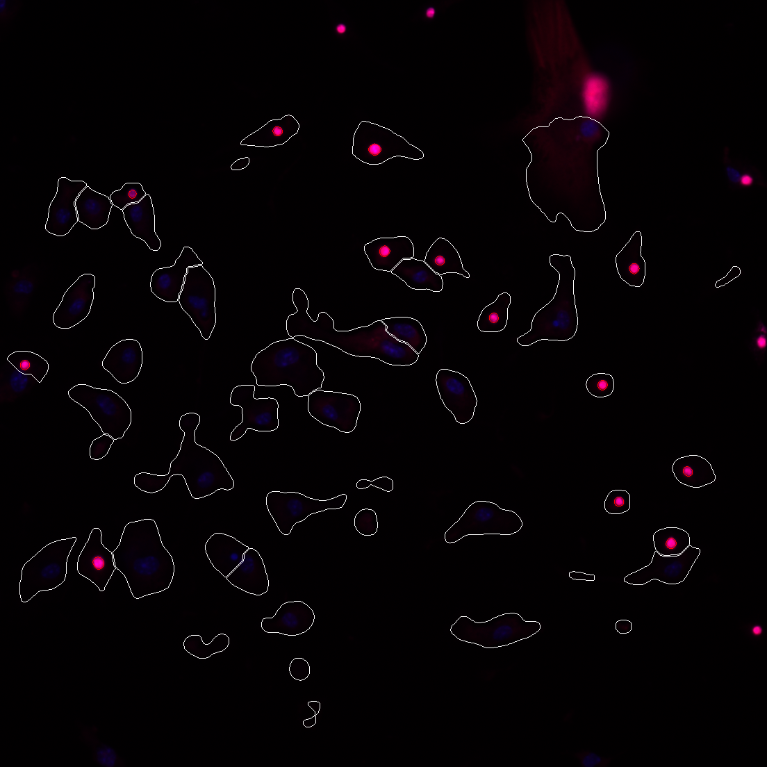

Supplement: Supplementary file 4 — Source data Fig. 1 [file 44318_2024_292_MOESM4_ESM.zip › Figure 1/1F/G3BPDKD-NT-PI.tif]

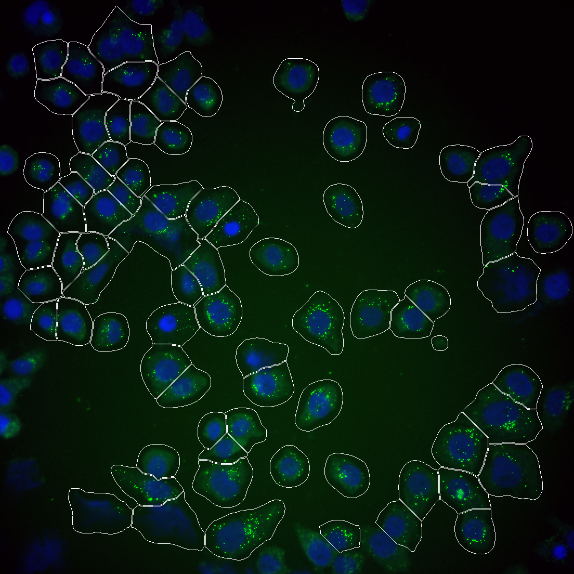

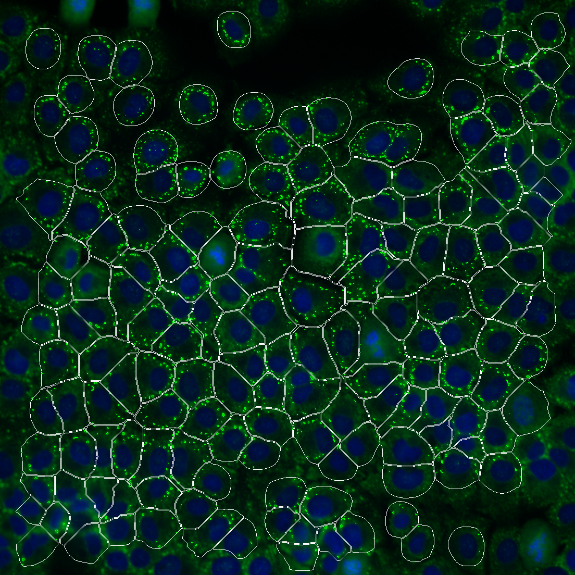

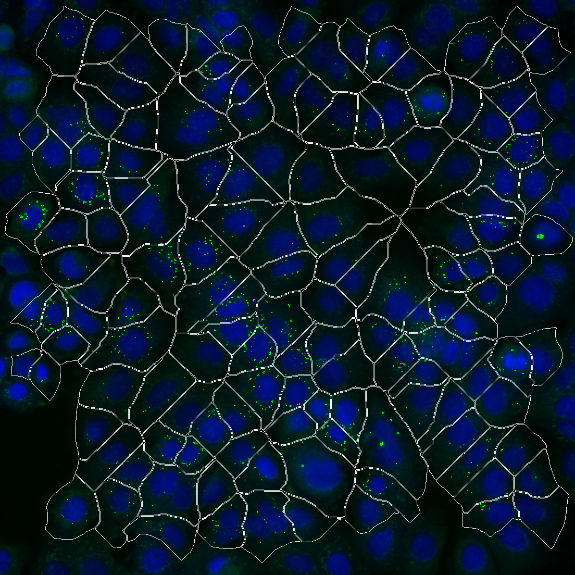

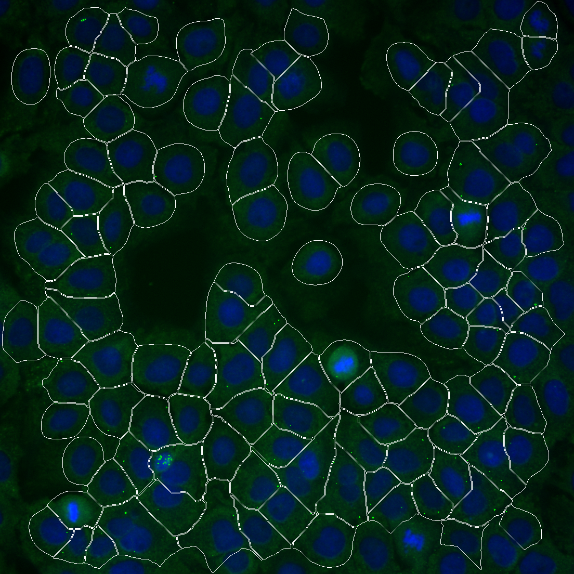
 SG/eIF4G:

G3BP1/2DKD-NT

SCR-NT

G3BP1/2DKD-LLOMe

SCR-LLOMe


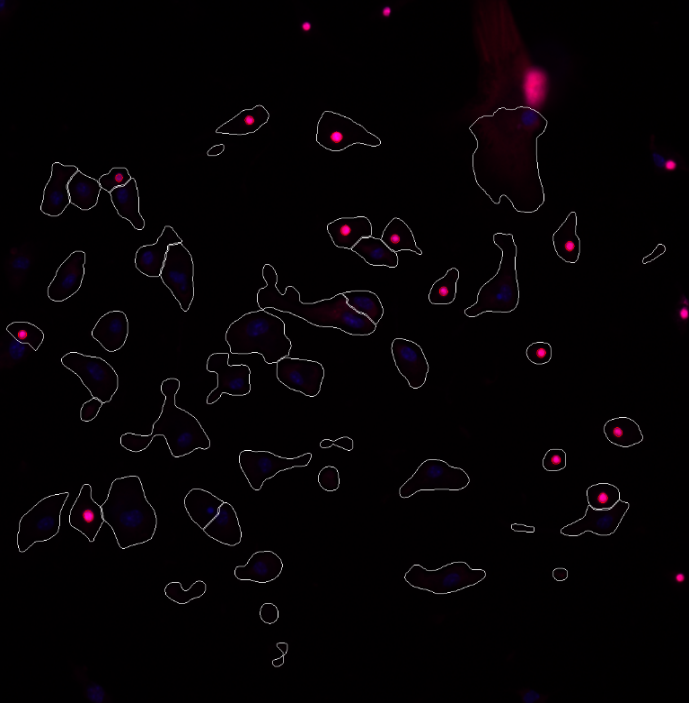

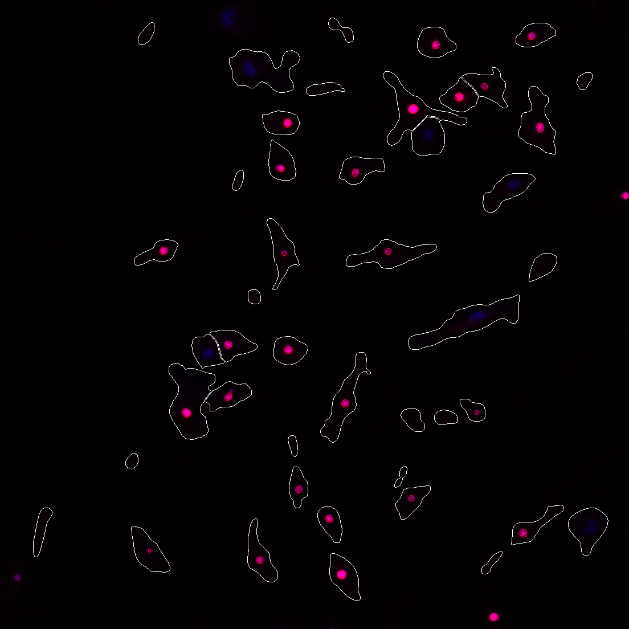

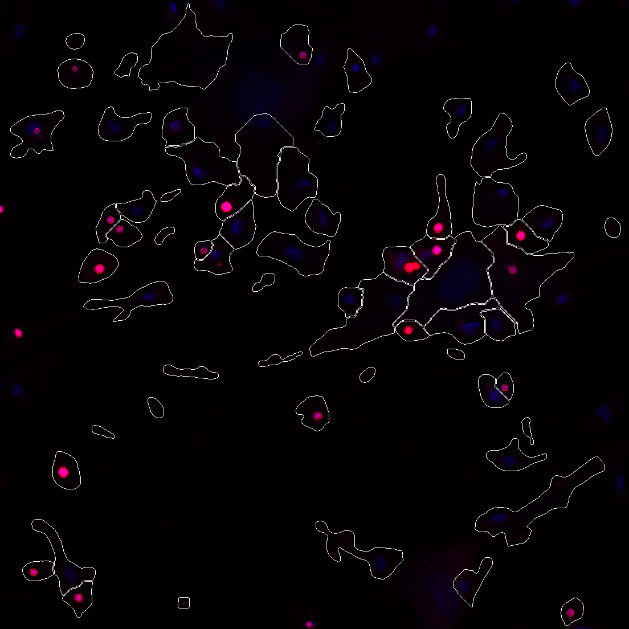

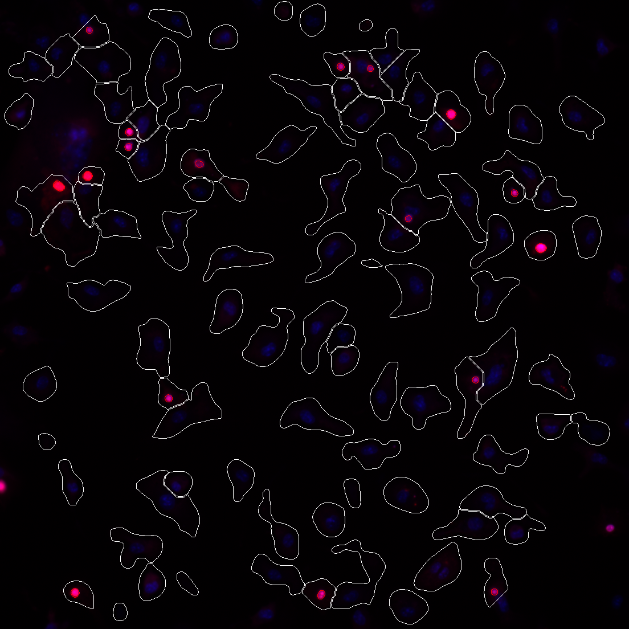
PI:

G3BP1/2DKD-LLOMe

SCR-LLOMe

G3BP1/2DKD-NT

SCR-NT

Supplement: Supplementary file 4 — Source data Fig. 1 [file 44318_2024_292_MOESM4_ESM.zip › Figure 1/1F/README.docx]

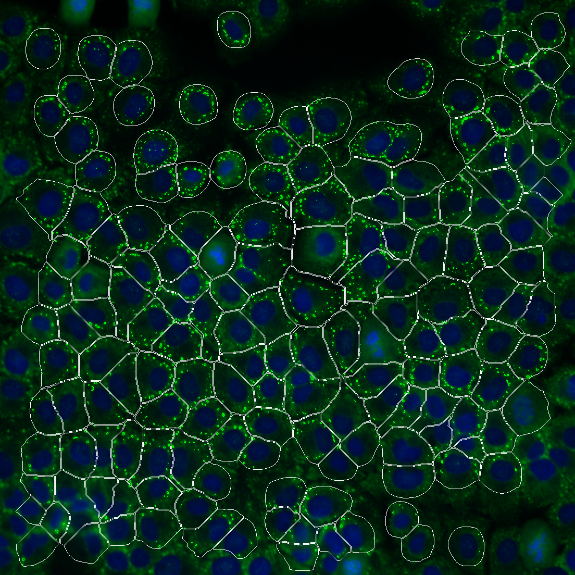

Supplement: Supplementary file 4 — Source data Fig. 1 [file 44318_2024_292_MOESM4_ESM.zip › Figure 1/1F/SCR-LLOMe-eIF4G.tif]

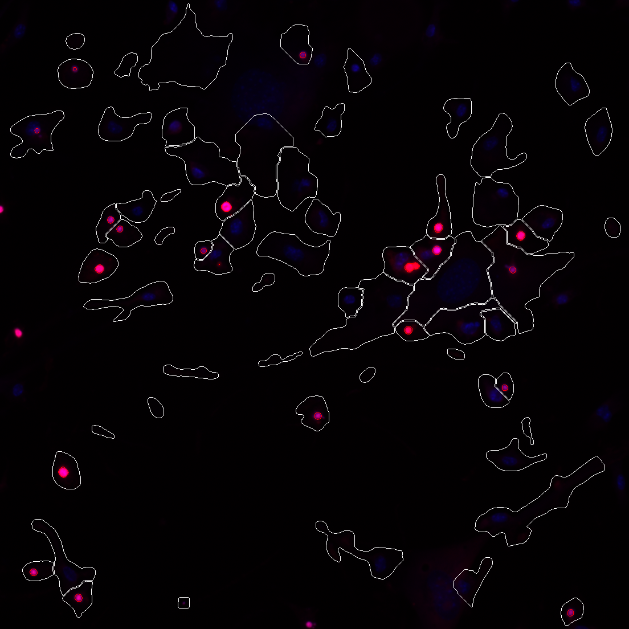

Supplement: Supplementary file 4 — Source data Fig. 1 [file 44318_2024_292_MOESM4_ESM.zip › Figure 1/1F/SCR-LLOMe-PI.tif]

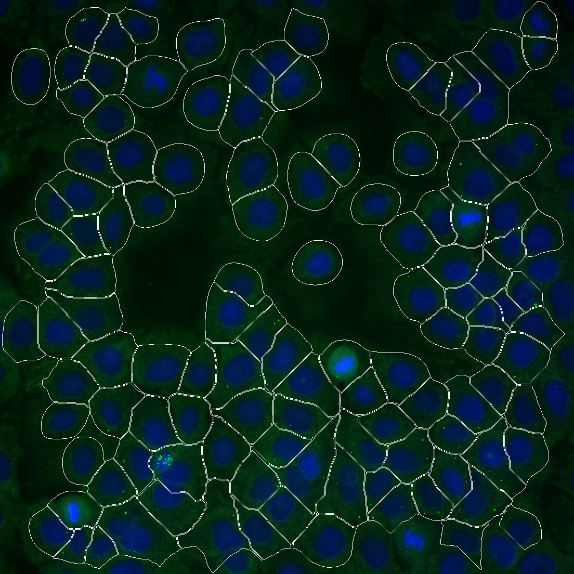

Supplement: Supplementary file 4 — Source data Fig. 1 [file 44318_2024_292_MOESM4_ESM.zip › Figure 1/1F/SCR-NT-eIF4G.tif]

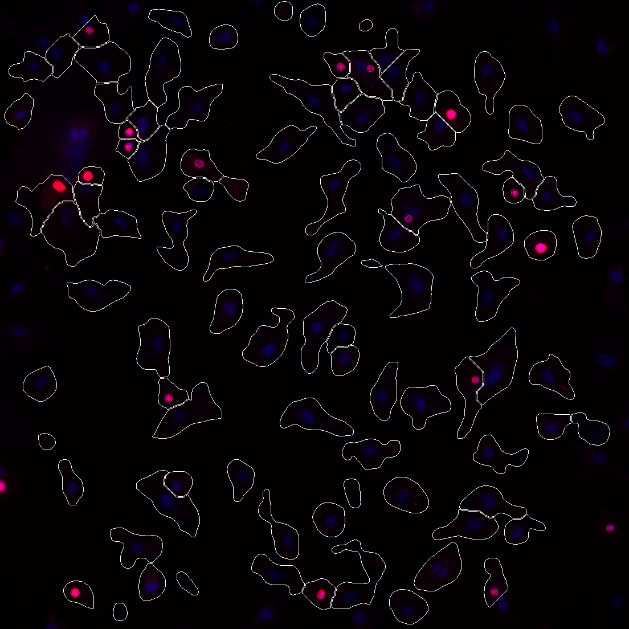

Supplement: Supplementary file 4 — Source data Fig. 1 [file 44318_2024_292_MOESM4_ESM.zip › Figure 1/1F/SCR-NT-PI.tif]

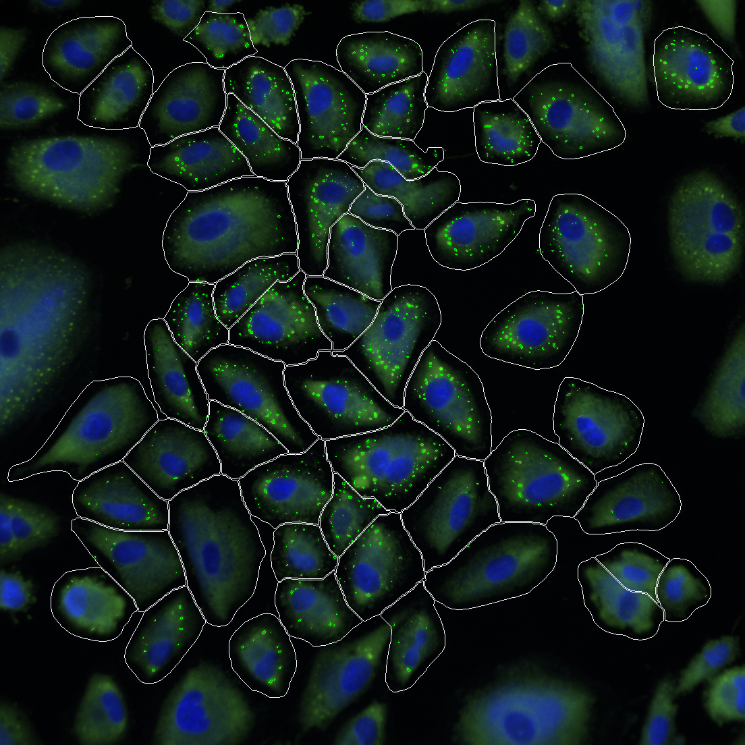

Supplement: Supplementary file 4 — Source data Fig. 1 [file 44318_2024_292_MOESM4_ESM.zip › Figure 1/1H/CTR-LLOMe.tif]

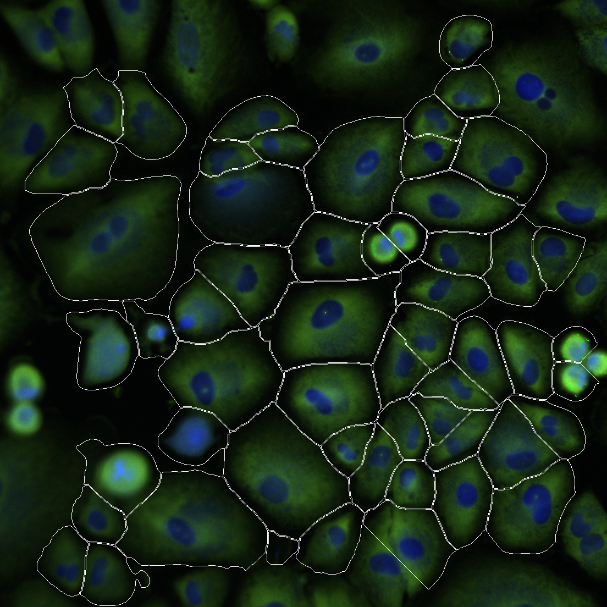

Supplement: Supplementary file 4 — Source data Fig. 1 [file 44318_2024_292_MOESM4_ESM.zip › Figure 1/1H/CTR-NT.tif]

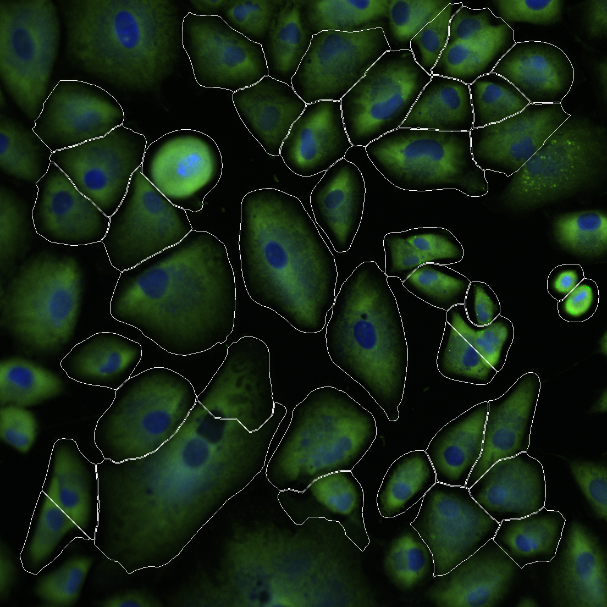

Supplement: Supplementary file 4 — Source data Fig. 1 [file 44318_2024_292_MOESM4_ESM.zip › Figure 1/1H/FAZ3532-LLOMe.tif]

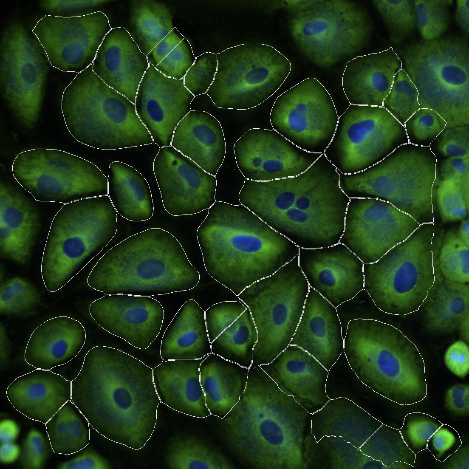

Supplement: Supplementary file 4 — Source data Fig. 1 [file 44318_2024_292_MOESM4_ESM.zip › Figure 1/1H/FAZ3532-NT.tif]

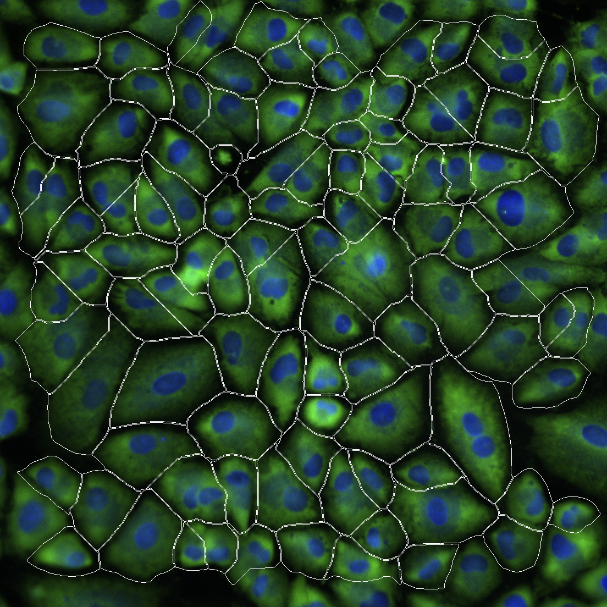

Supplement: Supplementary file 4 — Source data Fig. 1 [file 44318_2024_292_MOESM4_ESM.zip › Figure 1/1H/FAZ3780-LLOMe.tif]

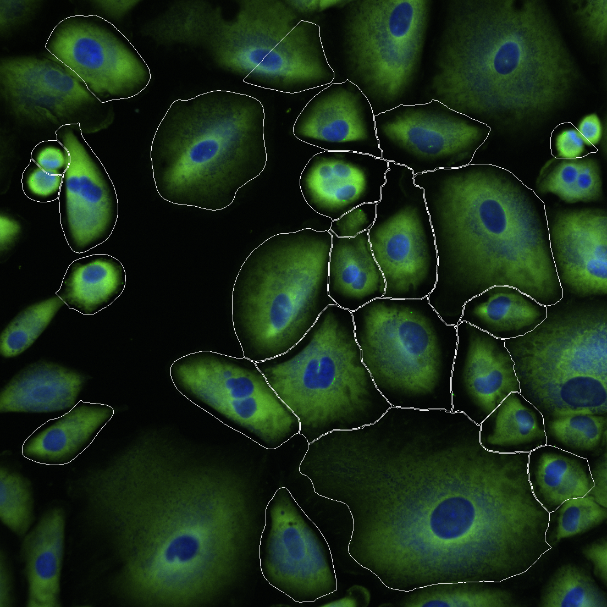

Supplement: Supplementary file 4 — Source data Fig. 1 [file 44318_2024_292_MOESM4_ESM.zip › Figure 1/1H/FAZ3780-NT.tif]

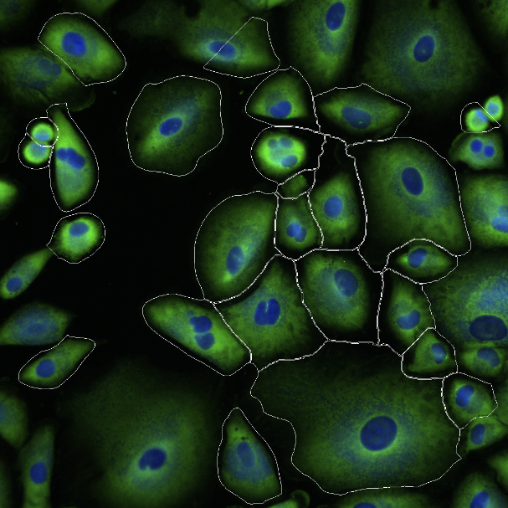

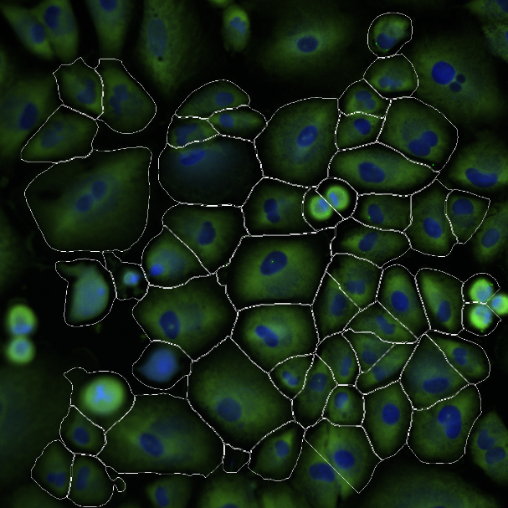


FAZ3780-NT

FAZ3532-NT

CTR-NT


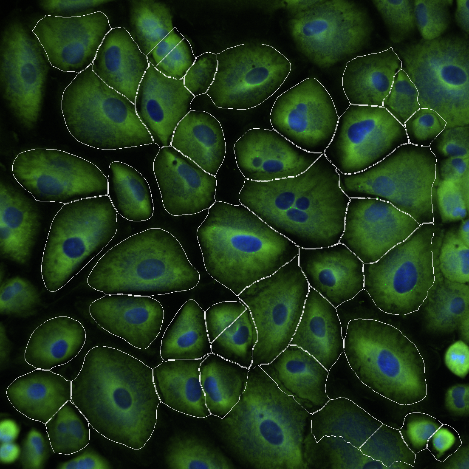

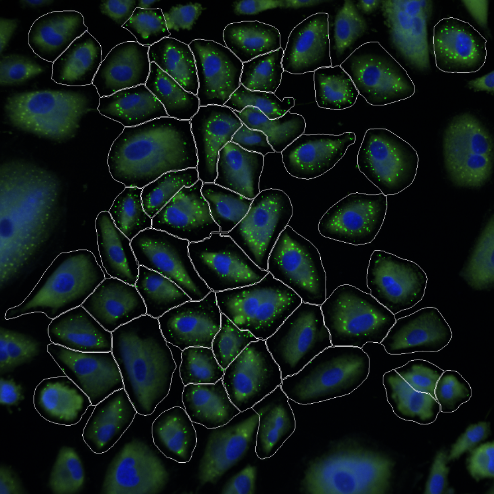


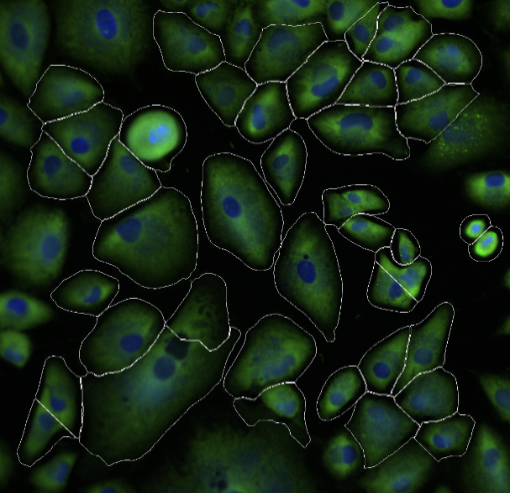

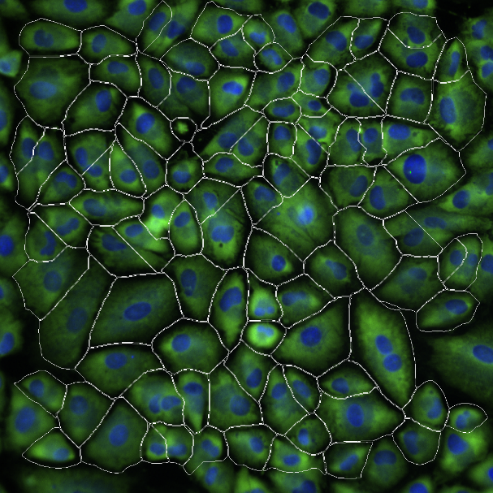


FAZ3780- LLOMe

CTR-LLOMe

FAZ3532- LLOMe

Supplement: Supplementary file 4 — Source data Fig. 1 [file 44318_2024_292_MOESM4_ESM.zip › Figure 1/1H/README.docx]

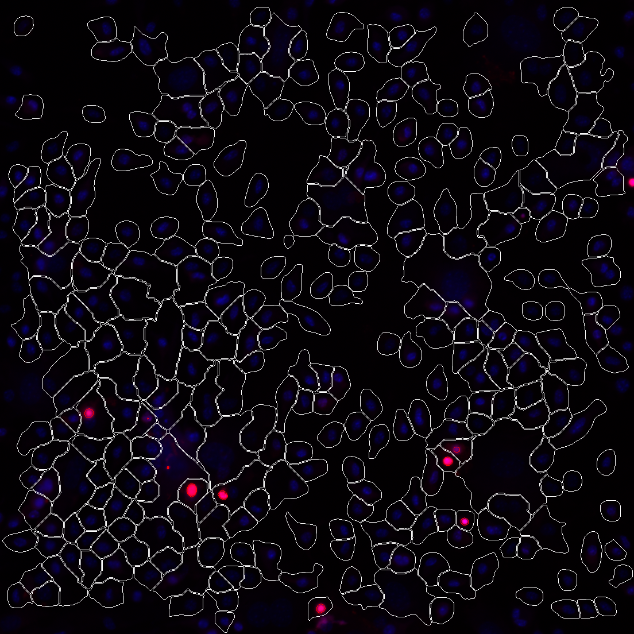

Supplement: Supplementary file 4 — Source data Fig. 1 [file 44318_2024_292_MOESM4_ESM.zip › Figure 1/1I/CTR-LLOMe.tif]

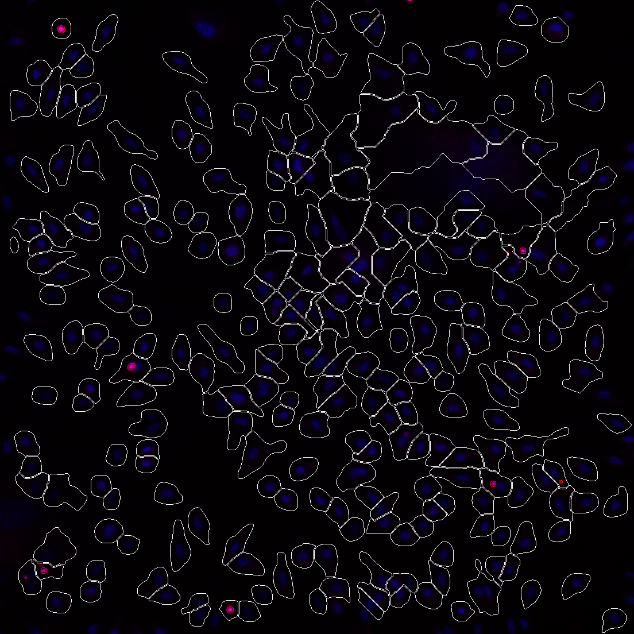

Supplement: Supplementary file 4 — Source data Fig. 1 [file 44318_2024_292_MOESM4_ESM.zip › Figure 1/1I/CTR-NT.tif]

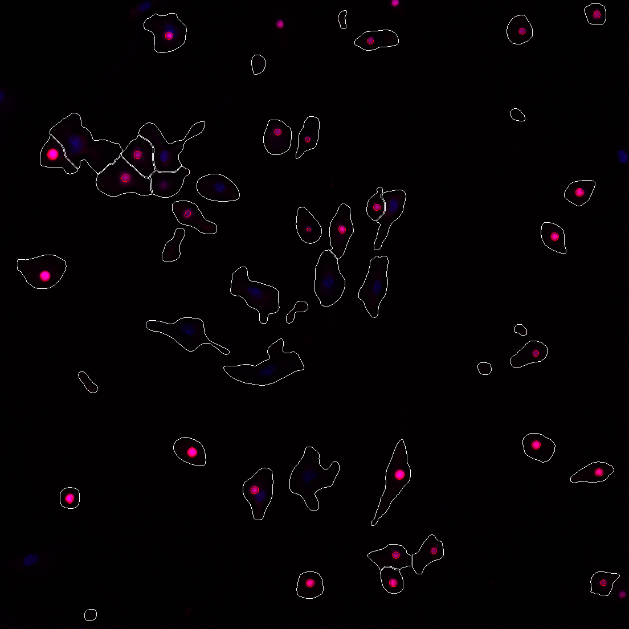

Supplement: Supplementary file 4 — Source data Fig. 1 [file 44318_2024_292_MOESM4_ESM.zip › Figure 1/1I/FAZ3532-LLOMe.tif]

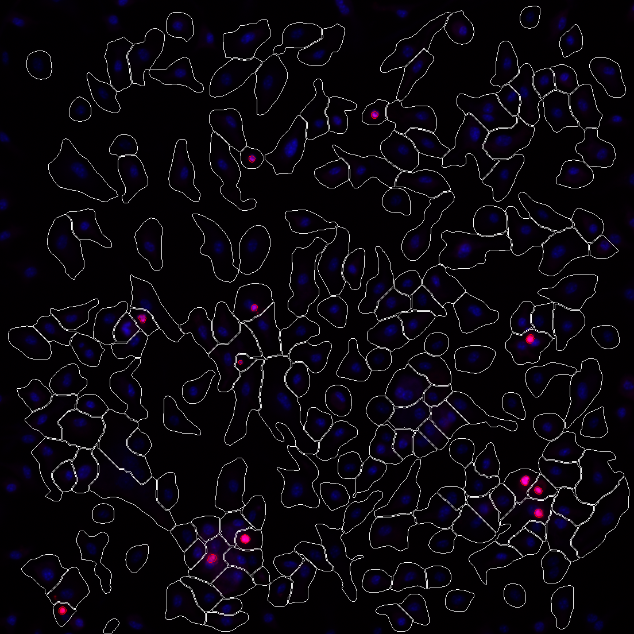

Supplement: Supplementary file 4 — Source data Fig. 1 [file 44318_2024_292_MOESM4_ESM.zip › Figure 1/1I/FAZ3532-NT.tif]

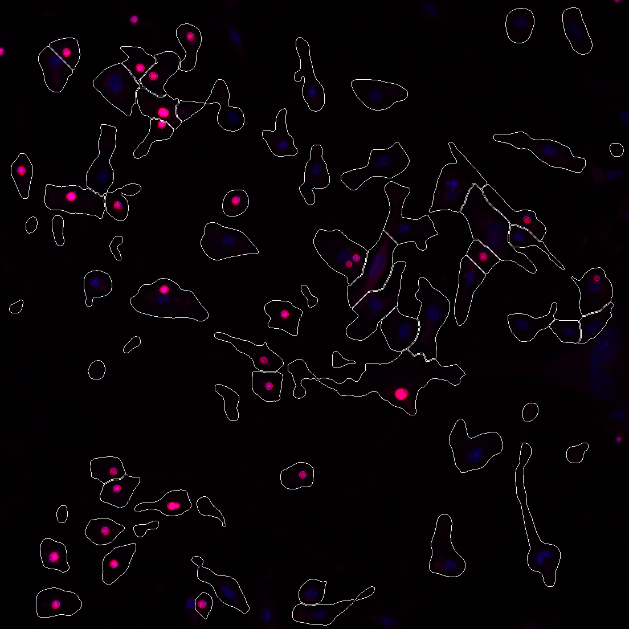

Supplement: Supplementary file 4 — Source data Fig. 1 [file 44318_2024_292_MOESM4_ESM.zip › Figure 1/1I/FAZ3780-LLOMe.tif]

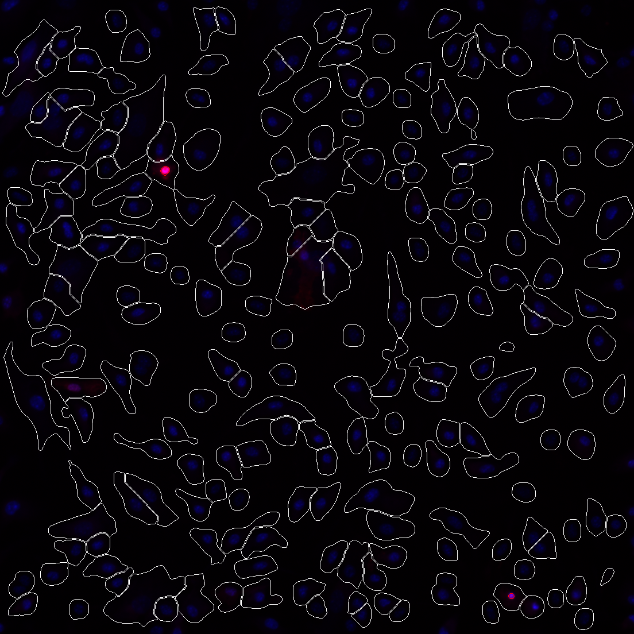

Supplement: Supplementary file 4 — Source data Fig. 1 [file 44318_2024_292_MOESM4_ESM.zip › Figure 1/1I/FAZ3780-NT.tif]

CTR-NT


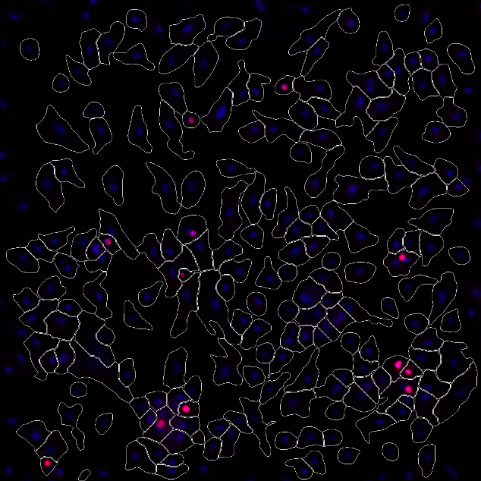

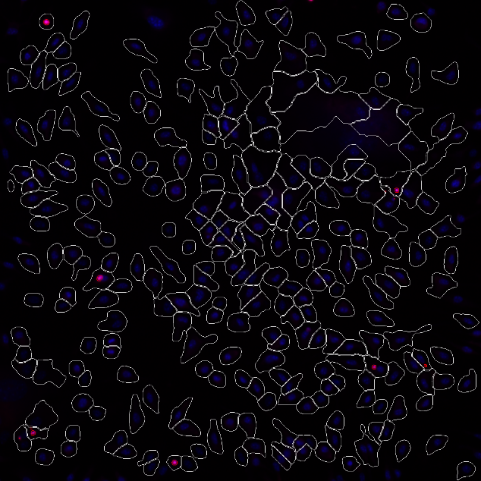


FAZ3780-NT

FAZ3532-NT


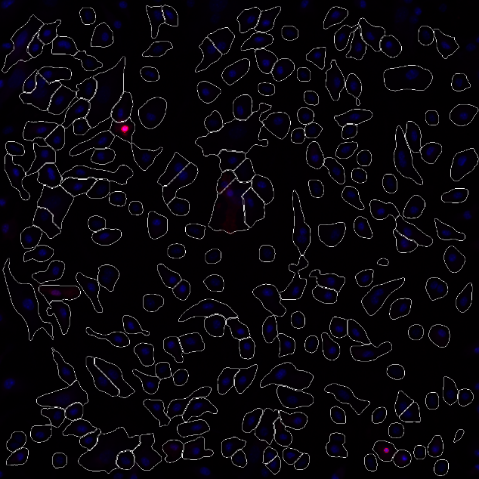


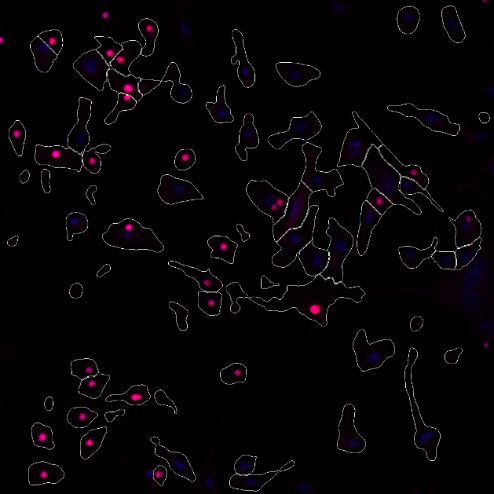

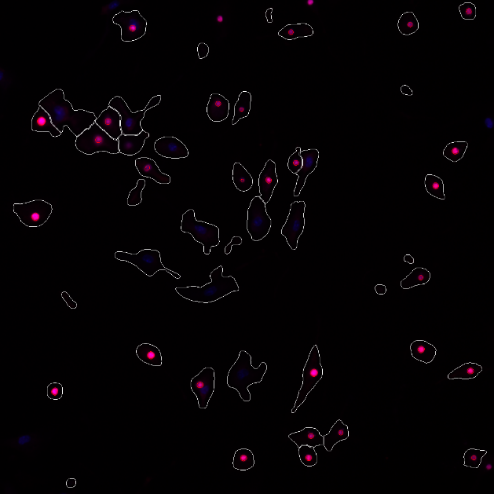

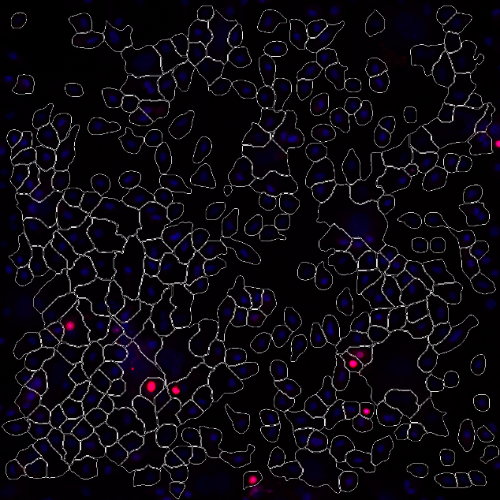


FAZ3532-LLOMe

FAZ3780-LLOMe

CTR-LLOMe

Supplement: Supplementary file 4 — Source data Fig. 1 [file 44318_2024_292_MOESM4_ESM.zip › Figure 1/1I/README.docx]

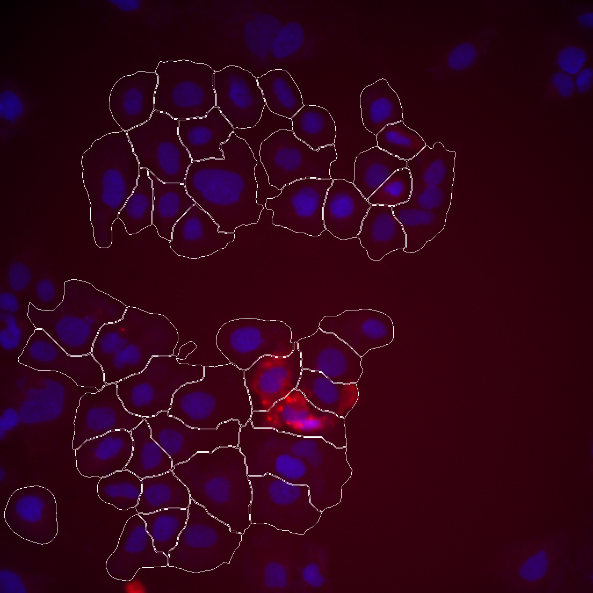

Supplement: Supplementary file 5 — Source data Fig. 2 [file 44318_2024_292_MOESM5_ESM.zip › Figure 2/2A/eIF2a-NT.tif]

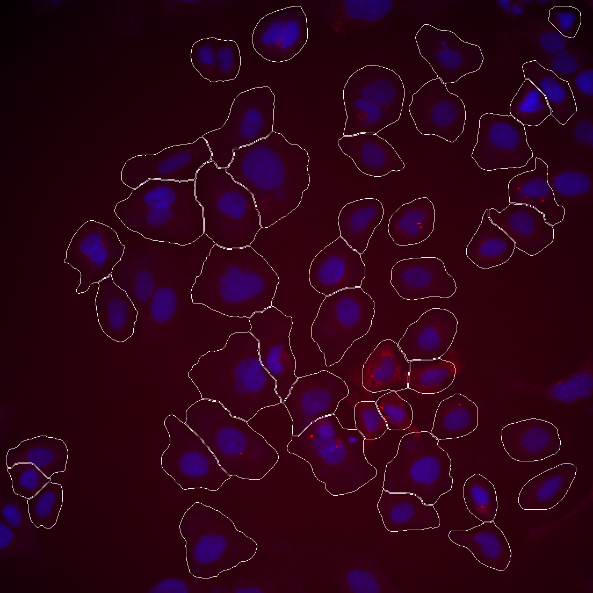

Supplement: Supplementary file 5 — Source data Fig. 2 [file 44318_2024_292_MOESM5_ESM.zip › Figure 2/2A/eIF2aKD-LLOMe.tif]

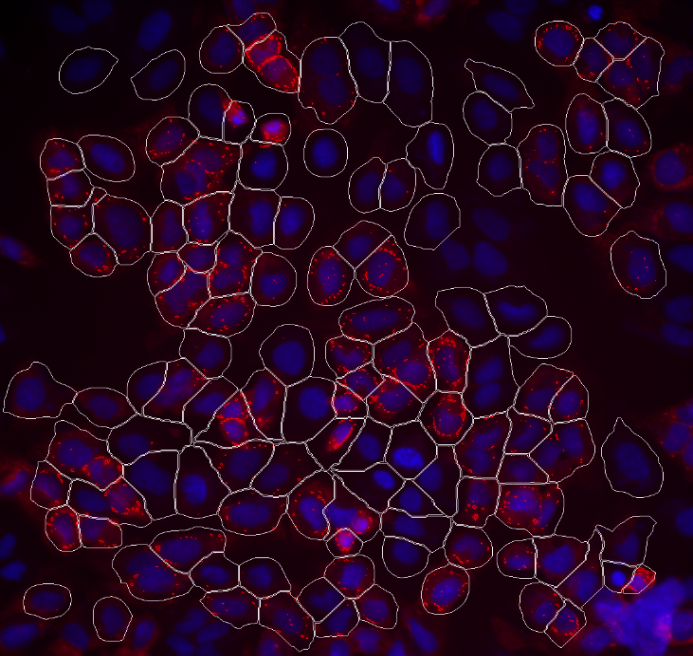

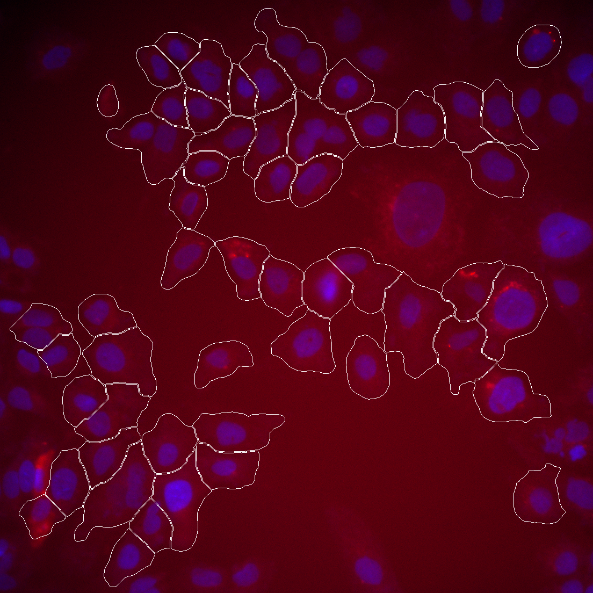

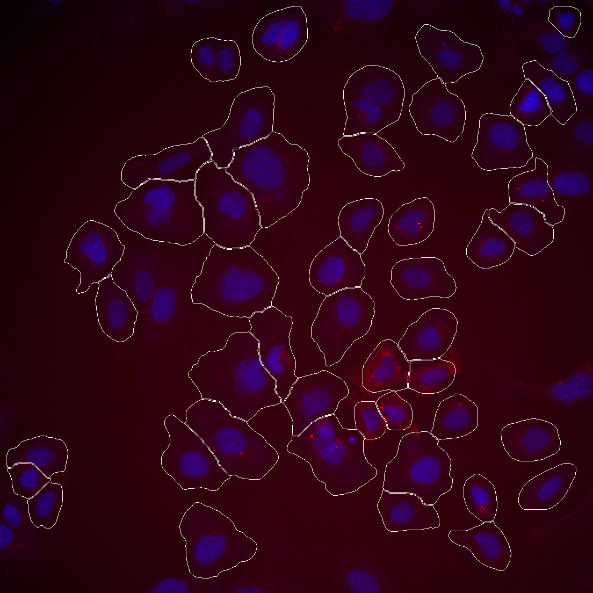

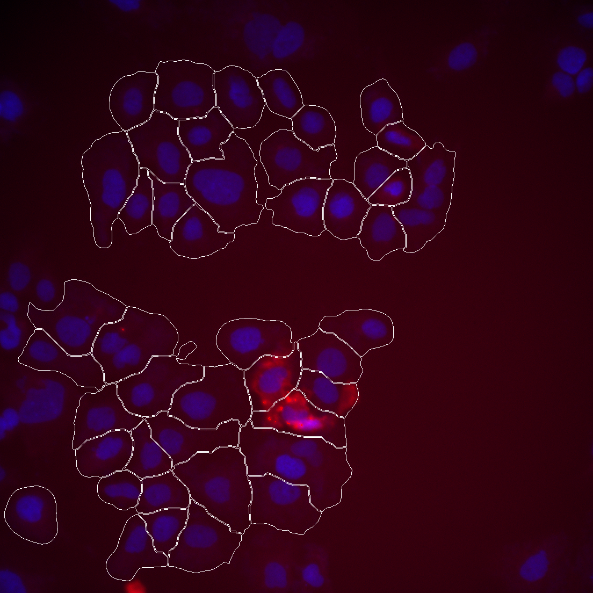


eIF2aKD -NT

SCR-NT

SCR-LLOMe

eIF2aKD -LLOMe


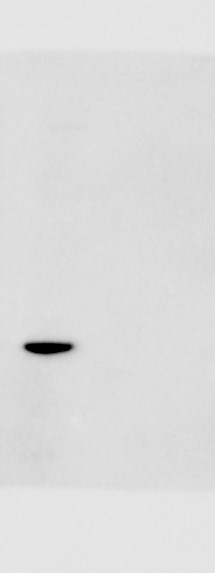

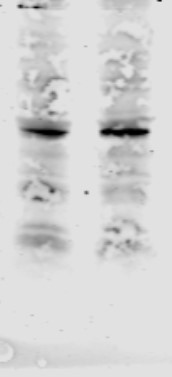


SCR

KDa

50

37

25

75

SCR

eIF2aKD

eIF2aKD

KDa

50

37

75

β-actin

eIF2α

Supplement: Supplementary file 5 — Source data Fig. 2 [file 44318_2024_292_MOESM5_ESM.zip › Figure 2/2A/README.docx]

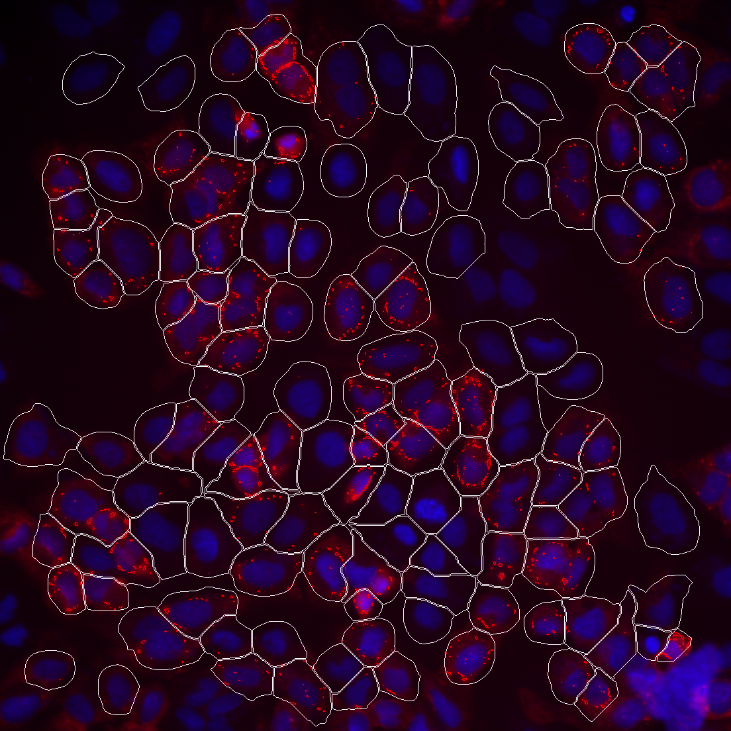

Supplement: Supplementary file 5 — Source data Fig. 2 [file 44318_2024_292_MOESM5_ESM.zip › Figure 2/2A/SCR-LLOMe.tif]

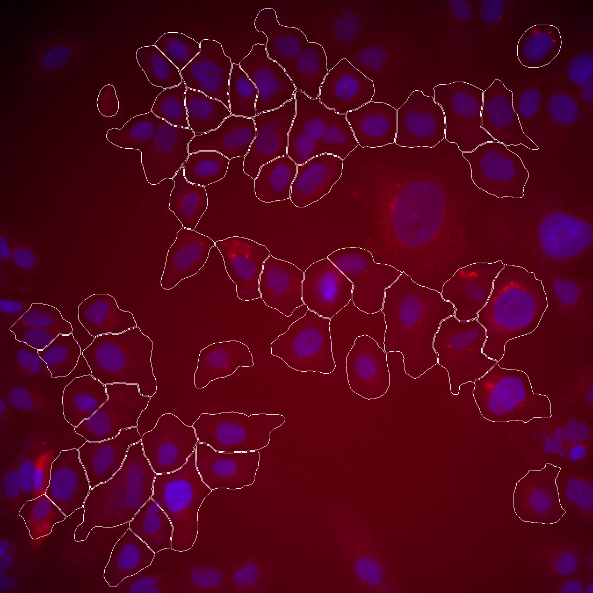

Supplement: Supplementary file 5 — Source data Fig. 2 [file 44318_2024_292_MOESM5_ESM.zip › Figure 2/2A/SCR-NT.tif]

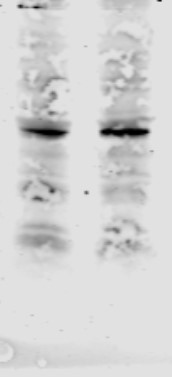

Supplement: Supplementary file 5 — Source data Fig. 2 [file 44318_2024_292_MOESM5_ESM.zip › Figure 2/2A/WB-actin.jpg]

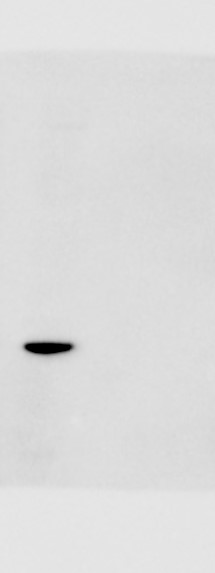

Supplement: Supplementary file 5 — Source data Fig. 2 [file 44318_2024_292_MOESM5_ESM.zip › Figure 2/2A/WB-eIF2a.jpg]

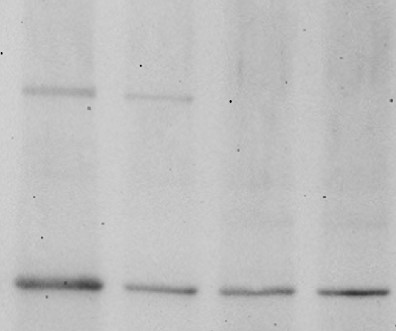

Supplement: Supplementary file 5 — Source data Fig. 2 [file 44318_2024_292_MOESM5_ESM.zip › Figure 2/2B/4EBP.jpg]

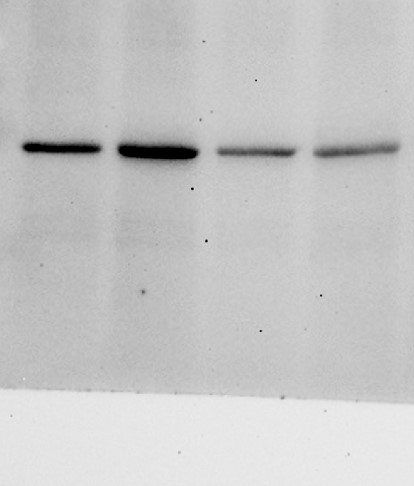

Supplement: Supplementary file 5 — Source data Fig. 2 [file 44318_2024_292_MOESM5_ESM.zip › Figure 2/2B/b-actin.jpg]

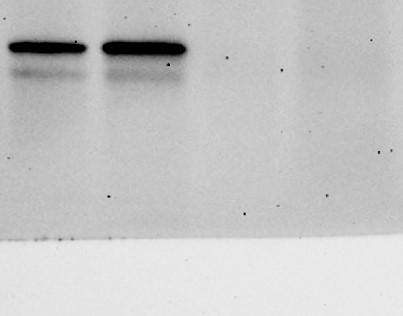

Supplement: Supplementary file 5 — Source data Fig. 2 [file 44318_2024_292_MOESM5_ESM.zip › Figure 2/2B/eIF2a.jpg]

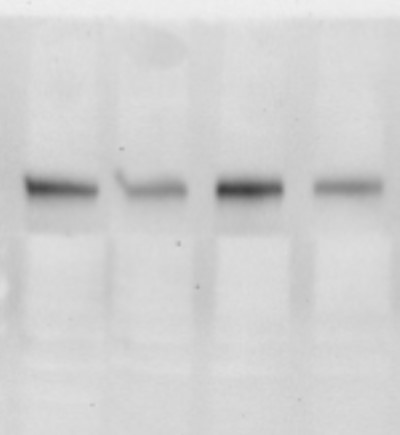

Supplement: Supplementary file 5 — Source data Fig. 2 [file 44318_2024_292_MOESM5_ESM.zip › Figure 2/2B/S142-TFEB.jpg]

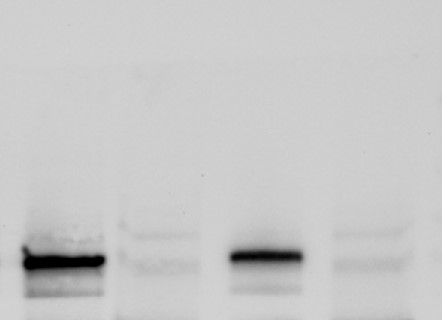

Supplement: Supplementary file 5 — Source data Fig. 2 [file 44318_2024_292_MOESM5_ESM.zip › Figure 2/2B/S65-4EBP.jpg]

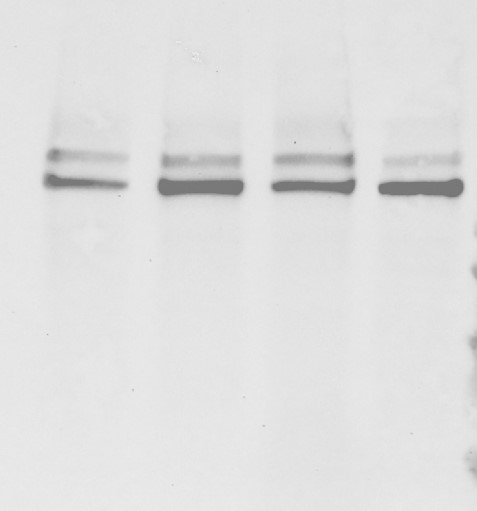

Supplement: Supplementary file 5 — Source data Fig. 2 [file 44318_2024_292_MOESM5_ESM.zip › Figure 2/2B/S6K.jpg]

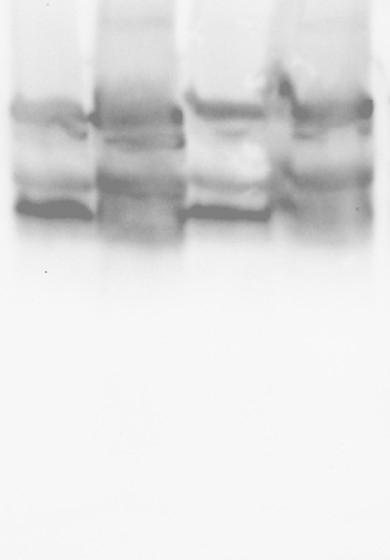

Supplement: Supplementary file 5 — Source data Fig. 2 [file 44318_2024_292_MOESM5_ESM.zip › Figure 2/2B/S757-ULK1.jpg]

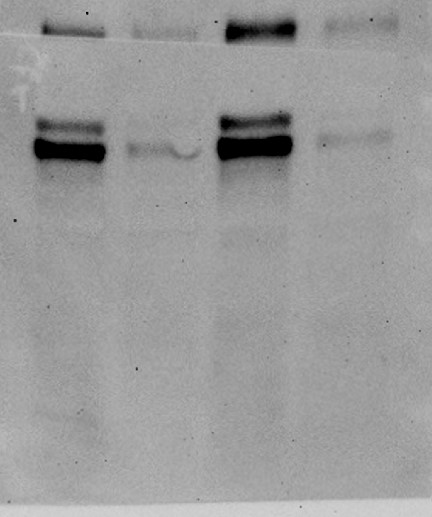

Supplement: Supplementary file 5 — Source data Fig. 2 [file 44318_2024_292_MOESM5_ESM.zip › Figure 2/2B/T389-S6K.jpg]

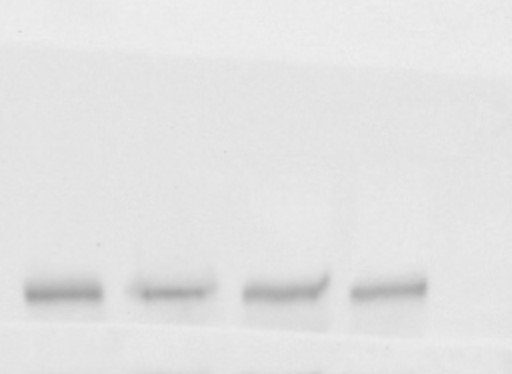

Supplement: Supplementary file 5 — Source data Fig. 2 [file 44318_2024_292_MOESM5_ESM.zip › Figure 2/2B/TFEB.jpg]

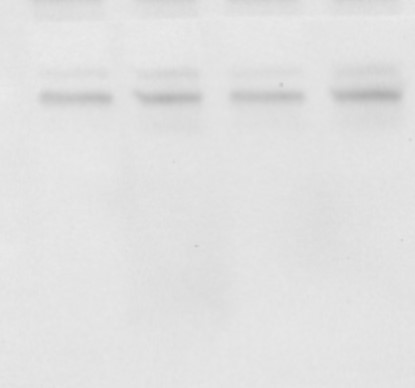

Supplement: Supplementary file 5 — Source data Fig. 2 [file 44318_2024_292_MOESM5_ESM.zip › Figure 2/2B/ULK1.jpg]

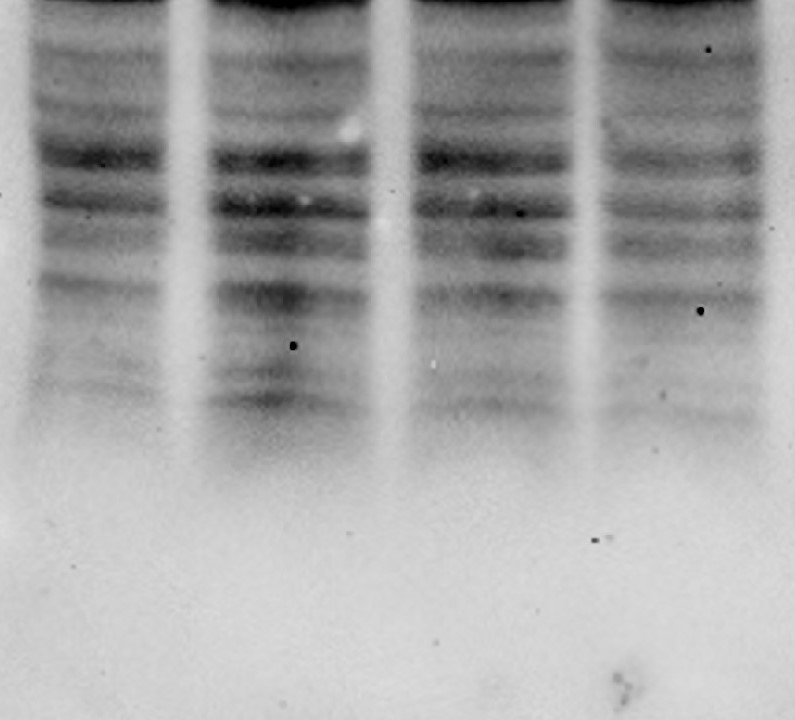

Supplement: Supplementary file 5 — Source data Fig. 2 [file 44318_2024_292_MOESM5_ESM.zip › Figure 2/2C/4EBP.jpg]

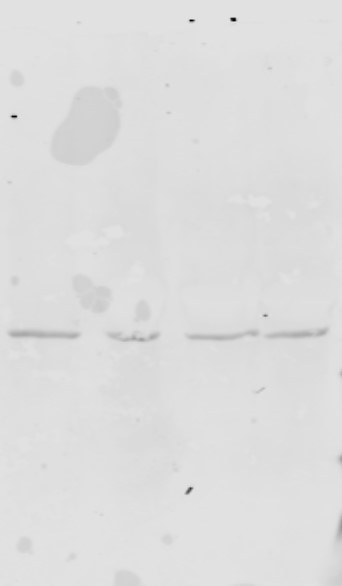

Supplement: Supplementary file 5 — Source data Fig. 2 [file 44318_2024_292_MOESM5_ESM.zip › Figure 2/2C/eIF2a.jpg]

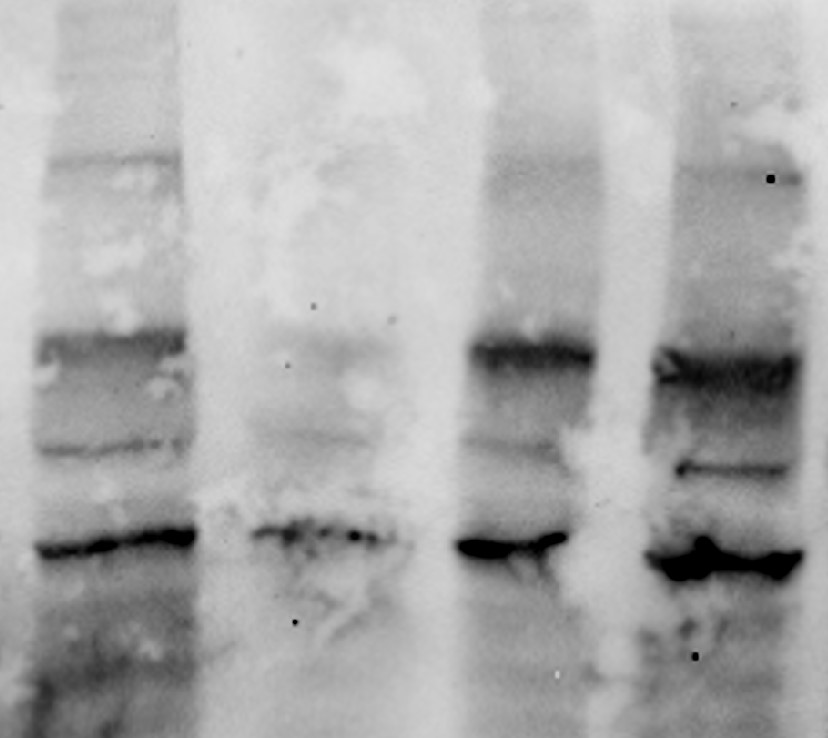

Supplement: Supplementary file 5 — Source data Fig. 2 [file 44318_2024_292_MOESM5_ESM.zip › Figure 2/2C/P-4EBP.jpg]

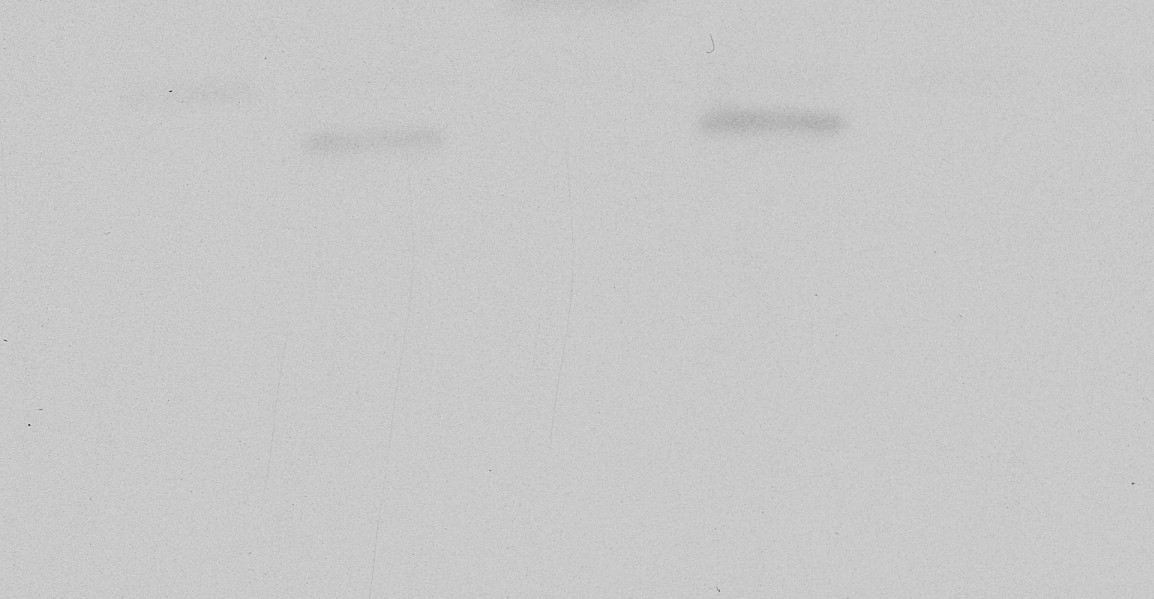

Supplement: Supplementary file 5 — Source data Fig. 2 [file 44318_2024_292_MOESM5_ESM.zip › Figure 2/2C/P-eIF2a.jpg]

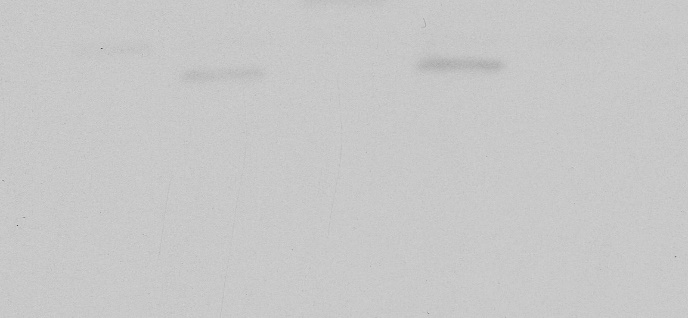

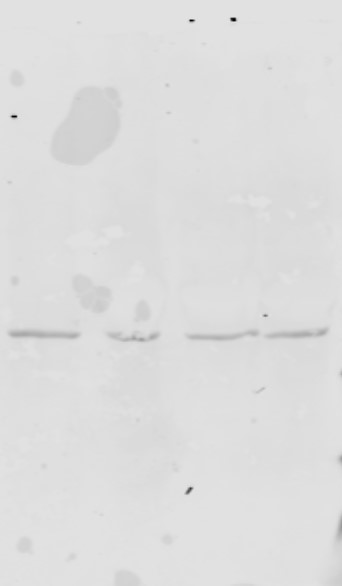


p-eIF2a

KDa

37

25

KDa

37

25

eIF2a

RagBQ99L

RagBWT

RagBQ99L

RagBWT

LLOMe: - + - +

LLOMe: - + - +

KDa

37

25


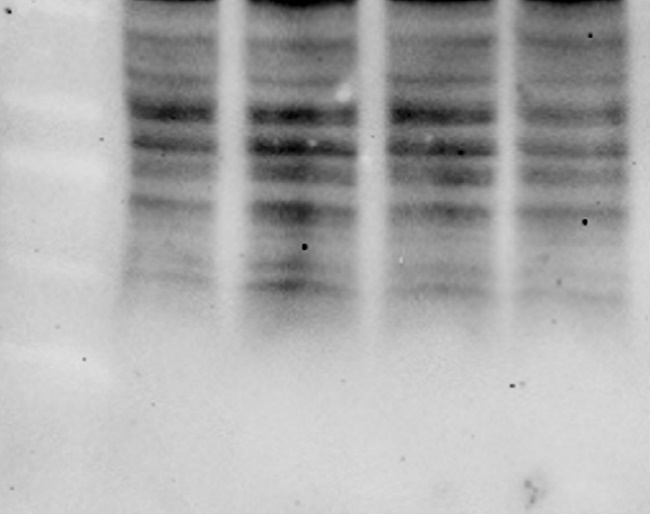

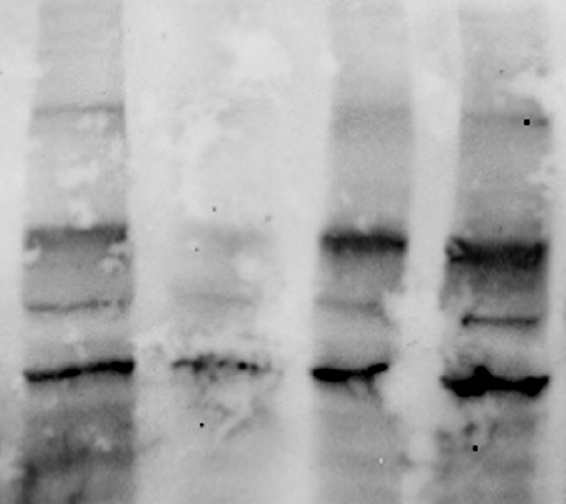


KDa

37

25

4EBP

P-4EBP

SCR

Supplement: Supplementary file 5 — Source data Fig. 2 [file 44318_2024_292_MOESM5_ESM.zip › Figure 2/2C/README.docx]

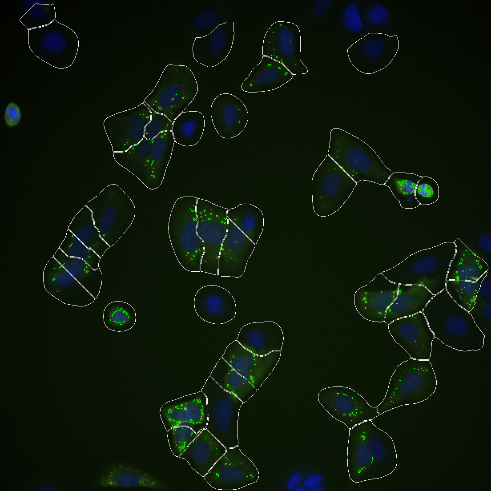

Supplement: Supplementary file 5 — Source data Fig. 2 [file 44318_2024_292_MOESM5_ESM.zip › Figure 2/2D/RAGBQ99-LLOMe.tif]

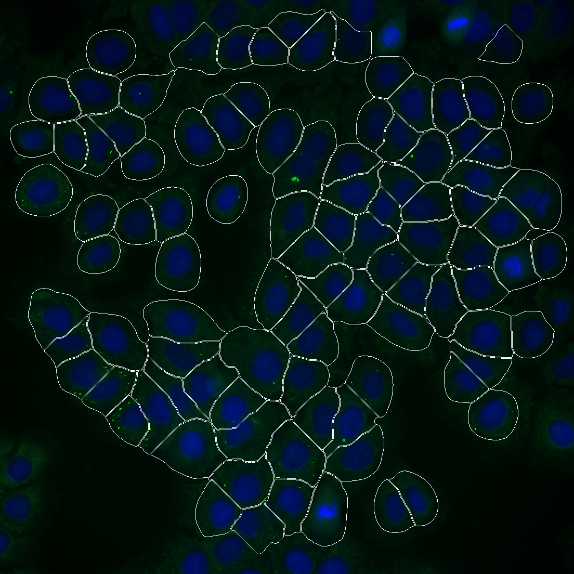

Supplement: Supplementary file 5 — Source data Fig. 2 [file 44318_2024_292_MOESM5_ESM.zip › Figure 2/2D/RAGBQ99-NT.tif]

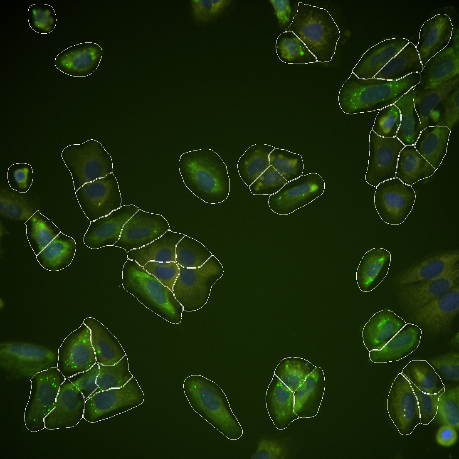

Supplement: Supplementary file 5 — Source data Fig. 2 [file 44318_2024_292_MOESM5_ESM.zip › Figure 2/2D/RAGBWT-LLOMe.tif]

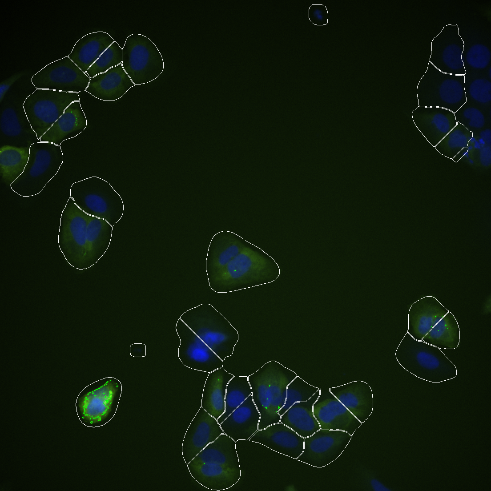

Supplement: Supplementary file 5 — Source data Fig. 2 [file 44318_2024_292_MOESM5_ESM.zip › Figure 2/2D/RAGBWT-NT.tif]

RagBQ99L -NT

RagBQ99L -LLOMe

RagBWT-NT


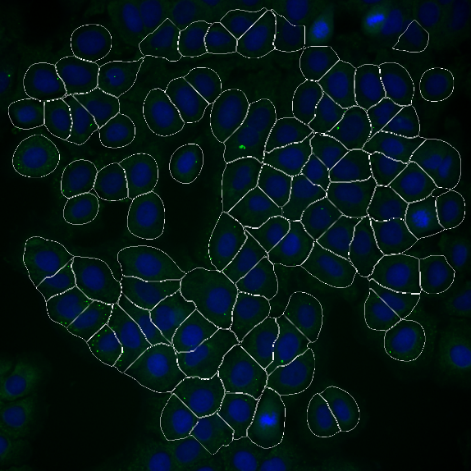

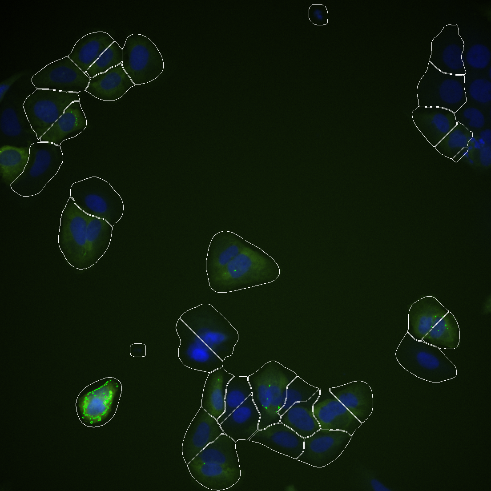


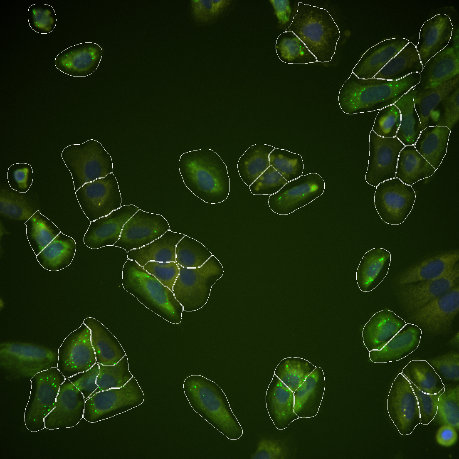

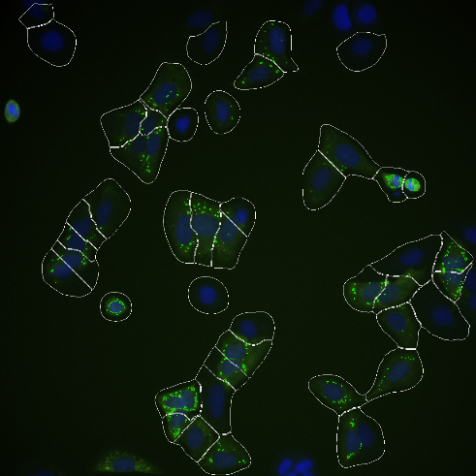


RagBWT-LLOMe

Supplement: Supplementary file 5 — Source data Fig. 2 [file 44318_2024_292_MOESM5_ESM.zip › Figure 2/2D/README.docx]

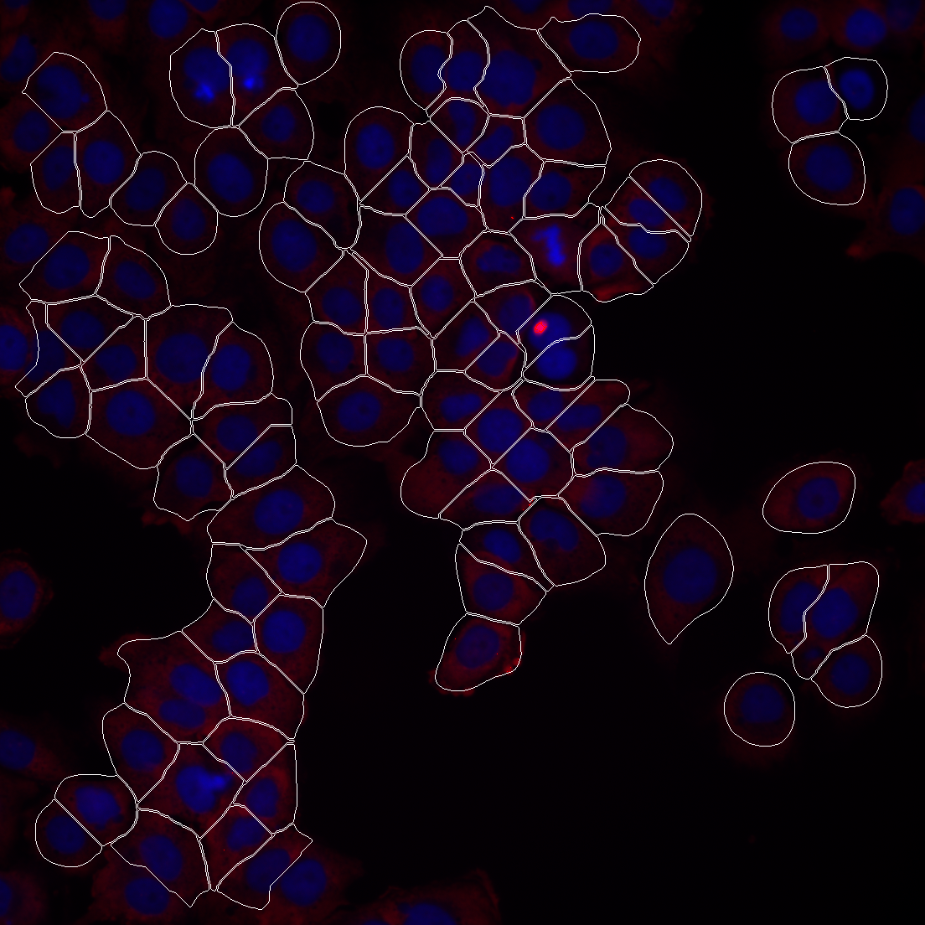

Supplement: Supplementary file 5 — Source data Fig. 2 [file 44318_2024_292_MOESM5_ESM.zip › Figure 2/2E/FLAG-eIF2AS51A.tif]

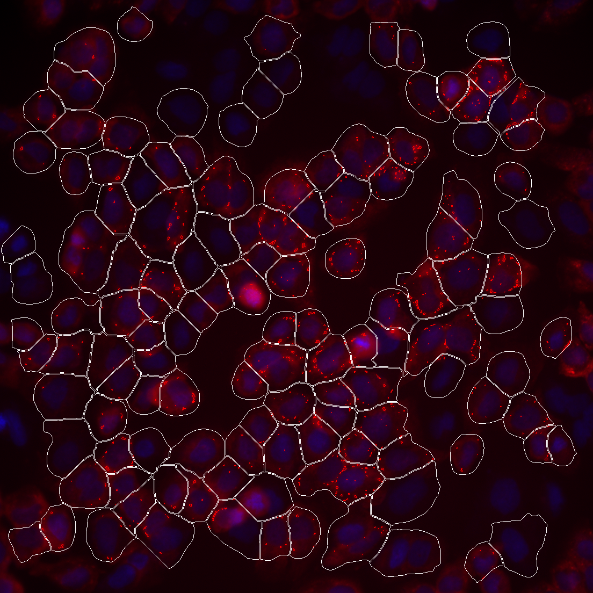

Supplement: Supplementary file 5 — Source data Fig. 2 [file 44318_2024_292_MOESM5_ESM.zip › Figure 2/2E/FLAG-eIF2AWT.tif]

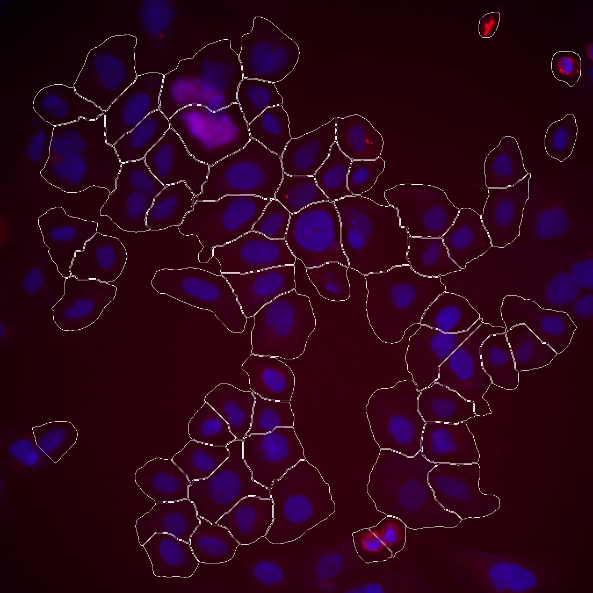

Supplement: Supplementary file 5 — Source data Fig. 2 [file 44318_2024_292_MOESM5_ESM.zip › Figure 2/2E/FLAG.tif]

FLAG-eIF2αWT

FLAG


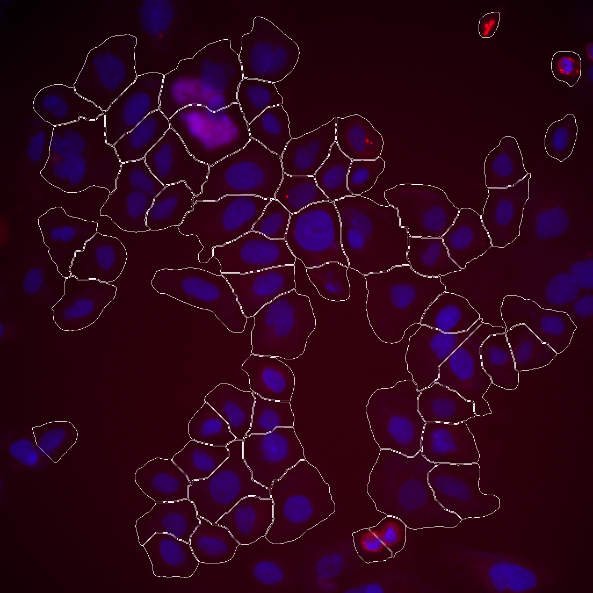


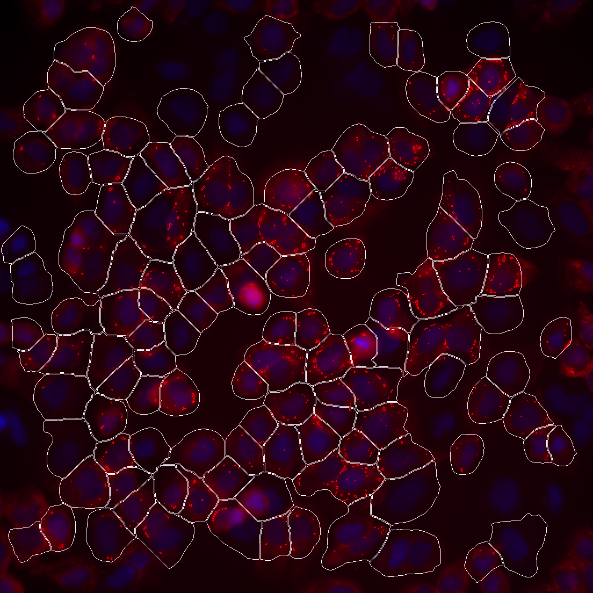


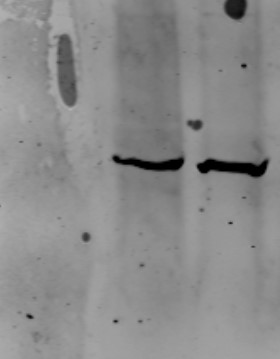


FLAG WT S51A

KDa

50

37

25

75

FLAG-eIF2αS51A


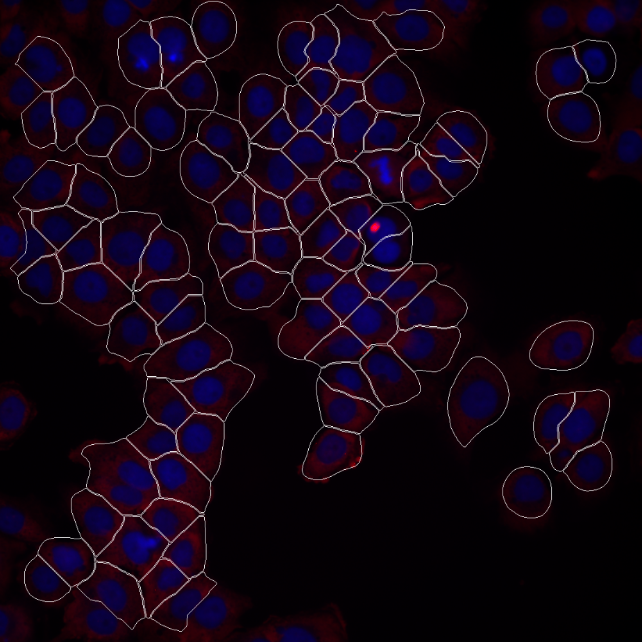


FLAG


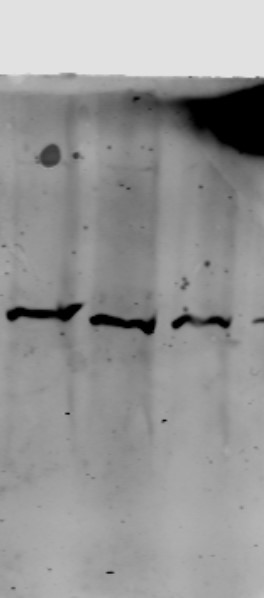


KDa

50

37

25

75

β-actin

Supplement: Supplementary file 5 — Source data Fig. 2 [file 44318_2024_292_MOESM5_ESM.zip › Figure 2/2E/README.docx]

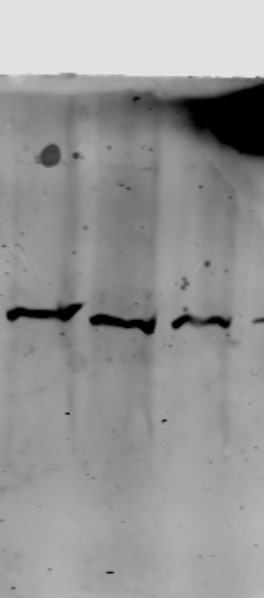

Supplement: Supplementary file 5 — Source data Fig. 2 [file 44318_2024_292_MOESM5_ESM.zip › Figure 2/2E/WB-b-actin.jpg]

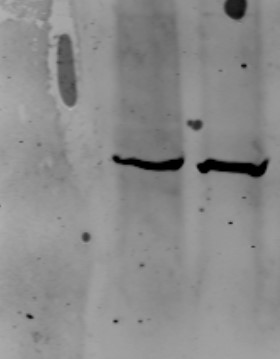

Supplement: Supplementary file 5 — Source data Fig. 2 [file 44318_2024_292_MOESM5_ESM.zip › Figure 2/2E/WB-FLAG.jpg]

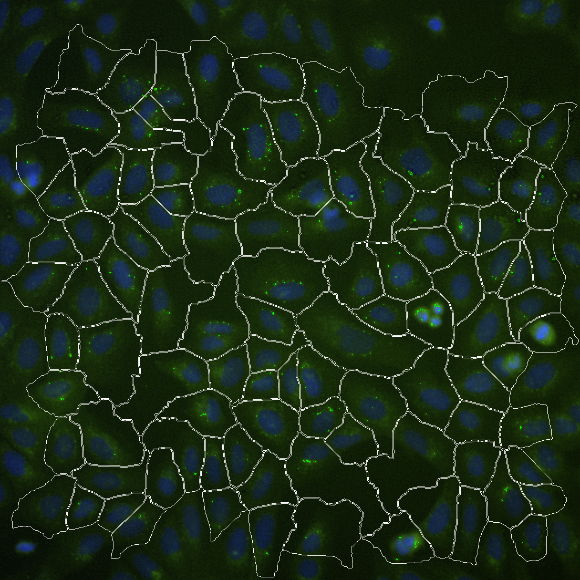

Supplement: Supplementary file 6 — Source data Fig. 3 [file 44318_2024_292_MOESM6_ESM.zip › Figure 3/3B/KO-LLOMe.tif]

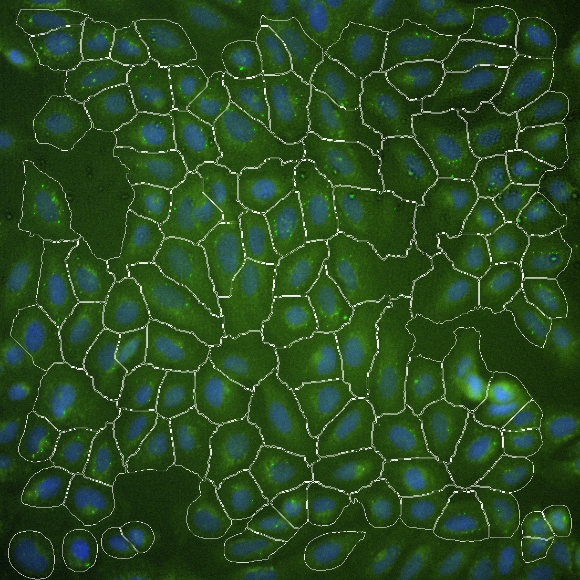

Supplement: Supplementary file 6 — Source data Fig. 3 [file 44318_2024_292_MOESM6_ESM.zip › Figure 3/3B/KO-NT.tif]

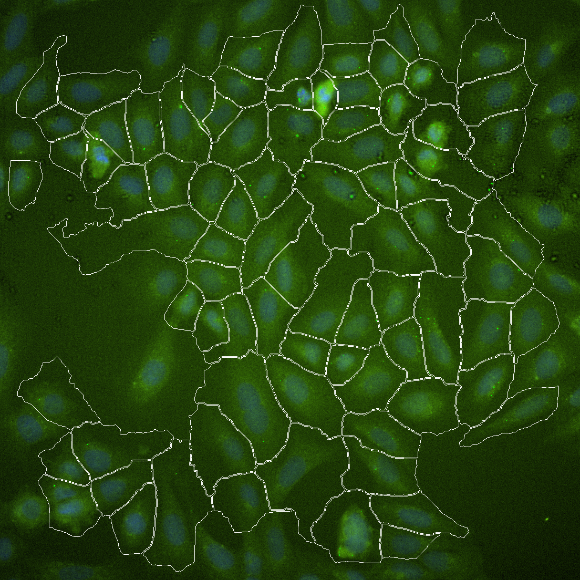


KO-NT

WT-NT


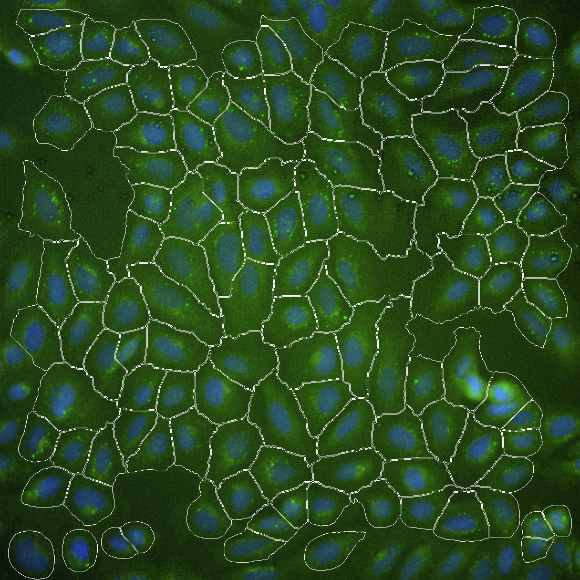


KO-LLOMe

WT-LLOMe


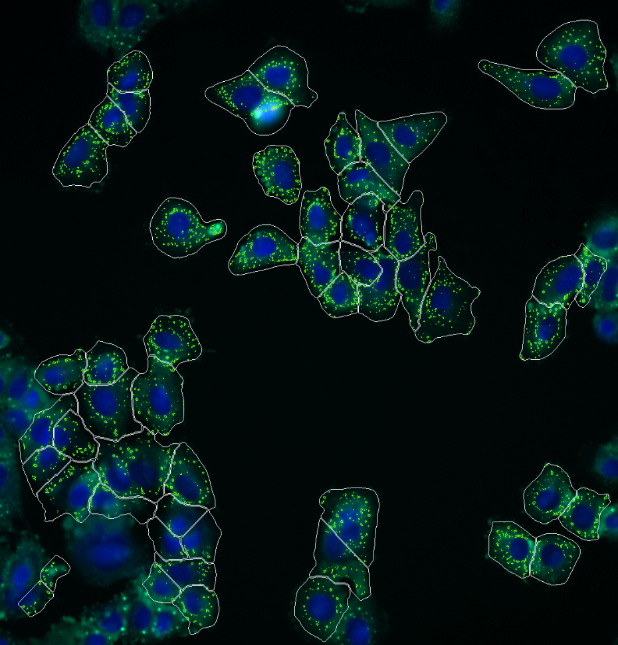

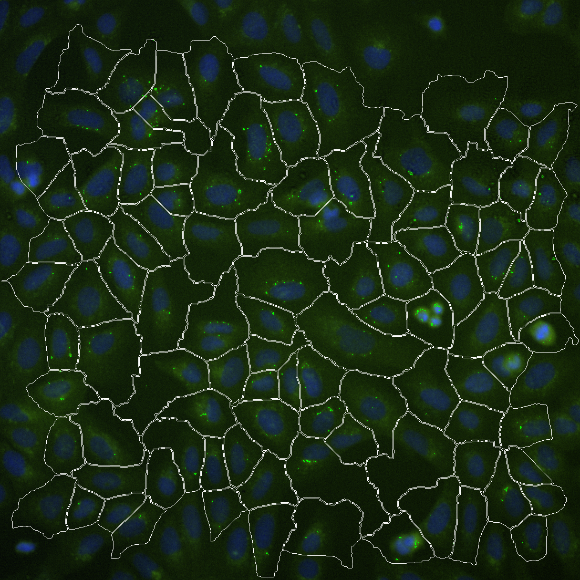


FLAG-EIF2AS51A


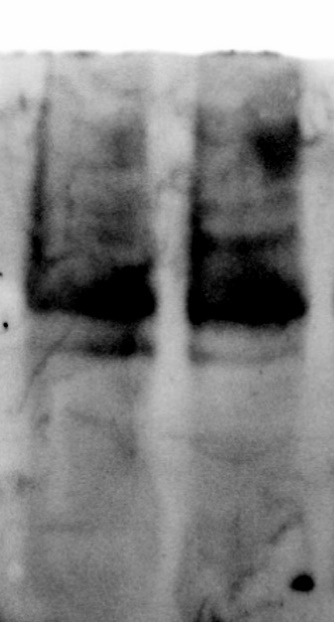


WT KO

WT KO

KDa

50

37

75

KDa

50

37

25

75


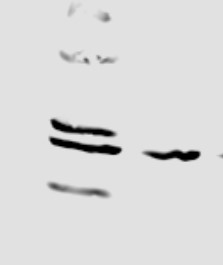


130

β-actin

PKR

Supplement: Supplementary file 6 — Source data Fig. 3 [file 44318_2024_292_MOESM6_ESM.zip › Figure 3/3B/README.docx]

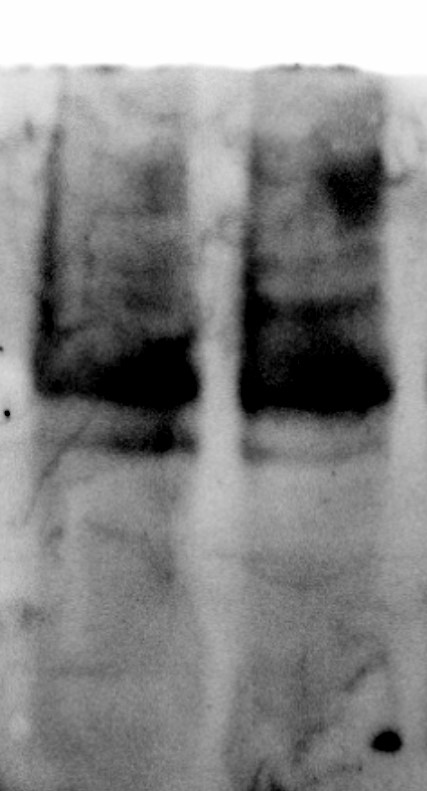

Supplement: Supplementary file 6 — Source data Fig. 3 [file 44318_2024_292_MOESM6_ESM.zip › Figure 3/3B/WB-actin.jpg]

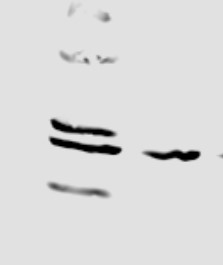

Supplement: Supplementary file 6 — Source data Fig. 3 [file 44318_2024_292_MOESM6_ESM.zip › Figure 3/3B/WB-PKR.jpg]

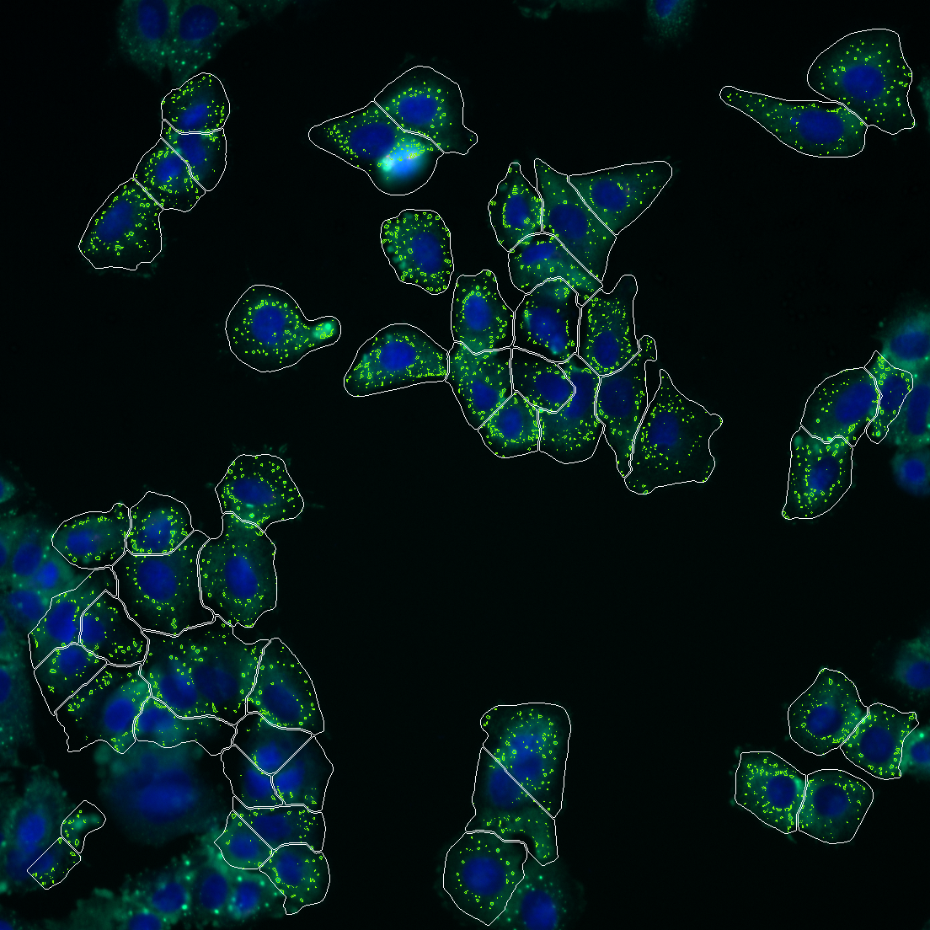

Supplement: Supplementary file 6 — Source data Fig. 3 [file 44318_2024_292_MOESM6_ESM.zip › Figure 3/3B/WT-LLOMe.tif]

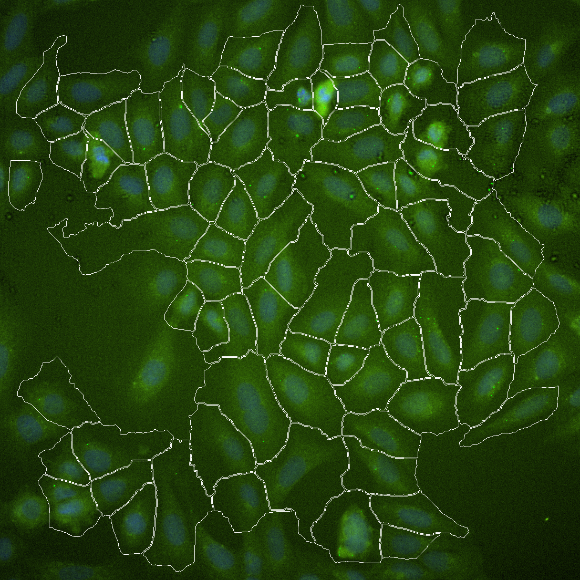

Supplement: Supplementary file 6 — Source data Fig. 3 [file 44318_2024_292_MOESM6_ESM.zip › Figure 3/3B/WT-NT.tif]

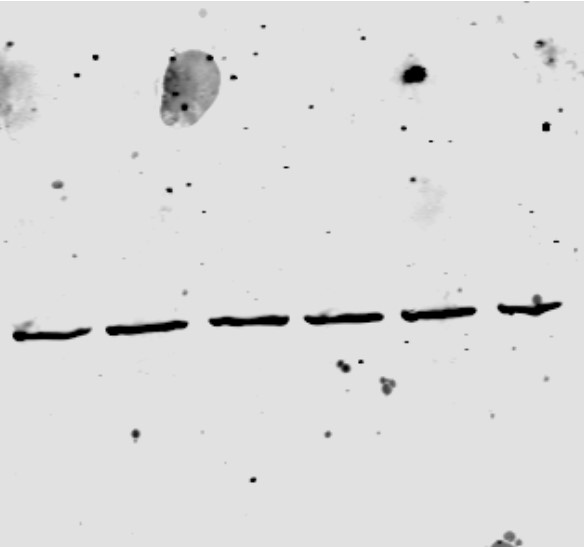

Supplement: Supplementary file 6 — Source data Fig. 3 [file 44318_2024_292_MOESM6_ESM.zip › Figure 3/3C/b-actin.jpg]

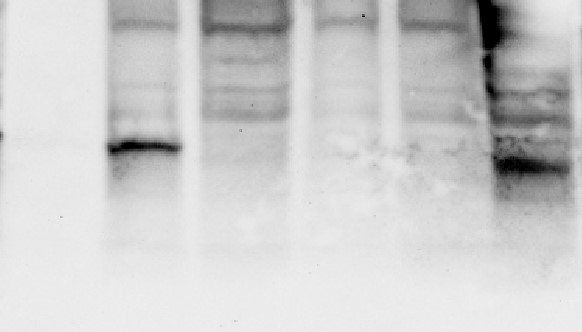

Supplement: Supplementary file 6 — Source data Fig. 3 [file 44318_2024_292_MOESM6_ESM.zip › Figure 3/3C/p-eIF2a.jpg]

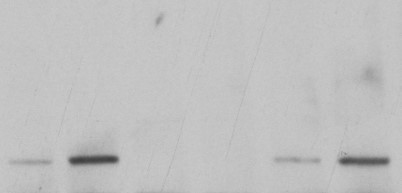

Supplement: Supplementary file 6 — Source data Fig. 3 [file 44318_2024_292_MOESM6_ESM.zip › Figure 3/3C/p-PKR.jpg]

WT

KO

LLOME: - + - + - +

KDa

50

37

25

75


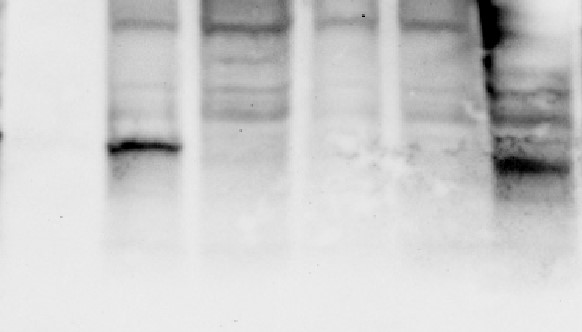


P-eIF2a

KDa

110

72

55


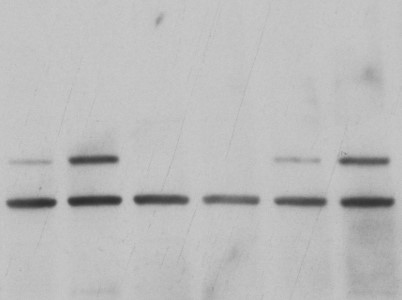


SCR

P-PKR


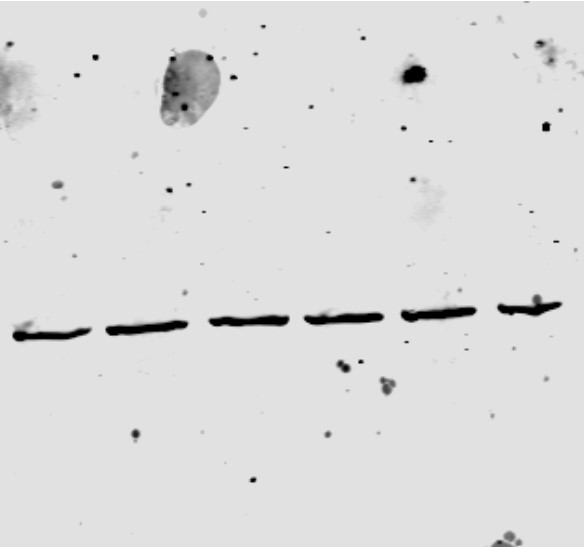


KDa

50

25

b-actin

Supplement: Supplementary file 6 — Source data Fig. 3 [file 44318_2024_292_MOESM6_ESM.zip › Figure 3/3C/README.docx]

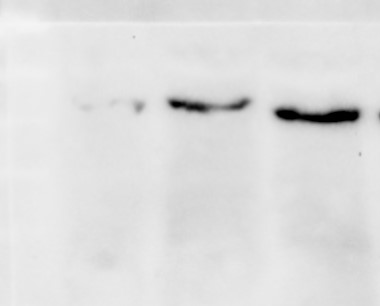

Supplement: Supplementary file 6 — Source data Fig. 3 [file 44318_2024_292_MOESM6_ESM.zip › Figure 3/3D/INPUT-FLAG.jpg]

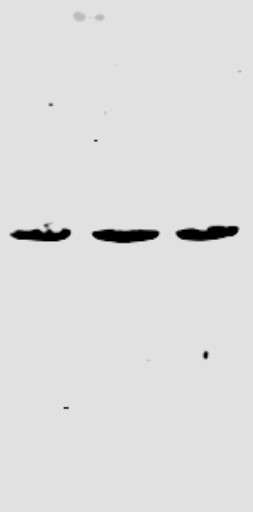

Supplement: Supplementary file 6 — Source data Fig. 3 [file 44318_2024_292_MOESM6_ESM.zip › Figure 3/3D/INPUT-PACT.jpg]

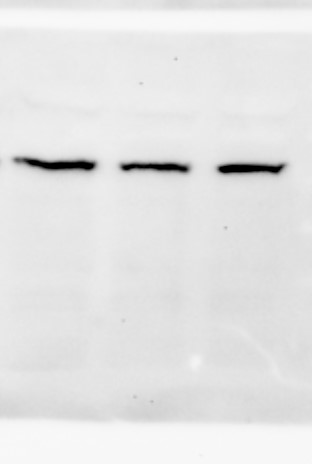

Supplement: Supplementary file 6 — Source data Fig. 3 [file 44318_2024_292_MOESM6_ESM.zip › Figure 3/3D/INPUT-PKR.jpg]

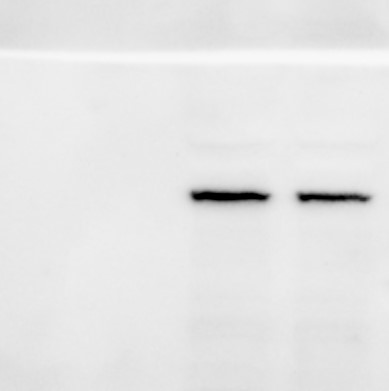

Supplement: Supplementary file 6 — Source data Fig. 3 [file 44318_2024_292_MOESM6_ESM.zip › Figure 3/3D/IP-FLAG.jpg]

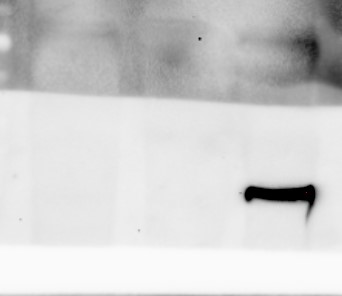

Supplement: Supplementary file 6 — Source data Fig. 3 [file 44318_2024_292_MOESM6_ESM.zip › Figure 3/3D/IP-PACT.jpg]

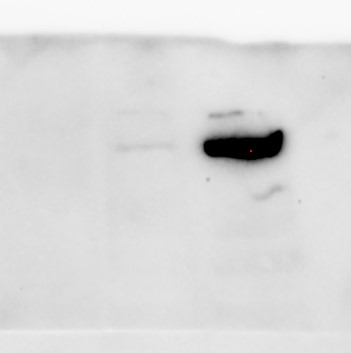

Supplement: Supplementary file 6 — Source data Fig. 3 [file 44318_2024_292_MOESM6_ESM.zip › Figure 3/3D/IP-PKR.jpg]

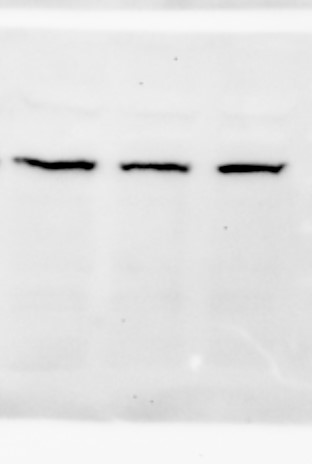

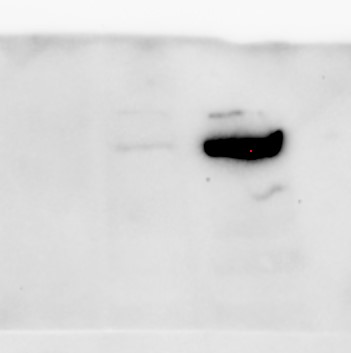


LLOMe: - - +

INPUT:

IP:

KDa

50

37

25

75

LLOMe: - - +

KDa

50

37

25

75

PKR

PKR


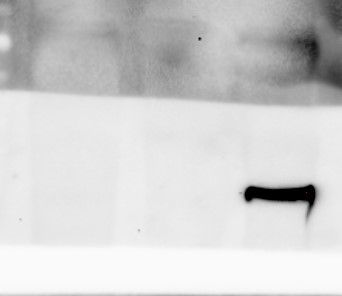


SCR


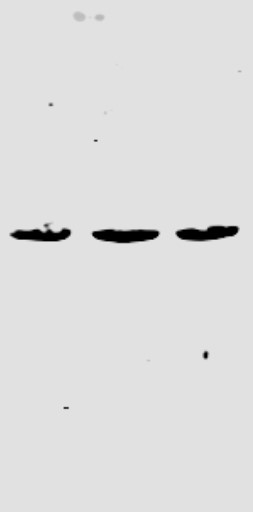


KDa

50

37

25

KDa

50

37

25

PACT

PACT


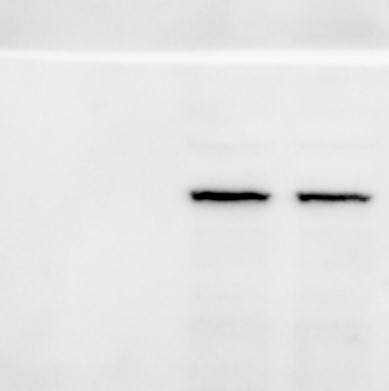


KDa

50

25


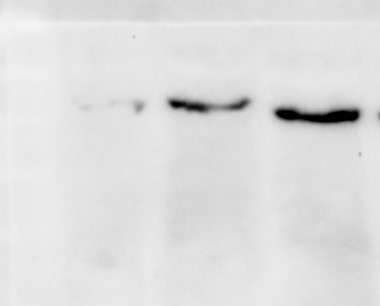


KDa

50

25

FLAG

FLAG

Supplement: Supplementary file 6 — Source data Fig. 3 [file 44318_2024_292_MOESM6_ESM.zip › Figure 3/3D/README.docx]

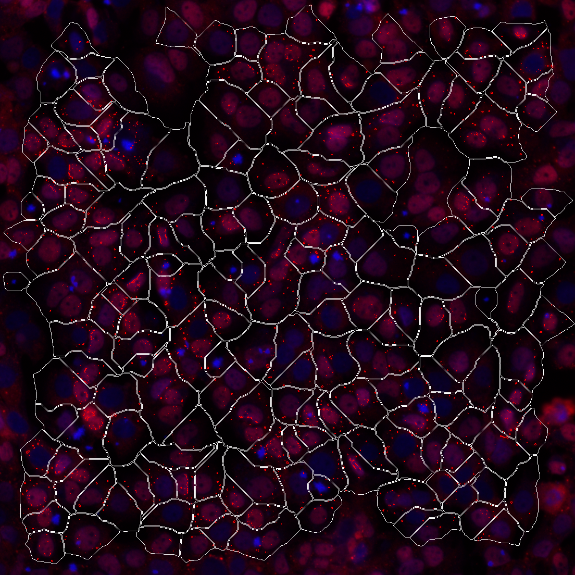

Supplement: Supplementary file 6 — Source data Fig. 3 [file 44318_2024_292_MOESM6_ESM.zip › Figure 3/3E/KO-LLOMe.tif]

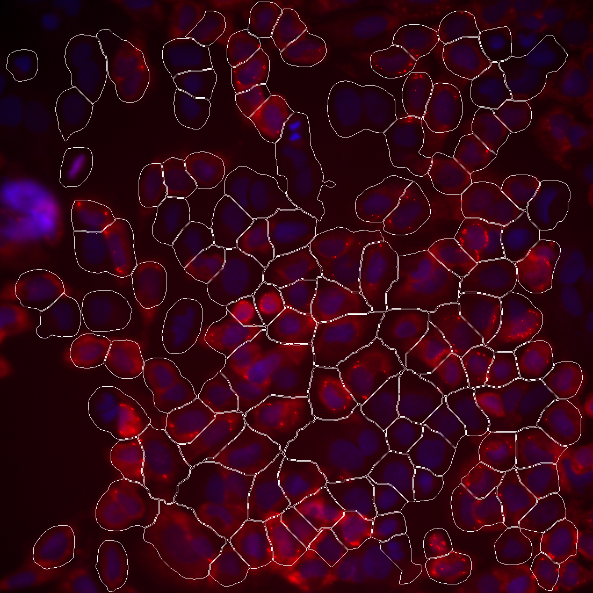

Supplement: Supplementary file 6 — Source data Fig. 3 [file 44318_2024_292_MOESM6_ESM.zip › Figure 3/3E/KO-NT.tif]

SCR-NT


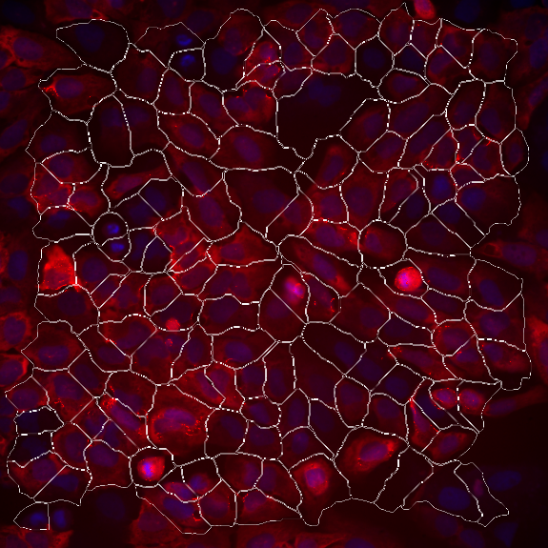

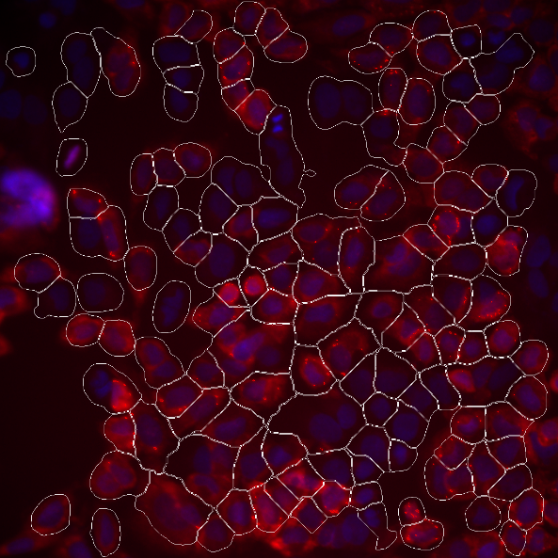


KO-NT

KO-LLOMe

SCR-LLOMe


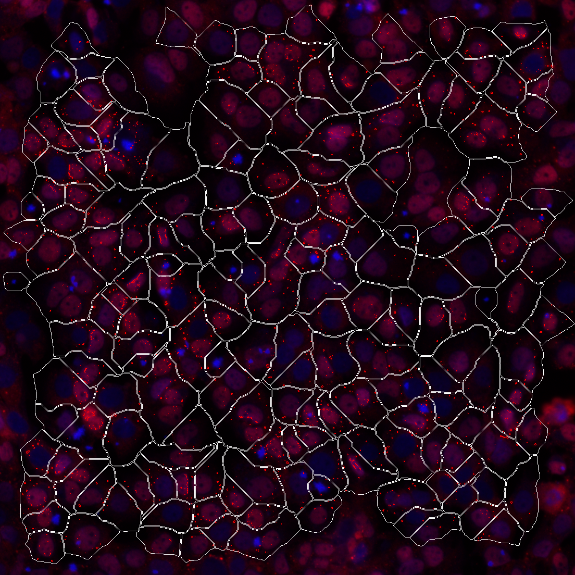

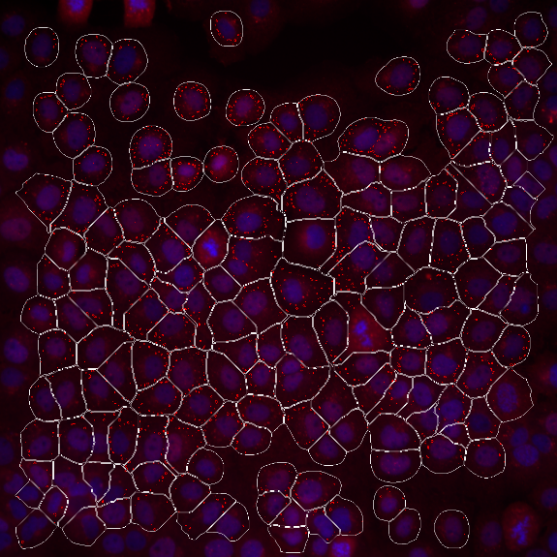


LLOMe: - + - +

SCR SCR KD KD


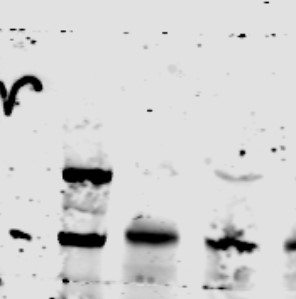


KDa

50

37

25

75

P-eIF2a


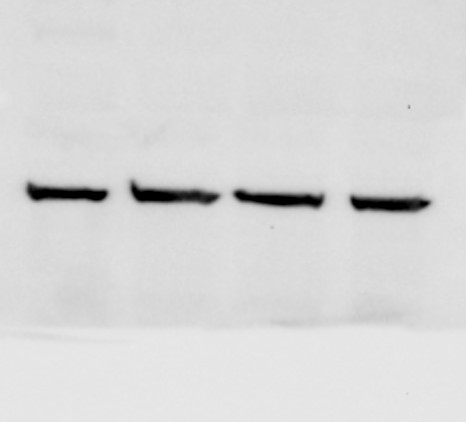

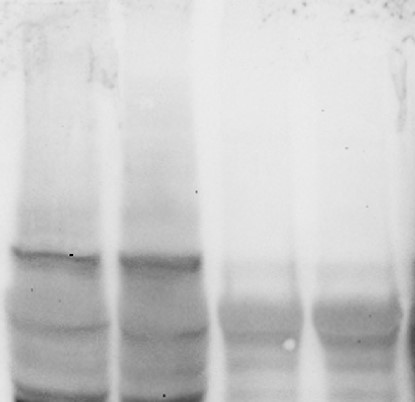

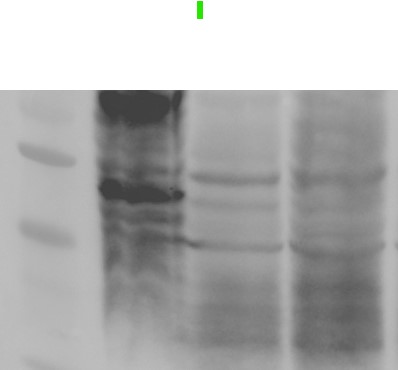


PACT

KDa

50

25

75

KDa

50

37

75

KDa

50

37

25

75

b-actin

P-PKR

Supplement: Supplementary file 6 — Source data Fig. 3 [file 44318_2024_292_MOESM6_ESM.zip › Figure 3/3E/README.docx]

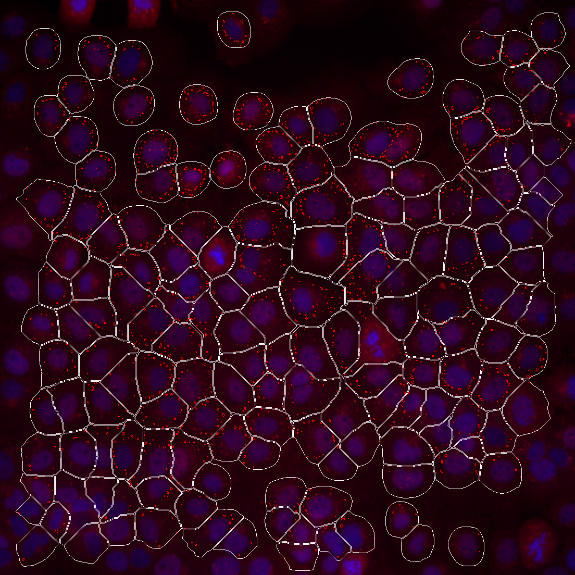

Supplement: Supplementary file 6 — Source data Fig. 3 [file 44318_2024_292_MOESM6_ESM.zip › Figure 3/3E/SCR-LLOMe.tif]

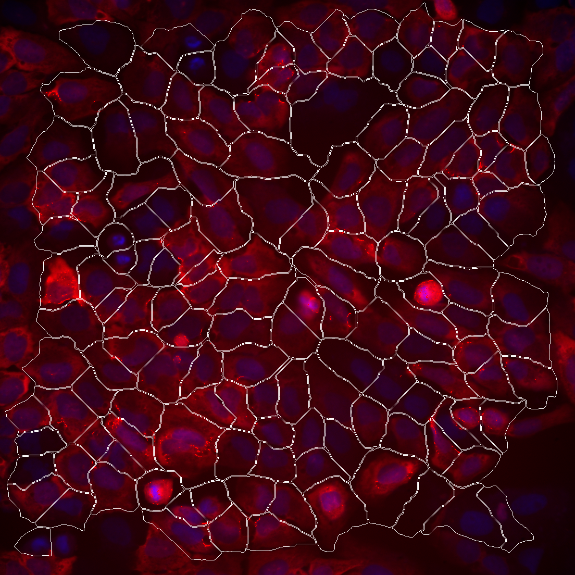

Supplement: Supplementary file 6 — Source data Fig. 3 [file 44318_2024_292_MOESM6_ESM.zip › Figure 3/3E/SCR-NT.tif]

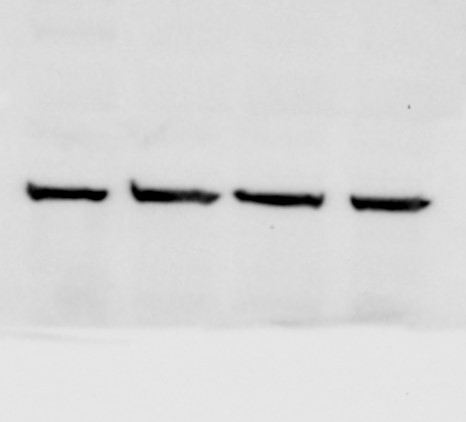

Supplement: Supplementary file 6 — Source data Fig. 3 [file 44318_2024_292_MOESM6_ESM.zip › Figure 3/3E/WB-b-actin.jpg]

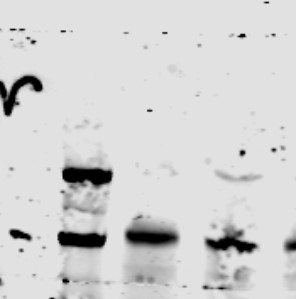

Supplement: Supplementary file 6 — Source data Fig. 3 [file 44318_2024_292_MOESM6_ESM.zip › Figure 3/3E/WB-p-eIF2a.jpg]

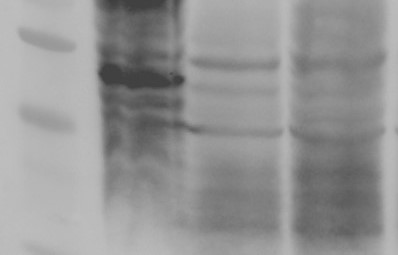

Supplement: Supplementary file 6 — Source data Fig. 3 [file 44318_2024_292_MOESM6_ESM.zip › Figure 3/3E/WB-P-PKR.jpg]

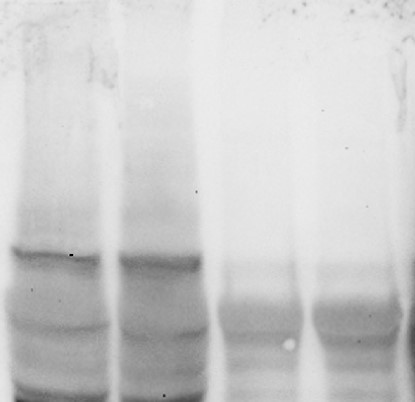

Supplement: Supplementary file 6 — Source data Fig. 3 [file 44318_2024_292_MOESM6_ESM.zip › Figure 3/3E/WB-PACT.jpg]

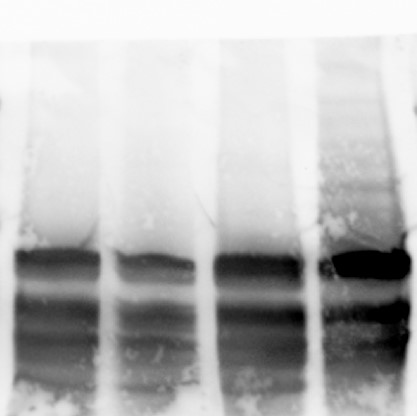

Supplement: Supplementary file 6 — Source data Fig. 3 [file 44318_2024_292_MOESM6_ESM.zip › Figure 3/3F/INPUT-eIF2a.jpg]

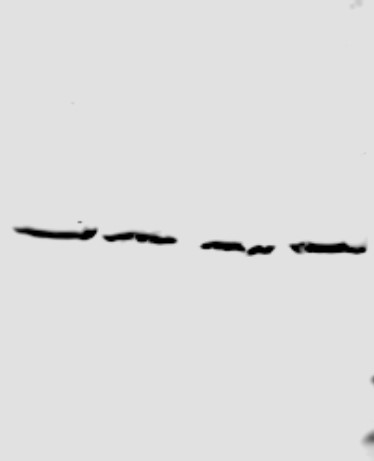

Supplement: Supplementary file 6 — Source data Fig. 3 [file 44318_2024_292_MOESM6_ESM.zip › Figure 3/3F/INPUT-LAMP2.jpg]
