# Supplementary material for: Calcium signaling from damaged lysosomes induces cytoprotective stress granules
Source: EMBO J. 2024 Nov 12;43(24):6410–43. doi: 10.1038/s44318-024-00292-1 (PMC11649789; doi:10.1038/s44318-024-00292-1)
Supplement: Supplementary file 5 — Source data Fig. 2 [file 44318_2024_292_MOESM5_ESM.zip › Figure 2/2B/README.docx]

eIF2a^KD^

SCR

LLOMe: - + - +

LLOMe: - + - +

eIF2a^KD^

SCR


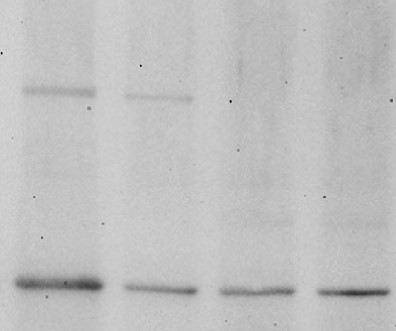

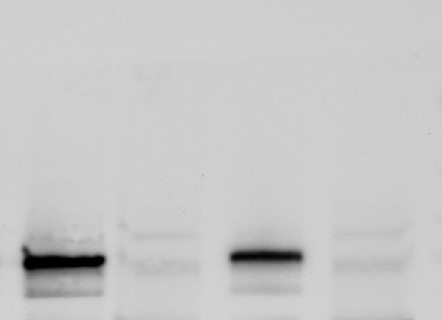


KDa

50

37

25

75

KDa

50

37

25

75

4EBP

P-4EBP


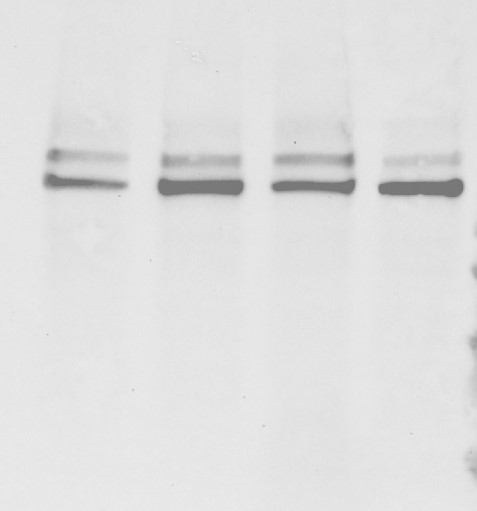


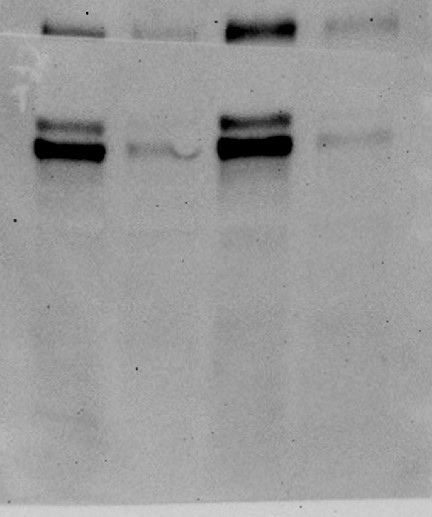


S6K

P-S6K

KDa

50

37

25

75

KDa

50

37

25

75

SCR


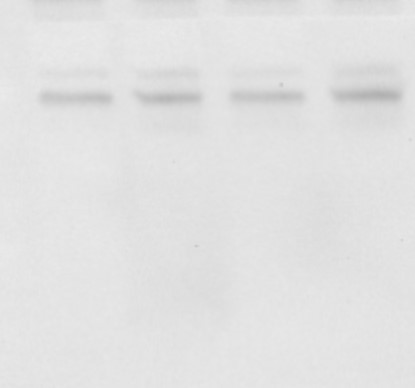


KDa

120

95

25

150

KDa

120

95

25

150


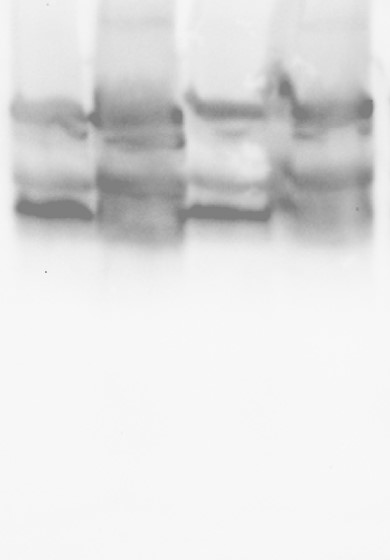


ULK1

P-ULK1

KDa

50

37

25

75


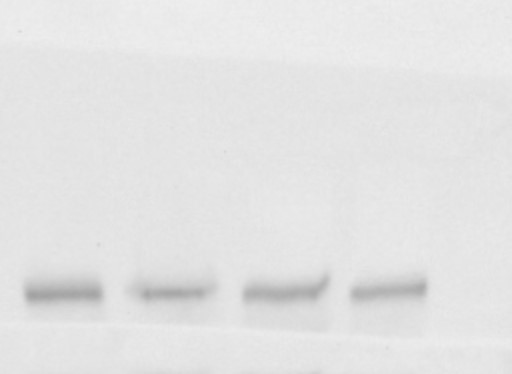

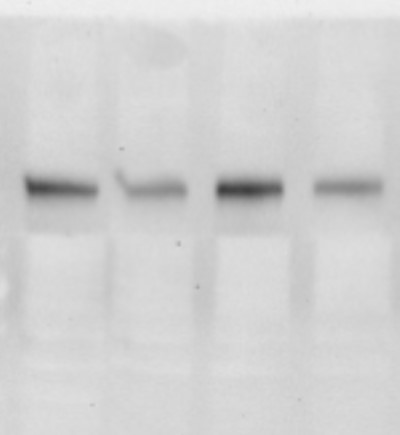


KDa

50

75

P-TFEB


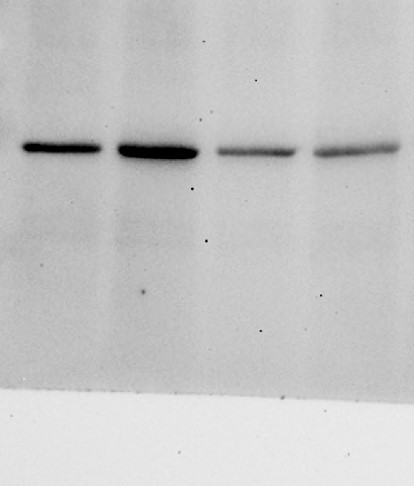

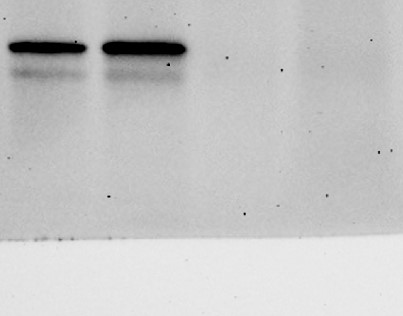


KDa

37

25

b-actin

eIF2a

TFEB

KDa

50

37

25

75
