# Supplementary material for: Calcium signaling from damaged lysosomes induces cytoprotective stress granules
Source: EMBO J. 2024 Nov 12;43(24):6410–43. doi: 10.1038/s44318-024-00292-1 (PMC11649789; doi:10.1038/s44318-024-00292-1)
Supplement: Supplementary file 6 — Source data Fig. 3 [file 44318_2024_292_MOESM6_ESM.zip › Figure 3/3F/README.docx]

LYSOIP:

LLOMe: - - + +


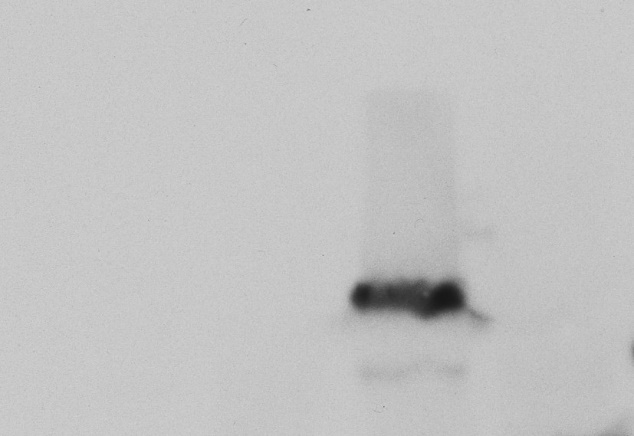


KDa

50

37

75

P-PKR


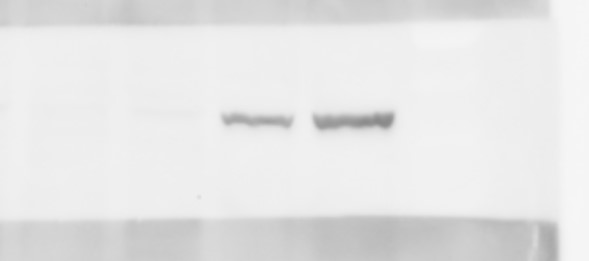

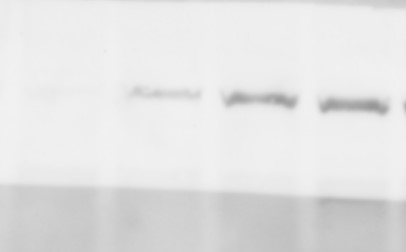

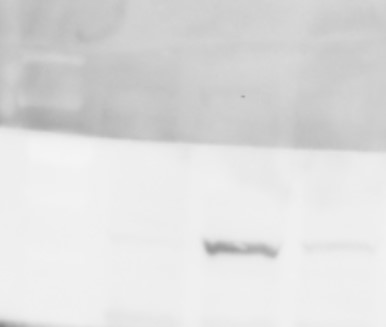


KDa

50

37

75

eIF2a

KDa

50

37

75

PKR

KDa

50

37

75

P-eIF2a


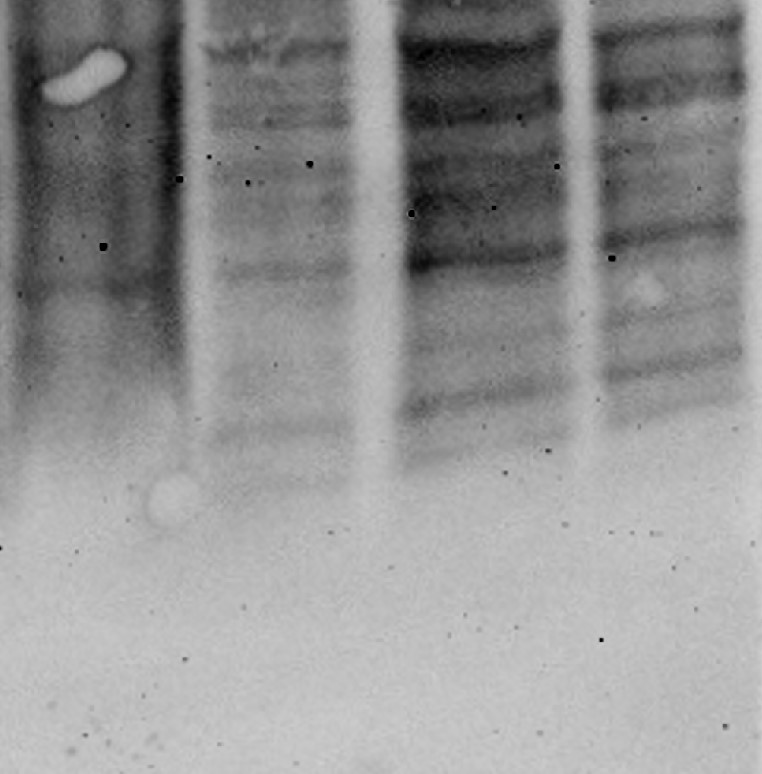


KDa

50

37

75

PACT


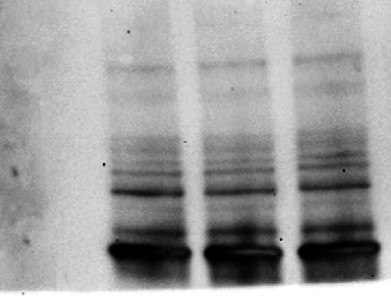


KDa

150

100

200

LAMP2

LLOMe: - - + +

INPUT:


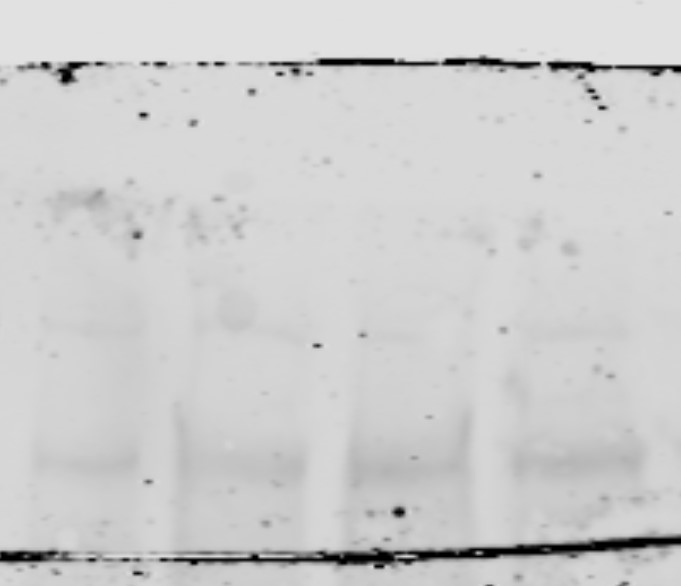


KDa

50

37

75

PKR


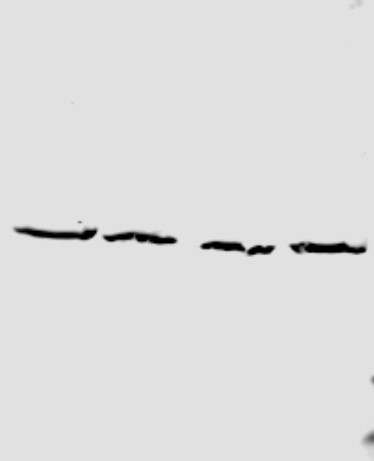

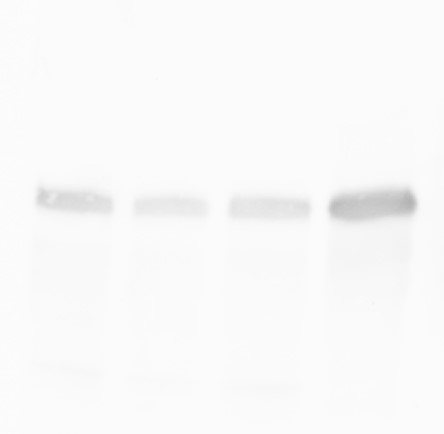

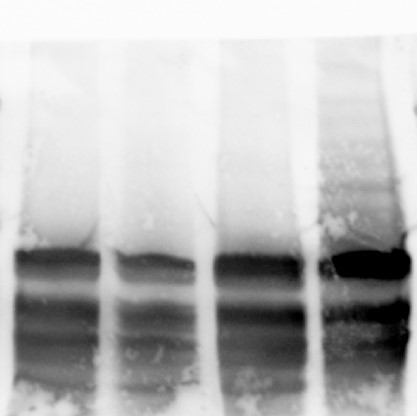


KDa

50

37

75

LAMP2

KDa

150

100

200

PACT

KDa

50

37

75

eIF2a
