# Supplementary figures and images for: Calcium signaling from damaged lysosomes induces cytoprotective stress granules (part 2 of 3)
Source: EMBO J. 2024 Nov 12;43(24):6410–43. doi: 10.1038/s44318-024-00292-1 (PMC11649789; doi:10.1038/s44318-024-00292-1)

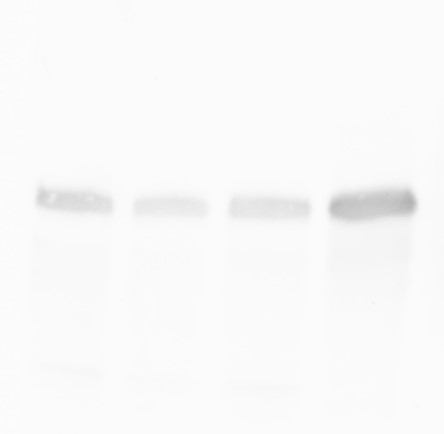

Supplement: Supplementary file 6 — Source data Fig. 3 [file 44318_2024_292_MOESM6_ESM.zip › Figure 3/3F/INPUT-PACT.jpg]

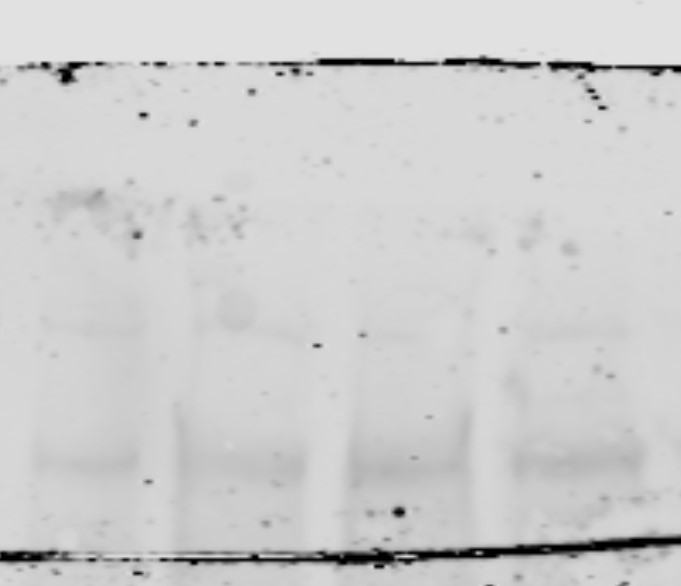

Supplement: Supplementary file 6 — Source data Fig. 3 [file 44318_2024_292_MOESM6_ESM.zip › Figure 3/3F/INPUT-PKR.jpg]

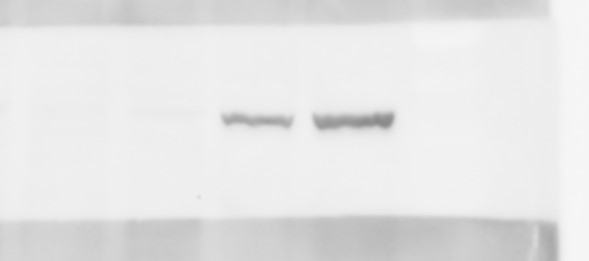

Supplement: Supplementary file 6 — Source data Fig. 3 [file 44318_2024_292_MOESM6_ESM.zip › Figure 3/3F/LYSOIP-eIF2a.jpg]

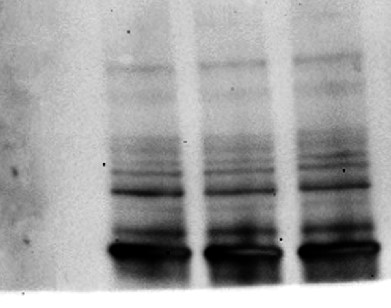

Supplement: Supplementary file 6 — Source data Fig. 3 [file 44318_2024_292_MOESM6_ESM.zip › Figure 3/3F/LYSOIP-LAMP2.jpg]

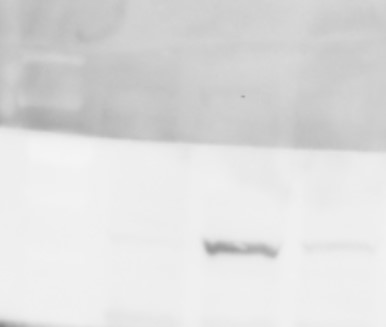

Supplement: Supplementary file 6 — Source data Fig. 3 [file 44318_2024_292_MOESM6_ESM.zip › Figure 3/3F/LYSOIP-P-eIF2a.jpg]

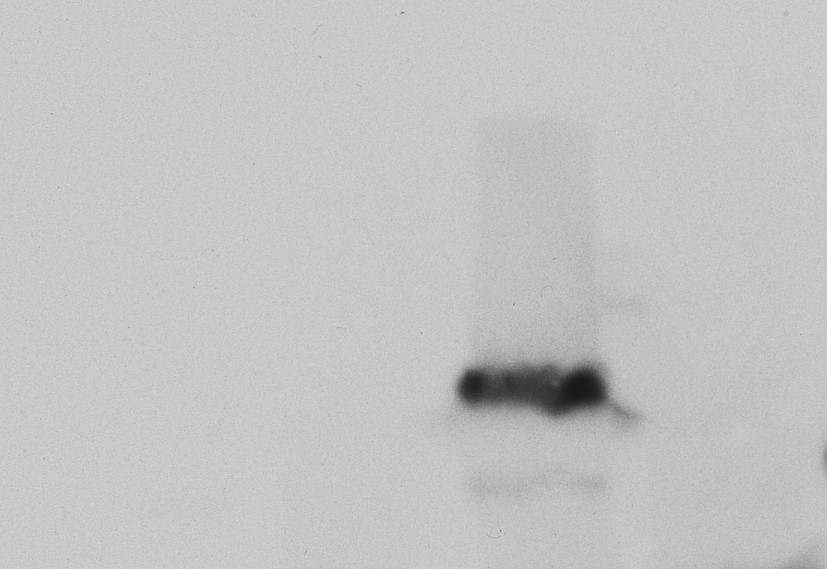

Supplement: Supplementary file 6 — Source data Fig. 3 [file 44318_2024_292_MOESM6_ESM.zip › Figure 3/3F/LYSOIP-P-PKR.jpg]

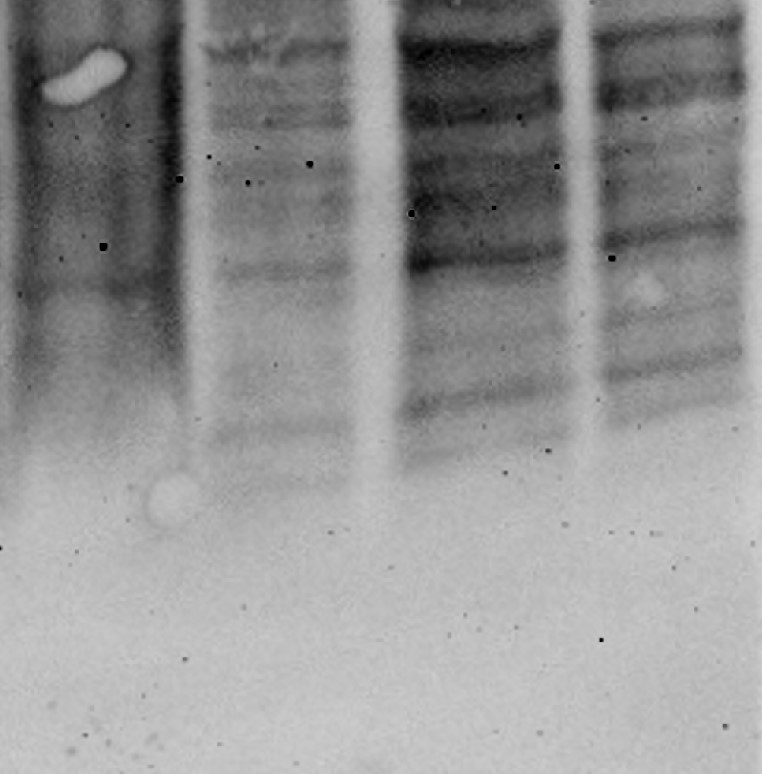

Supplement: Supplementary file 6 — Source data Fig. 3 [file 44318_2024_292_MOESM6_ESM.zip › Figure 3/3F/LYSOIP-PACT.jpg]

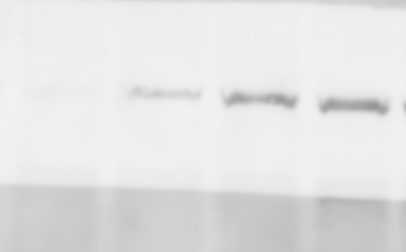

Supplement: Supplementary file 6 — Source data Fig. 3 [file 44318_2024_292_MOESM6_ESM.zip › Figure 3/3F/LYSOIP-PKR.jpg]

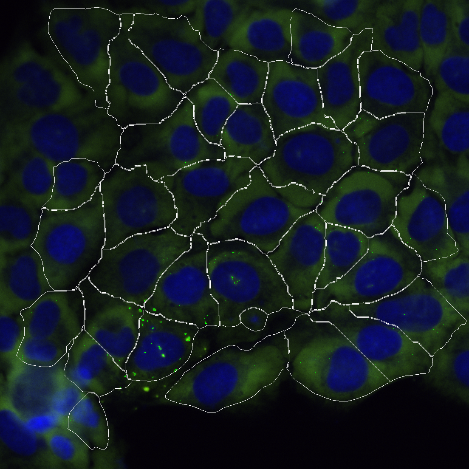

Supplement: Supplementary file 7 — Source data Fig. 4 [file 44318_2024_292_MOESM7_ESM.zip › Figure 4/4A/ALIXKD-LLOMe.tif]

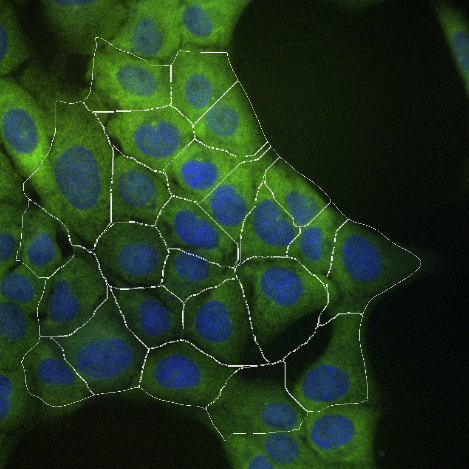

Supplement: Supplementary file 7 — Source data Fig. 4 [file 44318_2024_292_MOESM7_ESM.zip › Figure 4/4A/ALIXKD-NT.tif]

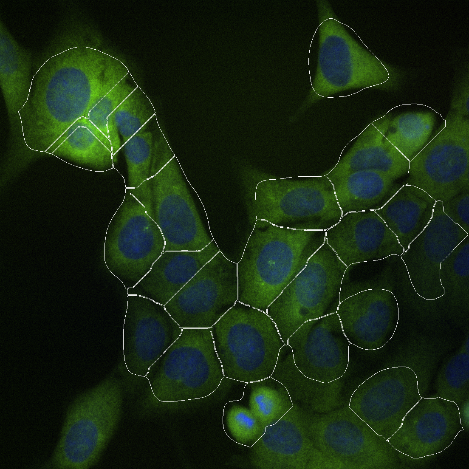

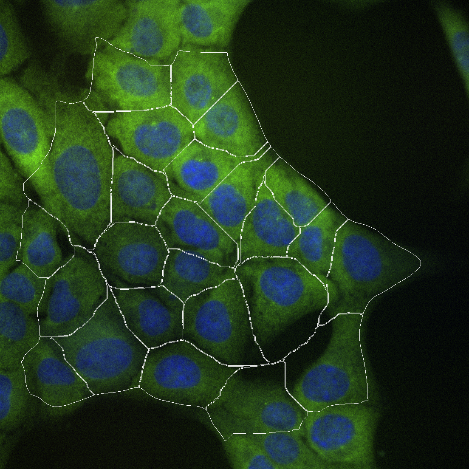

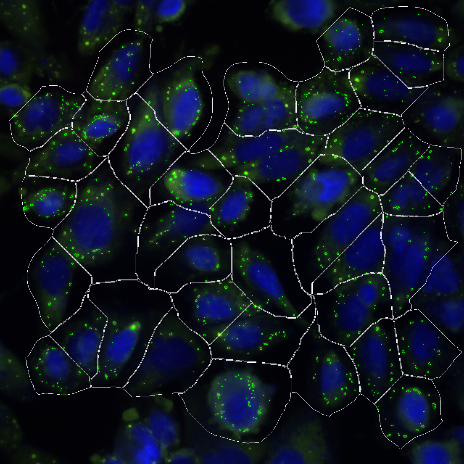

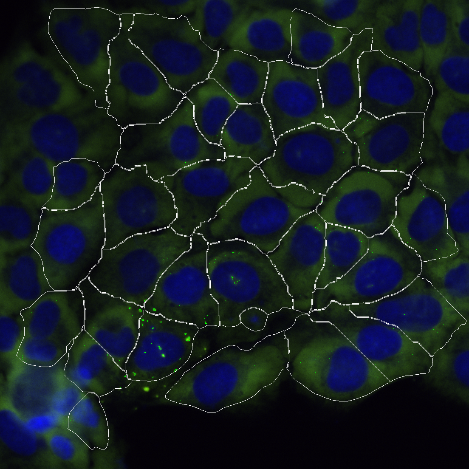


ALIXKD -LLOMe

SCR-LLOMe

ALIXKD -NT

SCR-NT

Supplement: Supplementary file 7 — Source data Fig. 4 [file 44318_2024_292_MOESM7_ESM.zip › Figure 4/4A/README.docx]

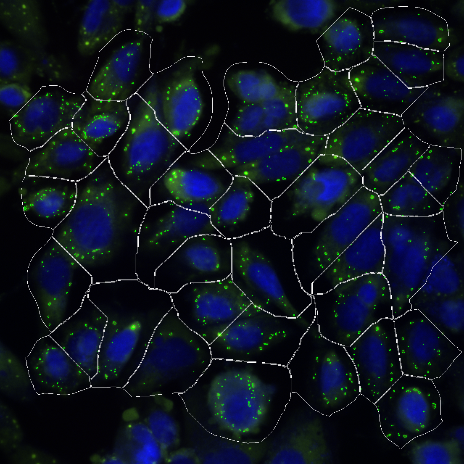

Supplement: Supplementary file 7 — Source data Fig. 4 [file 44318_2024_292_MOESM7_ESM.zip › Figure 4/4A/SCR-LLOMe.tif]

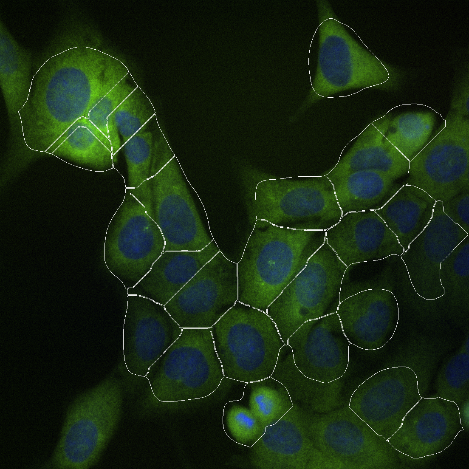

Supplement: Supplementary file 7 — Source data Fig. 4 [file 44318_2024_292_MOESM7_ESM.zip › Figure 4/4A/SCR-NT.tif]

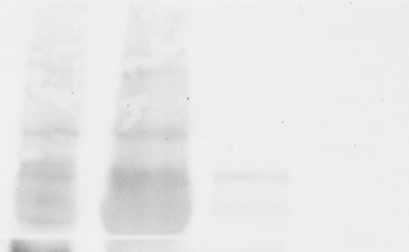

Supplement: Supplementary file 7 — Source data Fig. 4 [file 44318_2024_292_MOESM7_ESM.zip › Figure 4/4B/ALIX.jpg]

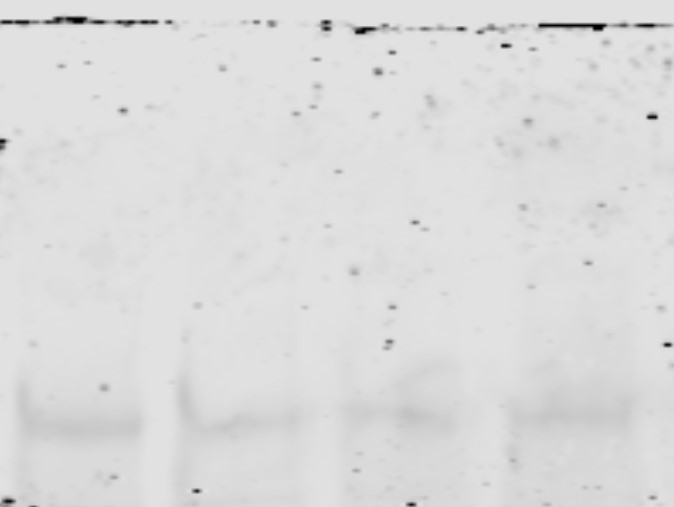

Supplement: Supplementary file 7 — Source data Fig. 4 [file 44318_2024_292_MOESM7_ESM.zip › Figure 4/4B/b-actin.jpg]

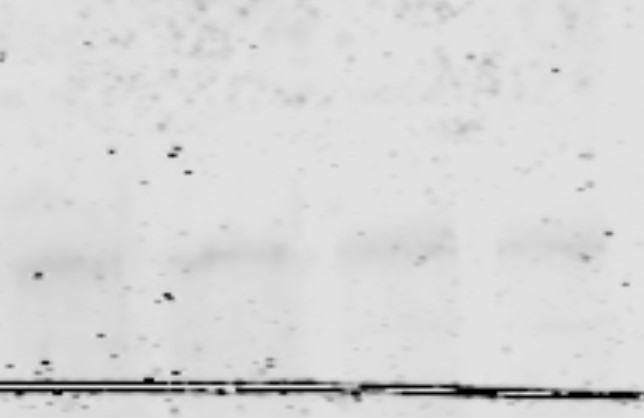

Supplement: Supplementary file 7 — Source data Fig. 4 [file 44318_2024_292_MOESM7_ESM.zip › Figure 4/4B/eIF2a.jpg]

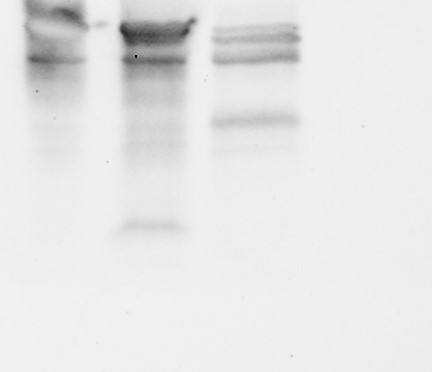

Supplement: Supplementary file 7 — Source data Fig. 4 [file 44318_2024_292_MOESM7_ESM.zip › Figure 4/4B/P-eIF2a.jpg]

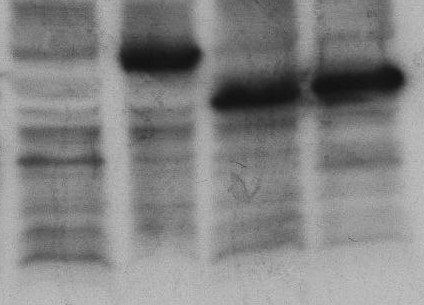

Supplement: Supplementary file 7 — Source data Fig. 4 [file 44318_2024_292_MOESM7_ESM.zip › Figure 4/4B/P-PKR.jpg]

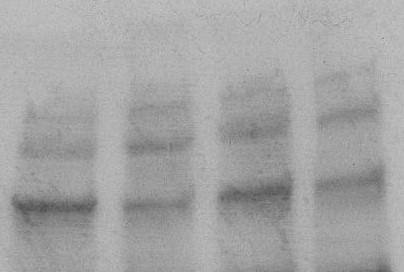

Supplement: Supplementary file 7 — Source data Fig. 4 [file 44318_2024_292_MOESM7_ESM.zip › Figure 4/4B/PKR.jpg]

ALIXKD

SCR

LLOMe: - + - +


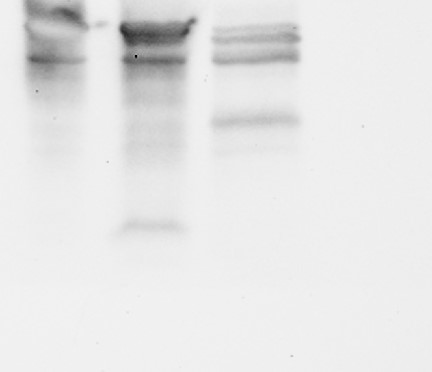


KDa

50

37

75

P-eIF2a


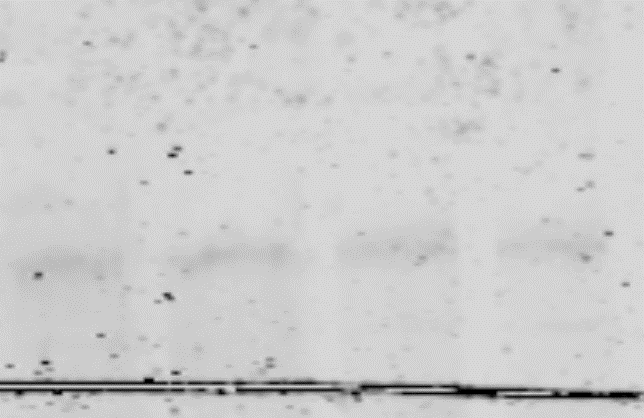


KDa

50

37

75

eIF2a

KDa

50

37

75


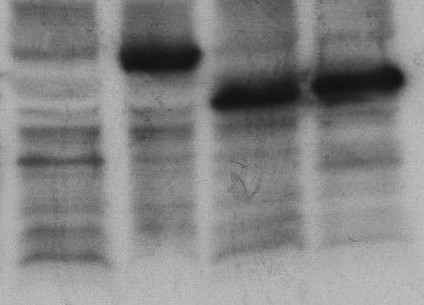


P-PKR


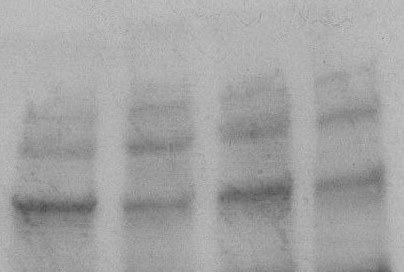


KDa

50

75

PKR

KDa

150

100

200


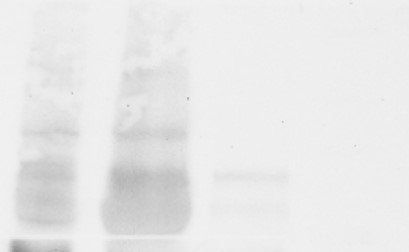


ALIX


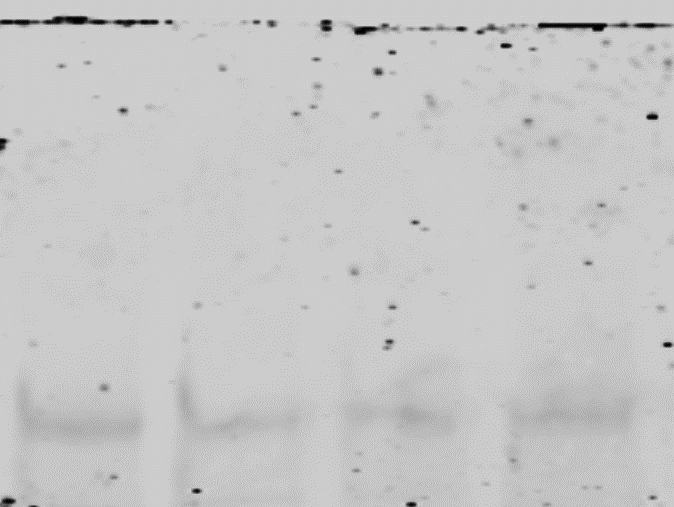


KDa

50

37

75

b-actin

Supplement: Supplementary file 7 — Source data Fig. 4 [file 44318_2024_292_MOESM7_ESM.zip › Figure 4/4B/README.docx]

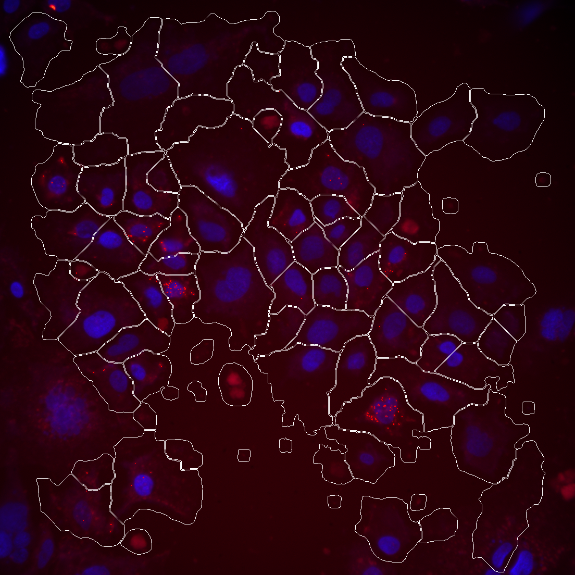

Supplement: Supplementary file 7 — Source data Fig. 4 [file 44318_2024_292_MOESM7_ESM.zip › Figure 4/4C/ALIXKD-LLOMe.tif]

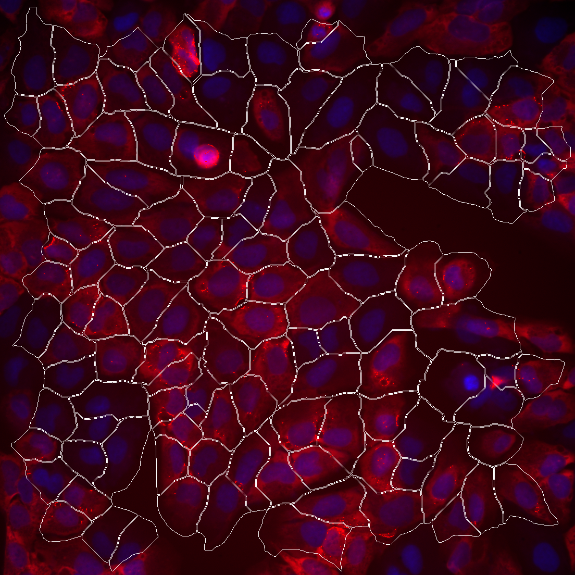

Supplement: Supplementary file 7 — Source data Fig. 4 [file 44318_2024_292_MOESM7_ESM.zip › Figure 4/4C/ALIXKD-NT.tif]

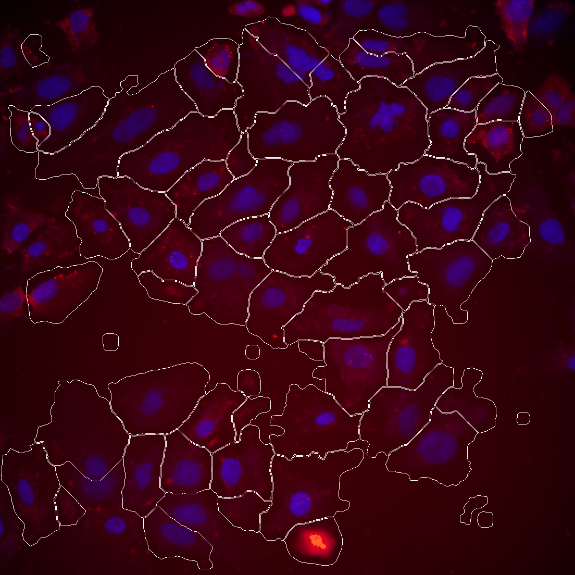

Supplement: Supplementary file 7 — Source data Fig. 4 [file 44318_2024_292_MOESM7_ESM.zip › Figure 4/4C/ALIXKD-TSG101KD-LLOME.tif]

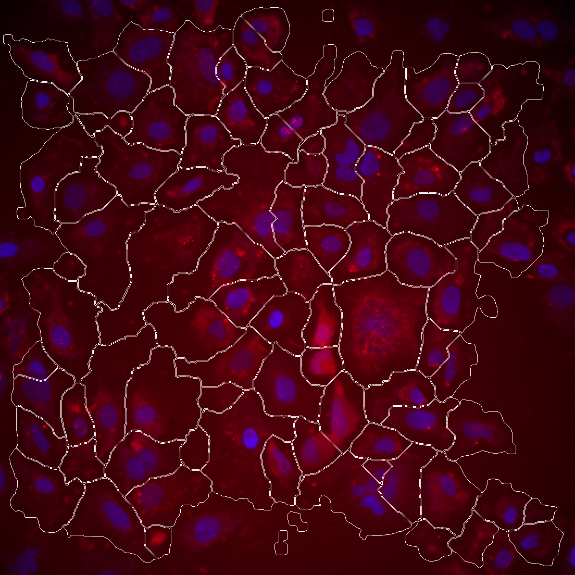

Supplement: Supplementary file 7 — Source data Fig. 4 [file 44318_2024_292_MOESM7_ESM.zip › Figure 4/4C/ALIXKD-TSG101KD-NT.tif]

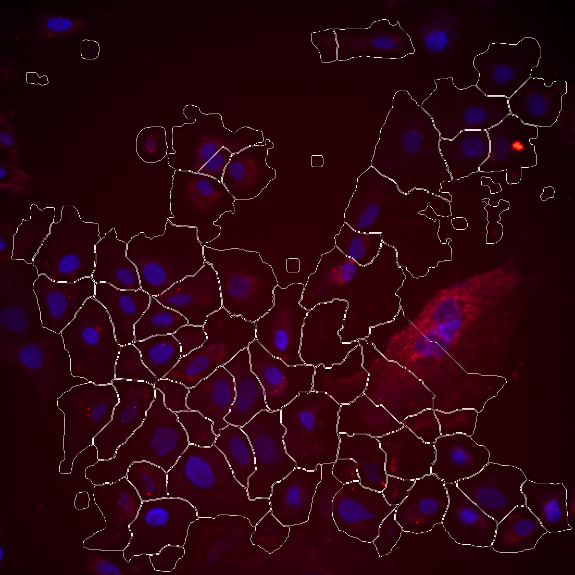

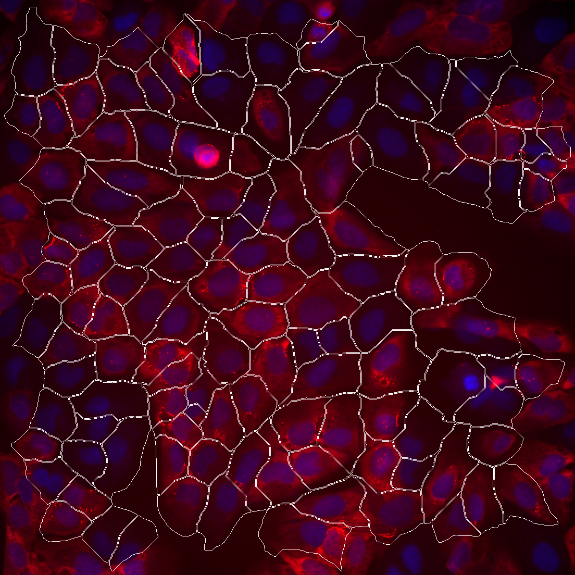


ALIXKD-NT

SCR-NT


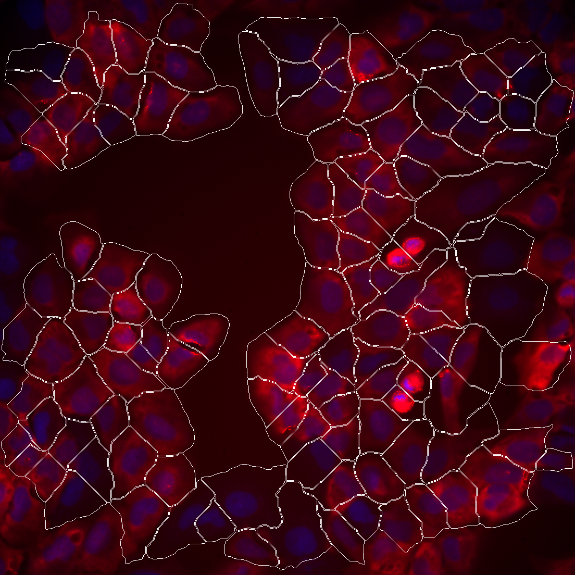

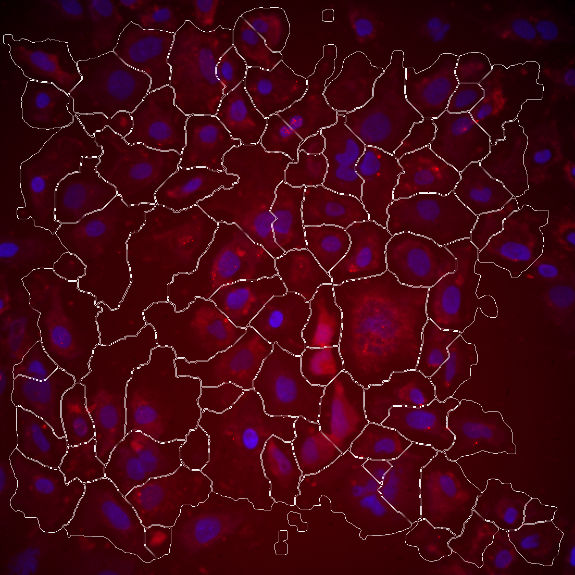


ALIXKD-TSG101KD-NT

TSG101KD-NT


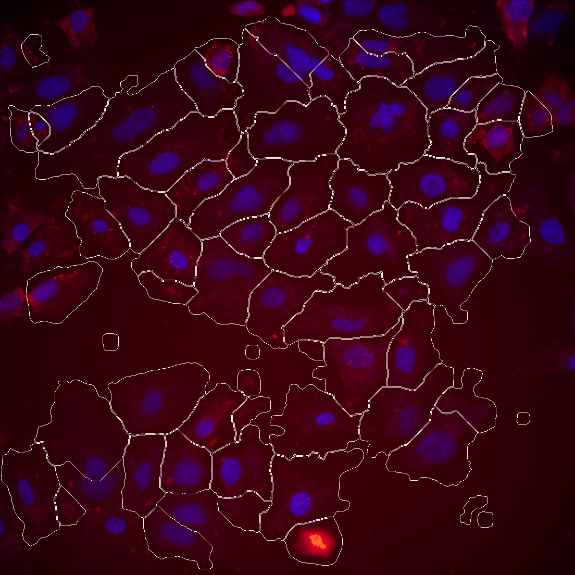

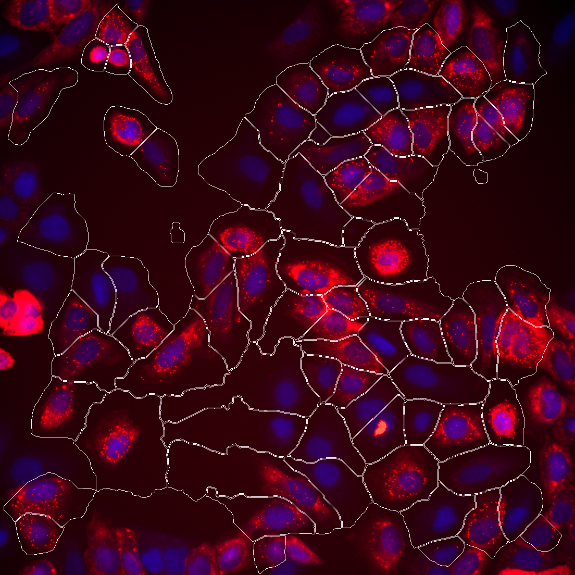

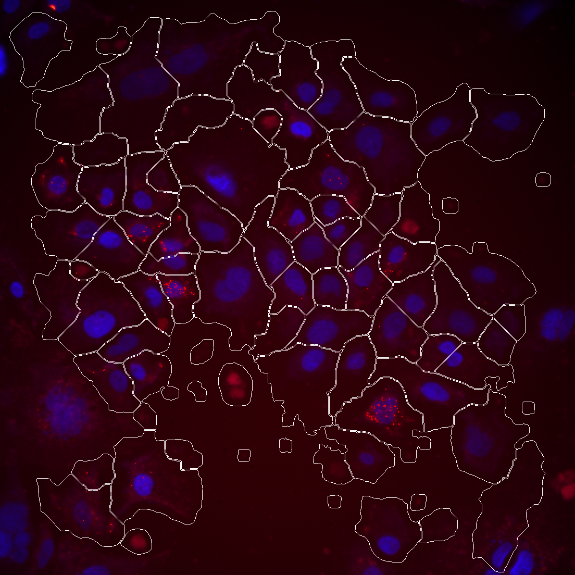

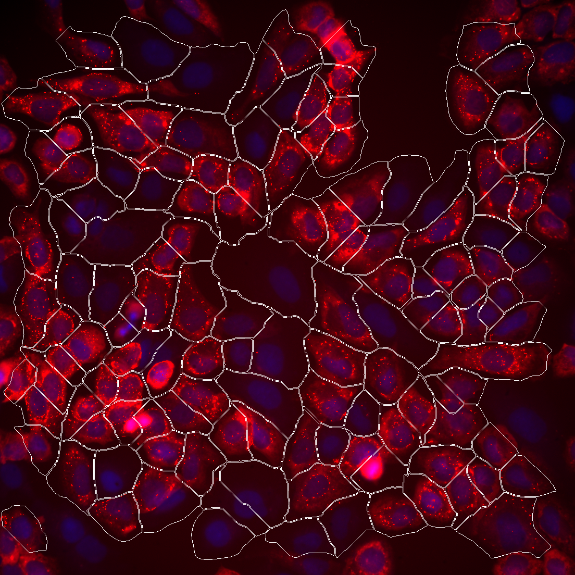


TSG101KD-LLOMe

SCR-LLOMe

ALIXKD -LLOMe

ALIXKD-TSG101KD -LLOMe

Supplement: Supplementary file 7 — Source data Fig. 4 [file 44318_2024_292_MOESM7_ESM.zip › Figure 4/4C/README.docx]

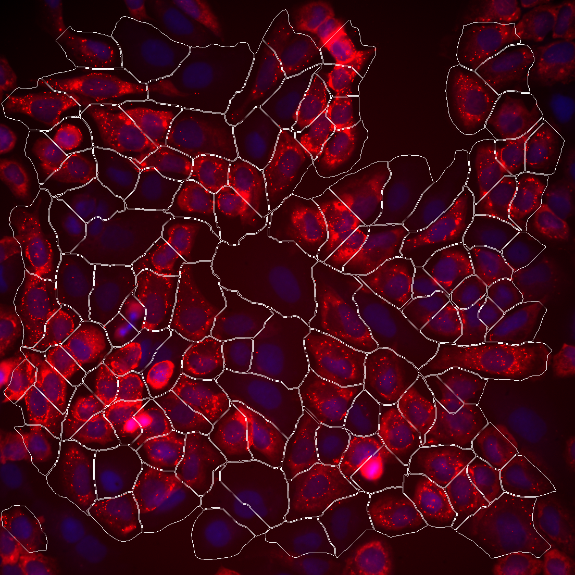

Supplement: Supplementary file 7 — Source data Fig. 4 [file 44318_2024_292_MOESM7_ESM.zip › Figure 4/4C/SCR-LLOMe.tif]

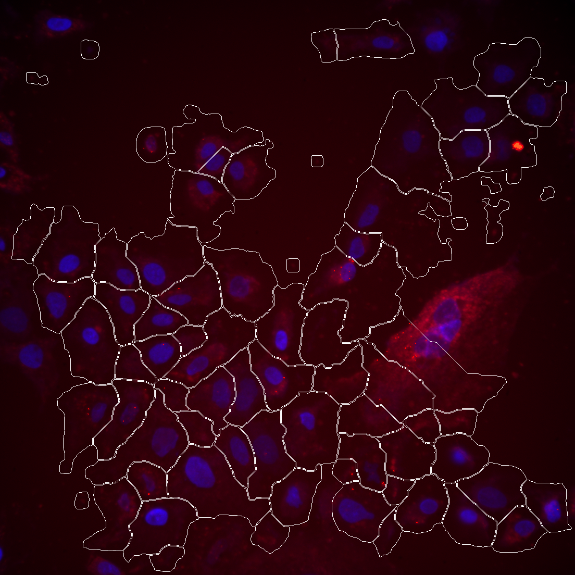

Supplement: Supplementary file 7 — Source data Fig. 4 [file 44318_2024_292_MOESM7_ESM.zip › Figure 4/4C/SCR-NT.tif]

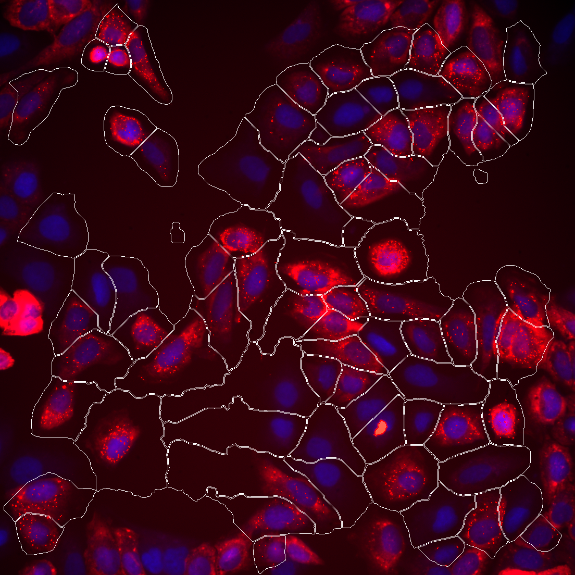

Supplement: Supplementary file 7 — Source data Fig. 4 [file 44318_2024_292_MOESM7_ESM.zip › Figure 4/4C/TSG101KD-LLOMe.tif]

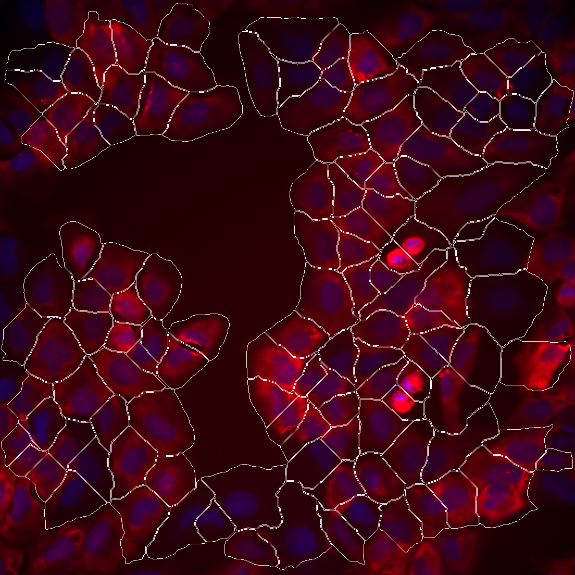

Supplement: Supplementary file 7 — Source data Fig. 4 [file 44318_2024_292_MOESM7_ESM.zip › Figure 4/4C/TSG101KD-NT.tif]

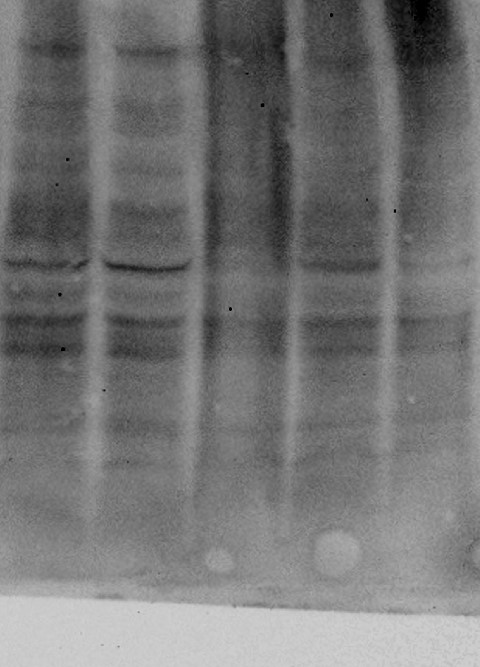

Supplement: Supplementary file 7 — Source data Fig. 4 [file 44318_2024_292_MOESM7_ESM.zip › Figure 4/4D/ALIX.jpg]

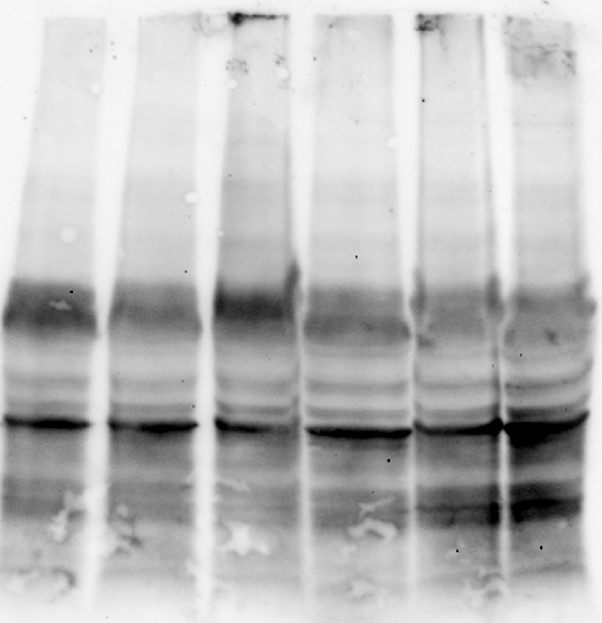

Supplement: Supplementary file 7 — Source data Fig. 4 [file 44318_2024_292_MOESM7_ESM.zip › Figure 4/4D/b-actin.jpg]

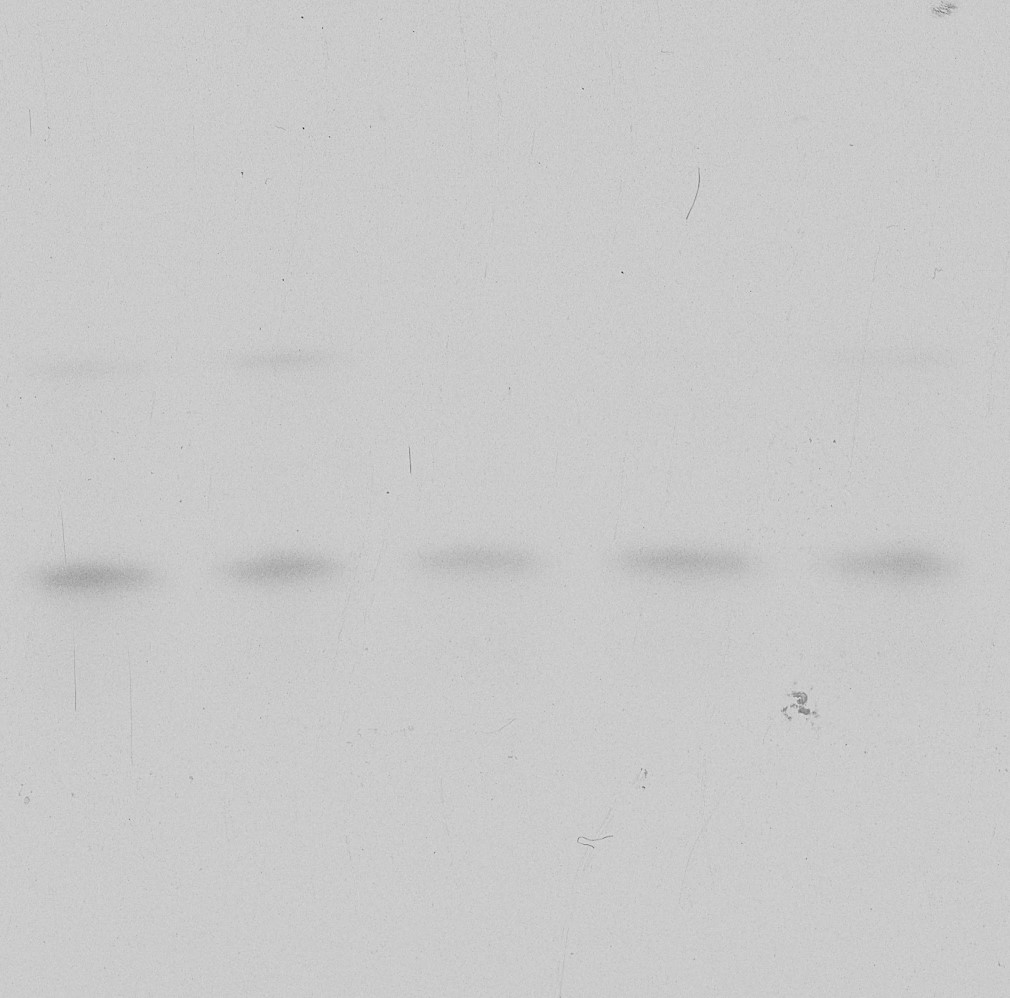

Supplement: Supplementary file 7 — Source data Fig. 4 [file 44318_2024_292_MOESM7_ESM.zip › Figure 4/4D/eIF2a.jpg]

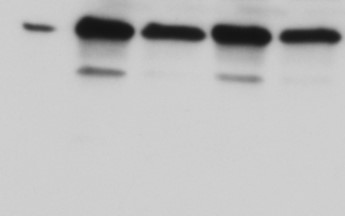

Supplement: Supplementary file 7 — Source data Fig. 4 [file 44318_2024_292_MOESM7_ESM.zip › Figure 4/4D/P-eIF2a.jpg]

SCR

ALIXKD

TSG101KD

TSG101KD

ALIXKD

LLOMe: - + + + +

KDa

50

37

75


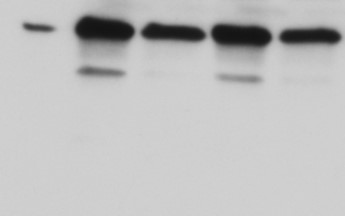


P-eIF2a


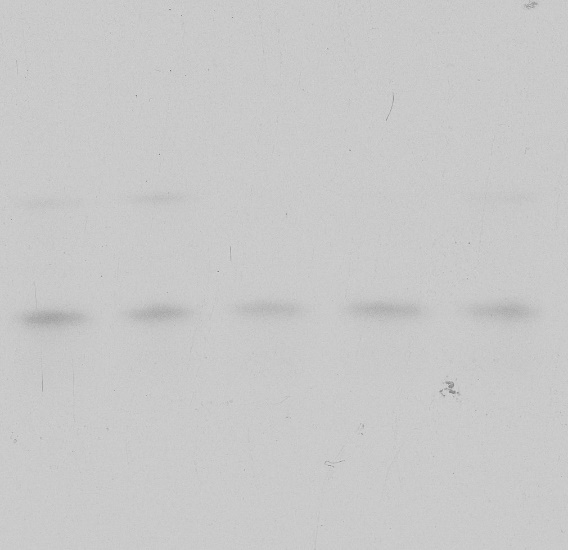


KDa

50

37

75

eIF2a


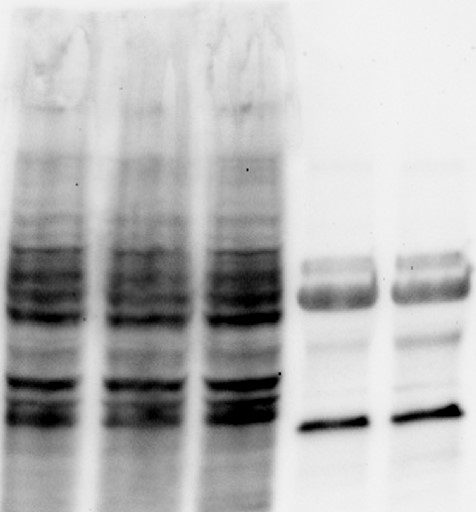


KDa

50

37

75

TSG101


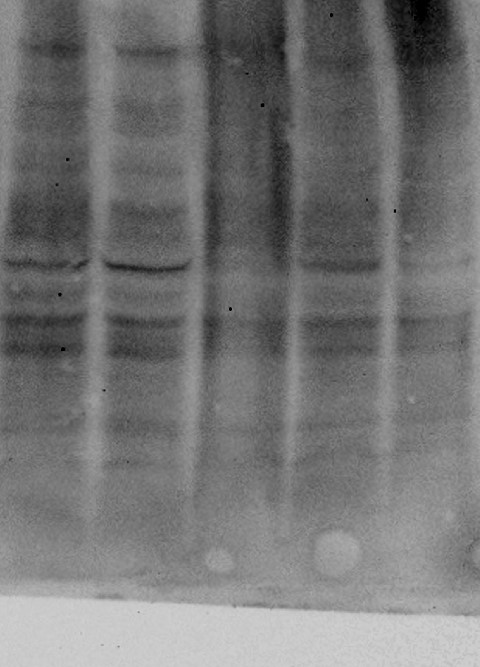


KDa

150

100

200

ALIX


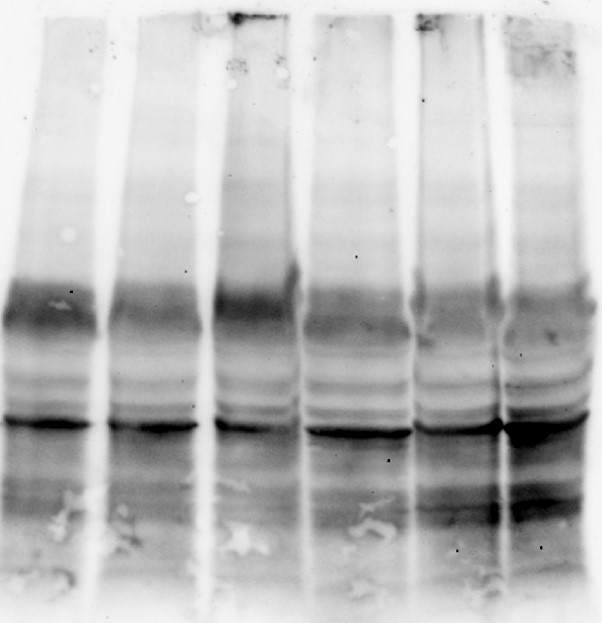


KDa

50

37

75

b-actin

Supplement: Supplementary file 7 — Source data Fig. 4 [file 44318_2024_292_MOESM7_ESM.zip › Figure 4/4D/README.docx]

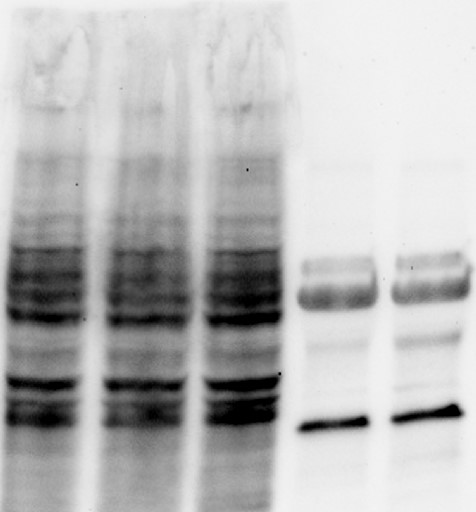

Supplement: Supplementary file 7 — Source data Fig. 4 [file 44318_2024_292_MOESM7_ESM.zip › Figure 4/4D/TSG101.jpg]

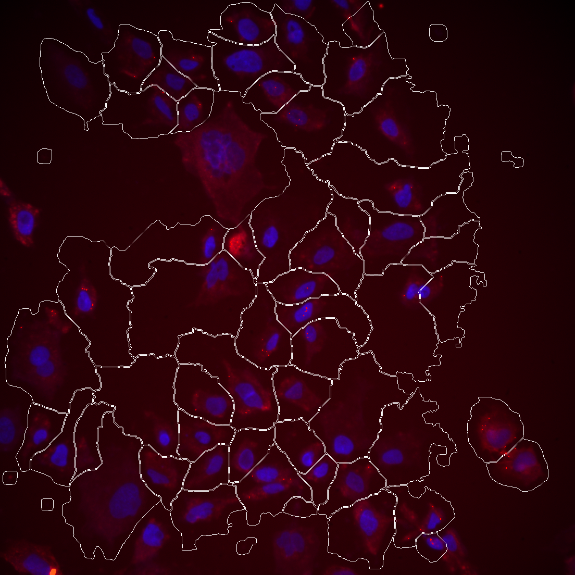

Supplement: Supplementary file 7 — Source data Fig. 4 [file 44318_2024_292_MOESM7_ESM.zip › Figure 4/4E/BAPTA-AM-LLOMe.tif]

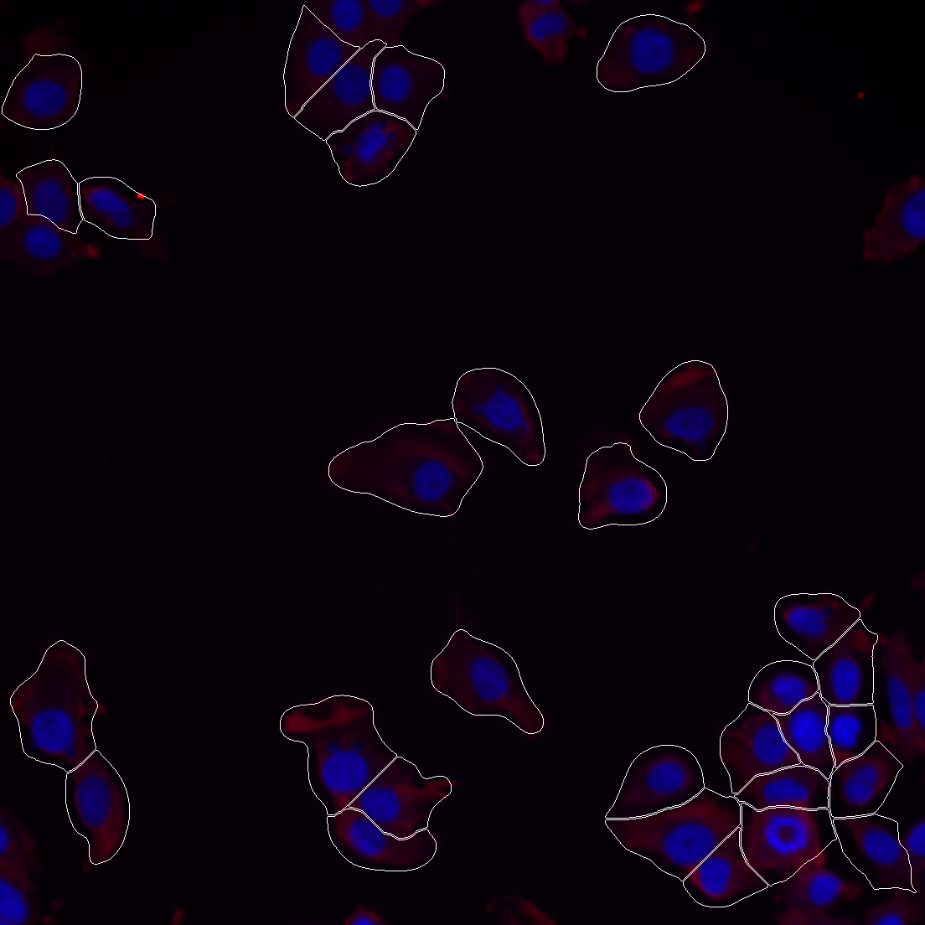

Supplement: Supplementary file 7 — Source data Fig. 4 [file 44318_2024_292_MOESM7_ESM.zip › Figure 4/4E/BAPTA-AM-NT.tif]

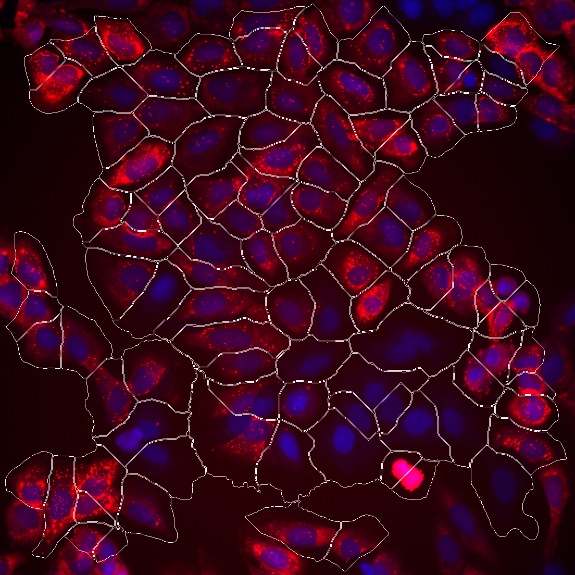

Supplement: Supplementary file 7 — Source data Fig. 4 [file 44318_2024_292_MOESM7_ESM.zip › Figure 4/4E/CTR-LLOMe.tif]

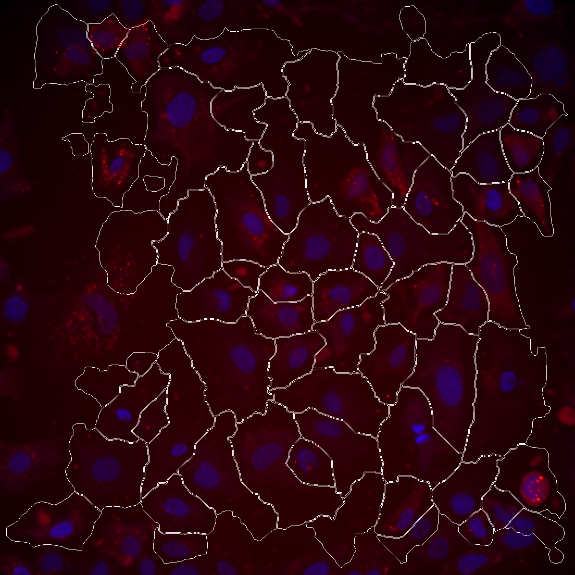

Supplement: Supplementary file 7 — Source data Fig. 4 [file 44318_2024_292_MOESM7_ESM.zip › Figure 4/4E/CTR-NT.tif]

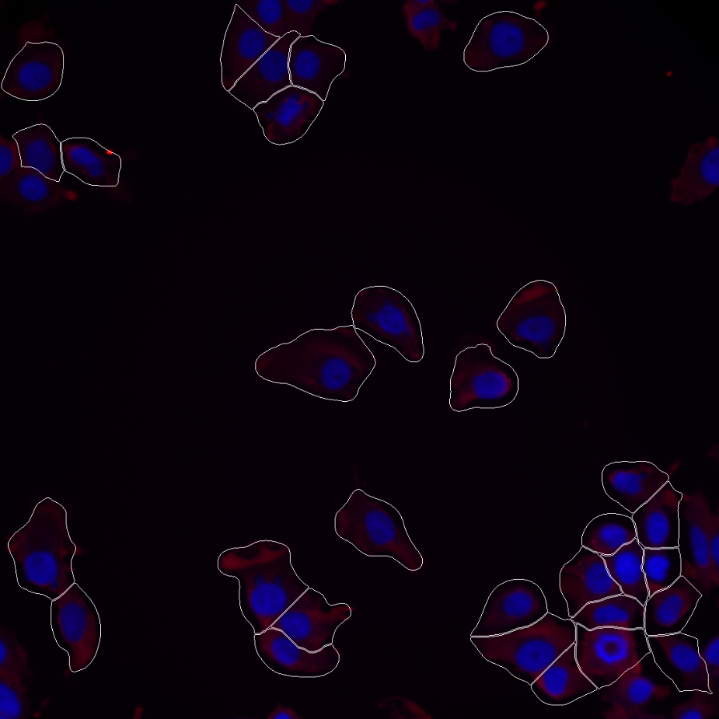

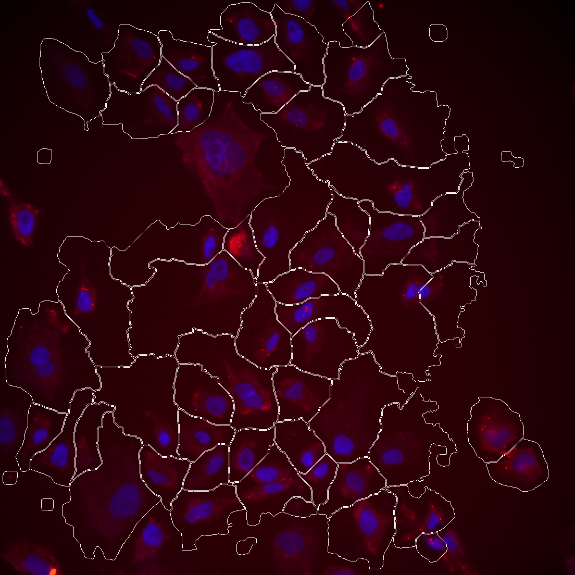

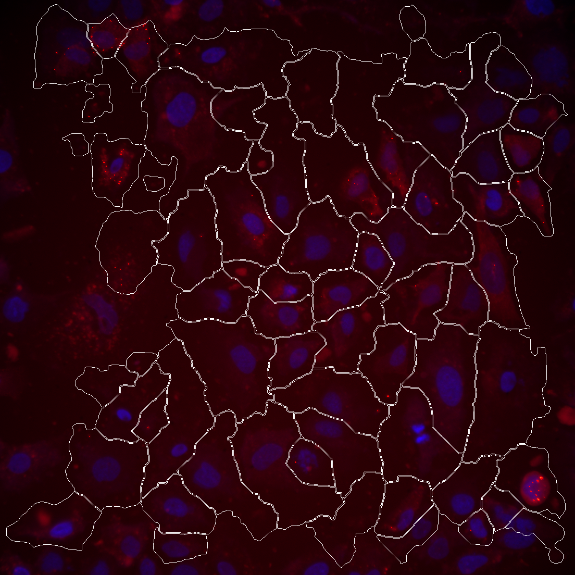

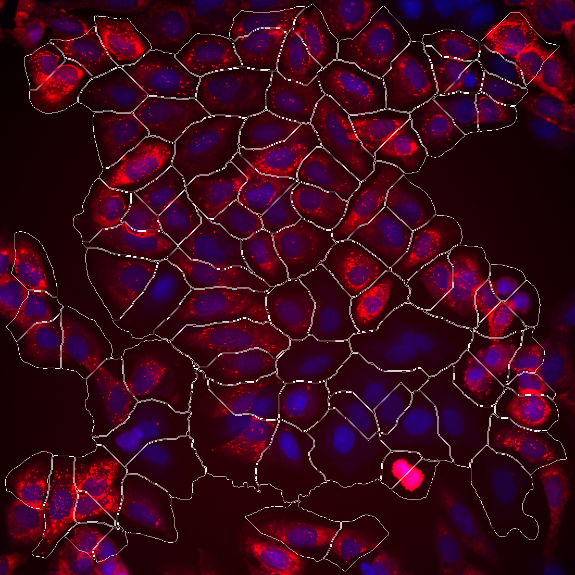


BAPTA-AM -NT

BAPTA-AM -LLOMe

CTR-LLOMe

CTR-NT

BAPTA-AM

CTR


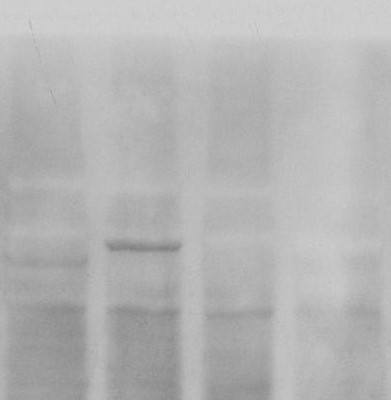


LLOMe: - + - +

KDa

50

37

75

P-eIF2a

KDa

50

37

75


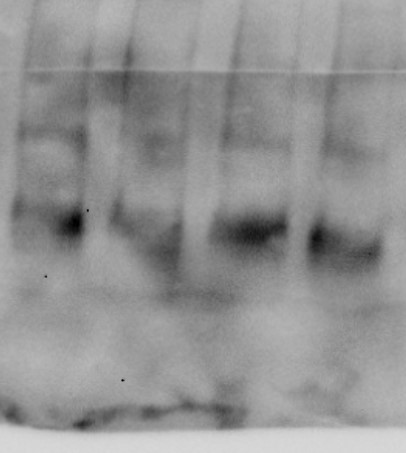


eIF2a

Supplement: Supplementary file 7 — Source data Fig. 4 [file 44318_2024_292_MOESM7_ESM.zip › Figure 4/4E/README.docx]

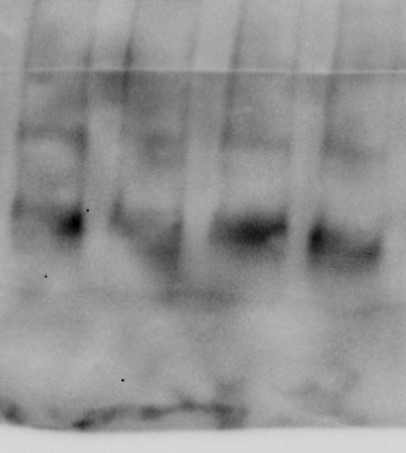

Supplement: Supplementary file 7 — Source data Fig. 4 [file 44318_2024_292_MOESM7_ESM.zip › Figure 4/4E/WB-eIF2a.jpg]

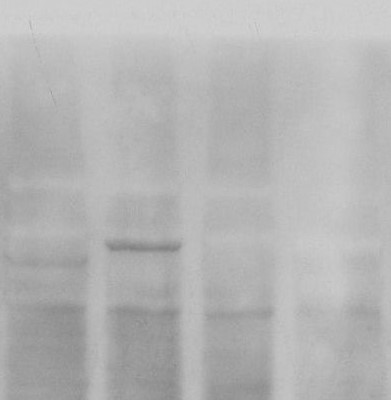

Supplement: Supplementary file 7 — Source data Fig. 4 [file 44318_2024_292_MOESM7_ESM.zip › Figure 4/4E/WB-P-eIF2a.jpg]

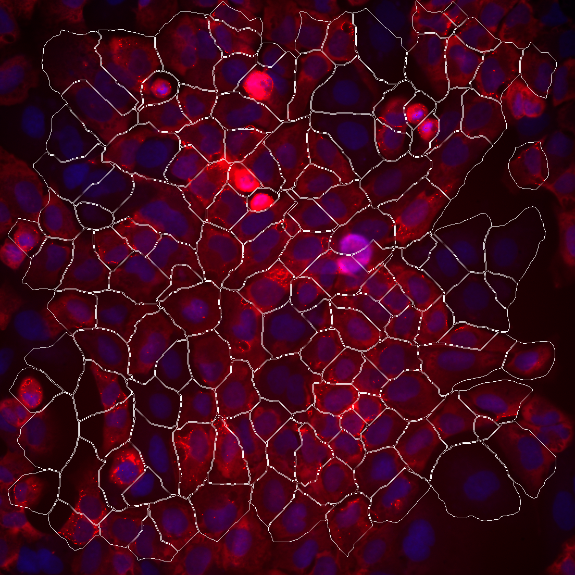

Supplement: Supplementary file 7 — Source data Fig. 4 [file 44318_2024_292_MOESM7_ESM.zip › Figure 4/4F/ALG2KD-LLOMe.tif]

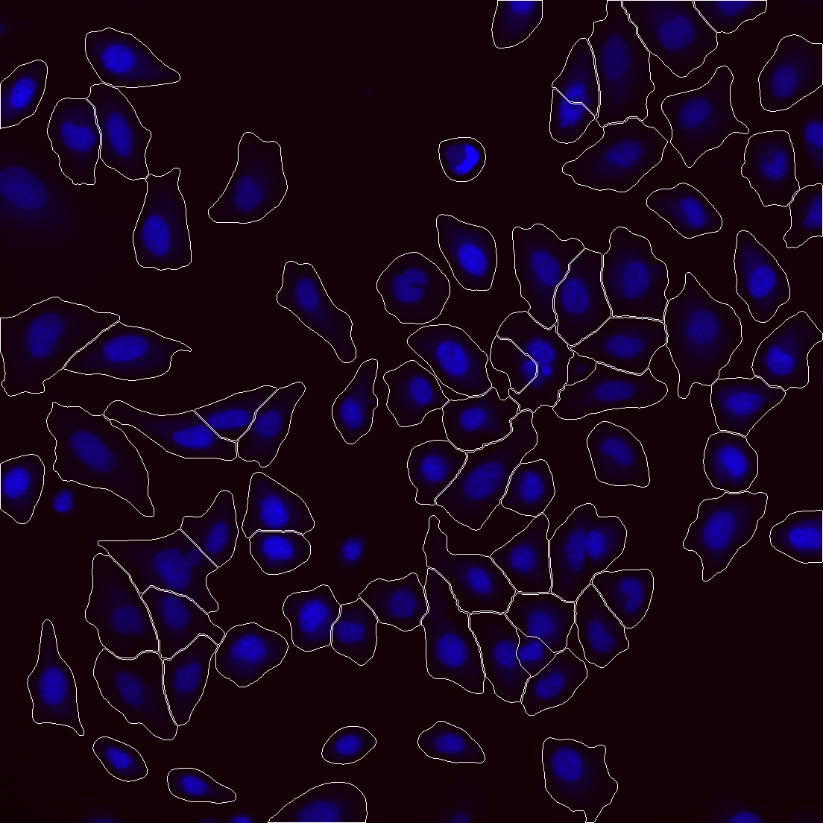

Supplement: Supplementary file 7 — Source data Fig. 4 [file 44318_2024_292_MOESM7_ESM.zip › Figure 4/4F/ALG2KD-NT.tif]

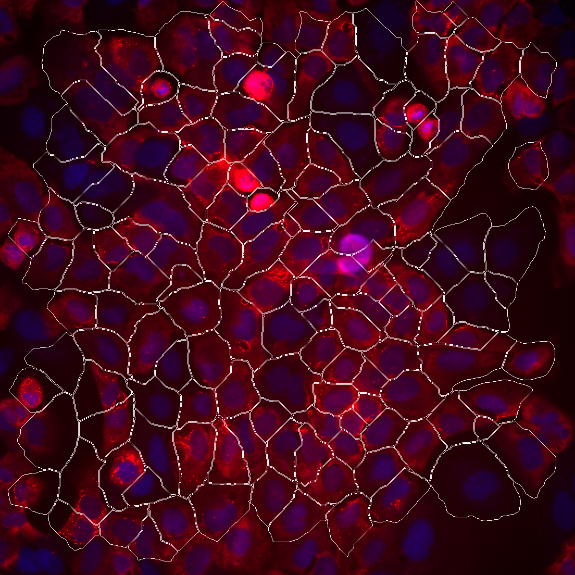

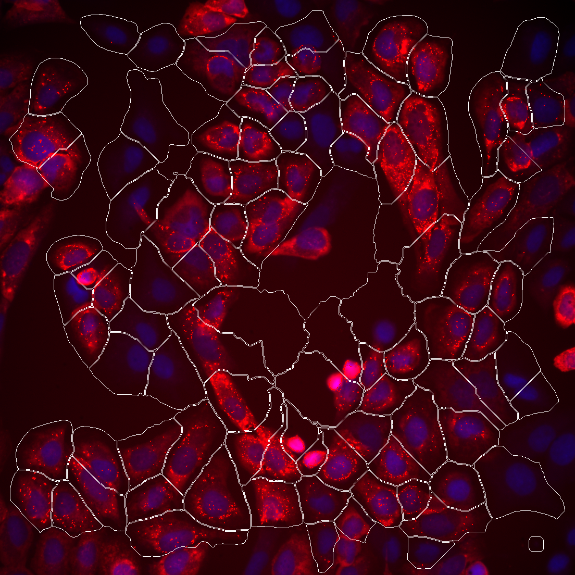

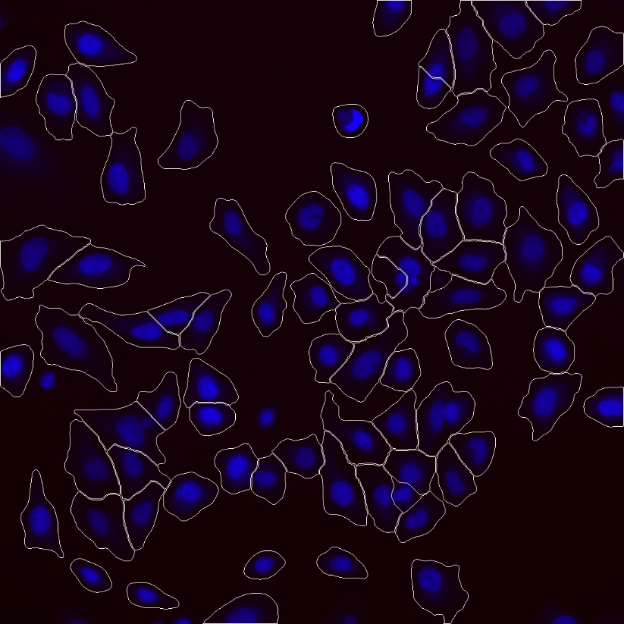

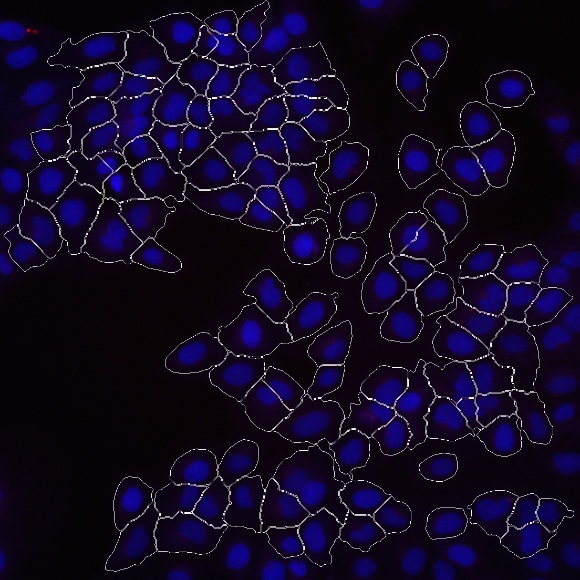


ALG2KD -NT

ALG2KD -LLOMe

SCR-LLOMe

SCR-NT


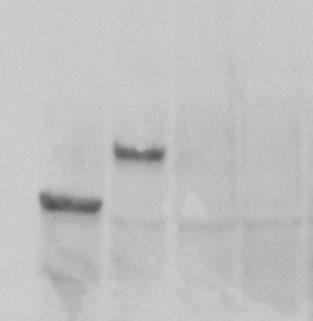

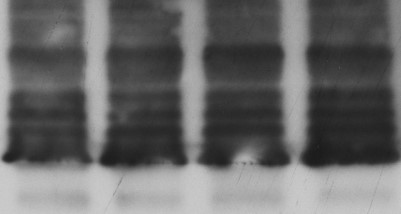

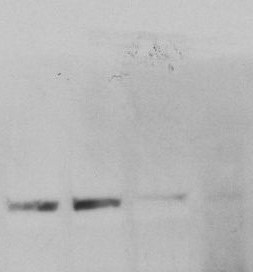

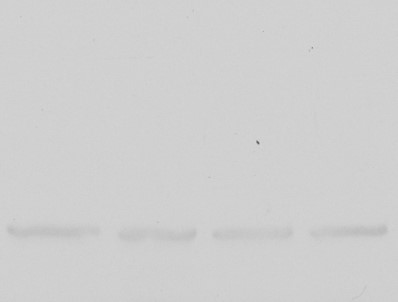


KDa

50

37

75

LLOMe: - + - +

ALG2KD

SCR

KDa

50

37

75

b-actin

KDa

50

37

75

ALG2

eIF2a

KDa

50

37

75

P-eIF2a

Supplement: Supplementary file 7 — Source data Fig. 4 [file 44318_2024_292_MOESM7_ESM.zip › Figure 4/4F/README.docx]

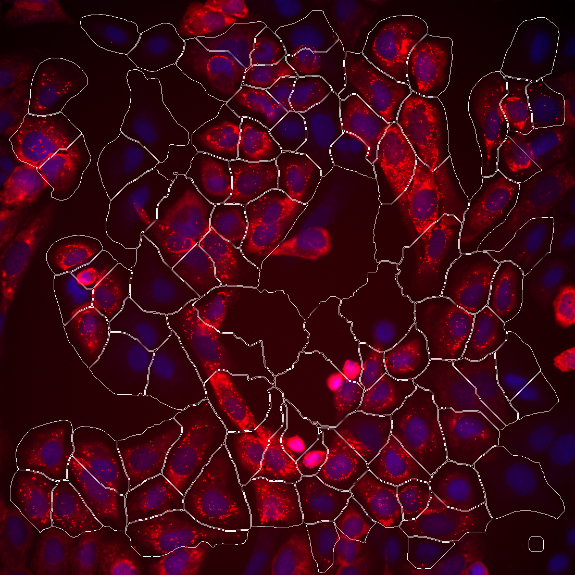

Supplement: Supplementary file 7 — Source data Fig. 4 [file 44318_2024_292_MOESM7_ESM.zip › Figure 4/4F/SCR-LLOMe.tif]

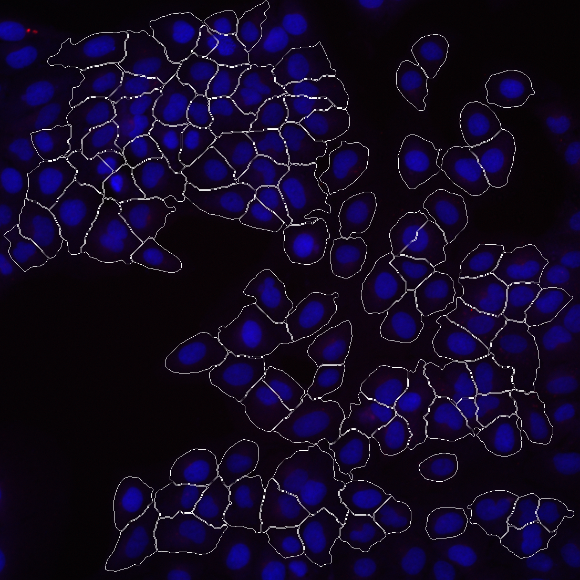

Supplement: Supplementary file 7 — Source data Fig. 4 [file 44318_2024_292_MOESM7_ESM.zip › Figure 4/4F/SCR-NT.tif]

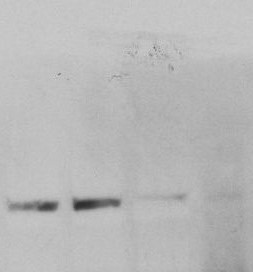

Supplement: Supplementary file 7 — Source data Fig. 4 [file 44318_2024_292_MOESM7_ESM.zip › Figure 4/4F/WB-ALG2.jpg]

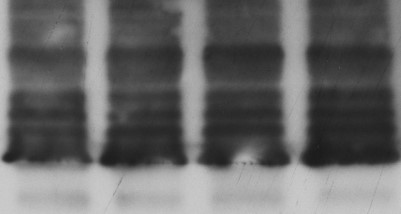

Supplement: Supplementary file 7 — Source data Fig. 4 [file 44318_2024_292_MOESM7_ESM.zip › Figure 4/4F/WB-b-actin.jpg]

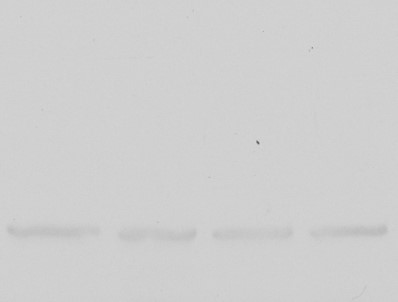

Supplement: Supplementary file 7 — Source data Fig. 4 [file 44318_2024_292_MOESM7_ESM.zip › Figure 4/4F/WB-eIF2a.jpg]

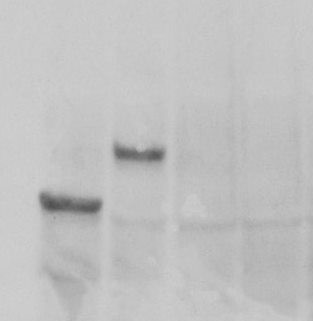

Supplement: Supplementary file 7 — Source data Fig. 4 [file 44318_2024_292_MOESM7_ESM.zip › Figure 4/4F/WB-P-eIF2a.jpg]

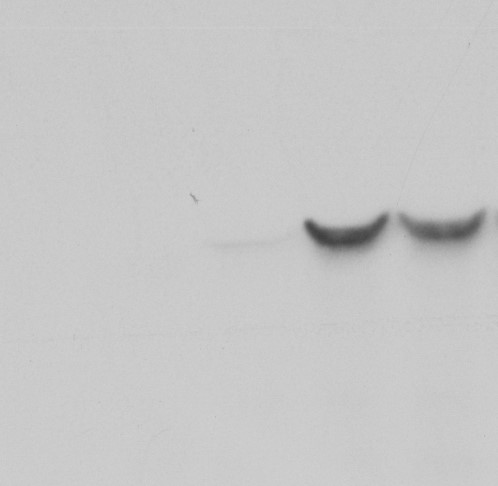

Supplement: Supplementary file 8 — Source data Fig. 5 [file 44318_2024_292_MOESM8_ESM.zip › Figure 5/5A/INPUT-FLAG.jpg]

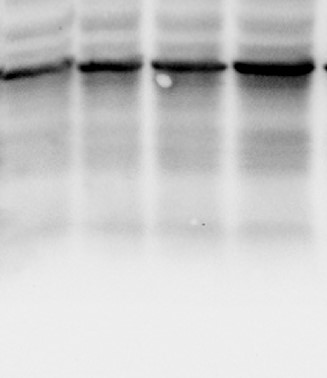

Supplement: Supplementary file 8 — Source data Fig. 5 [file 44318_2024_292_MOESM8_ESM.zip › Figure 5/5A/INPUT-PACT.jpg]

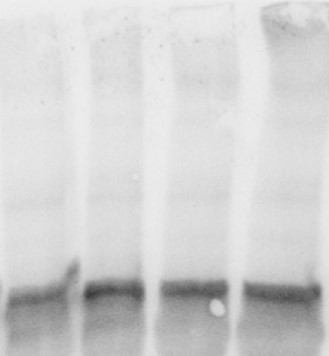

Supplement: Supplementary file 8 — Source data Fig. 5 [file 44318_2024_292_MOESM8_ESM.zip › Figure 5/5A/INPUT-PKR.jpg]

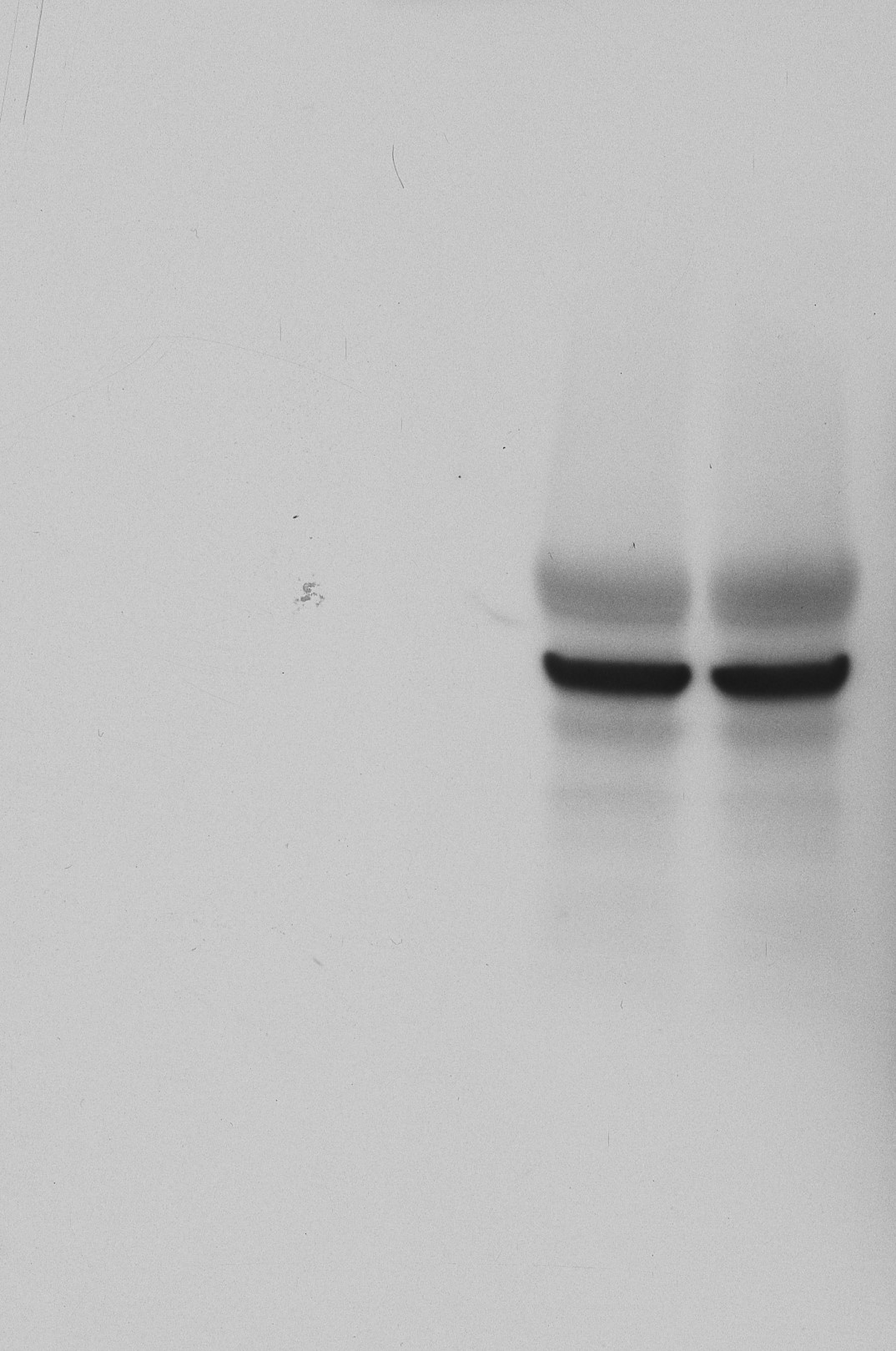

Supplement: Supplementary file 8 — Source data Fig. 5 [file 44318_2024_292_MOESM8_ESM.zip › Figure 5/5A/IP-FLAG.jpg]

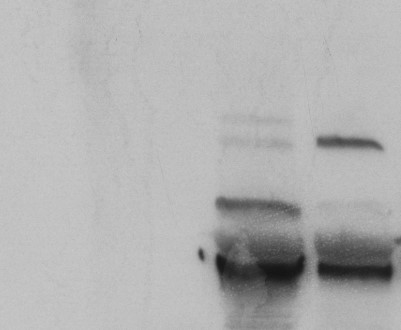

Supplement: Supplementary file 8 — Source data Fig. 5 [file 44318_2024_292_MOESM8_ESM.zip › Figure 5/5A/IP-PACT.jpg]

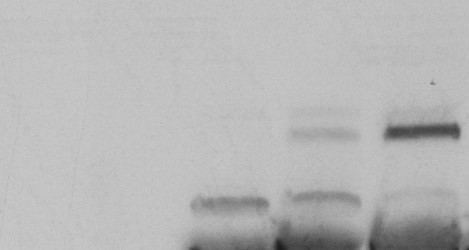

Supplement: Supplementary file 8 — Source data Fig. 5 [file 44318_2024_292_MOESM8_ESM.zip › Figure 5/5A/IP-PKR.jpg]

LLOMe: - + - +

IP:


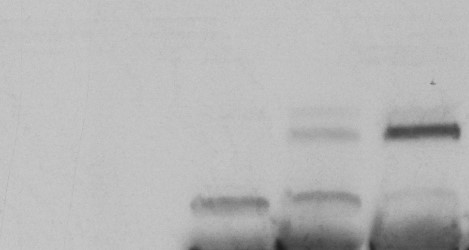


KDa

50

37

75

PKR

KDa

50

37

75


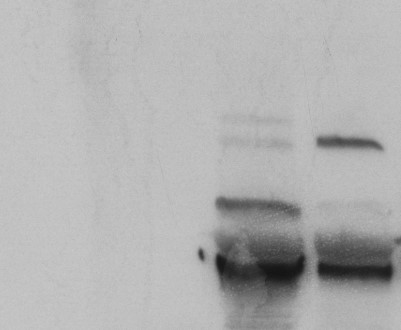


PACT


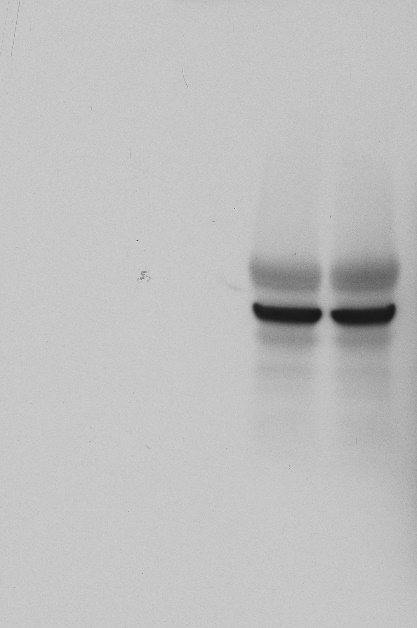


KDa

50

37

75

150

FLAG


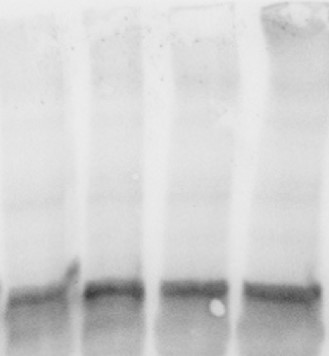


INPUT:

KDa

50

75

PKR


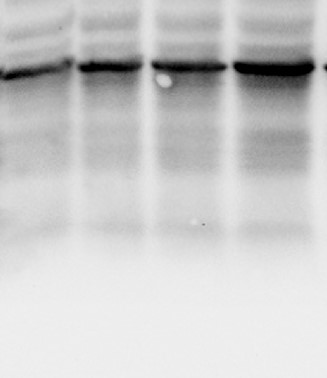


KDa

30

15

55

PACT

KDa

150

100

200


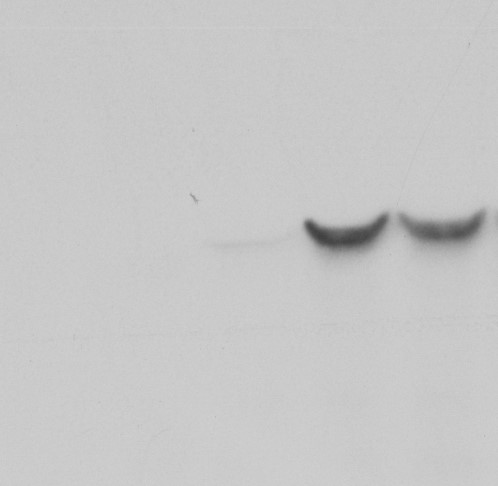


FLAG

Supplement: Supplementary file 8 — Source data Fig. 5 [file 44318_2024_292_MOESM8_ESM.zip › Figure 5/5A/README.docx]

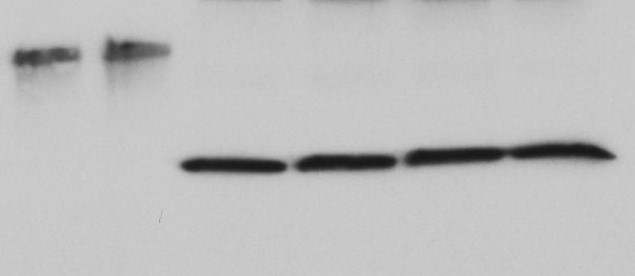

Supplement: Supplementary file 8 — Source data Fig. 5 [file 44318_2024_292_MOESM8_ESM.zip › Figure 5/5C/INPUT-FLAG.jpg]

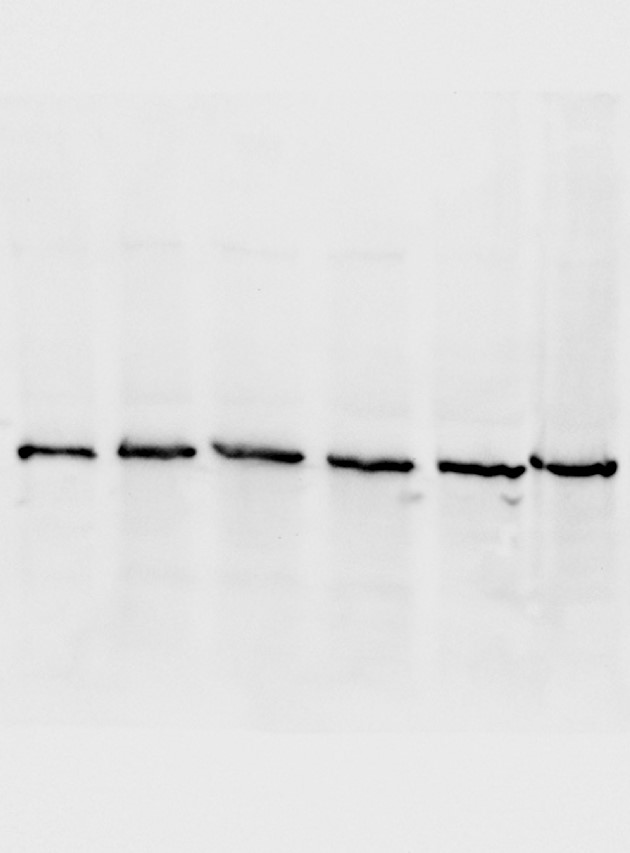

Supplement: Supplementary file 8 — Source data Fig. 5 [file 44318_2024_292_MOESM8_ESM.zip › Figure 5/5C/INPUT-PACT.jpg]

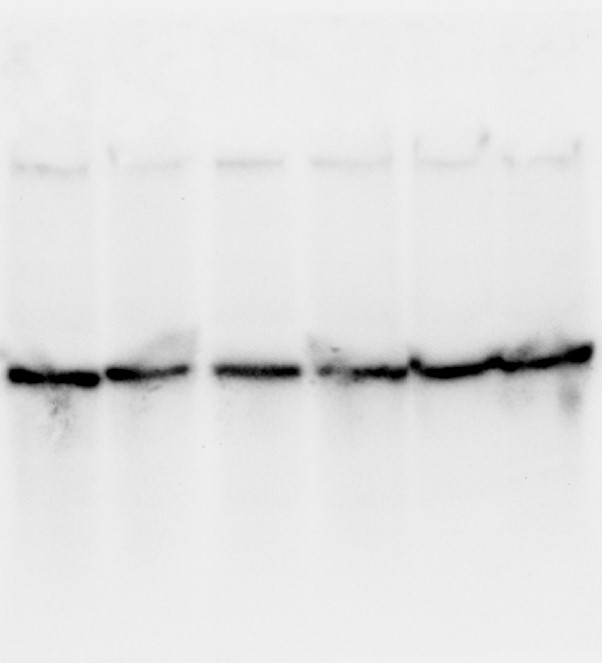

Supplement: Supplementary file 8 — Source data Fig. 5 [file 44318_2024_292_MOESM8_ESM.zip › Figure 5/5C/INPUT-PKR.jpg]

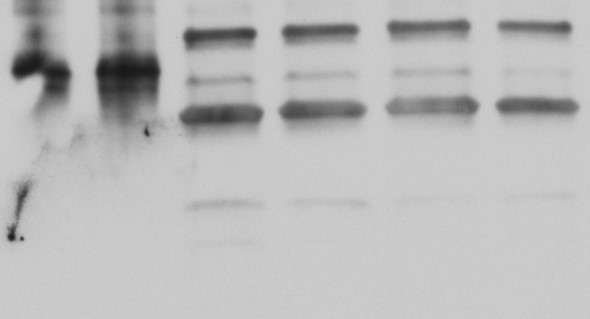

Supplement: Supplementary file 8 — Source data Fig. 5 [file 44318_2024_292_MOESM8_ESM.zip › Figure 5/5C/IP-FLAG.jpg]

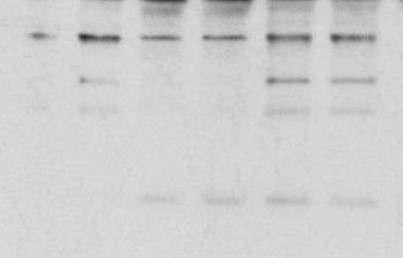

Supplement: Supplementary file 8 — Source data Fig. 5 [file 44318_2024_292_MOESM8_ESM.zip › Figure 5/5C/IP-PKR-PACT.jpg]

IP:

LLOME: - + - + - +

KDa

50

37

75


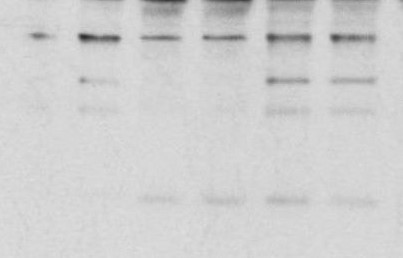


PACT

Myc-PKR


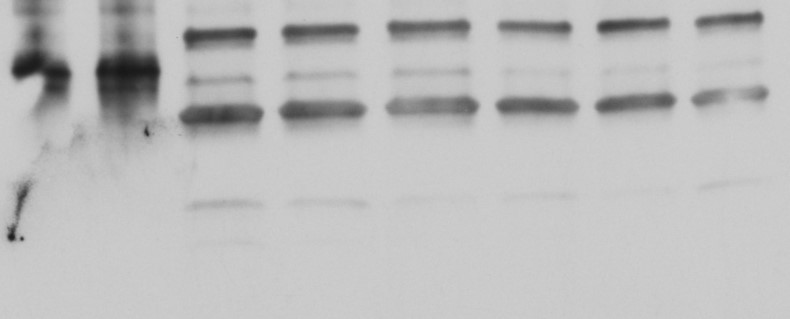


KDa

50

37

75

FLAG


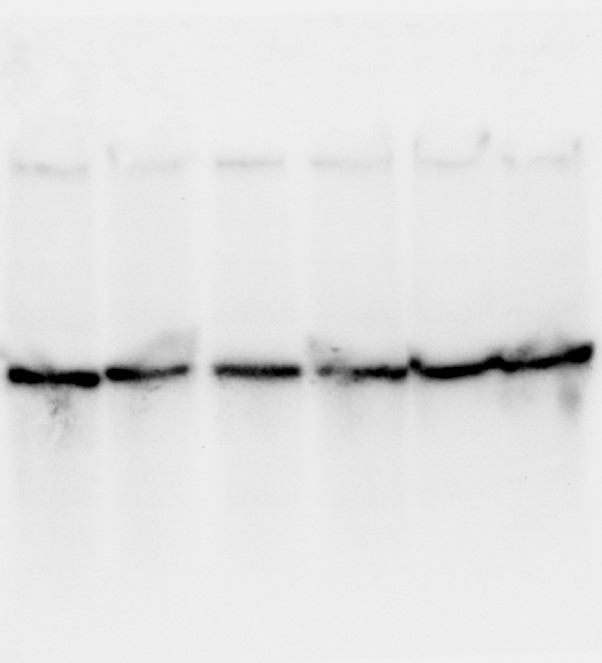


INPUT:

KDa

50

37

75

Myc-PKR


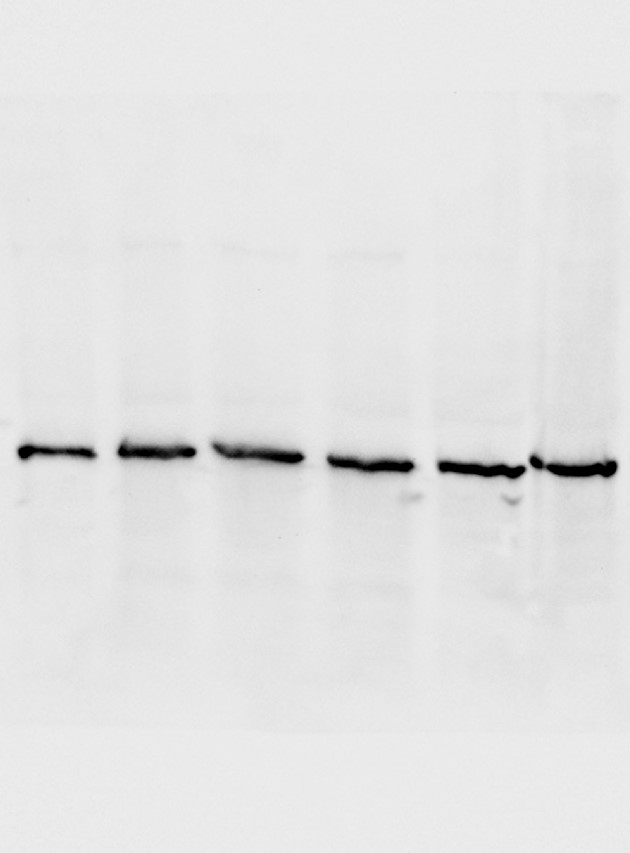


KDa

50

37

75

PACT


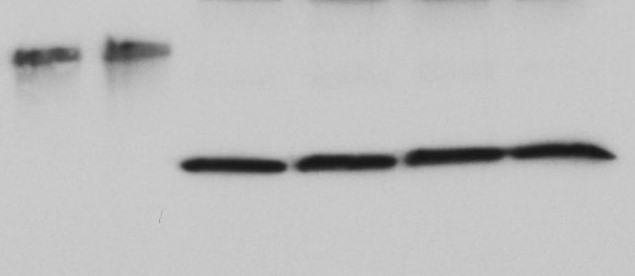


KDa

50

37

75

FLAG

Supplement: Supplementary file 8 — Source data Fig. 5 [file 44318_2024_292_MOESM8_ESM.zip › Figure 5/5C/README.docx]

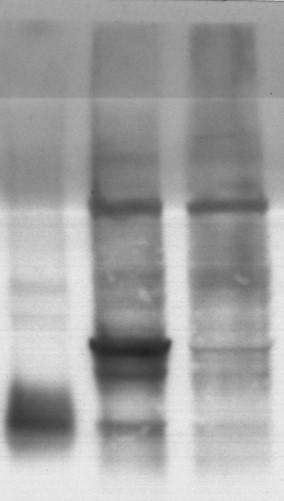

Supplement: Supplementary file 8 — Source data Fig. 5 [file 44318_2024_292_MOESM8_ESM.zip › Figure 5/5D/GST.jpg]

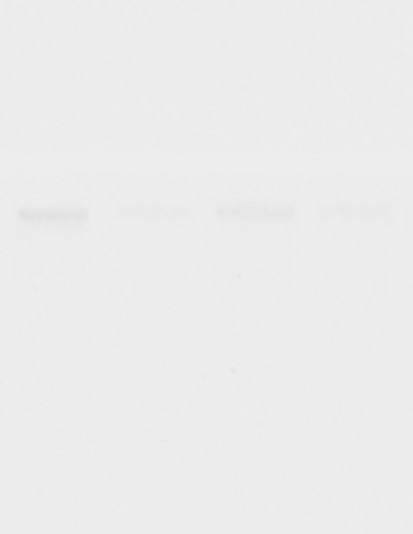

Supplement: Supplementary file 8 — Source data Fig. 5 [file 44318_2024_292_MOESM8_ESM.zip › Figure 5/5D/HIS-PACT.jpg]

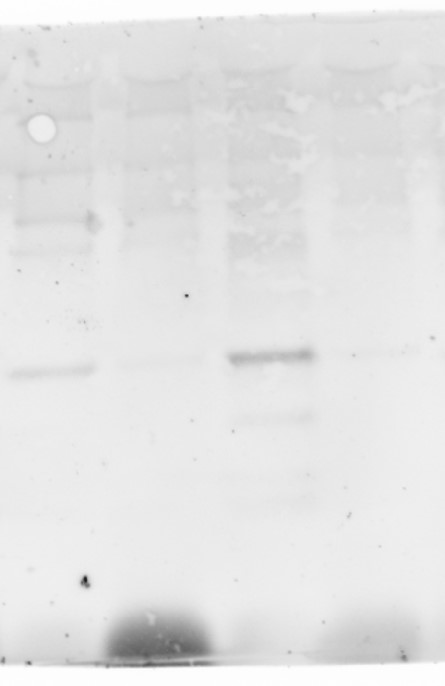

Supplement: Supplementary file 8 — Source data Fig. 5 [file 44318_2024_292_MOESM8_ESM.zip › Figure 5/5D/HIS-PKR.jpg]

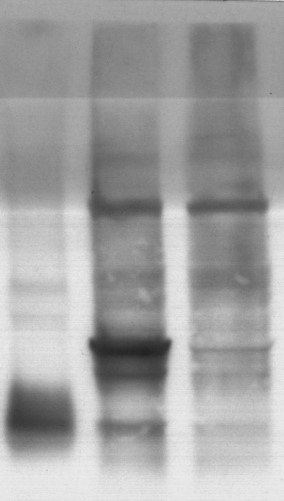

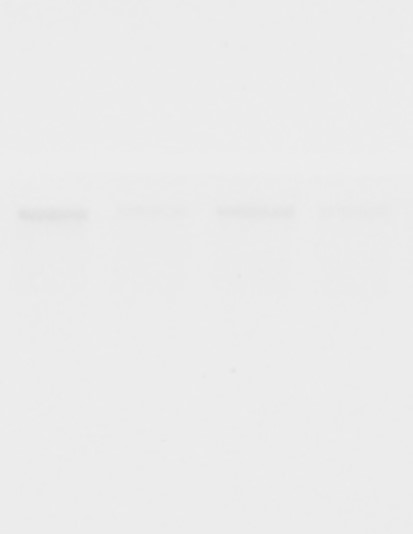

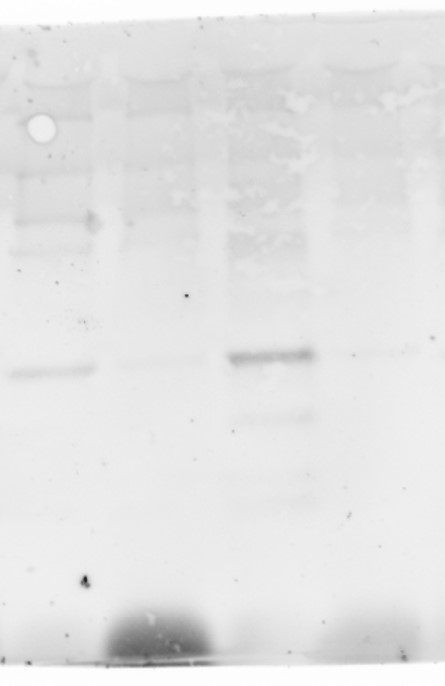


HIS-PACT

50

37

KDa

100

250

ALIX

GST

ALIX&LG2

GST

GST

INPUT

KDa

50

37

75

KDa

50

37

75

GST

HIS-PKR

Supplement: Supplementary file 8 — Source data Fig. 5 [file 44318_2024_292_MOESM8_ESM.zip › Figure 5/5D/README.docx]

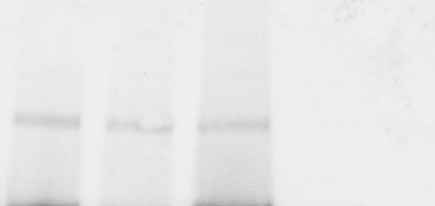

Supplement: Supplementary file 8 — Source data Fig. 5 [file 44318_2024_292_MOESM8_ESM.zip › Figure 5/5E/INPUT-ALIX.jpg]

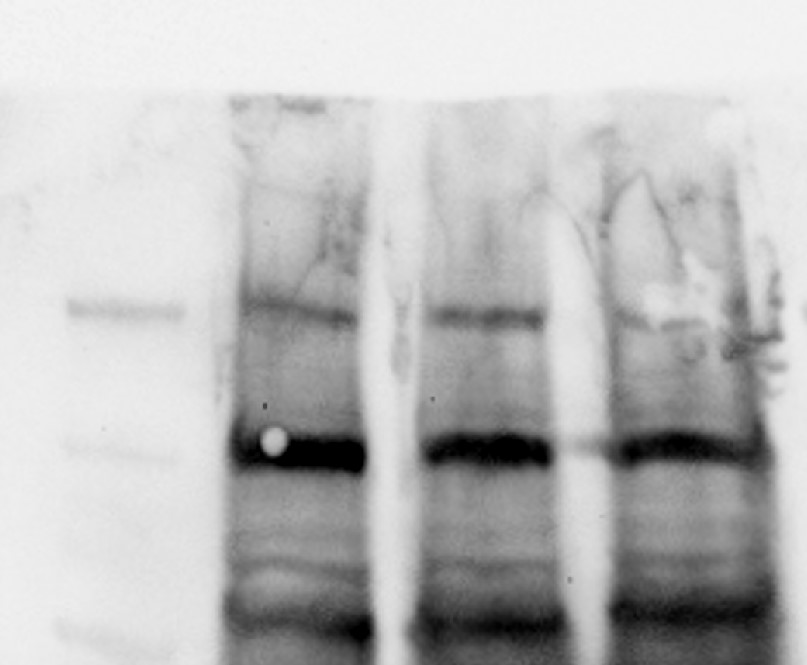

Supplement: Supplementary file 8 — Source data Fig. 5 [file 44318_2024_292_MOESM8_ESM.zip › Figure 5/5E/INPUT-FLAG.jpg]

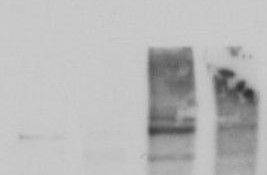

Supplement: Supplementary file 8 — Source data Fig. 5 [file 44318_2024_292_MOESM8_ESM.zip › Figure 5/5E/INPUT-P-PKR.jpg]

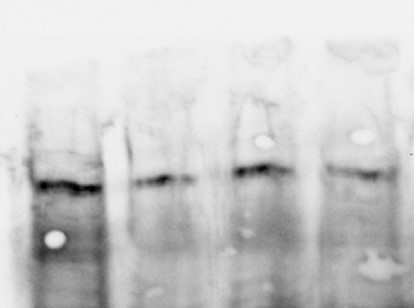

Supplement: Supplementary file 8 — Source data Fig. 5 [file 44318_2024_292_MOESM8_ESM.zip › Figure 5/5E/INPUT-PACT.jpg]

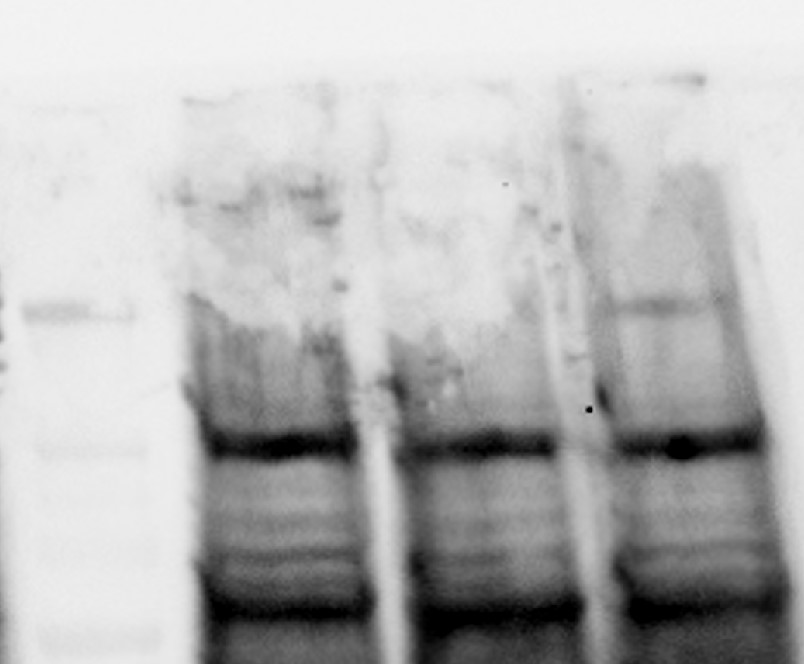

Supplement: Supplementary file 8 — Source data Fig. 5 [file 44318_2024_292_MOESM8_ESM.zip › Figure 5/5E/IP-FLAG.jpg]

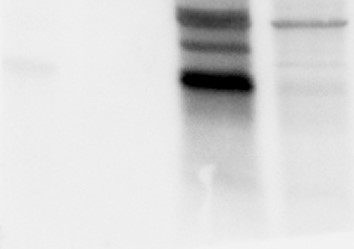

Supplement: Supplementary file 8 — Source data Fig. 5 [file 44318_2024_292_MOESM8_ESM.zip › Figure 5/5E/IP-PACT.jpg]

IP:

LLOMe: - - + +


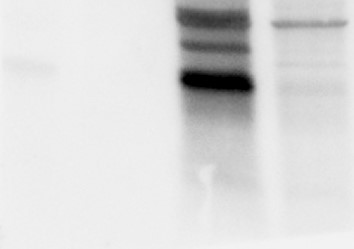


KDa

50

25

PACT


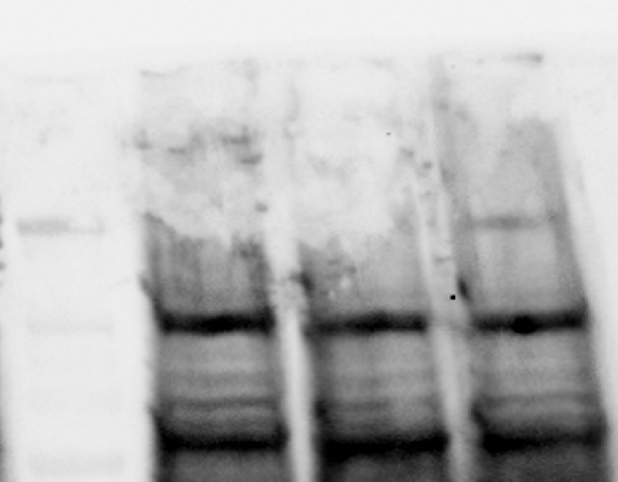


KDa

50

37

75

FLAG

INPUT:


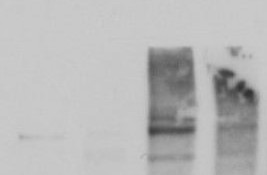


KDa

50

37

75

P-PKR


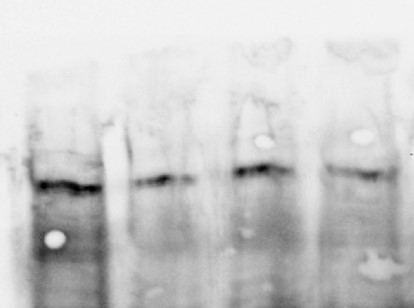


KDa

50

37

75

PACT


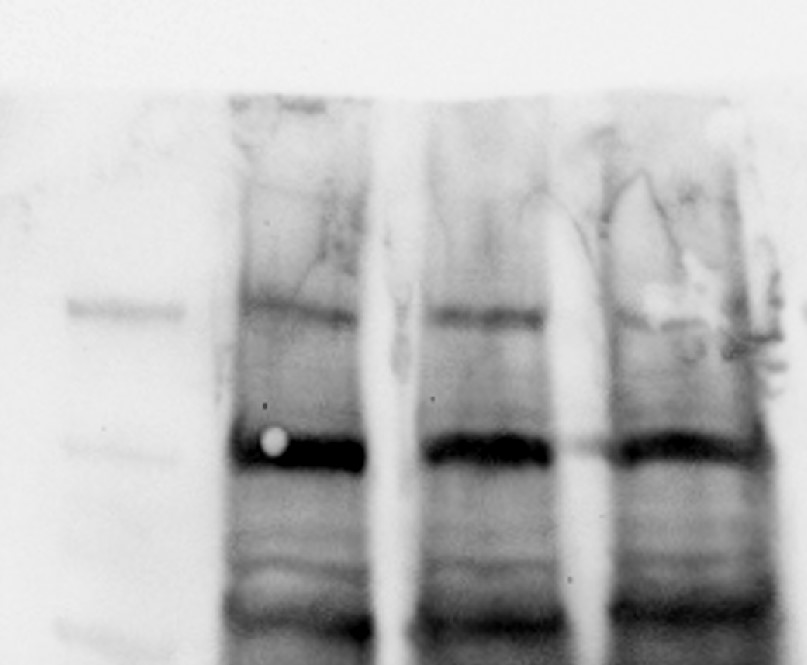


KDa

50

37

75

FLAG


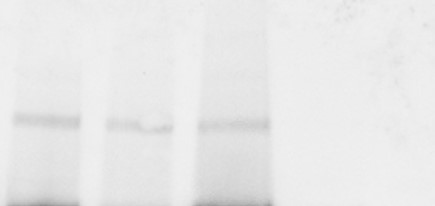


KDa

95

72

150

ALIX

Supplement: Supplementary file 8 — Source data Fig. 5 [file 44318_2024_292_MOESM8_ESM.zip › Figure 5/5E/README.docx]

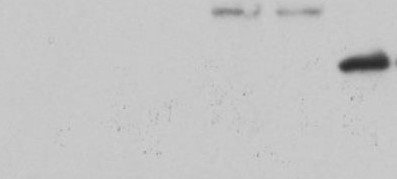

Supplement: Supplementary file 8 — Source data Fig. 5 [file 44318_2024_292_MOESM8_ESM.zip › Figure 5/5F/INPUT-FLAG.jpg]

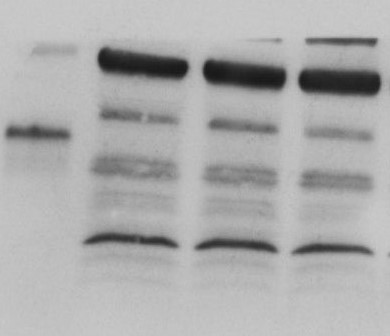

Supplement: Supplementary file 8 — Source data Fig. 5 [file 44318_2024_292_MOESM8_ESM.zip › Figure 5/5F/INPUT-GFP.jpg]

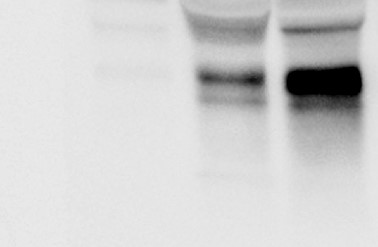

Supplement: Supplementary file 8 — Source data Fig. 5 [file 44318_2024_292_MOESM8_ESM.zip › Figure 5/5F/INPUT-P-PKR.jpg]

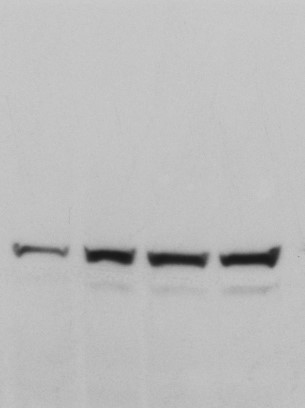

Supplement: Supplementary file 8 — Source data Fig. 5 [file 44318_2024_292_MOESM8_ESM.zip › Figure 5/5F/INPUT-PKR.jpg]

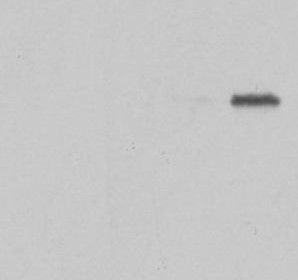

Supplement: Supplementary file 8 — Source data Fig. 5 [file 44318_2024_292_MOESM8_ESM.zip › Figure 5/5F/IP-FLAG.jpg]

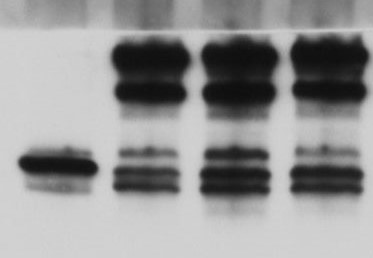

Supplement: Supplementary file 8 — Source data Fig. 5 [file 44318_2024_292_MOESM8_ESM.zip › Figure 5/5F/IP-GFP.jpg]

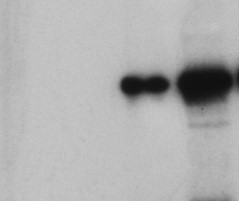

Supplement: Supplementary file 8 — Source data Fig. 5 [file 44318_2024_292_MOESM8_ESM.zip › Figure 5/5F/IP-PKR.jpg]

LLOMe: - - + +


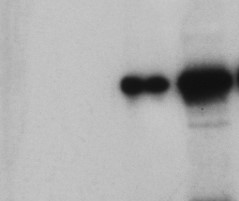


IP:

KDa

50

37

75

PKR


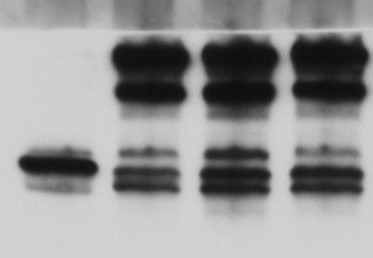


KDa

50

37

75

GFP


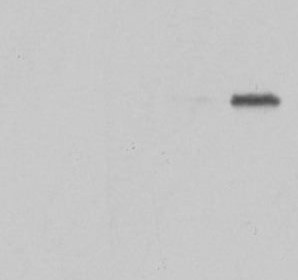


KDa

95

72

150

FLAG

INPUT:

KDa

95

72

150


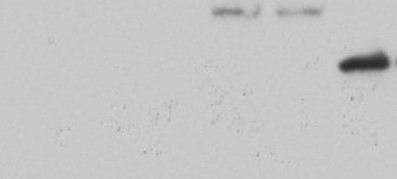


FLAG

KDa

50

37

75


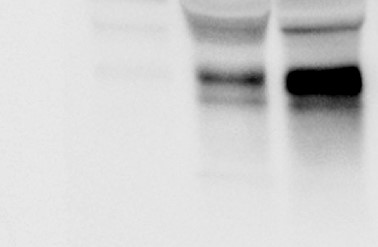


P-PKR


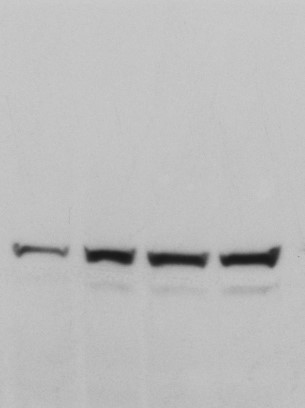


KDa

50

37

75

PKR

KDa

50

37

75


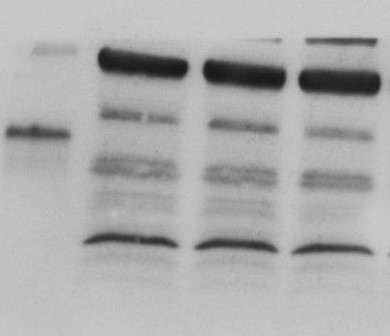


GFP

Supplement: Supplementary file 8 — Source data Fig. 5 [file 44318_2024_292_MOESM8_ESM.zip › Figure 5/5F/README.docx]

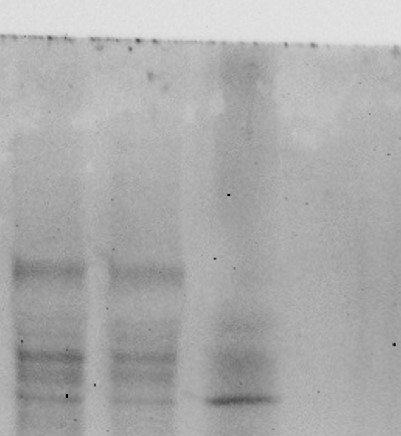

Supplement: Supplementary file 8 — Source data Fig. 5 [file 44318_2024_292_MOESM8_ESM.zip › Figure 5/5G/INPUT-ALIX.jpg]

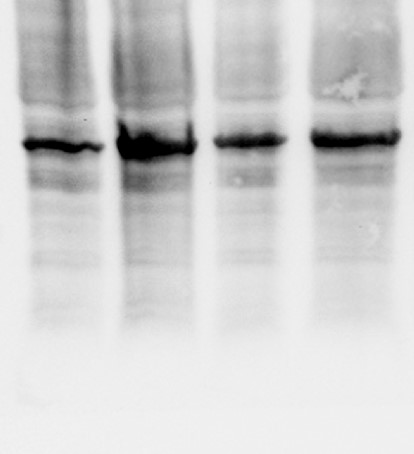

Supplement: Supplementary file 8 — Source data Fig. 5 [file 44318_2024_292_MOESM8_ESM.zip › Figure 5/5G/INPUT-LAMP2.jpg]

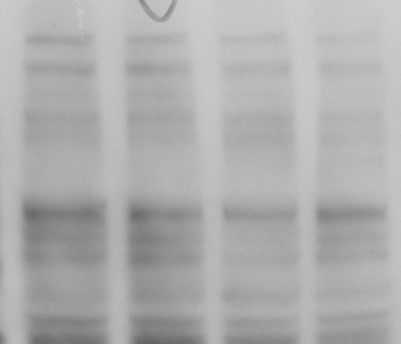

Supplement: Supplementary file 8 — Source data Fig. 5 [file 44318_2024_292_MOESM8_ESM.zip › Figure 5/5G/INPUT-PACT.jpg]

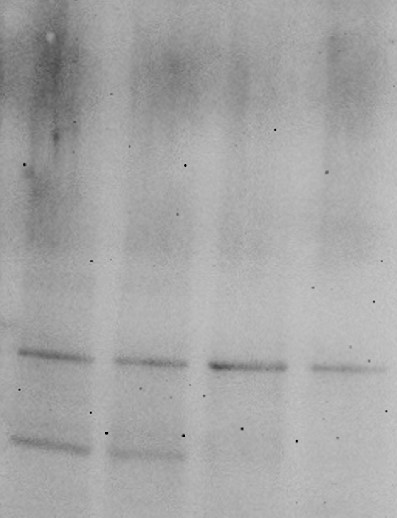

Supplement: Supplementary file 8 — Source data Fig. 5 [file 44318_2024_292_MOESM8_ESM.zip › Figure 5/5G/INPUT-PKR.jpg]

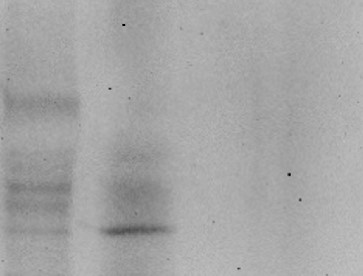

Supplement: Supplementary file 8 — Source data Fig. 5 [file 44318_2024_292_MOESM8_ESM.zip › Figure 5/5G/LYSOIP-ALIX.jpg]

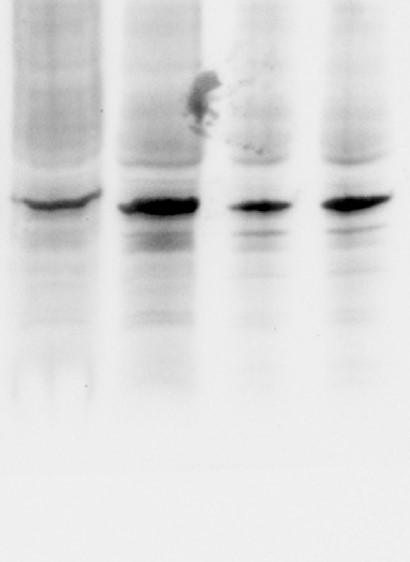

Supplement: Supplementary file 8 — Source data Fig. 5 [file 44318_2024_292_MOESM8_ESM.zip › Figure 5/5G/LYSOIP-LAMP2.jpg]

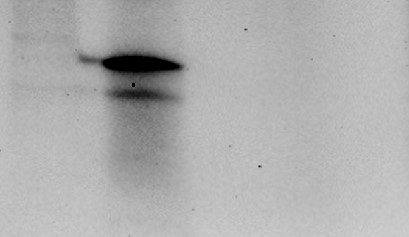

Supplement: Supplementary file 8 — Source data Fig. 5 [file 44318_2024_292_MOESM8_ESM.zip › Figure 5/5G/LYSOIP-P-PKR.jpg]

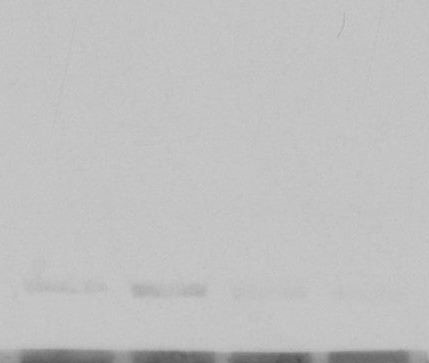

Supplement: Supplementary file 8 — Source data Fig. 5 [file 44318_2024_292_MOESM8_ESM.zip › Figure 5/5G/LYSOIP-PACT.jpg]

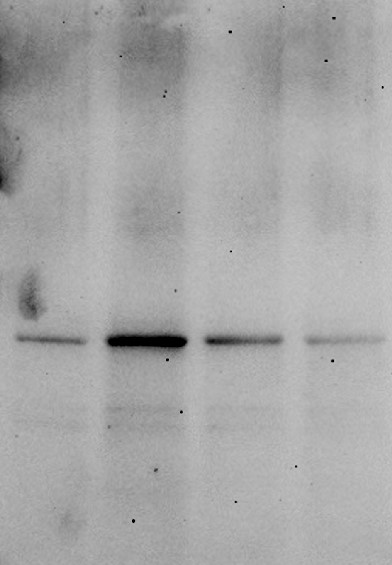

Supplement: Supplementary file 8 — Source data Fig. 5 [file 44318_2024_292_MOESM8_ESM.zip › Figure 5/5G/LYSOIP-PKR.jpg]

IP:

KDa

50

37

75

LLOMe: - - + +


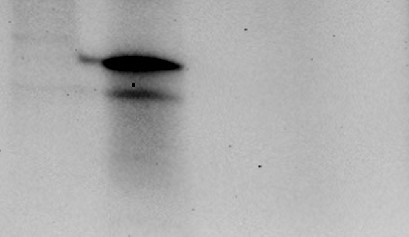


P-PKR


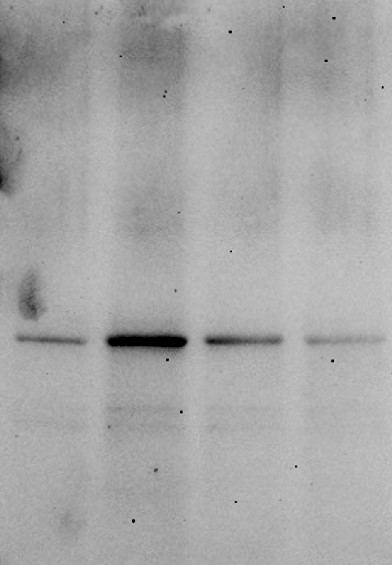


KDa

50

37

75

PKR


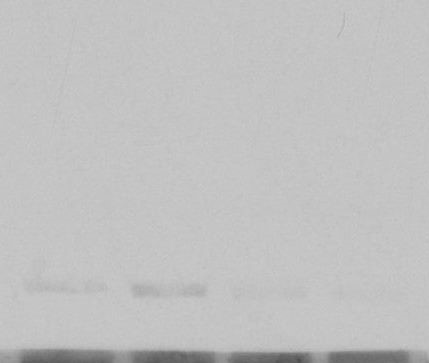


KDa

50

37

75

PACT


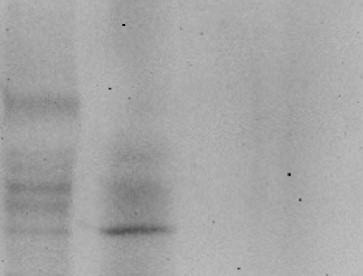


KDa

95

150

ALIX

KDa

95

150


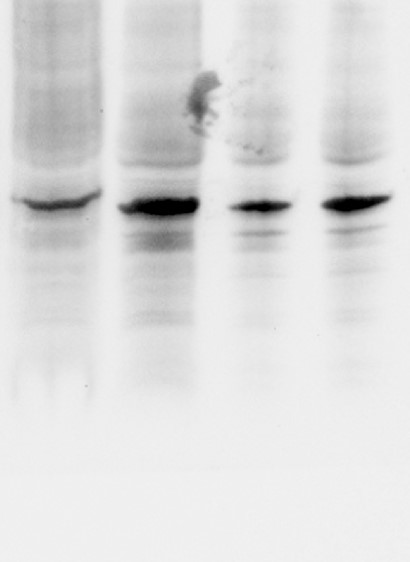


LAMP2

INPUT:


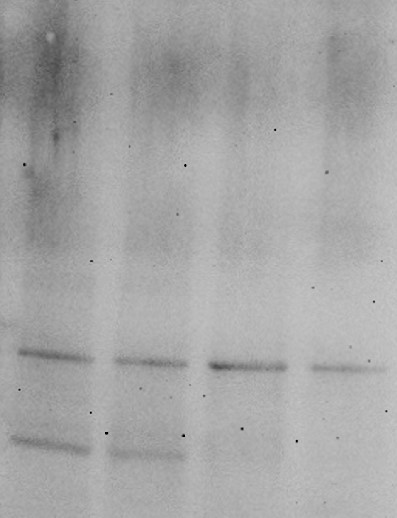


PKR

KDa

50

37

75


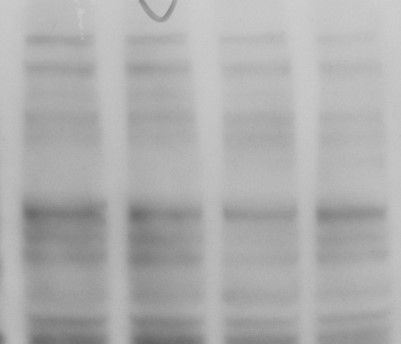

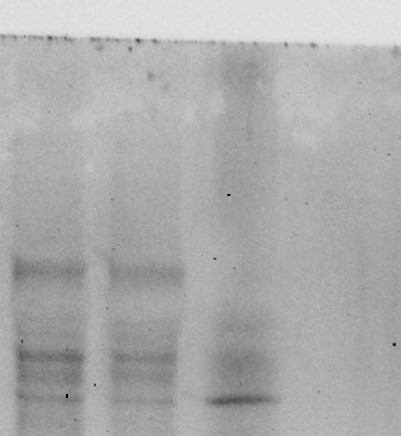

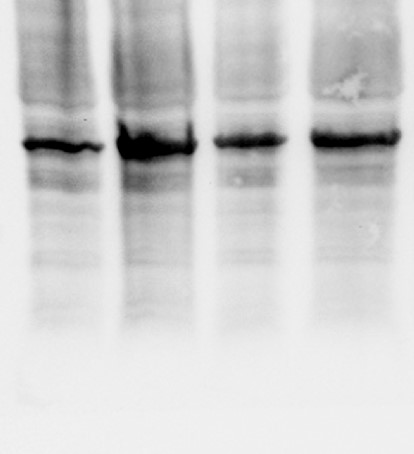


LAMP2

KDa

95

150

KDa

95

150

ALIX

KDa

50

37

75

PACT

Supplement: Supplementary file 8 — Source data Fig. 5 [file 44318_2024_292_MOESM8_ESM.zip › Figure 5/5G/README.docx]

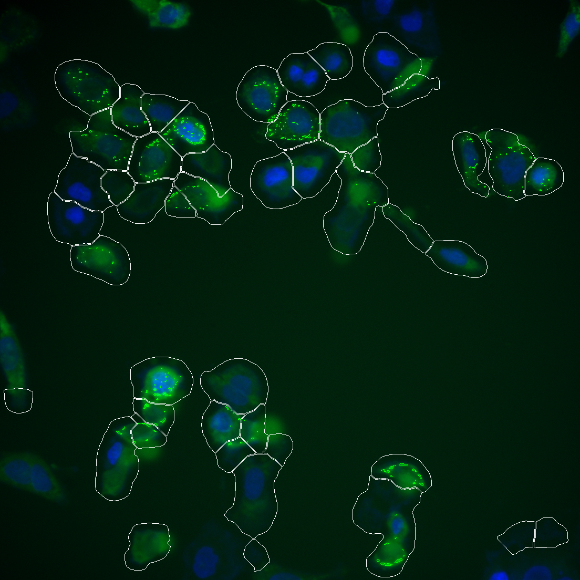

Supplement: Supplementary file 9 — Source data Fig. 6 [file 44318_2024_292_MOESM9_ESM.zip › Figure 6/6A/GAL3KD-LLOMe.tif]

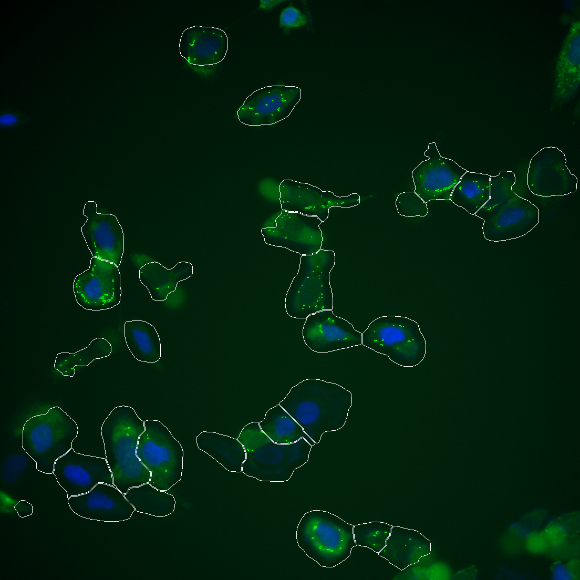

Supplement: Supplementary file 9 — Source data Fig. 6 [file 44318_2024_292_MOESM9_ESM.zip › Figure 6/6A/GAL3KD-NT.tif]

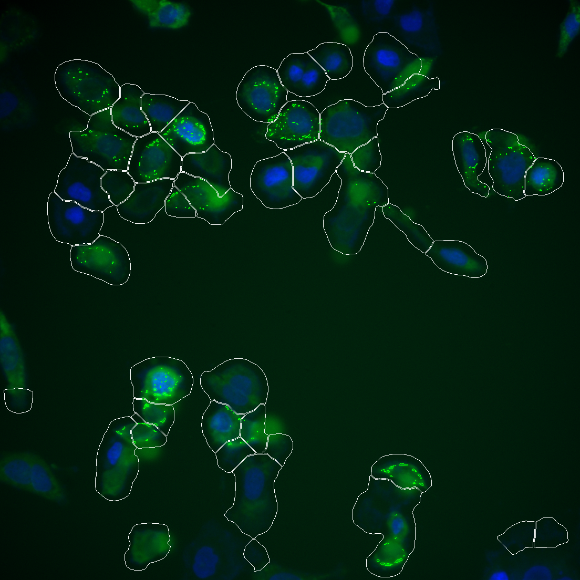

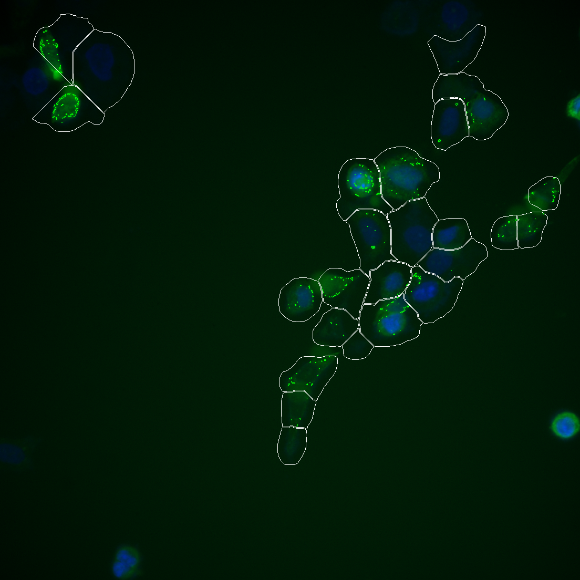

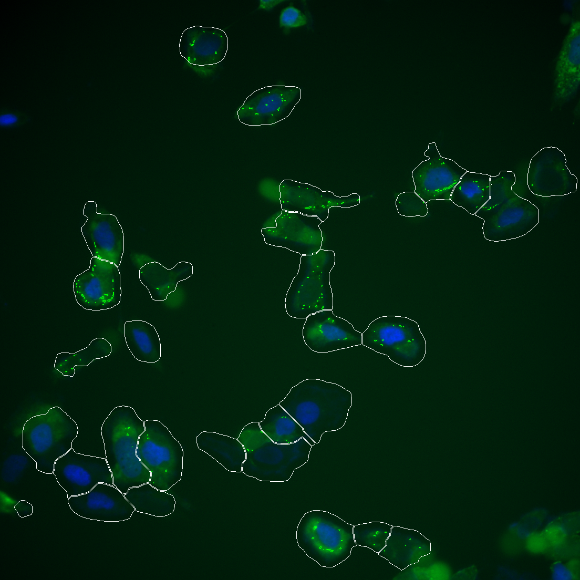

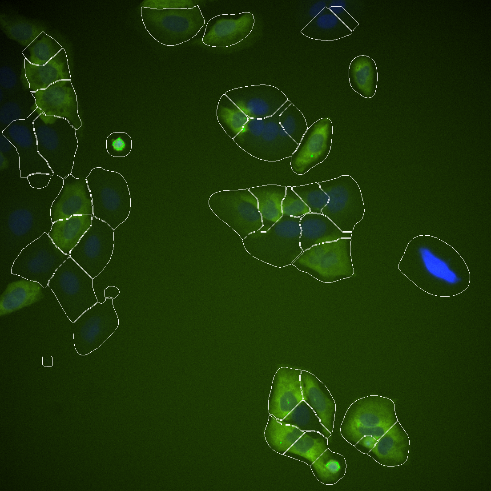


SCR-NT

GAL3KD -NT

GAL3KD -LLOMe

SCR-LLOMe

Supplement: Supplementary file 9 — Source data Fig. 6 [file 44318_2024_292_MOESM9_ESM.zip › Figure 6/6A/README.docx]

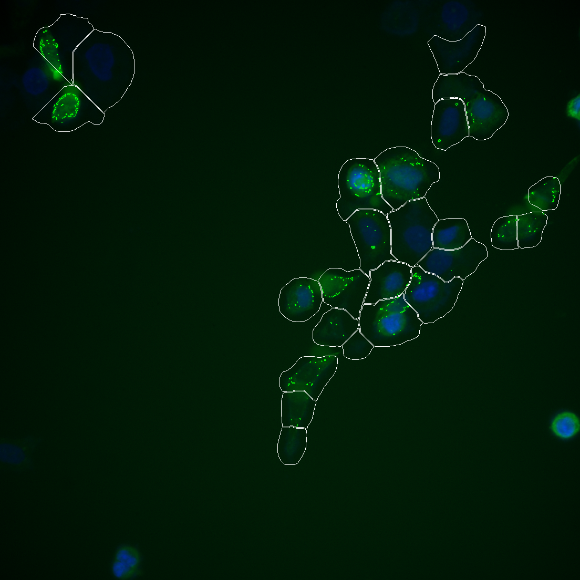

Supplement: Supplementary file 9 — Source data Fig. 6 [file 44318_2024_292_MOESM9_ESM.zip › Figure 6/6A/SCR-LLOMe.tif]

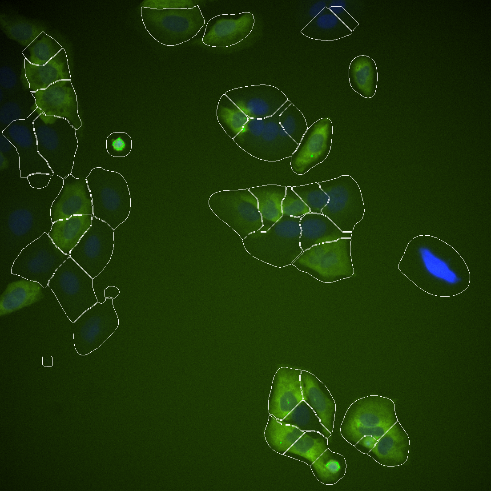

Supplement: Supplementary file 9 — Source data Fig. 6 [file 44318_2024_292_MOESM9_ESM.zip › Figure 6/6A/SCR-NT.tif]

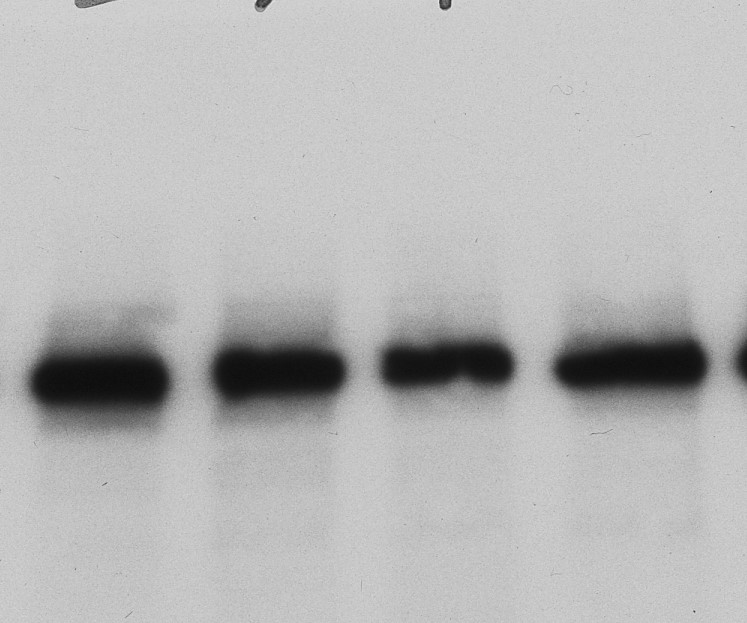

Supplement: Supplementary file 9 — Source data Fig. 6 [file 44318_2024_292_MOESM9_ESM.zip › Figure 6/6B/b-actin.jpg]

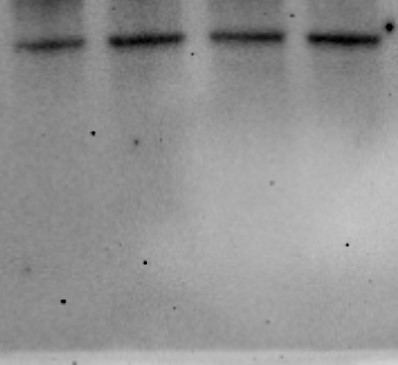

Supplement: Supplementary file 9 — Source data Fig. 6 [file 44318_2024_292_MOESM9_ESM.zip › Figure 6/6B/eIF2a.jpg]
