# Supplementary figures and images for: Calcium signaling from damaged lysosomes induces cytoprotective stress granules (part 3 of 3)
Source: EMBO J. 2024 Nov 12;43(24):6410–43. doi: 10.1038/s44318-024-00292-1 (PMC11649789; doi:10.1038/s44318-024-00292-1)

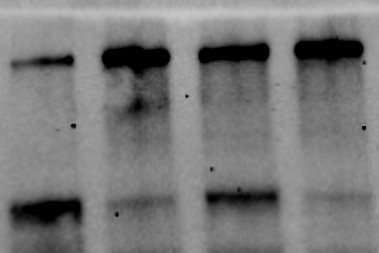

Supplement: Supplementary file 9 — Source data Fig. 6 [file 44318_2024_292_MOESM9_ESM.zip › Figure 6/6B/GAL3.jpg]

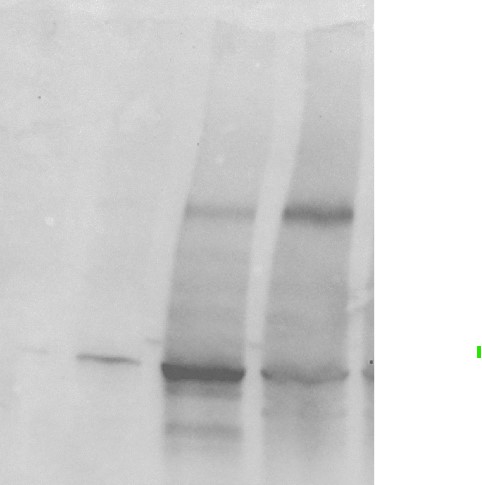

Supplement: Supplementary file 9 — Source data Fig. 6 [file 44318_2024_292_MOESM9_ESM.zip › Figure 6/6B/P-eIF2a.jpg]

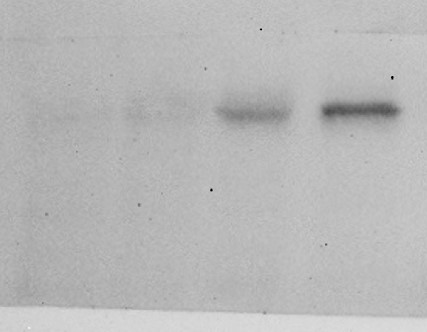

Supplement: Supplementary file 9 — Source data Fig. 6 [file 44318_2024_292_MOESM9_ESM.zip › Figure 6/6B/P-PKR.jpg]

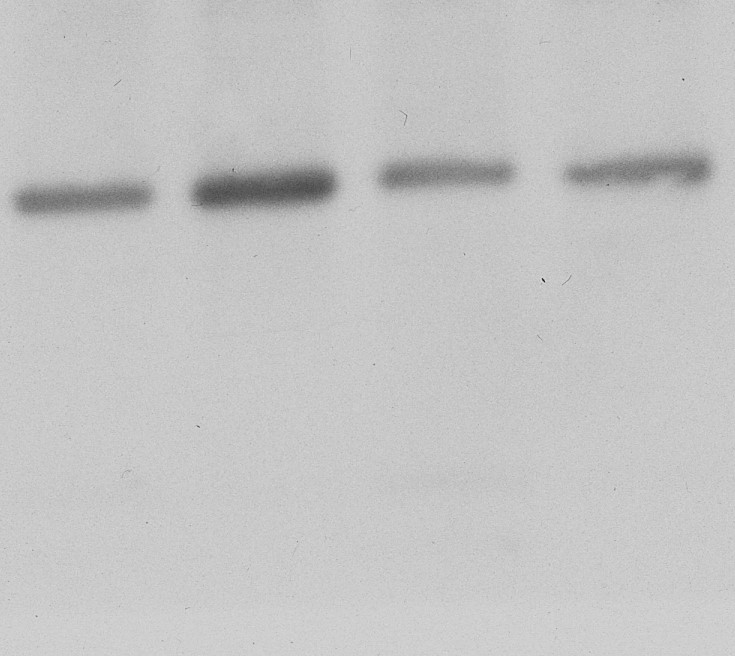

Supplement: Supplementary file 9 — Source data Fig. 6 [file 44318_2024_292_MOESM9_ESM.zip › Figure 6/6B/PKR.jpg]

LLOMe: - - + +


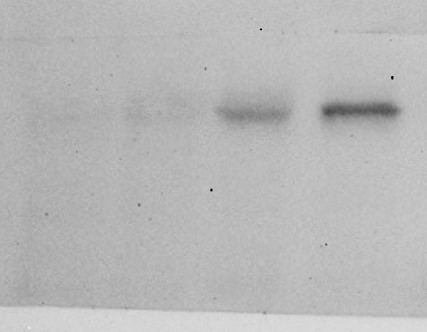


KDa

50

37

75

P-PKR


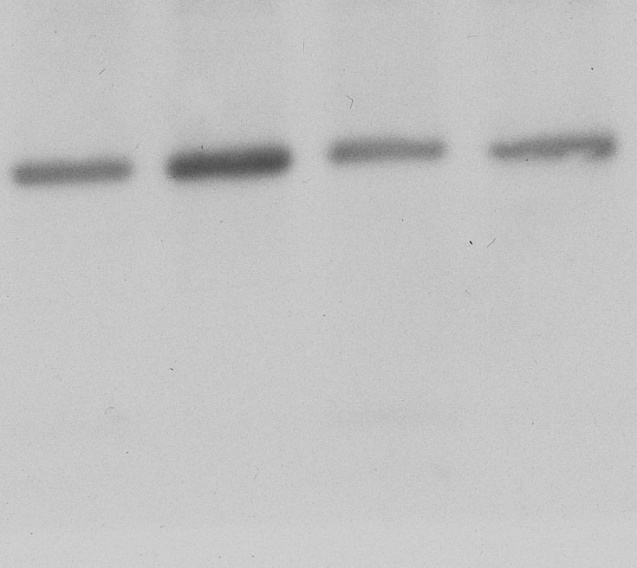


KDa

50

37

75

PKR


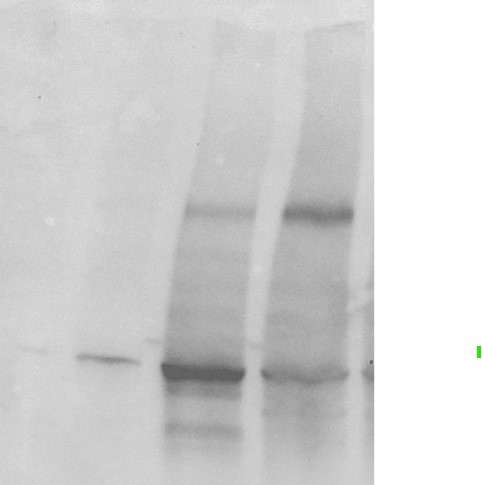


KDa

50

37

75

P-eIF2a

KDa

15

30


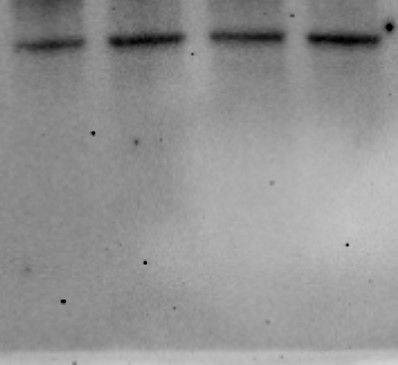


eIF2a

KDa

50

25

75


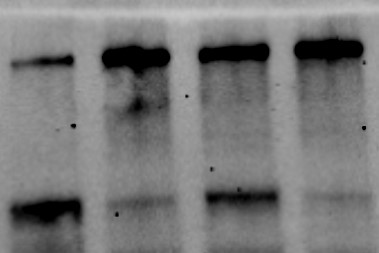


Gal3


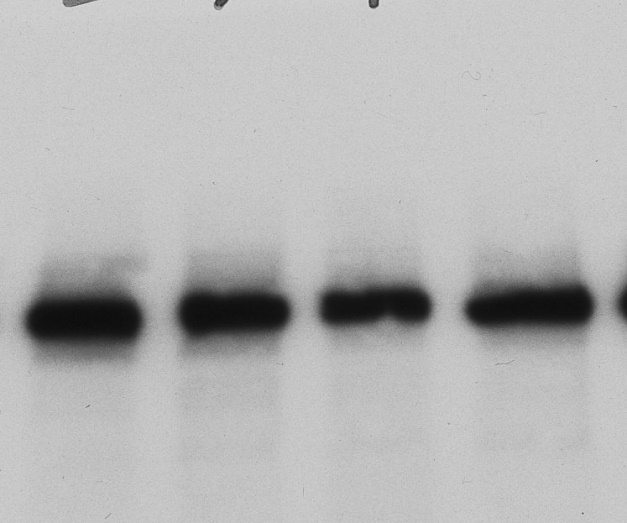


KDa

50

37

75

b-actin

KDa

95

150

KDa

95

150

Supplement: Supplementary file 9 — Source data Fig. 6 [file 44318_2024_292_MOESM9_ESM.zip › Figure 6/6B/README.docx]

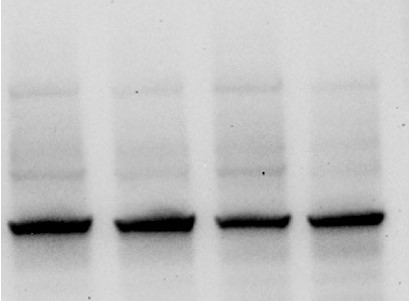

Supplement: Supplementary file 9 — Source data Fig. 6 [file 44318_2024_292_MOESM9_ESM.zip › Figure 6/6C/INPUT-ALIX-PACT.jpg]

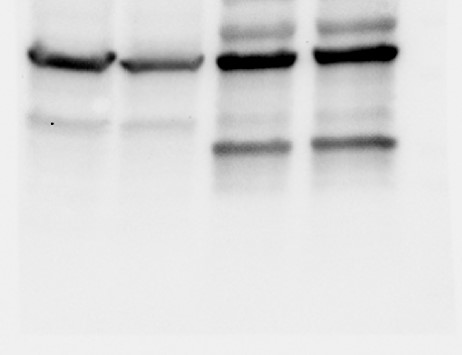

Supplement: Supplementary file 9 — Source data Fig. 6 [file 44318_2024_292_MOESM9_ESM.zip › Figure 6/6C/INPUT-PKR-FLAG.jpg]

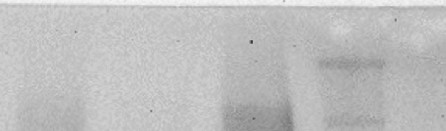

Supplement: Supplementary file 9 — Source data Fig. 6 [file 44318_2024_292_MOESM9_ESM.zip › Figure 6/6C/IP-ALIX.jpg]

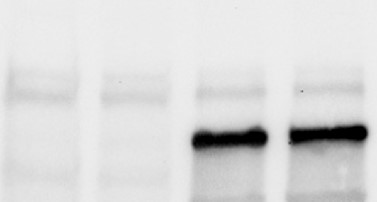

Supplement: Supplementary file 9 — Source data Fig. 6 [file 44318_2024_292_MOESM9_ESM.zip › Figure 6/6C/IP-FLAG.jpg]

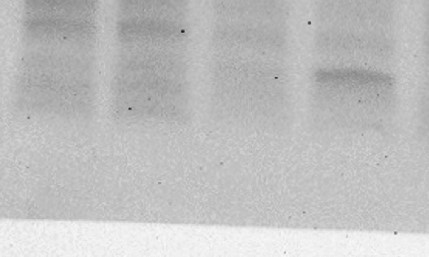

Supplement: Supplementary file 9 — Source data Fig. 6 [file 44318_2024_292_MOESM9_ESM.zip › Figure 6/6C/IP-PACT.jpg]

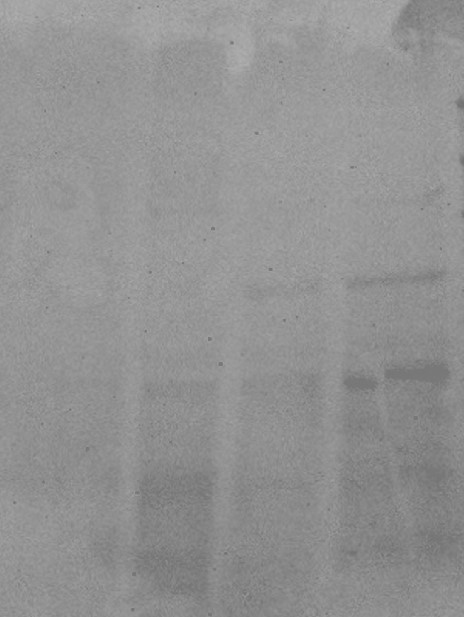

Supplement: Supplementary file 9 — Source data Fig. 6 [file 44318_2024_292_MOESM9_ESM.zip › Figure 6/6C/IP-PKR.jpg]

LLOMe: - + - +

KDa

95

150


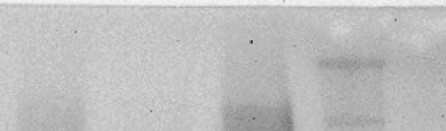


IP:

ALIX


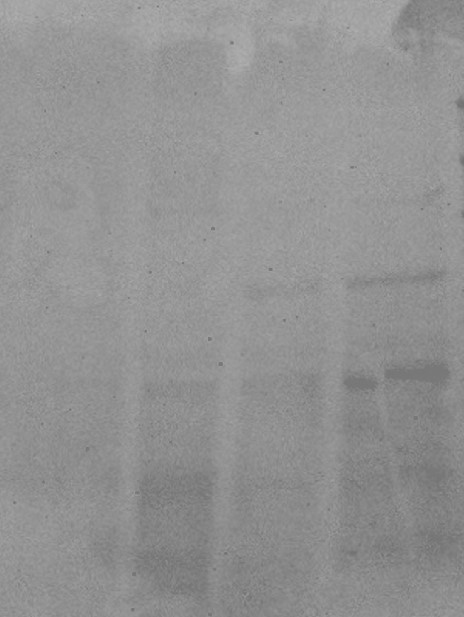


KDa

50

37

75

PKR


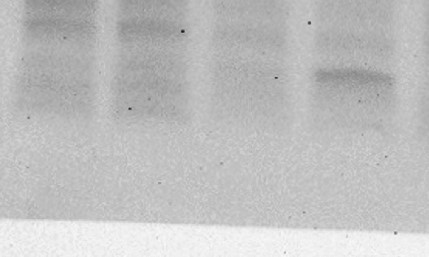


KDa

50

37

PACT


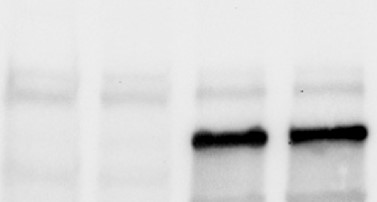


KDa

15

30

FLAG

INPUT:


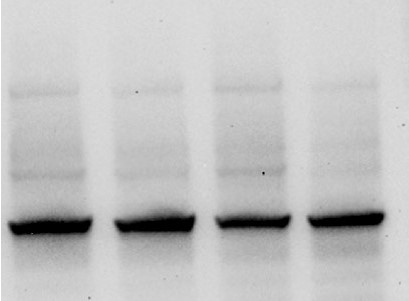


KDa

55

37

95

ALIX

PACT


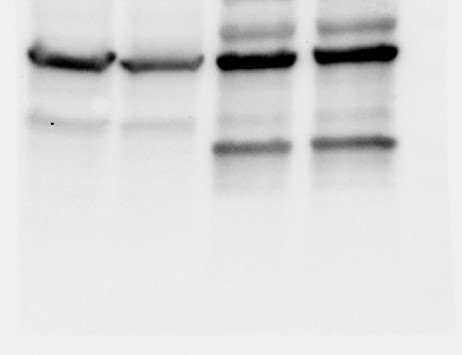


KDa

50

37

75

PKR

FLAG

KDa

95

150

KDa

95

150

Supplement: Supplementary file 9 — Source data Fig. 6 [file 44318_2024_292_MOESM9_ESM.zip › Figure 6/6C/README.docx]

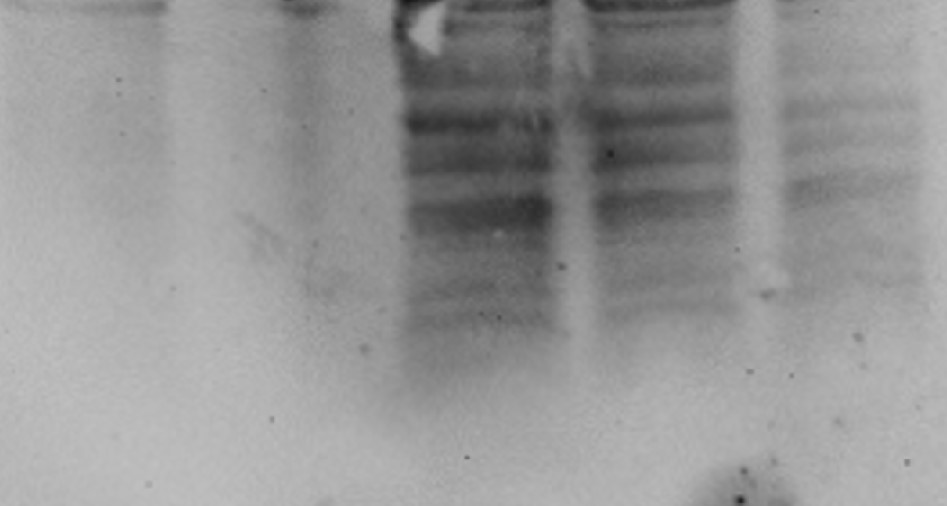

Supplement: Supplementary file 9 — Source data Fig. 6 [file 44318_2024_292_MOESM9_ESM.zip › Figure 6/6D/INPUT-FLAG.jpg]

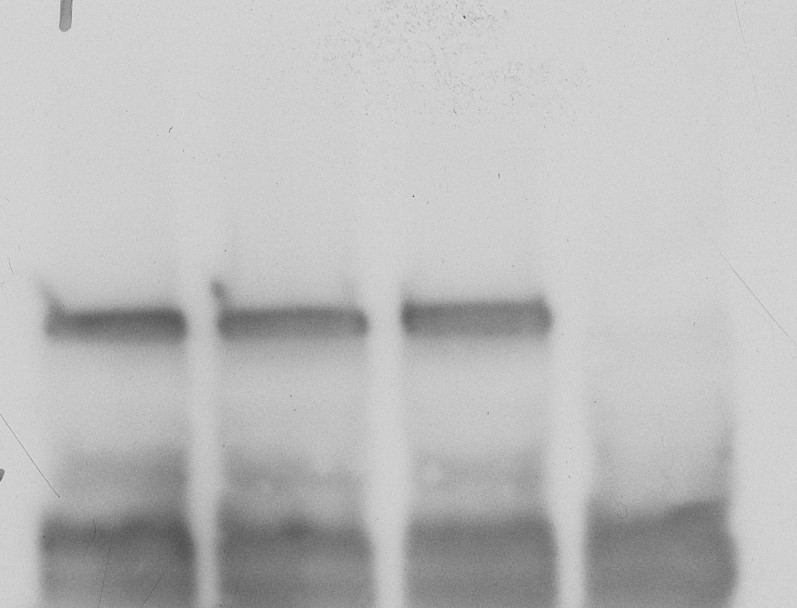

Supplement: Supplementary file 9 — Source data Fig. 6 [file 44318_2024_292_MOESM9_ESM.zip › Figure 6/6D/INPUT-Gal3.jpg]

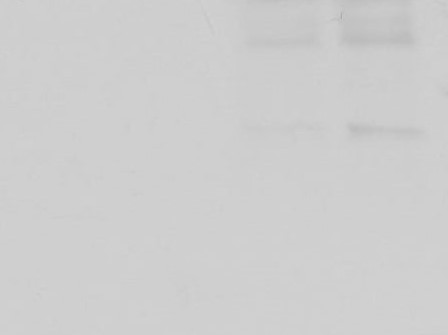

Supplement: Supplementary file 9 — Source data Fig. 6 [file 44318_2024_292_MOESM9_ESM.zip › Figure 6/6D/INPUT-P-PKR.jpg]

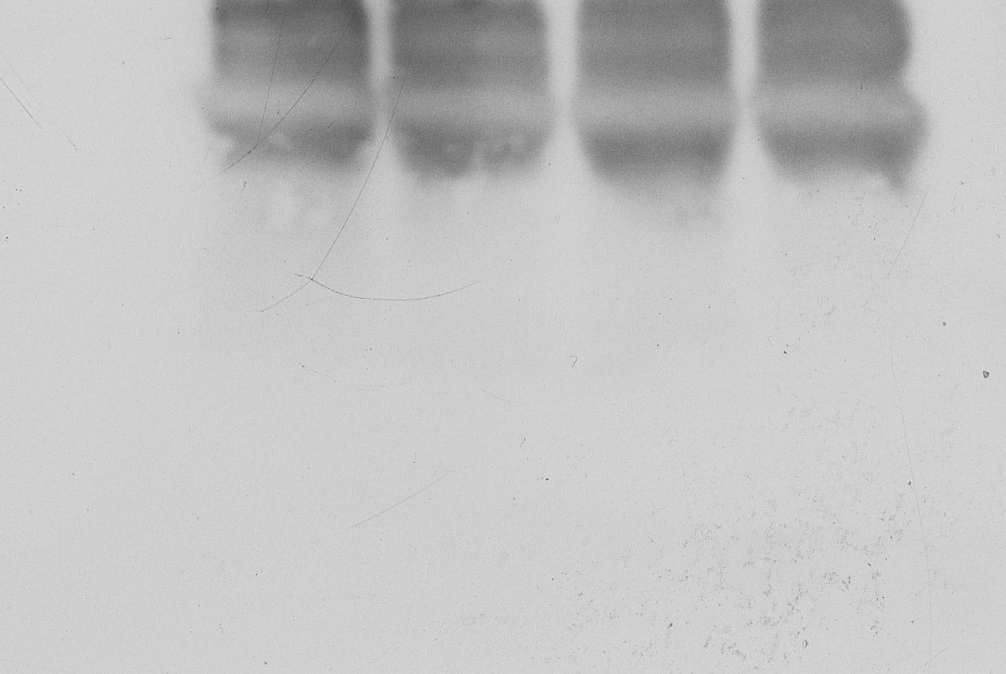

Supplement: Supplementary file 9 — Source data Fig. 6 [file 44318_2024_292_MOESM9_ESM.zip › Figure 6/6D/INPUT-PACT.jpg]

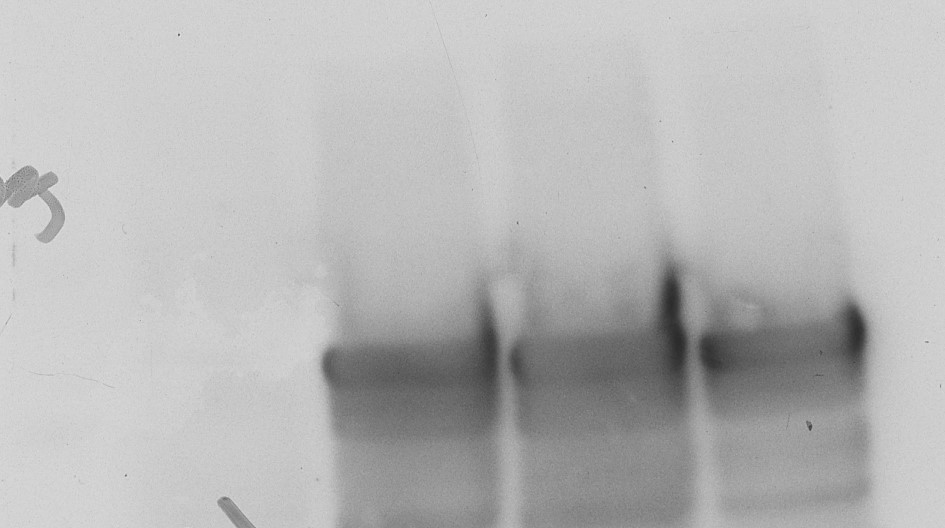

Supplement: Supplementary file 9 — Source data Fig. 6 [file 44318_2024_292_MOESM9_ESM.zip › Figure 6/6D/IP-FLAG.jpg]

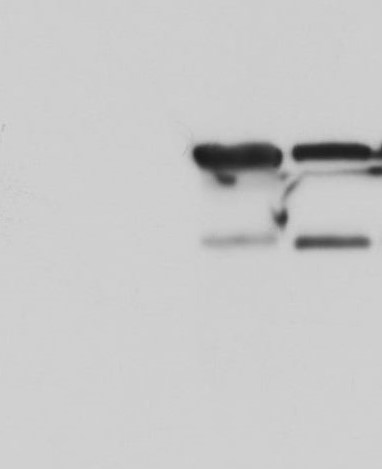

Supplement: Supplementary file 9 — Source data Fig. 6 [file 44318_2024_292_MOESM9_ESM.zip › Figure 6/6D/IP-PACT.jpg]

LLOMe: - - + +


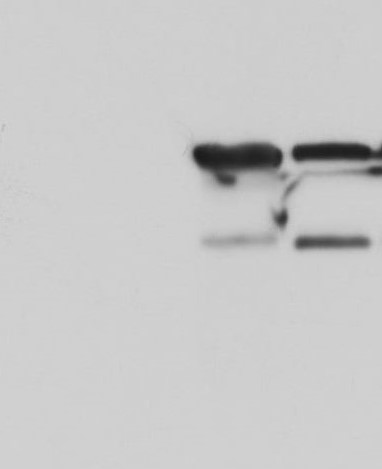


IP:

KDa

50

37

75

PACT


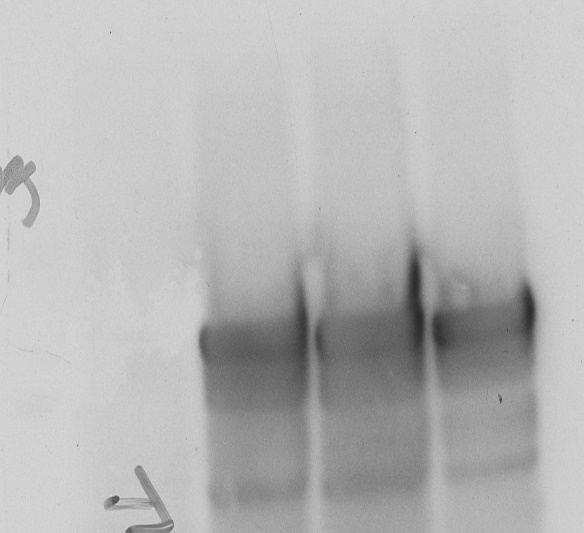


KDa

50

37

FLAG


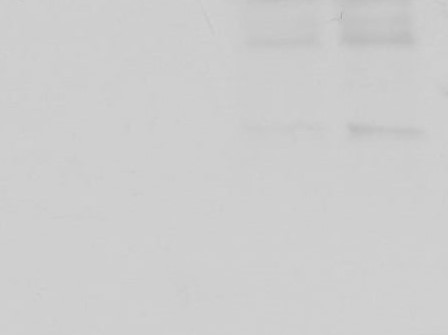


INPUT:

KDa

50

37

P-PKR


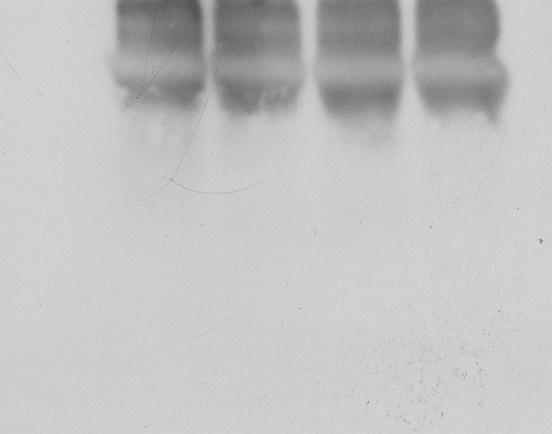


KDa

50

37

PACT

KDa

50

37

75


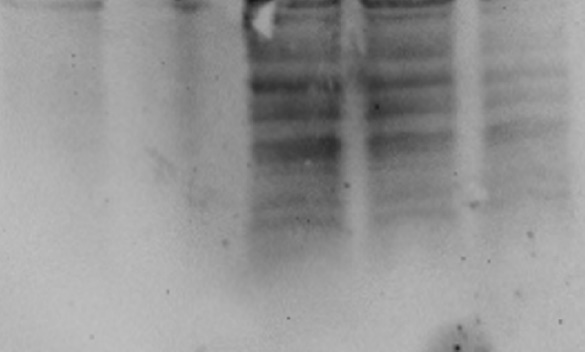


FLAG

KDa

50

25

75


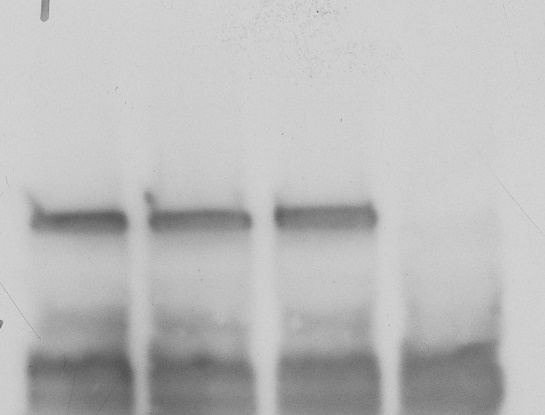


Gal3

KDa

95

150

KDa

95

150

Supplement: Supplementary file 9 — Source data Fig. 6 [file 44318_2024_292_MOESM9_ESM.zip › Figure 6/6D/README.docx]

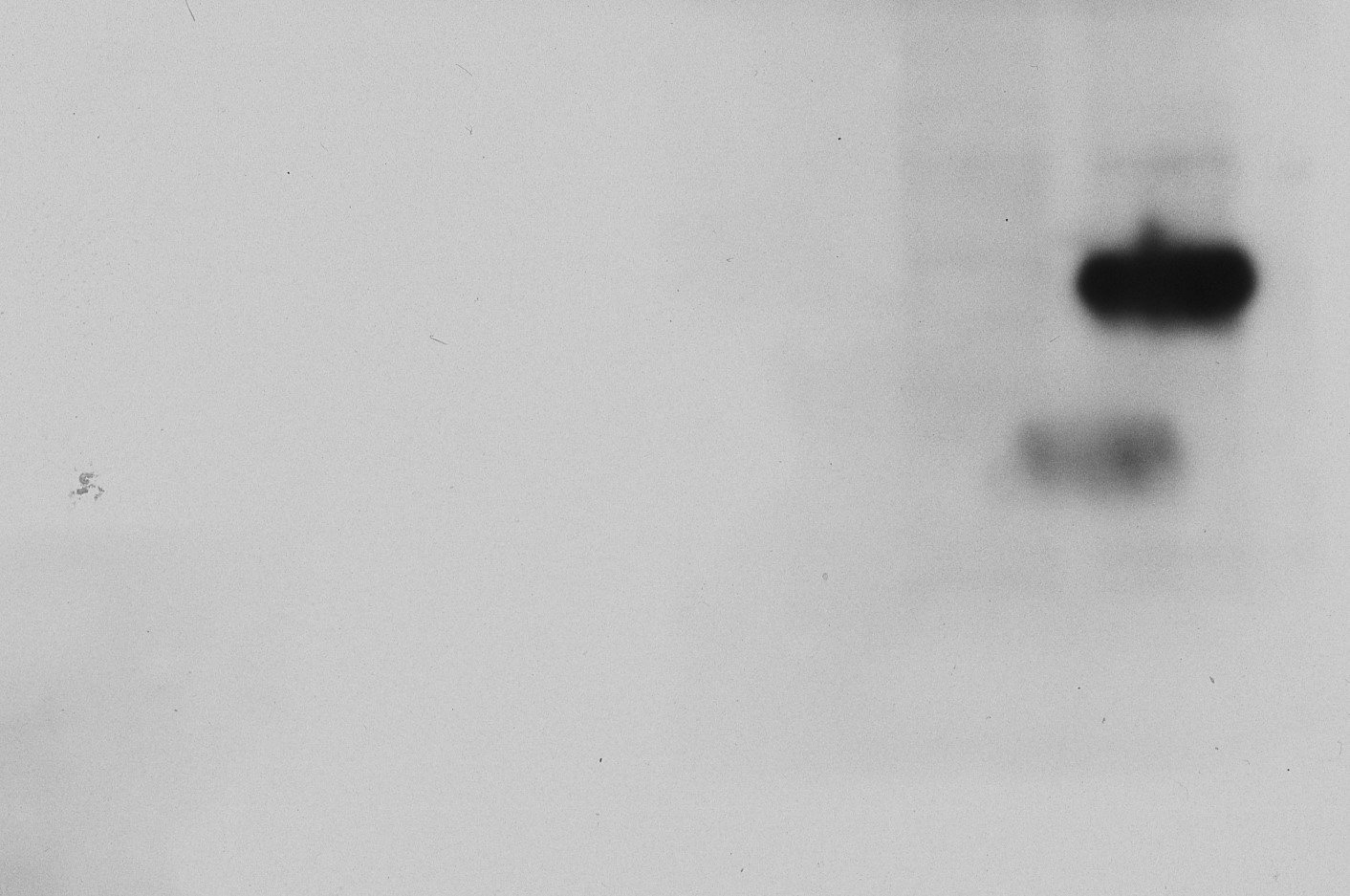

Supplement: Supplementary file 9 — Source data Fig. 6 [file 44318_2024_292_MOESM9_ESM.zip › Figure 6/6E/INPUT-FLAG.jpg]

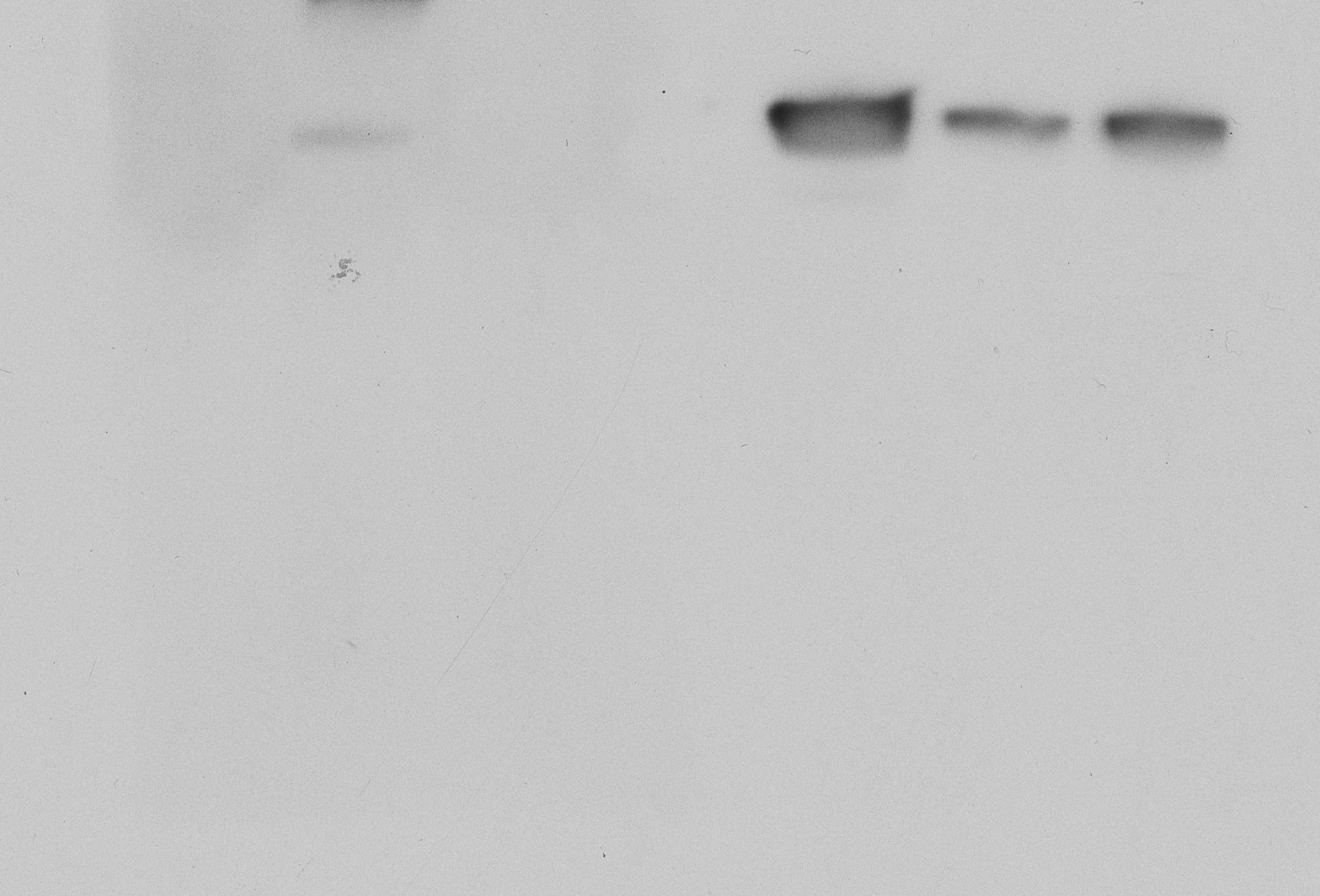

Supplement: Supplementary file 9 — Source data Fig. 6 [file 44318_2024_292_MOESM9_ESM.zip › Figure 6/6E/INPUT-Myc.jpg]

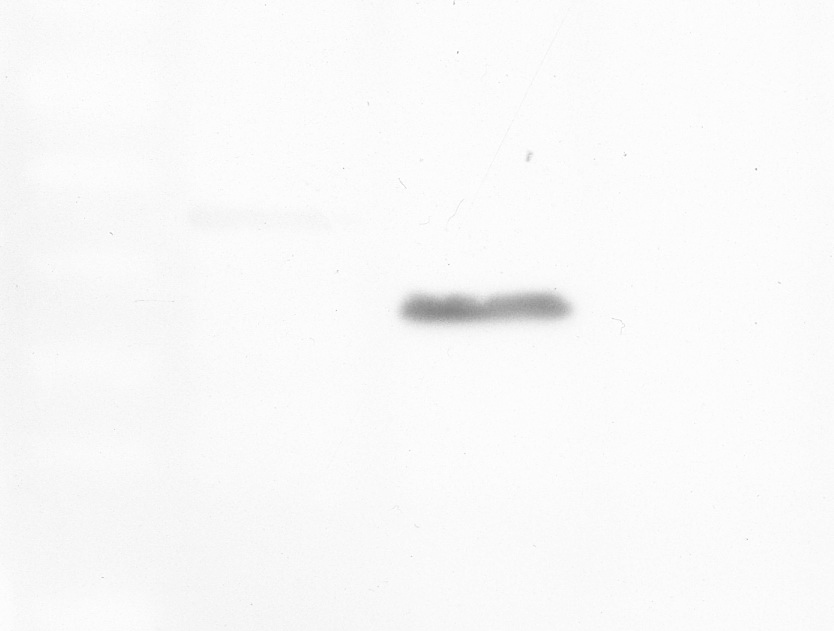

Supplement: Supplementary file 9 — Source data Fig. 6 [file 44318_2024_292_MOESM9_ESM.zip › Figure 6/6E/INPUT-P-PKR.jpg]

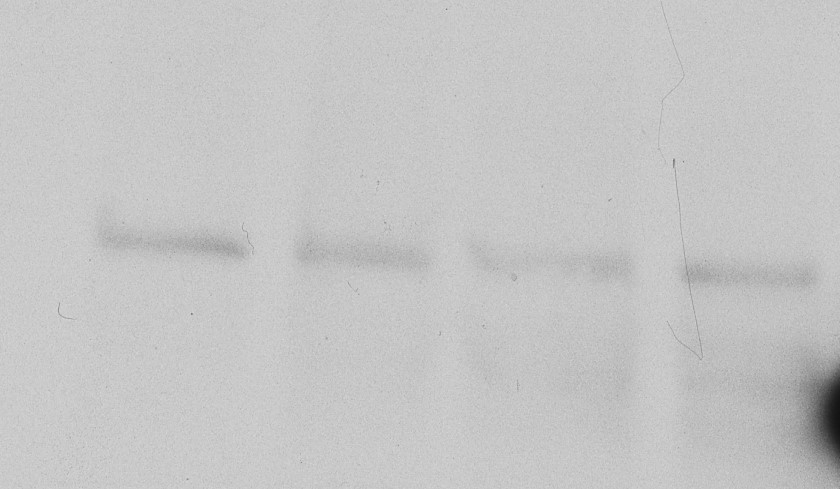

Supplement: Supplementary file 9 — Source data Fig. 6 [file 44318_2024_292_MOESM9_ESM.zip › Figure 6/6E/INPUT-PKR.jpg]

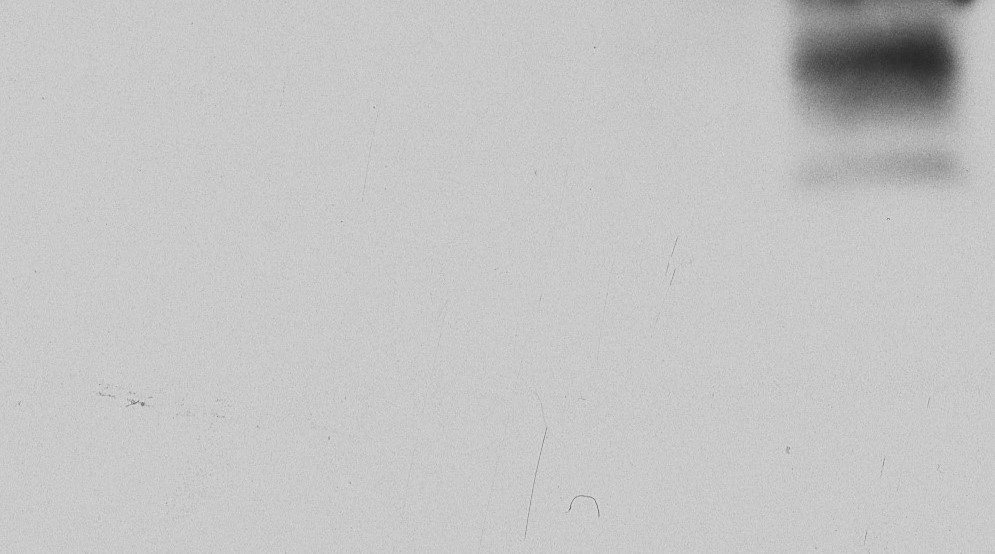

Supplement: Supplementary file 9 — Source data Fig. 6 [file 44318_2024_292_MOESM9_ESM.zip › Figure 6/6E/IP-FLAG.jpg]

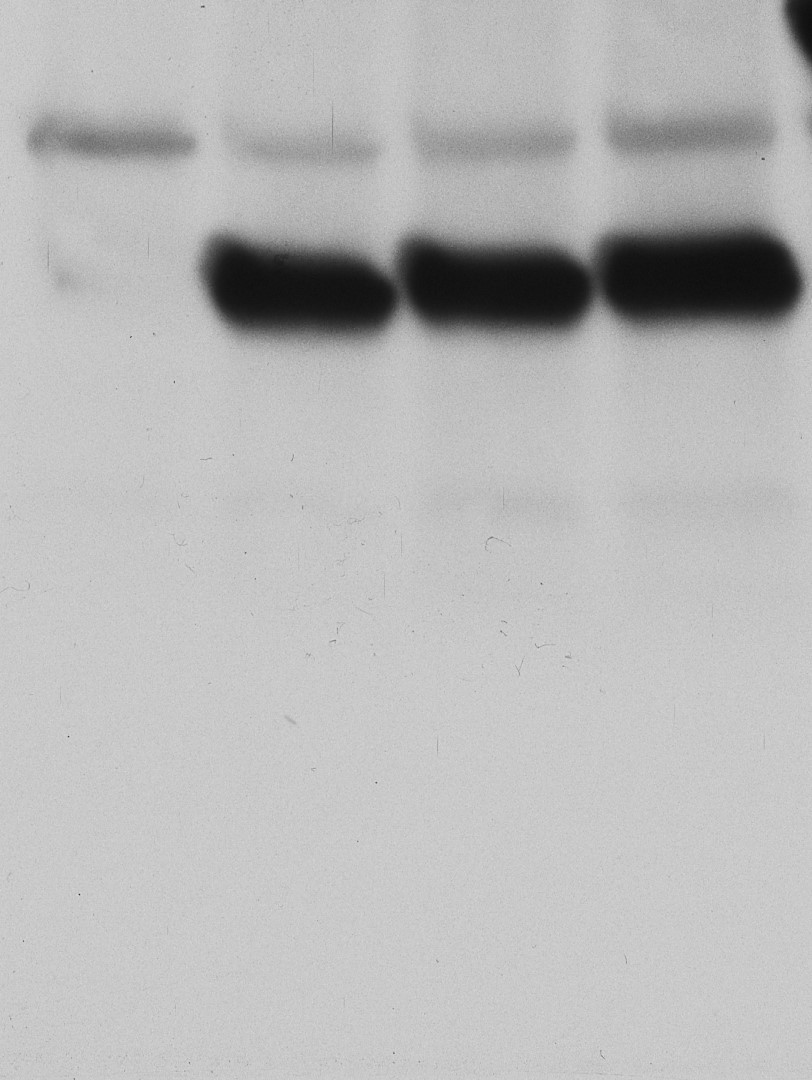

Supplement: Supplementary file 9 — Source data Fig. 6 [file 44318_2024_292_MOESM9_ESM.zip › Figure 6/6E/IP-Myc.jpg]

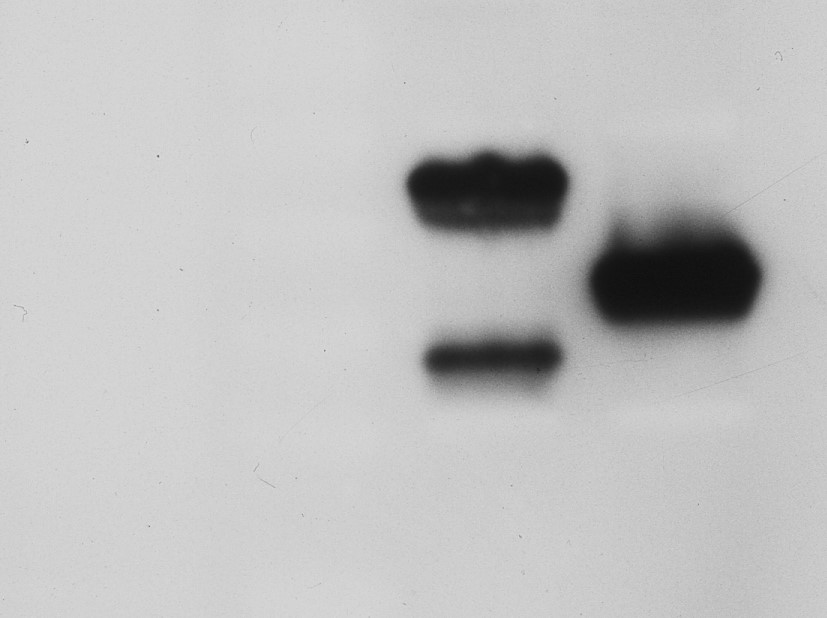

Supplement: Supplementary file 9 — Source data Fig. 6 [file 44318_2024_292_MOESM9_ESM.zip › Figure 6/6E/IP-PKR.jpg]

LLOMe: - - + +


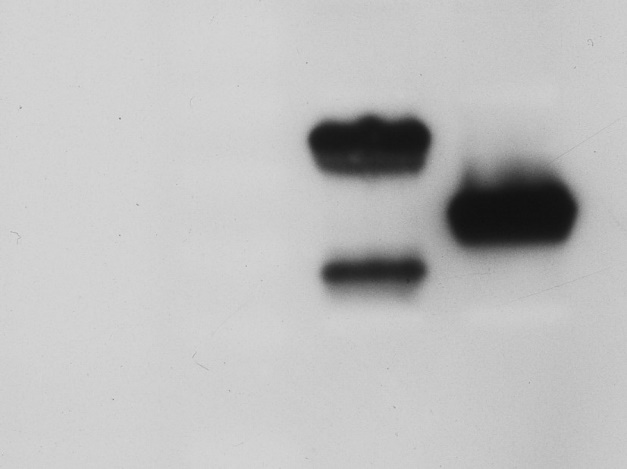


IP:

KDa

50

37

75

PKR


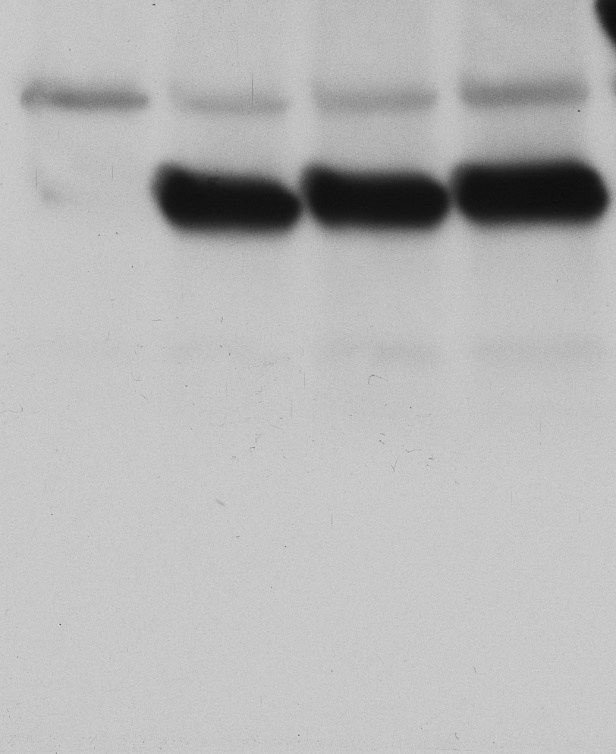


KDa

50

37

Myc


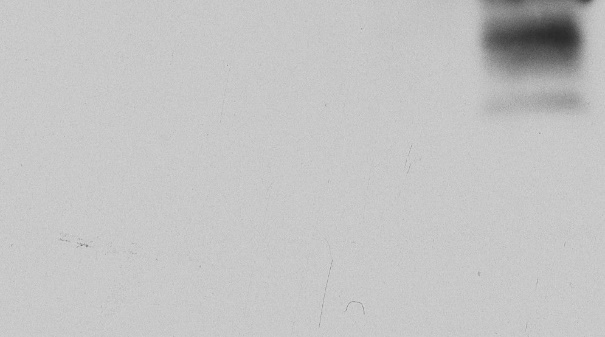


KDa

37

15

FLAG


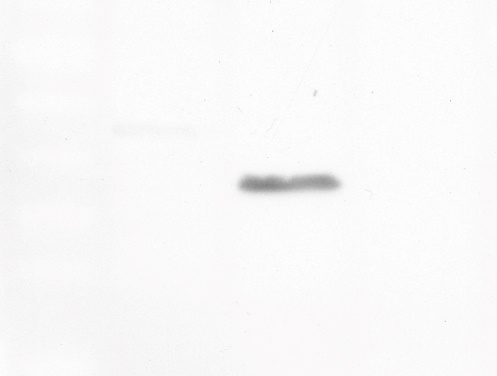


INPUT:

KDa

50

37

P-PKR

KDa

50

37

75


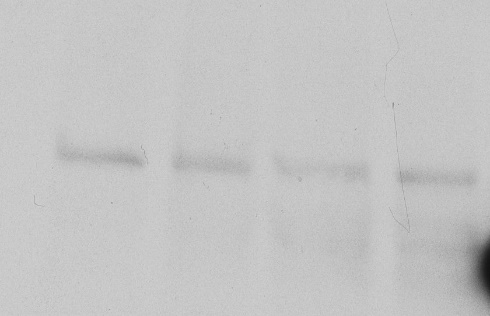


PKR

KDa

15

37


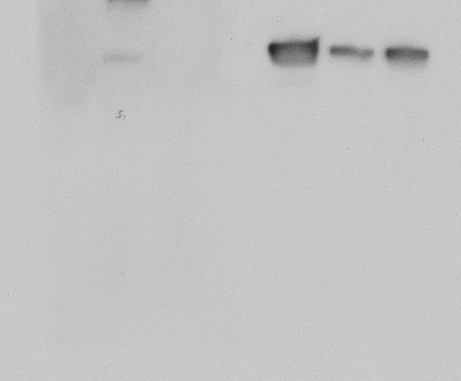


Myc


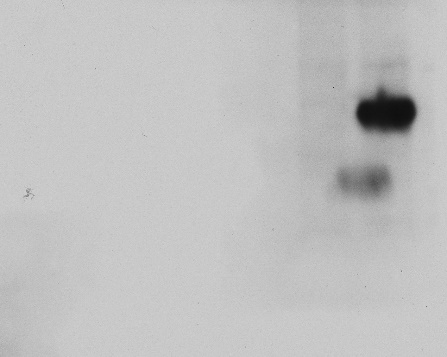


KDa

15

37

FLAG

KDa

95

150

KDa

95

150

Supplement: Supplementary file 9 — Source data Fig. 6 [file 44318_2024_292_MOESM9_ESM.zip › Figure 6/6E/README.docx]

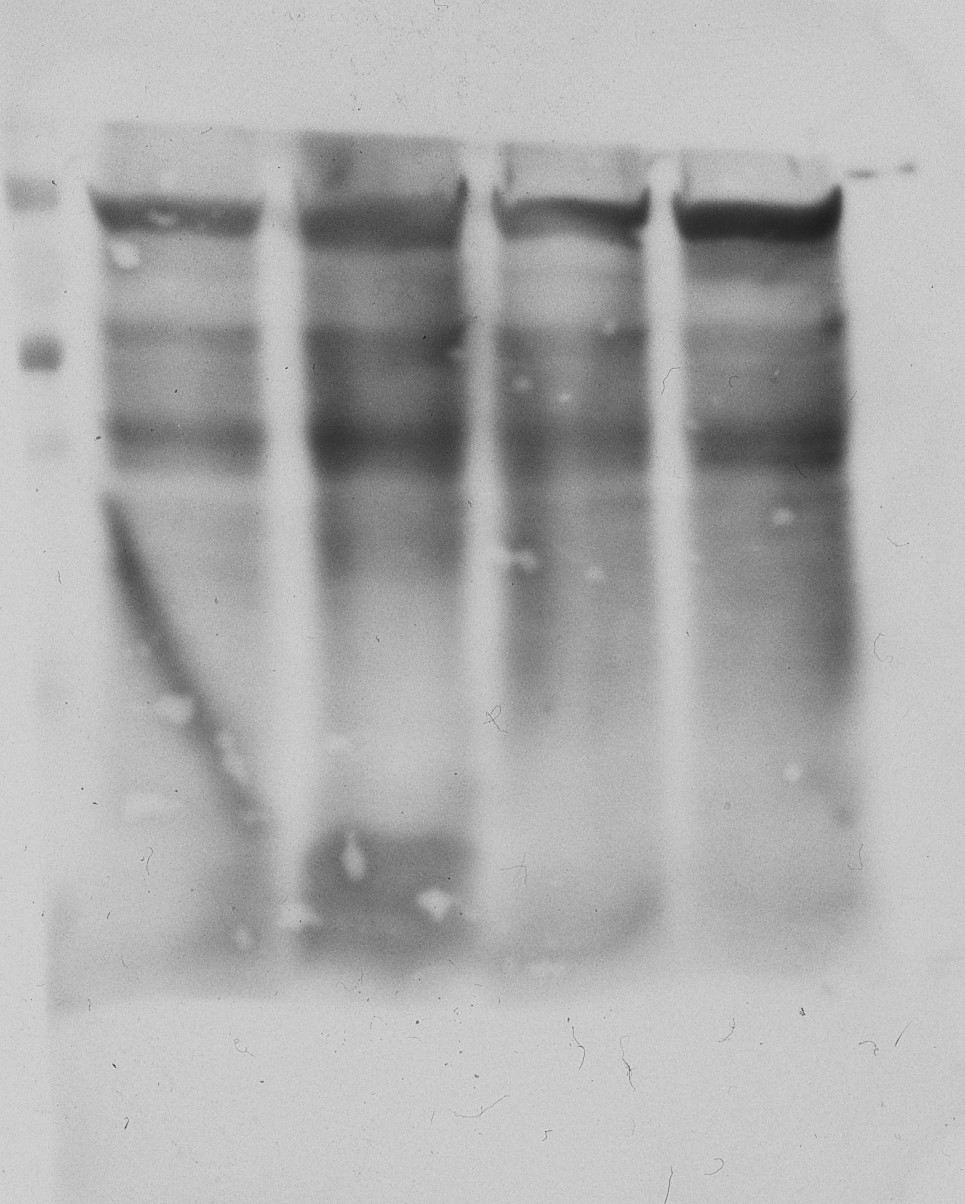

Supplement: Supplementary file 9 — Source data Fig. 6 [file 44318_2024_292_MOESM9_ESM.zip › Figure 6/6F/INPUT-FLAG.jpg]

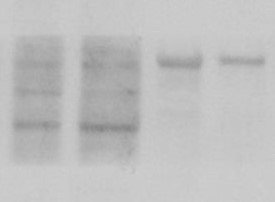

Supplement: Supplementary file 9 — Source data Fig. 6 [file 44318_2024_292_MOESM9_ESM.zip › Figure 6/6F/INPUT-GFP.jpg]

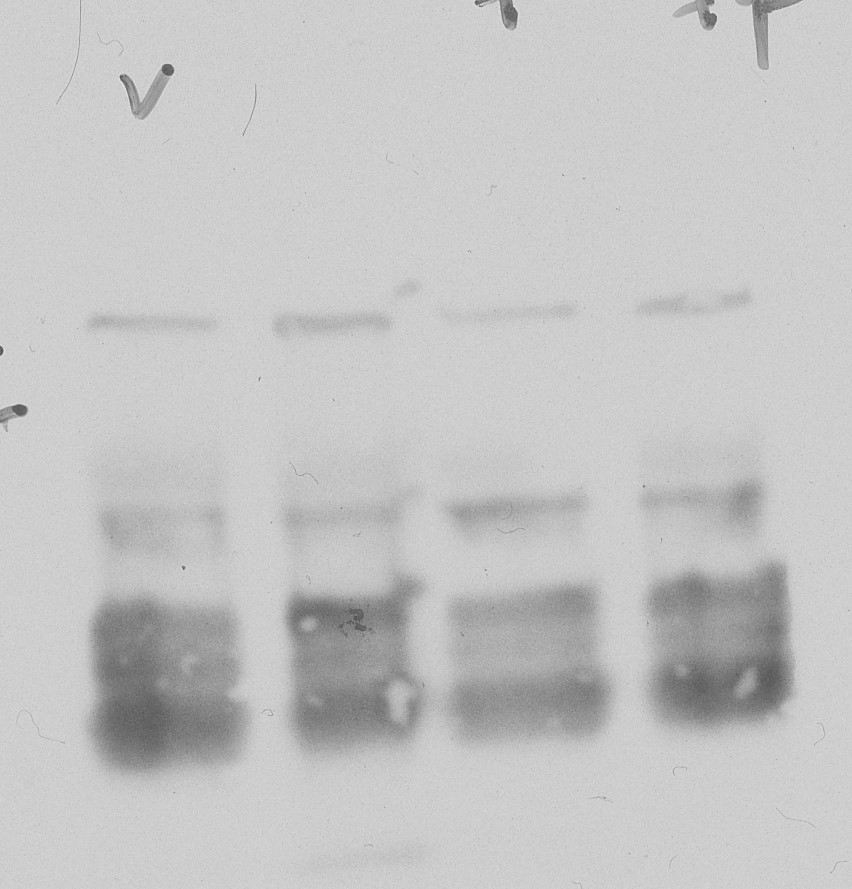

Supplement: Supplementary file 9 — Source data Fig. 6 [file 44318_2024_292_MOESM9_ESM.zip › Figure 6/6F/INPUT-PKR-PACT.jpg]

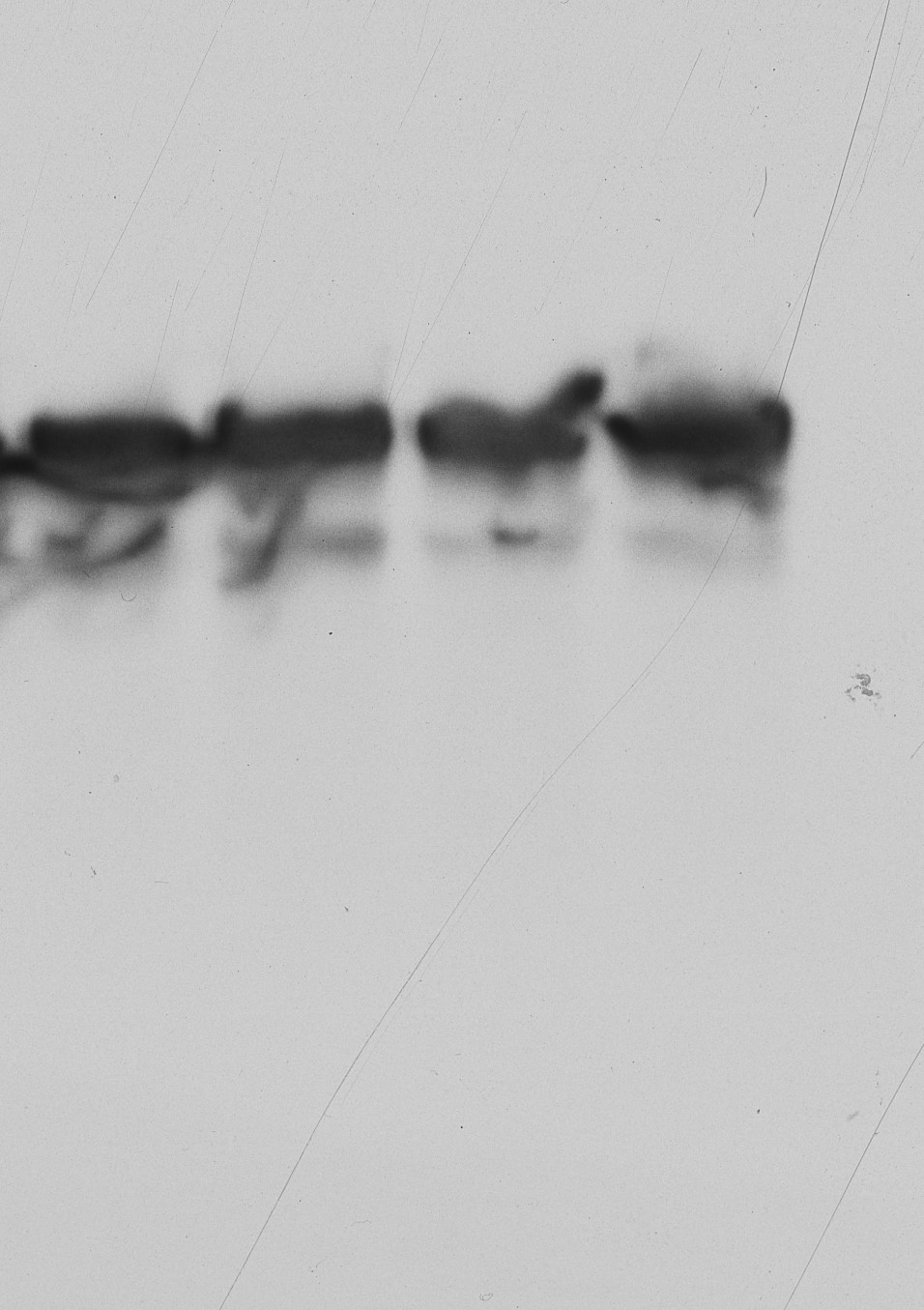

Supplement: Supplementary file 9 — Source data Fig. 6 [file 44318_2024_292_MOESM9_ESM.zip › Figure 6/6F/IP-FLAG.jpg]

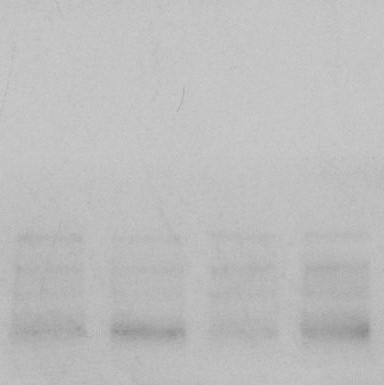

Supplement: Supplementary file 9 — Source data Fig. 6 [file 44318_2024_292_MOESM9_ESM.zip › Figure 6/6F/IP-PACT.jpg]

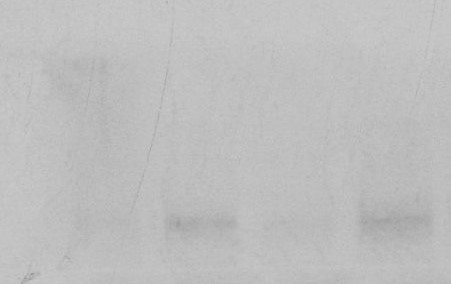

Supplement: Supplementary file 9 — Source data Fig. 6 [file 44318_2024_292_MOESM9_ESM.zip › Figure 6/6F/IP-PKR.jpg]

LLOMe: - + + +


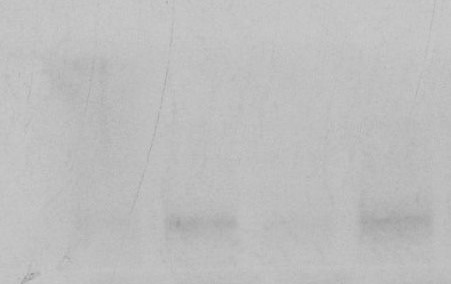


IP:

KDa

50

37

75

PKR


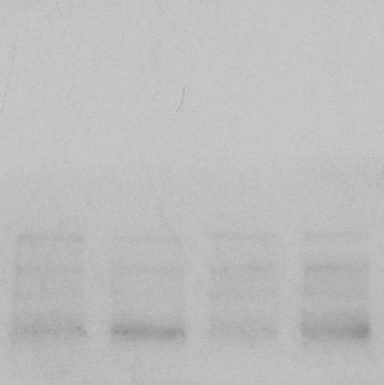


KDa

50

37

PACT


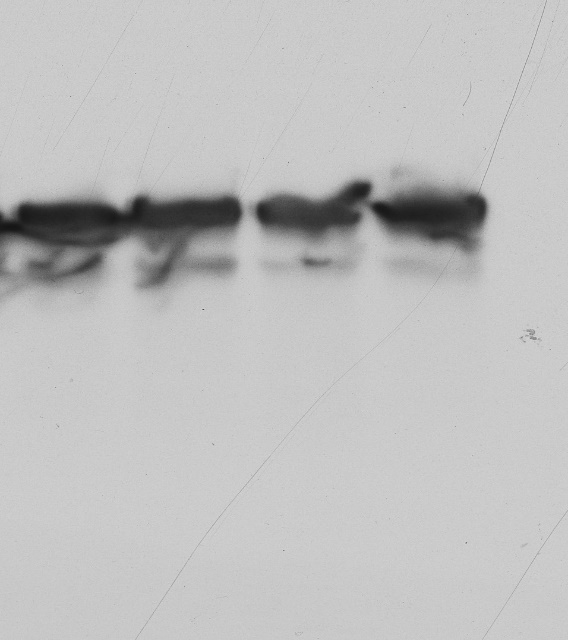


KDa

150

95

FLAG


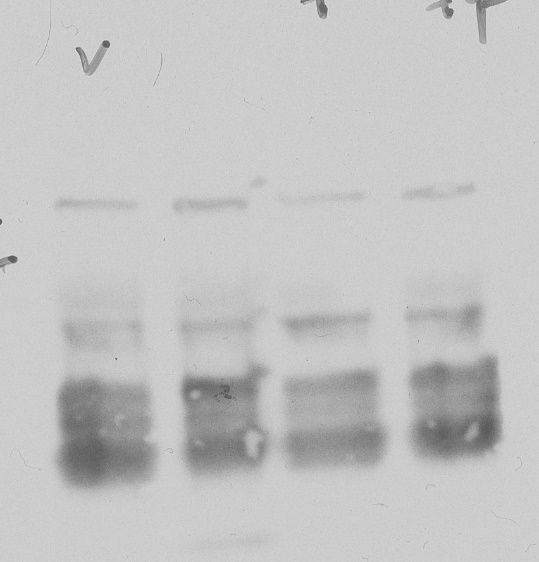


INPUT:

KDa

50

37

75

PKR

PACT


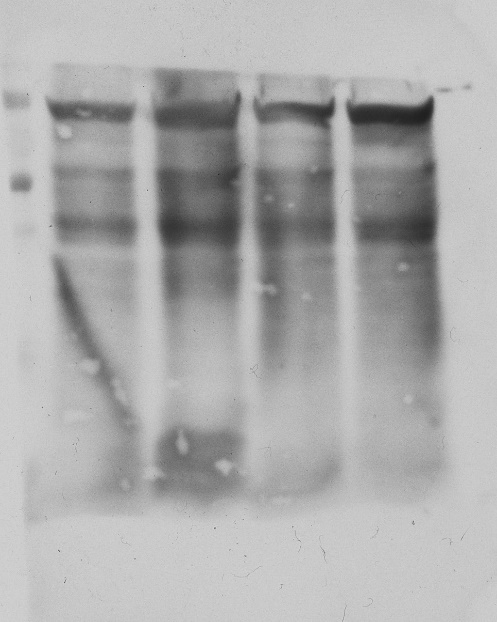


KDa

130

72

FLAG


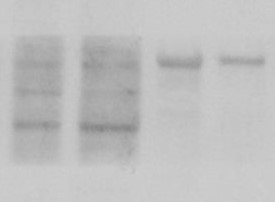


GFP

KDa

15

37

KDa

95

150

KDa

95

150

Supplement: Supplementary file 9 — Source data Fig. 6 [file 44318_2024_292_MOESM9_ESM.zip › Figure 6/6F/README.docx]

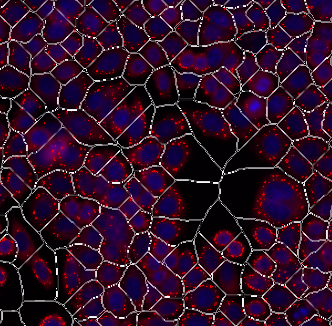

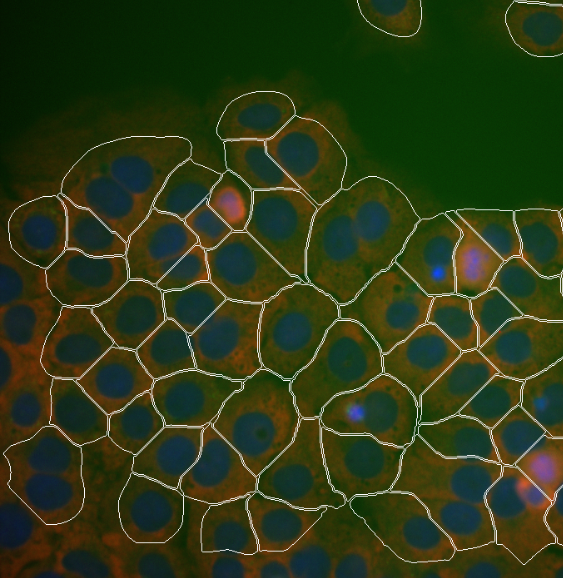

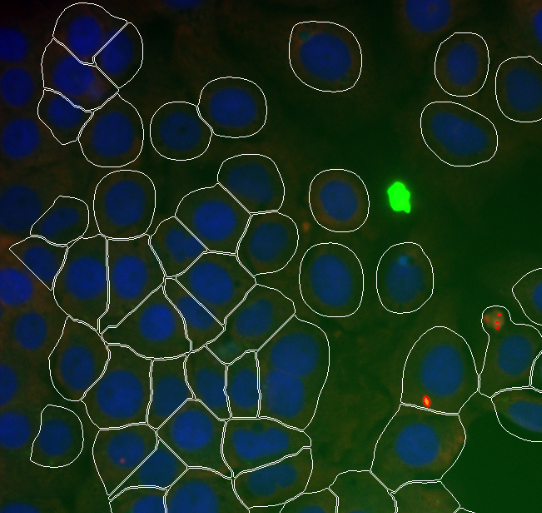

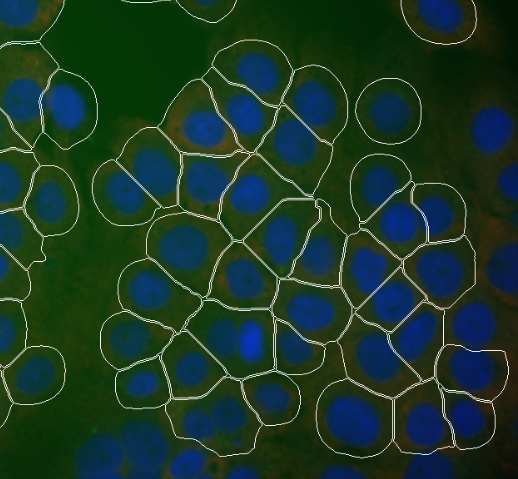


TS1 -1h

TS1 -NT

WT-NT

WT-1h

Supplement: Supplementary file 10 — Source data Fig. 7 [file 44318_2024_292_MOESM10_ESM.zip › Figure 7/7A/README.docx]

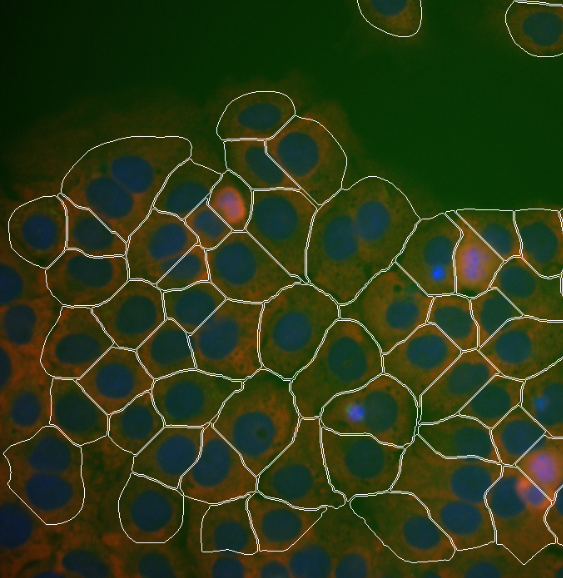

Supplement: Supplementary file 10 — Source data Fig. 7 [file 44318_2024_292_MOESM10_ESM.zip › Figure 7/7A/TS1-1h.tif]

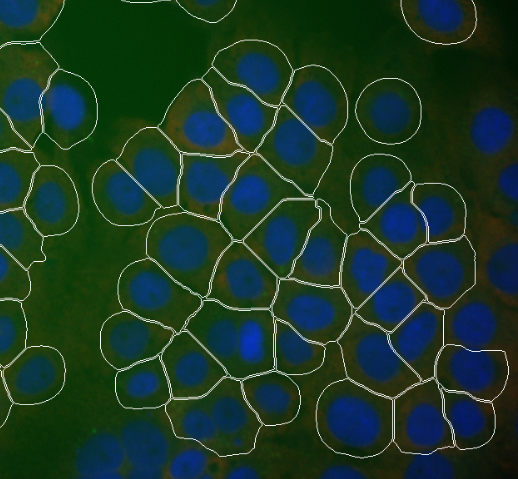

Supplement: Supplementary file 10 — Source data Fig. 7 [file 44318_2024_292_MOESM10_ESM.zip › Figure 7/7A/TS1-NT.tif]

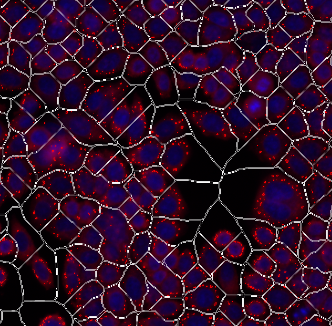

Supplement: Supplementary file 10 — Source data Fig. 7 [file 44318_2024_292_MOESM10_ESM.zip › Figure 7/7A/WT-1h.tif]

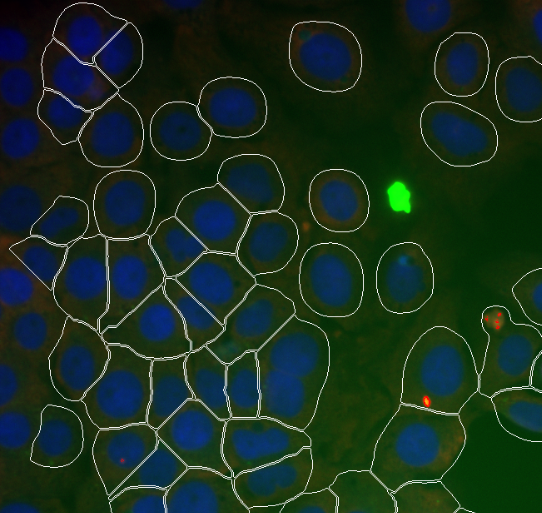

Supplement: Supplementary file 10 — Source data Fig. 7 [file 44318_2024_292_MOESM10_ESM.zip › Figure 7/7A/WT-NT.tif]

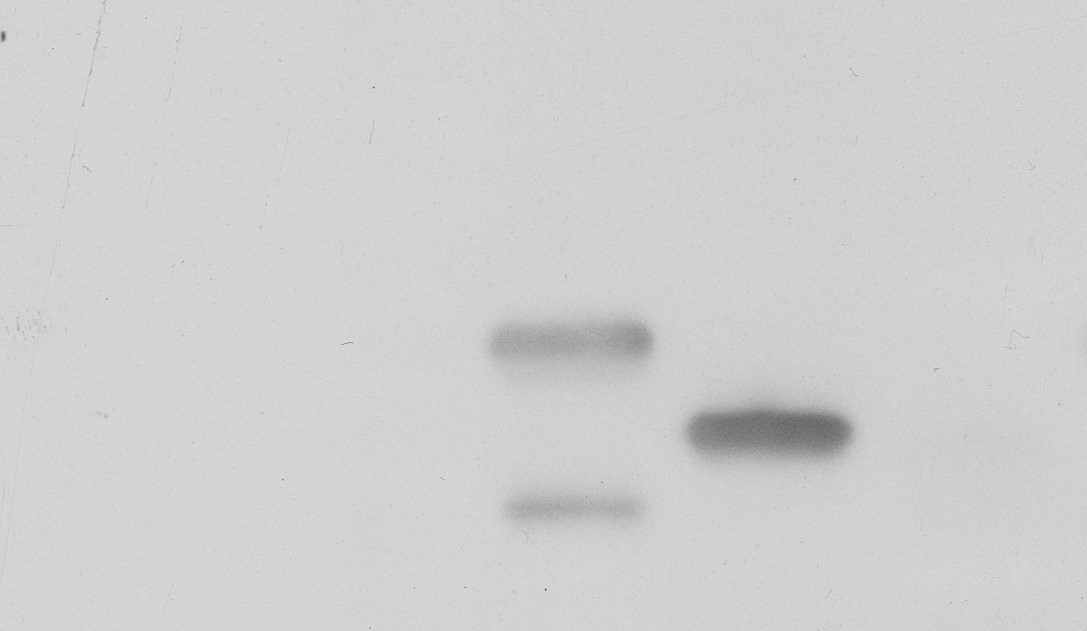

Supplement: Supplementary file 10 — Source data Fig. 7 [file 44318_2024_292_MOESM10_ESM.zip › Figure 7/7B/P-PKR.jpg]

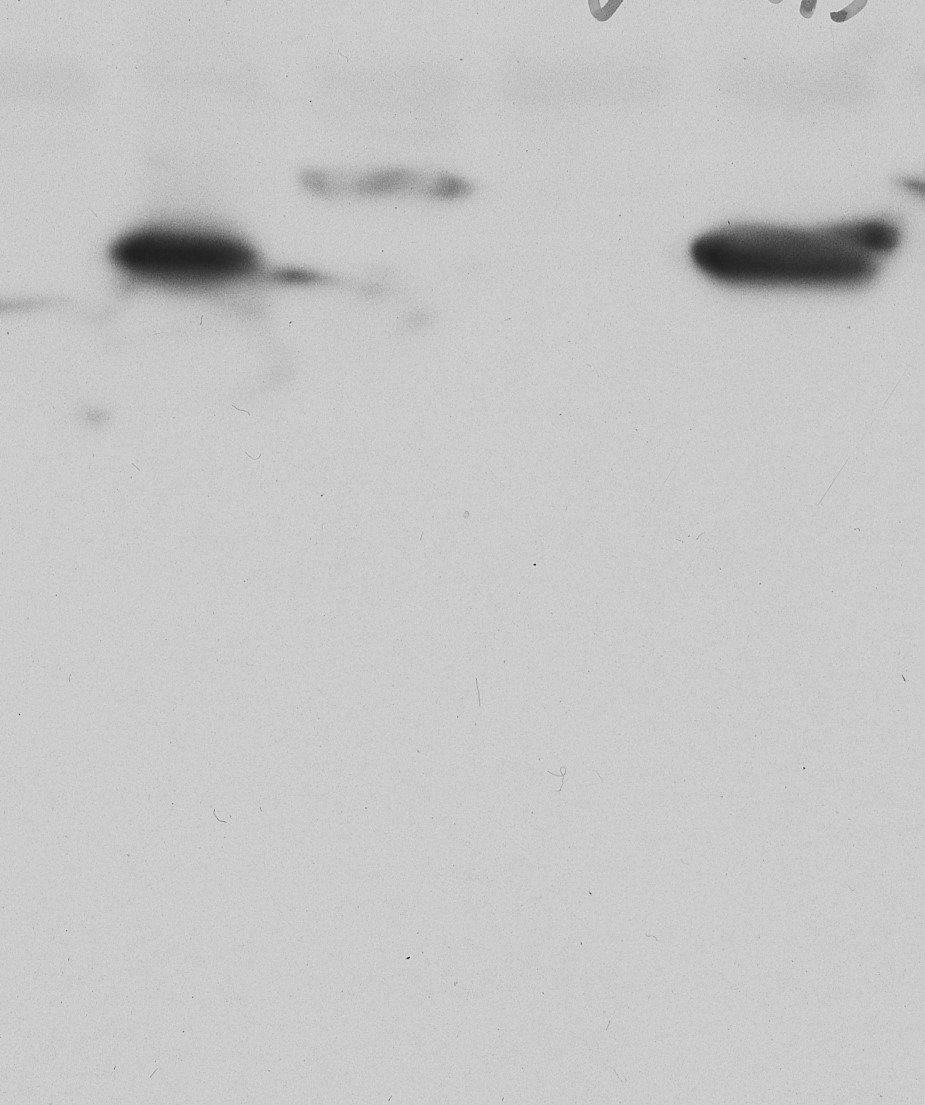

Supplement: Supplementary file 10 — Source data Fig. 7 [file 44318_2024_292_MOESM10_ESM.zip › Figure 7/7B/P-eIF2a.jpg]

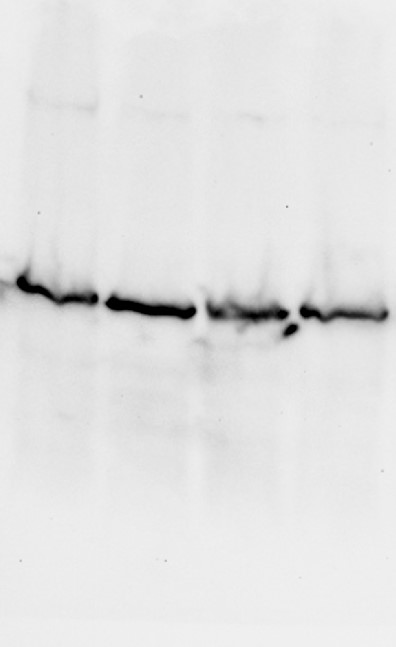

Supplement: Supplementary file 10 — Source data Fig. 7 [file 44318_2024_292_MOESM10_ESM.zip › Figure 7/7B/PKR.jpg]

LLOMe: - - + +


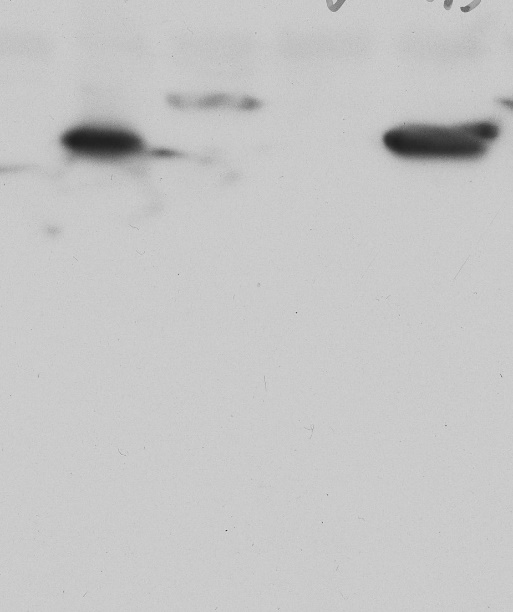


KDa

15

30

P-eIF2a


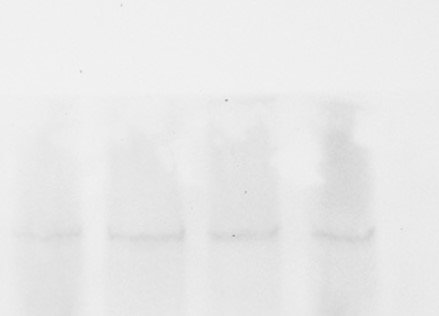


KDa

50

37

75

eIF2a


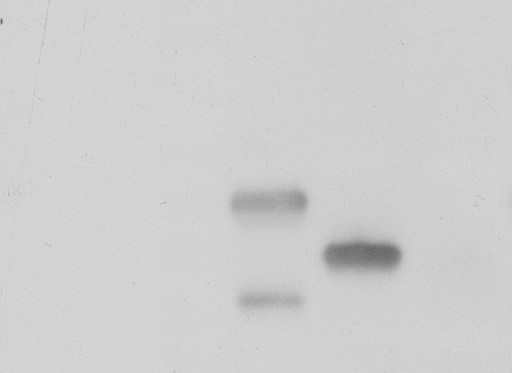


KDa

50

37

75

P-PKR


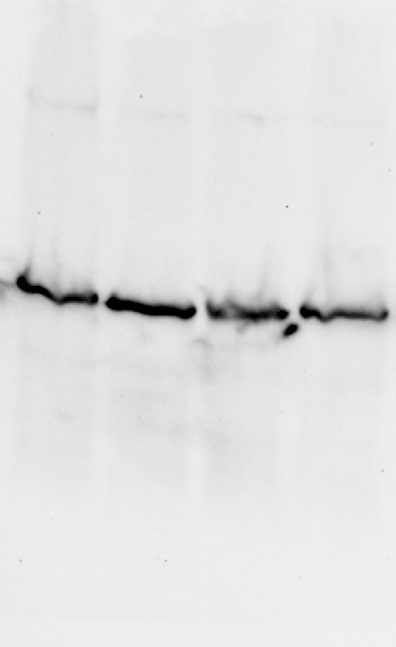


KDa

50

37

75

PKR

KDa

50

37

75


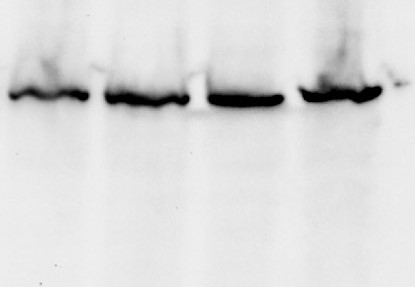


b-actin

KDa

95

150

KDa

95

150

Supplement: Supplementary file 10 — Source data Fig. 7 [file 44318_2024_292_MOESM10_ESM.zip › Figure 7/7B/README.docx]

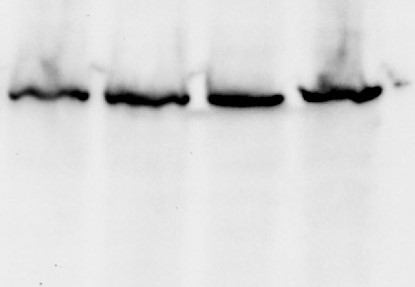

Supplement: Supplementary file 10 — Source data Fig. 7 [file 44318_2024_292_MOESM10_ESM.zip › Figure 7/7B/b-actin.jpg]

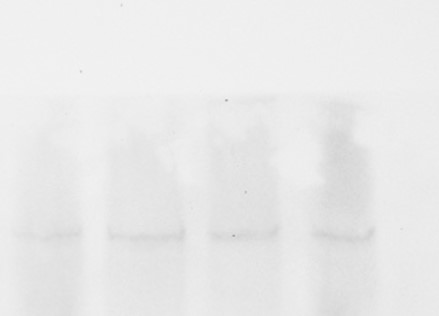

Supplement: Supplementary file 10 — Source data Fig. 7 [file 44318_2024_292_MOESM10_ESM.zip › Figure 7/7B/eIF2a.jpg]

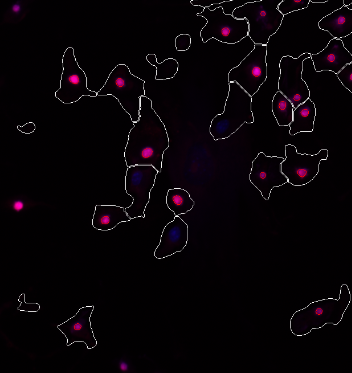

Supplement: Supplementary file 10 — Source data Fig. 7 [file 44318_2024_292_MOESM10_ESM.zip › Figure 7/7C/KO-1h.tif]

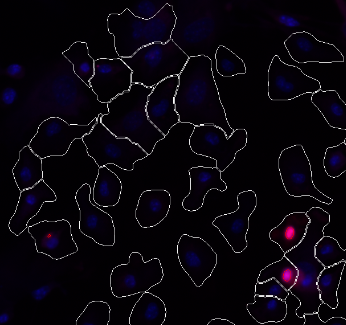

Supplement: Supplementary file 10 — Source data Fig. 7 [file 44318_2024_292_MOESM10_ESM.zip › Figure 7/7C/KO-NT.tif]

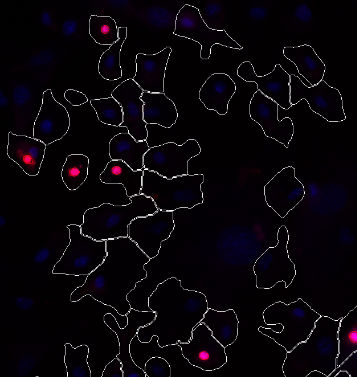

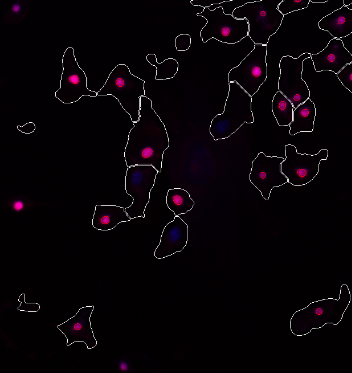

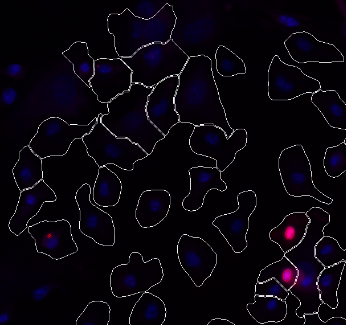

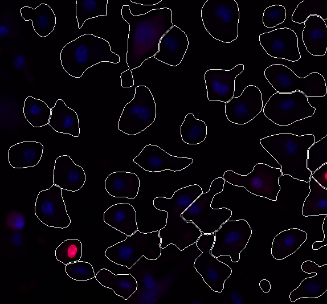


KO -1h

KO -NT

WT-NT

WT-1h

Supplement: Supplementary file 10 — Source data Fig. 7 [file 44318_2024_292_MOESM10_ESM.zip › Figure 7/7C/README.docx]

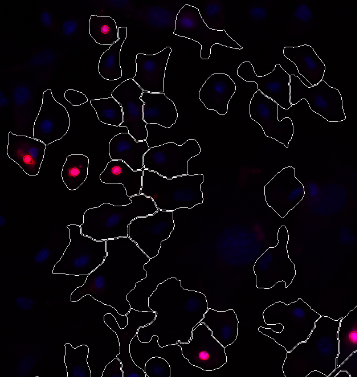

Supplement: Supplementary file 10 — Source data Fig. 7 [file 44318_2024_292_MOESM10_ESM.zip › Figure 7/7C/WT-1h.tif]

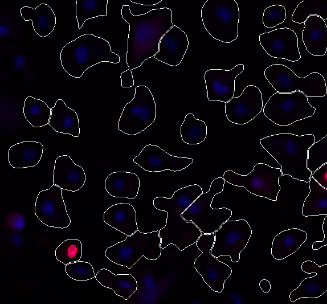

Supplement: Supplementary file 10 — Source data Fig. 7 [file 44318_2024_292_MOESM10_ESM.zip › Figure 7/7C/WT-NT.tif]
