# Supplementary material for: Calcium signaling from damaged lysosomes induces cytoprotective stress granules
Source: EMBO J. 2024 Nov 12;43(24):6410–43. doi: 10.1038/s44318-024-00292-1 (PMC11649789; doi:10.1038/s44318-024-00292-1)
Supplement: Supplementary file 11 — Expanded View Figures [file 44318_2024_292_MOESM11_ESM.pdf]

Expanded View Figures

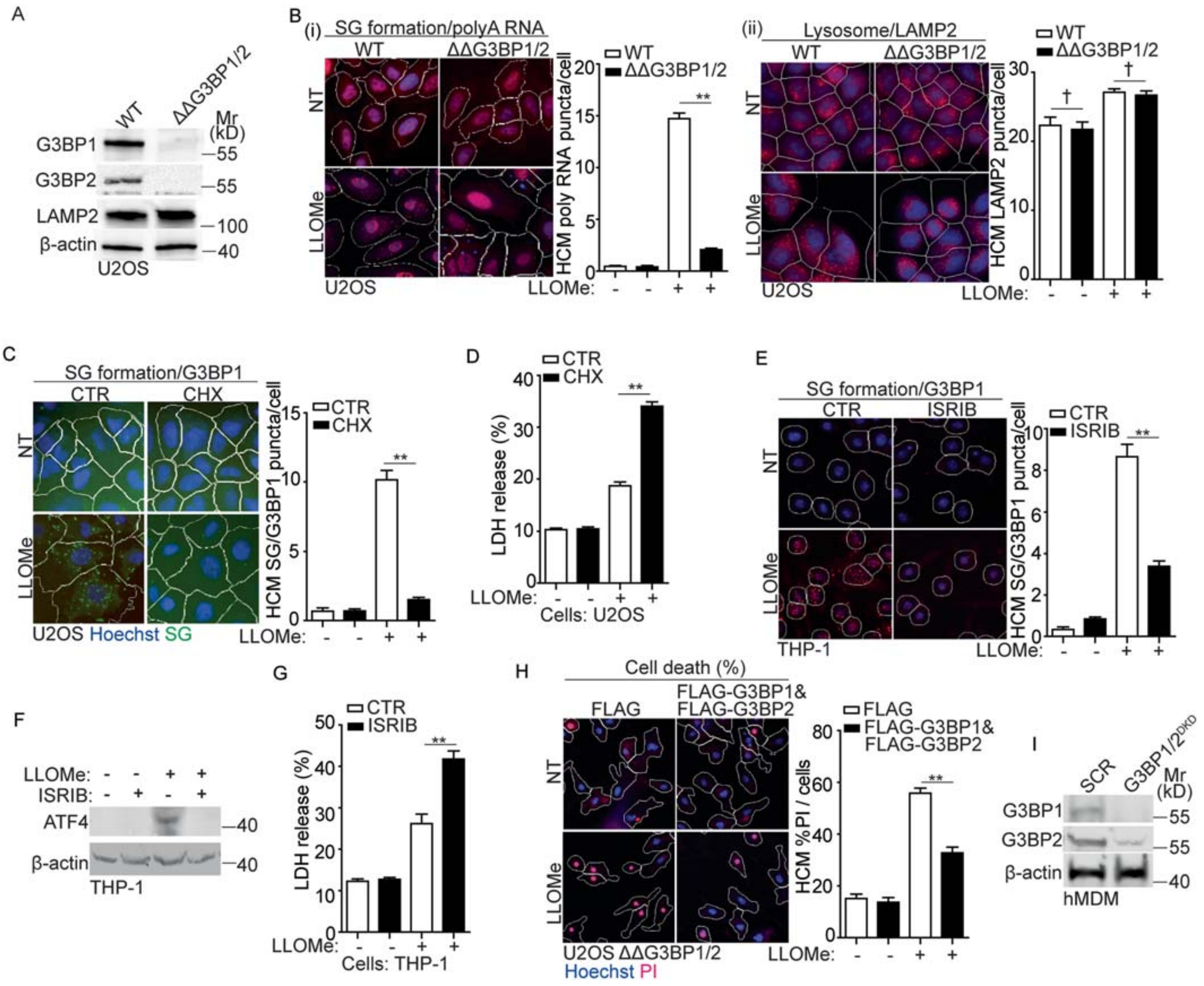

Figure EV1. Stress granule formation is important for cell survival during lysosomal damage.

(**A**) Immunoblot analysis of G3BP1 and G3BP2 in U2OS WT and ΔΔG3BP1/2 cells. (**B**) Quantification by high-content microscopy (HCM) of polyA RNA (Cy3-oligo[dT]) by FISH (i) and LAMP2 (ii) in U2OS WT and ΔΔG3BP1/2 cells. Cells were treated with 2 mM LLOMe for 30 min. White masks, algorithm-defined cell boundaries (primary objects); red masks, computer-identified polyA RNA or LAMP2 puncta respectively (target objects). (**C**) Quantification by HCM of G3BP1 puncta in U2OS cells. Cells were treated with 2 mM LLOMe in the presence or absence of 10 μg/ml cycloheximide (CHX) for 30 min. White masks, algorithm-defined cell boundaries; green masks, computer-identified G3BP1 puncta. (**D**) Cell death analysis of supernatants of U2OS cells by a LDH release assay. Cells were treated with 2 mM LLOMe in the presence or absence of 10 μg/ml CHX for 30 min. (**E**) Quantification by HCM of G3BP1 puncta in human monocytic THP-1 cells. Cells were treated with 1 mM LLOMe in the presence or absence of 200 nM ISRIB for 30 min. White masks, algorithm-defined cell boundaries; red masks, computer-identified G3BP1 puncta. (**F**) Immunoblot analysis of ATF4 in THP-1 cells treated with 1 mM LLOMe in the presence or absence of 200 nM ISRIB for 30 min. (**G**) Cell death analysis of supernatants of THP-1 cells by a LDH release assay. Cells were treated with 1 mM LLOMe in the presence or absence of 200 nM ISRIB for 30 min. (**H**) Quantification of cell death by HCM using a propidium iodide (PI) uptake assay in U2OS G3BP1&2 double knockout (ΔΔG3BP1/2) cells overexpressing either FLAG or FLAG-G3BP1 & FLAG-G3BP2. Cells were treated with 2 mM LLOMe for 30 min, and then stained with propidium iodide (PI) (dead cells) and Hoechst-33342 (total cells). White masks, algorithm-defined cell boundaries; red masks, computer-identified PI+ nuclei. (**I**) Immunoblot analysis of the protein level of G3BP1 and G3BP2 in hMDM transfected with scrambled siRNA as control (SCR) or G3BP1 and G3BP2 siRNA for double knockdown (DKD). CTR, control; NT, untreated cells. Data, means ± SEM ($n = 3$); HCM: $n \geq 3$ (each experiment: 500 valid primary objects/cells per well, ≥5 wells/sample). †$p \geq 0.05$ (not significant), **$p < 0.01$, ANOVA. See also Fig. 1.

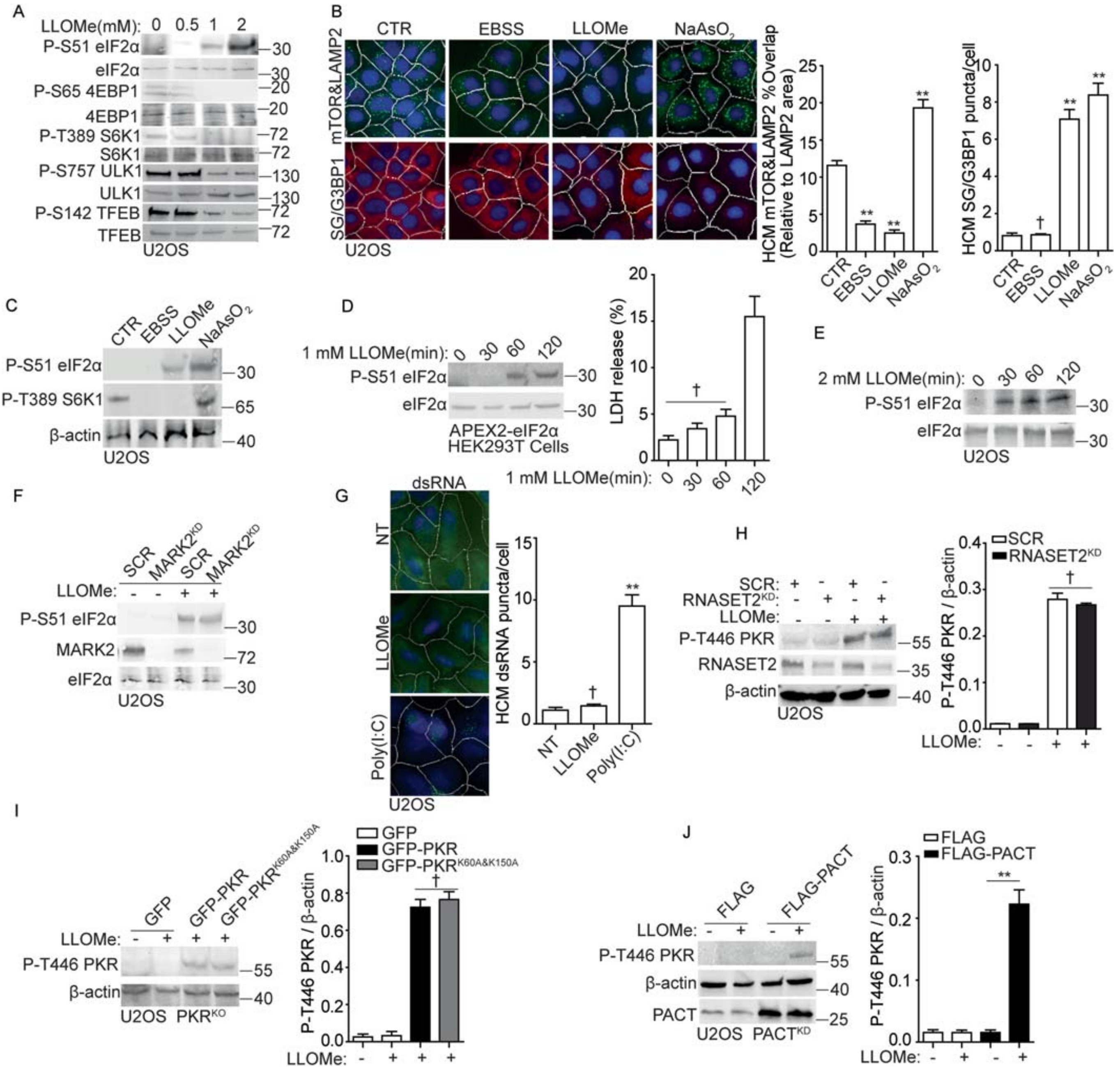

Figure EV2. PACT-PKR- eIF2α pathway controls stress granule formation in response to lysosomal damage.

(**A**) Immunoblot analysis of phosphorylation of eIF2α (S51), 4EBP1 (Ser65), S6K (Thr389), ULK1 (Ser757) and TFEB (Ser142) in U2OS cells treated with the indicated dose of LLOMe for 30 min. (**B**) Quantification by HCM of overlaps between mTOR and LAMP2 or G3BP1 puncta in U2OS cells. Cells were treated with EBSS, 2 mM LLOMe or 100 μM NaAsO2 for 30 min. White masks, algorithm-defined cell boundaries; green masks, computer-identified overlap between mTOR and LAMP2; red masks, computer-identified G3BP1 puncta. (**C**) Immunoblot analysis of phosphorylation of eIF2α (S51) and S6K1 (T389) in U2OS cells treated as in (**B**). (**D**) Immunoblot analysis of phosphorylation of eIF2α (S51) and cell death analysis by a LDH release assay in HEK293T cells expressing APEX2-eIF2α. Cells were treated with 1 mM LLOMe for the indicated durations. (**E**) Immunoblot analysis of phosphorylation of eIF2α (S51) in U2OS cells. Cells were treated with 2 mM LLOMe for the indicated durations. (**F**) Immunoblot analysis of phosphorylation of eIF2α (S51) in U2OS cells transfected with either scrambled siRNA as control (SCR) or MARK2 siRNA for knockdown ($MARK2^{KD}$). Cells were treated with 2 mM LLOMe for 30 min. (**G**) Quantification by HCM of dsRNA puncta in U2OS cells. Cells were treated with 2 mM LLOMe or 100 ng/mL Poly (I:C) for 30 min. Green masks, computer-identified dsRNA puncta. (**H**) Immunoblot analysis of phosphorylation of PKR (T446) in U2OS cells transfected with either scrambled siRNA as control (SCR) or RNASET2 siRNA for knockdown ($RNASET2^{KD}$). Cells were treated with 2 mM LLOMe for 30 min. The level of phosphorylation of PKR (T446) was quantified based on three independent experiments. (**I**) Immunoblot analysis of phosphorylation of PKR (T446) in PKR^{KO} U2OS G3BP1-GFP cells, overexpressing GFP, GFP-PKR and $GFP\text{-}PKR^{K60A\&K150A}$. Cells were treated with 2 mM LLOMe for 30 min. The level of phosphorylation of PKR (T446) was quantified based on three independent experiments. (**J**) Immunoblot analysis of phosphorylation of PKR (T446) in U2OS PACT knockdown cells ($PACT^{KD}$) overexpressing FLAG or FLAG-PACT. Cells were treated with 2 mM LLOMe for 30 min. The level of phosphorylation of PKR (T446) was quantified based on three independent experiments. CTR, control. Data, means ± SEM ($n = 3$); HCM: $n \geq 3$ (each experiment: 500 valid primary objects/cells per well, ≥5 wells/sample). †$p \geq 0.05$ (not significant), **$p < 0.01$, ANOVA. See also Figs. 2 and 3.

 © The Author(s)

A

Proteomic analysis of lysosomes:

LysoIP	Jia J. et al., 2022	Eapen V. et al., 2021	Wyant G. et al., 2018
**Gene Name**	**Peptide count**	**Peptide count**	**Peptide count**
eIF2α/EIF2S1	13	3	22
PKR/EIF2AK2	10	1	18
PACT/PRKRA	3	0	14

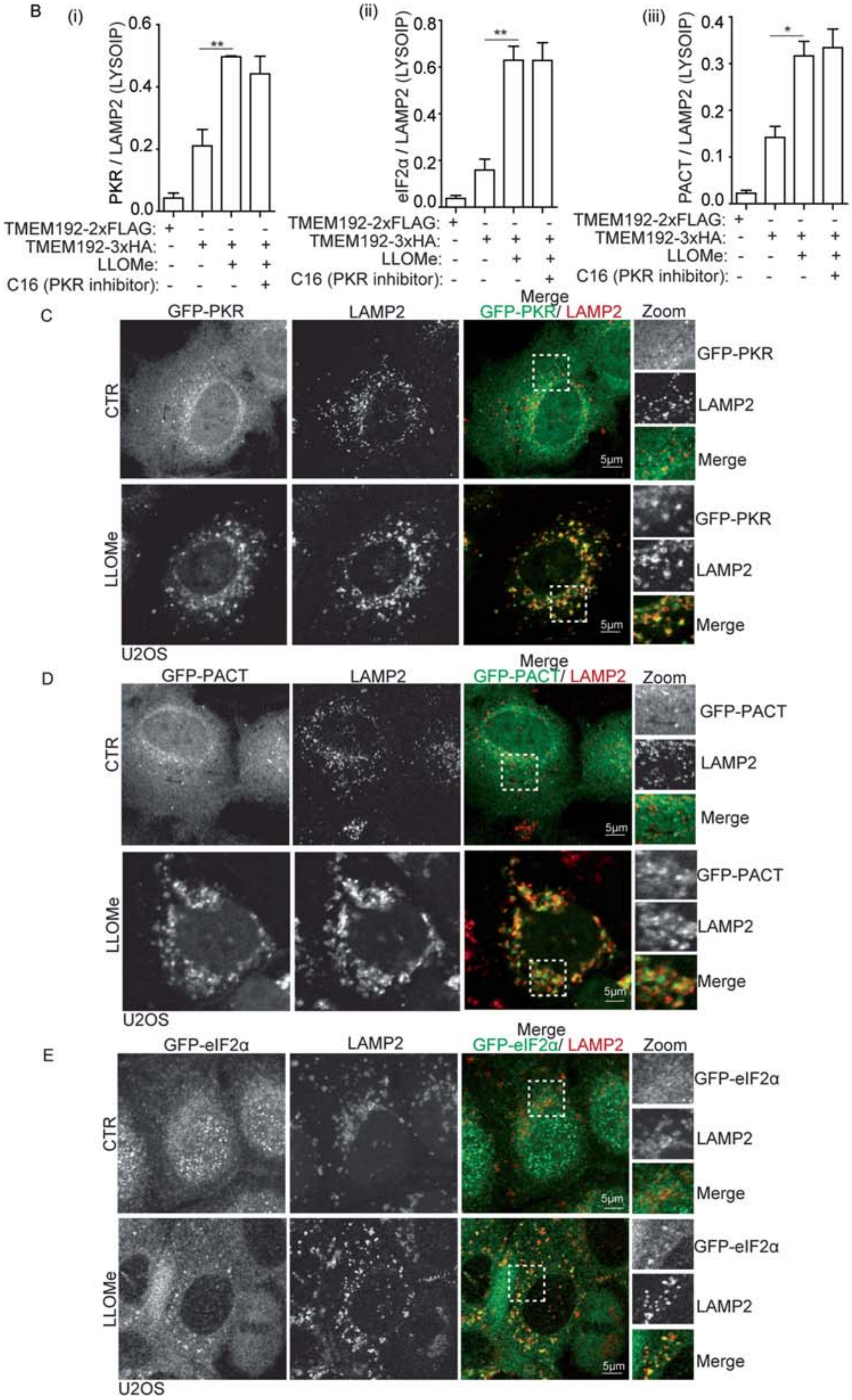

© The Author(s)

Figure EV3. PKR, PACT and eIF2α are associated with damaged lysosomes.

(**A**) Summary of the literature on the detected peptide count of PKR, PACT and eIF2α in the proteomic analysis of lysosomes based on LysoIP LC/MS/MS analysis. (**B**) Quantification of Fig. 3F; the level of PKR, eIF2α and PACT in LysoIP was quantified based on three independent experiments. (**C**) Confocal microscopy imaging of GFP-PKR and LAMP2 in U2OS cells treated with 2 mM LLOMe for 30 min. Scale bar, 5 µm. (**D**) Confocal microscopy imaging of GFP-PACT and LAMP2 in U2OS cells treated with 2 mM LLOMe for 30 min. Scale bar, 5 µm. (**E**) Confocal microscopy imaging of GFP-eIF2α and LAMP2 in U2OS cells treated with 2 mM LLOMe for 30 min. Scale bar, 5 µm. *$p < 0.05$, **$p < 0.01$, ANOVA. See also Fig. 3.

 © The Author(s)

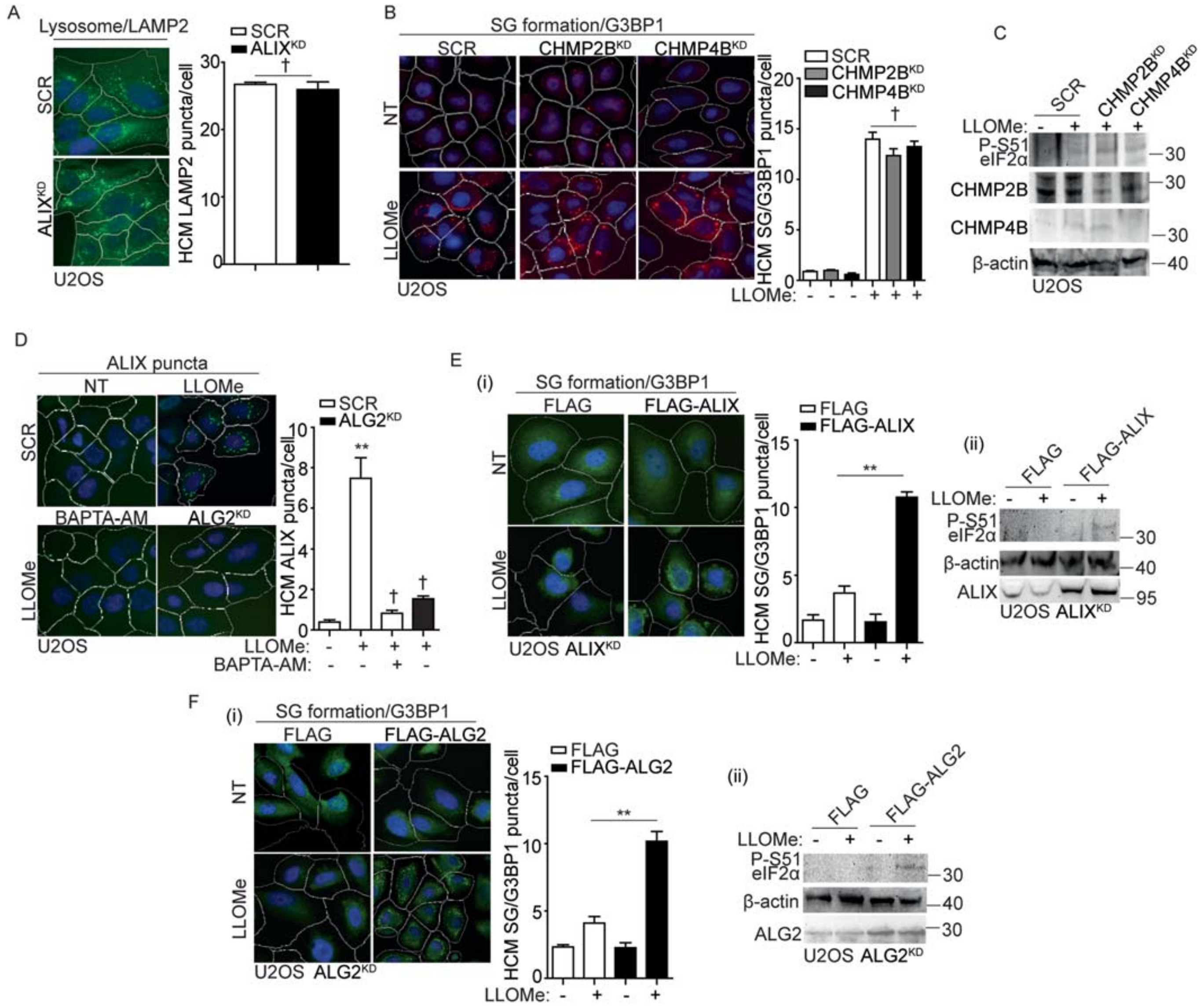

Figure EV4. ALIX regulates stress granule formation during lysosomal damage.

(**A**) Quantification by HCM of LAMP2 in U2OS cells transfected with scrambled siRNA as control (SCR), or ALIX siRNA for knockdown ($ALIX^{KD}$). White masks, algorithm-defined cell boundaries; green masks, computer-identified LAMP2 puncta. (**B**) Quantification by HCM of G3BP1 puncta in U2OS cells transfected with scrambled siRNA as control (SCR), CHMP2B siRNA for knockdown ($CHMP2B^{KD}$) or CHMP4B siRNA for knockdown ($CHMP4B^{KD}$). Cells were treated with 2 mM LLOMe for 30 min. White masks, algorithm-defined cell boundaries; red masks, computer-identified G3BP1 puncta. (**C**) Immunoblot analysis of phosphorylation of eIF2α (S51) in U2OS transfected with scrambled siRNA as control (SCR), CHMP2B siRNA for knockdown ($CHMP2B^{KD}$) or CHMP4B siRNA for knockdown ($CHMP4B^{KD}$), subjected to 2 mM LLOMe treatment for 30 min. (**D**) Quantification by HCM of ALIX puncta in U2OS cells transfected with scrambled siRNA as control (SCR), or ALG2 siRNA for knockdown ($ALG2^{KD}$), or pre-treated with 15 µM BAPTA-AM for 1 h. Cells were treated with 2 mM LLOMe for 30 min. White masks, algorithm-defined cell boundaries; green masks, computer-identified ALIX puncta. (**E**) (i) Quantification by HCM of G3BP1 puncta in U2OS ALIX knockdown cells ($ALIX^{KD}$) overexpressing FLAG or FLAG-ALIX. Cells were treated with 2 mM LLOMe for 30 min. White masks, algorithm-defined cell boundaries; green masks, computer-identified G3BP1 puncta. (ii) Immunoblot analysis of phosphorylation of eIF2α (S51) in U2OS cells as described in (i). (**F**) (i) Quantification by HCM of G3BP1 puncta in U2OS ALG2 knockdown cells ($ALG2^{KD}$) overexpressing FLAG or FLAG-ALG2. Cells were treated with 2 mM LLOMe for 30 min. White masks, algorithm-defined cell boundaries; green masks, computer-identified G3BP1 puncta. (ii) Immunoblot analysis of phosphorylation of eIF2α (S51) in U2OS cells as described in (i). NT, untreated cells. Data, means ± SEM ($n = 3$); HCM: $n \geq 3$ (each experiment: 500 valid primary objects/cells per well, ≥5 wells/sample). †$p \geq 0.05$ (not significant), **$p < 0.01$, ANOVA. See also Fig. 4.

© The Author(s)

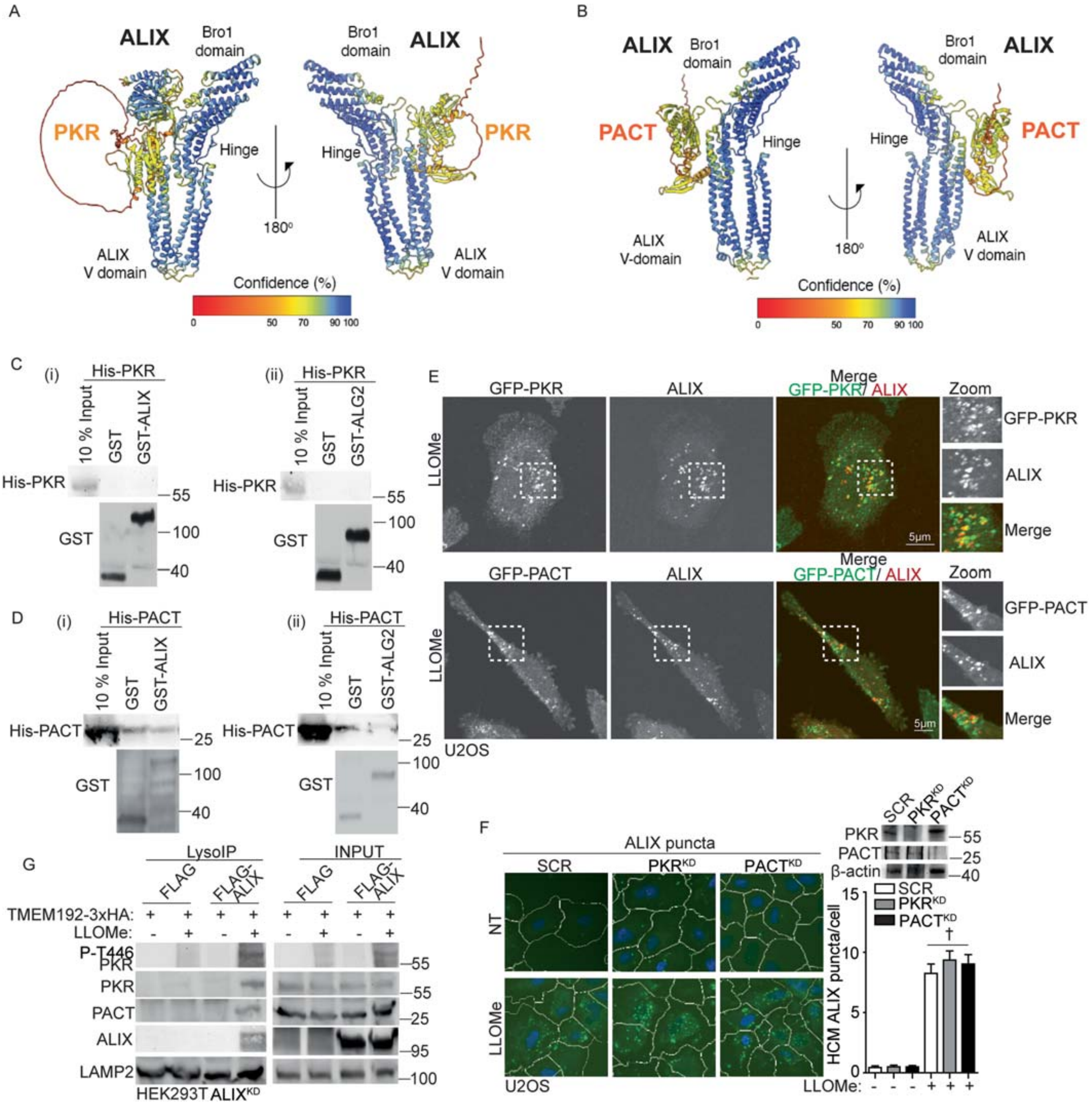

Figure EV5. PKR and PACT associate with ALIX during lysosomal damage.

(**A**) AlphaFold 2 predicted the interaction between PKR and ALIX, with the C-terminal PRD domain removed. (**B**) AlphaFold 2 predicted the interaction between PACT and ALIX, with the C-terminal PRD domain removed. (**C**) GST pulldown assay of in vitro translated His-tagged PKR with GST or GST-tagged ALIX (i) or ALG2 (ii) in the presence of 10 μM $CaCl_2$. (**D**) GST pulldown assay of in vitro translated His-tagged PACT with GST or GST-tagged ALIX (i) or ALG2 (ii) in the presence of 10 μM $CaCl_2$. (**E**) Confocal microscopy imaging of GFP-PKR/PACT and ALIX in U2OS cells treated with 2 mM LLOMe for 30 min. Scale bar, 5 μm. (**F**) Quantification by HCM of ALIX puncta in U2OS cells transfected with scrambled siRNA as control (SCR), PKR siRNA for knockdown (PKR^{KD}), or PACT siRNA for knockdown (PACT^{KD}). Cells were treated with 2 mM LLOMe for 30 min. White masks, algorithm-defined cell boundaries; green masks, computer-identified ALIX puncta. (**G**) Analysis of proteins associated with purified lysosomes (LysoIP; TMEM192-3xHA) from HEK293T ALIX knockdown cells (ALIX^{KD}) overexpressing FLAG or FLAG-ALIX. Cells were treated with 1 mM LLOMe for 1 h. NT, untreated cells. Data, means ± SEM ($n = 3$); HCM: $n \geq 3$ (each experiment: 500 valid primary objects/cells per well, ≥5 wells/sample). †$p \geq 0.05$ (not significant), ANOVA. See also Fig. 5.

 © The Author(s)
